# Supplementary material for: YIPF3 and YIPF4 regulate autophagic turnover of the Golgi apparatus
Source: EMBO J. 2024 May 31;43(14):8. doi: 10.1038/s44318-024-00131-3 (PMC11250848; doi:10.1038/s44318-024-00131-3)
Supplement: Supplementary file 1 — Appendix [file 44318_2024_131_MOESM1_ESM.pdf]

YIPF3 and YIPF4 regulate autophagic turnover of the Golgi apparatus

Shinri Kitta, Tatsuya Kaminishi, Momoko Higashi, Takayuki Shima, Kohei Nishino, Nobuhiro Nakamura, Hidetaka Kosako, Tamotsu Yoshimori*, Akiko Kuma*

*Corresponding author: Tamotsu Yoshimori, tamyoshi@fbs.osaka-u.ac.jp
Akiko Kuma, kuma@fbs.osaka-u.ac.jp

Table of contents

Page	Content
2	**Appendix Figure S1**. The Golgi morphology is not changed in cells stably coexpressing YIPF3 and YIPF4

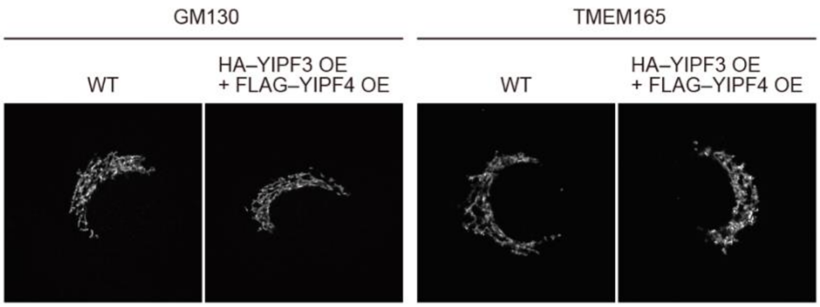

Appendix Figure S1. The Golgi morphology is not changed in cells stably coexpressing YIPF3 and YIPF4

Immunofluorescence image of WT MEFs and MEFs stably coexpressing HA-YIPF3 and FLAG-YIPF4 under growing conditions. Cells were stained with anti-GM130 or anti-TMEM165 antibodies. Scale bars, 10 μm.
