# Supplementary figures and images for: YIPF3 and YIPF4 regulate autophagic turnover of the Golgi apparatus (part 1 of 2)
Source: EMBO J. 2024 May 31;43(14):8. doi: 10.1038/s44318-024-00131-3 (PMC11250848; doi:10.1038/s44318-024-00131-3)

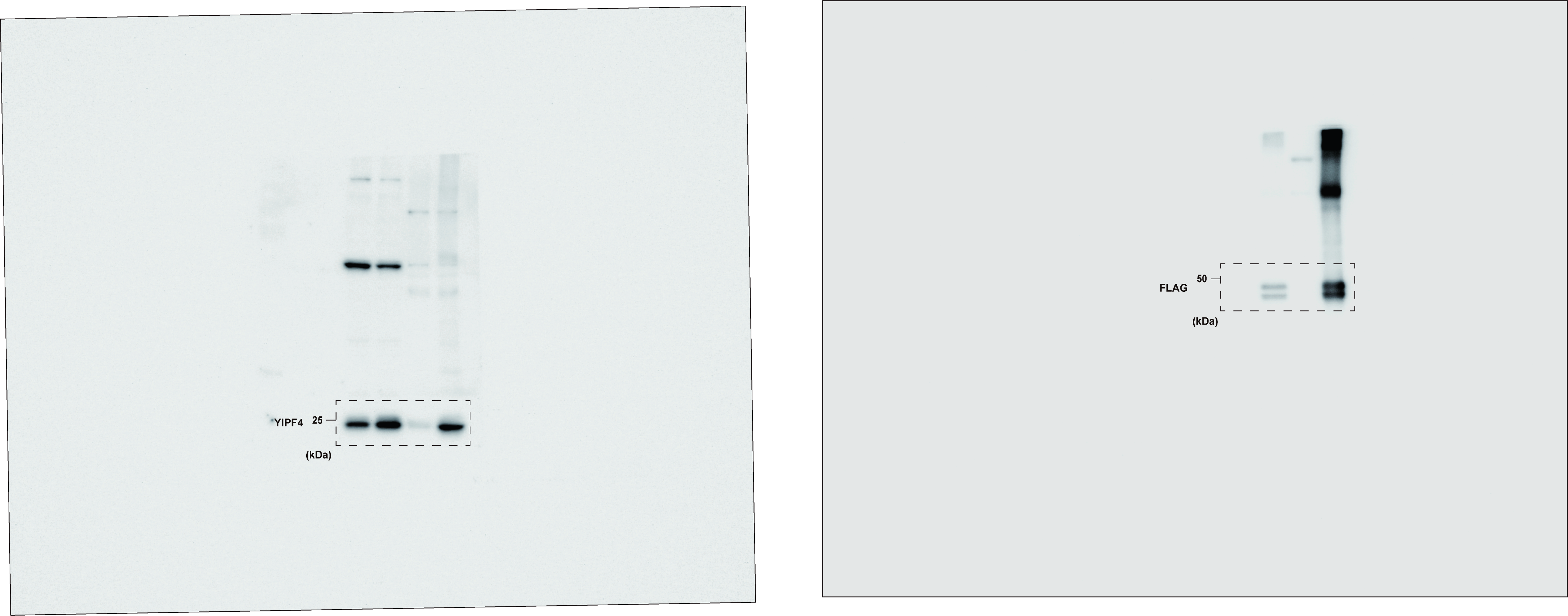

Supplement: Supplementary file 4 — Source data Fig. 1 [file 44318_2024_131_MOESM4_ESM.zip › Figure 1/1C/western_Figure1C.tif]

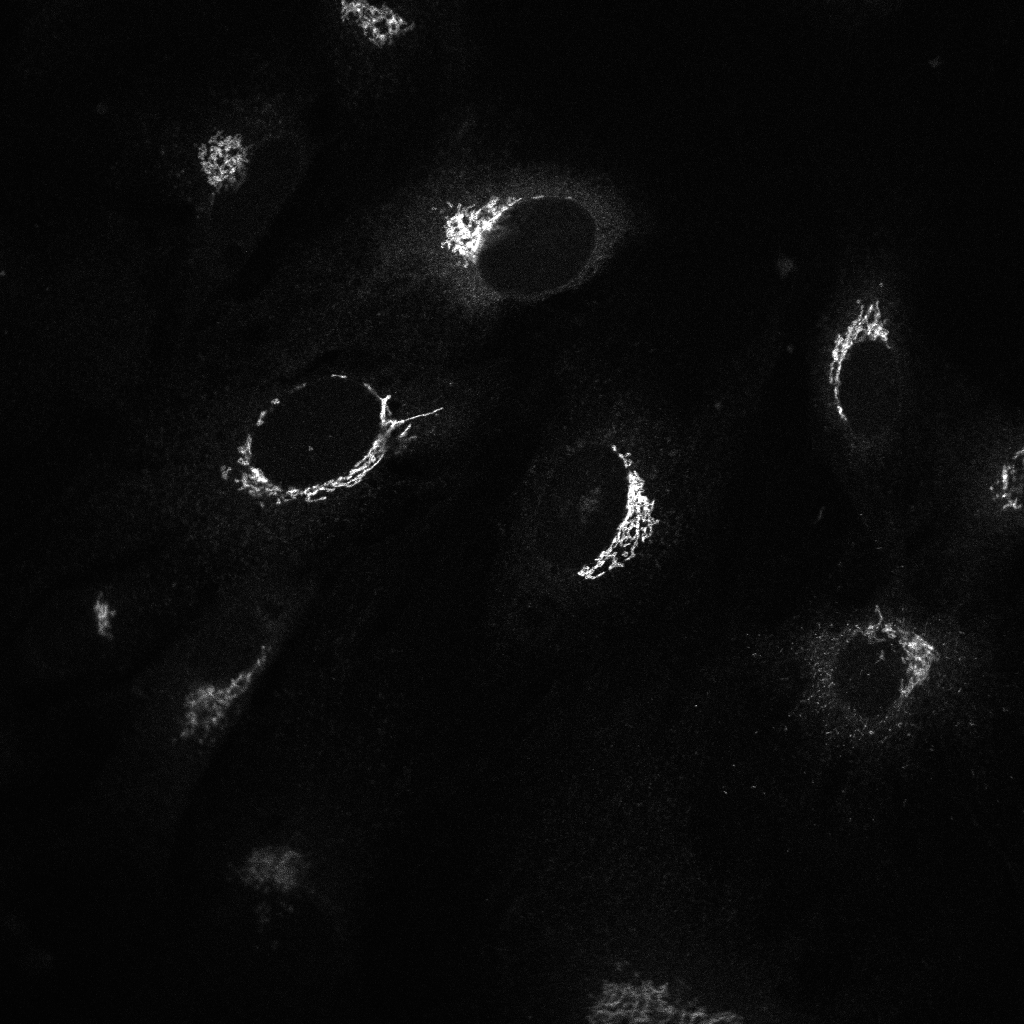

Supplement: Supplementary file 5 — Source data Fig. 2 [file 44318_2024_131_MOESM5_ESM.zip › Figure 2/2A/Figure2A_Growing_EGFP-YIPF3.tif]

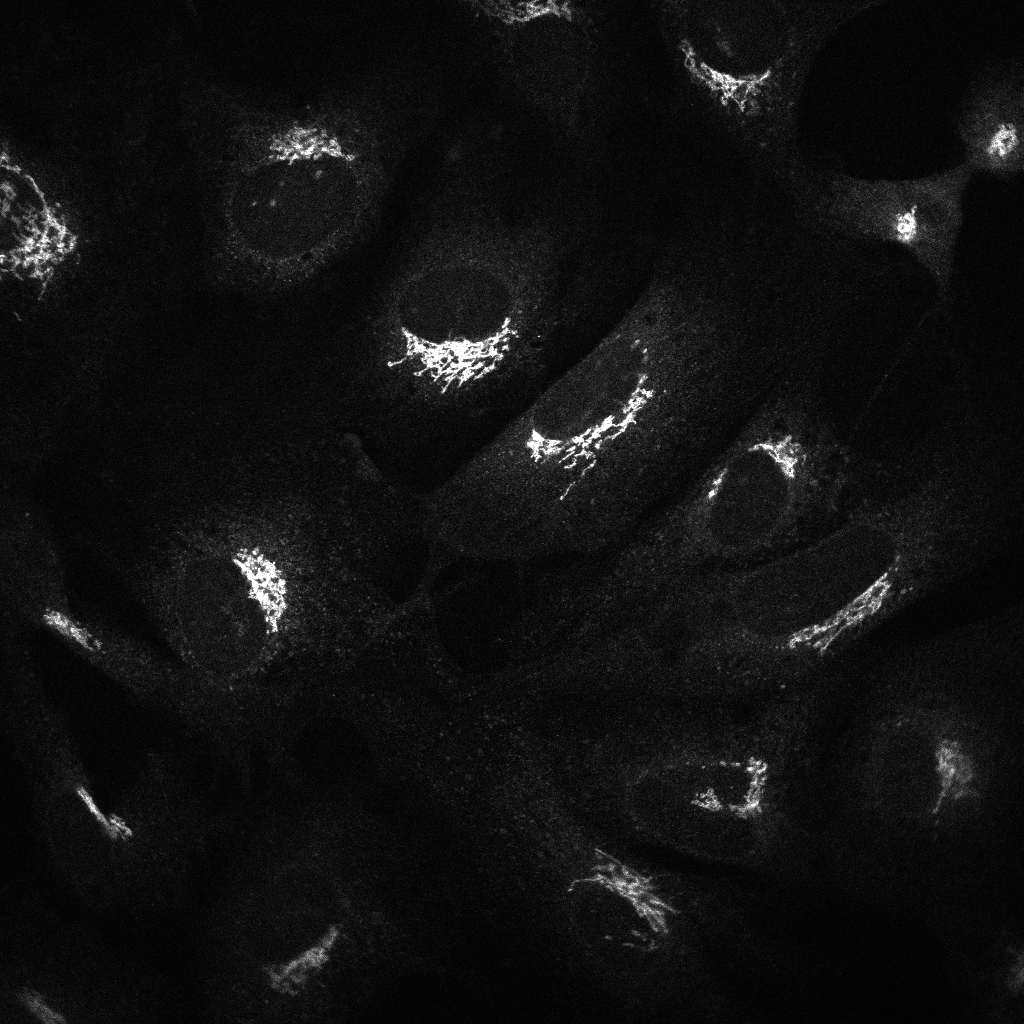

Supplement: Supplementary file 5 — Source data Fig. 2 [file 44318_2024_131_MOESM5_ESM.zip › Figure 2/2A/Figure2A_Growing_EGFP-YIPF4.tif]

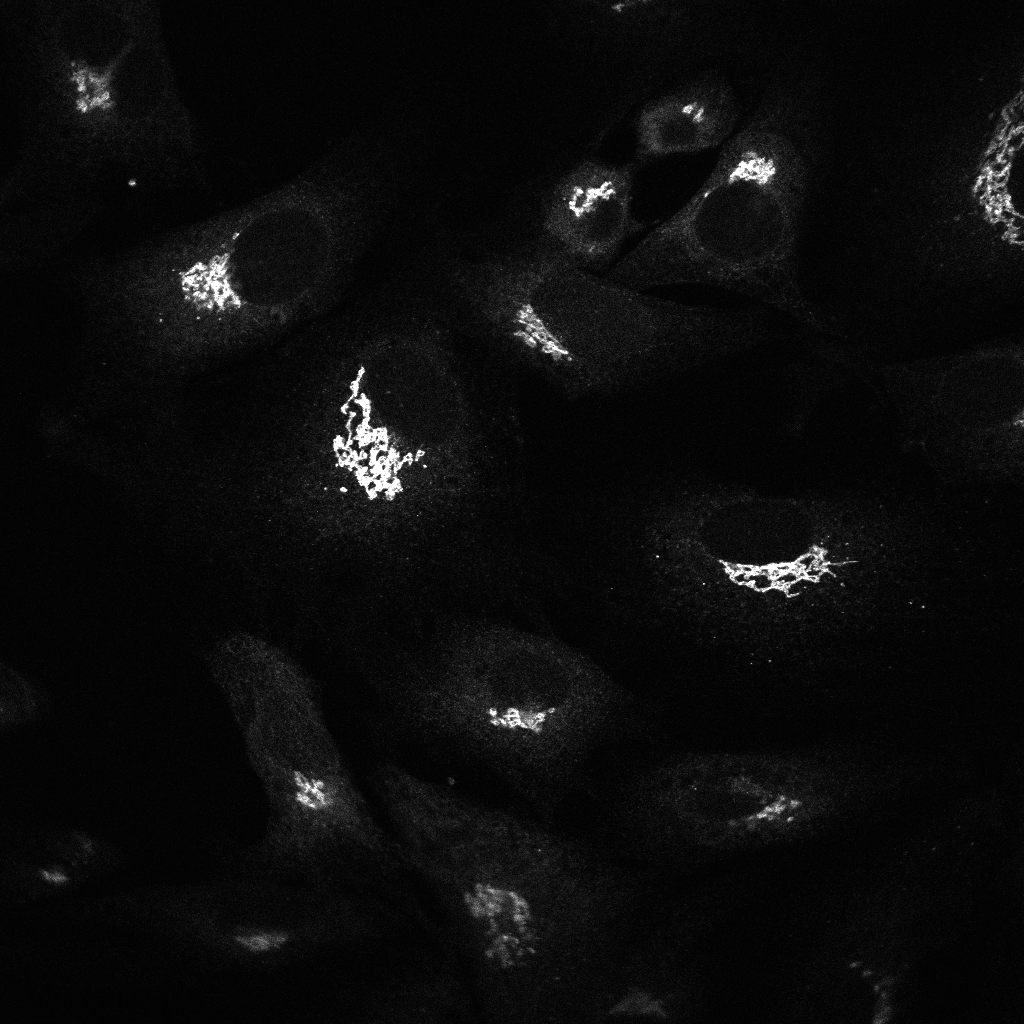

Supplement: Supplementary file 5 — Source data Fig. 2 [file 44318_2024_131_MOESM5_ESM.zip › Figure 2/2A/Figure2A_GrowingBafA1_EGFP-YIPF3.tif]

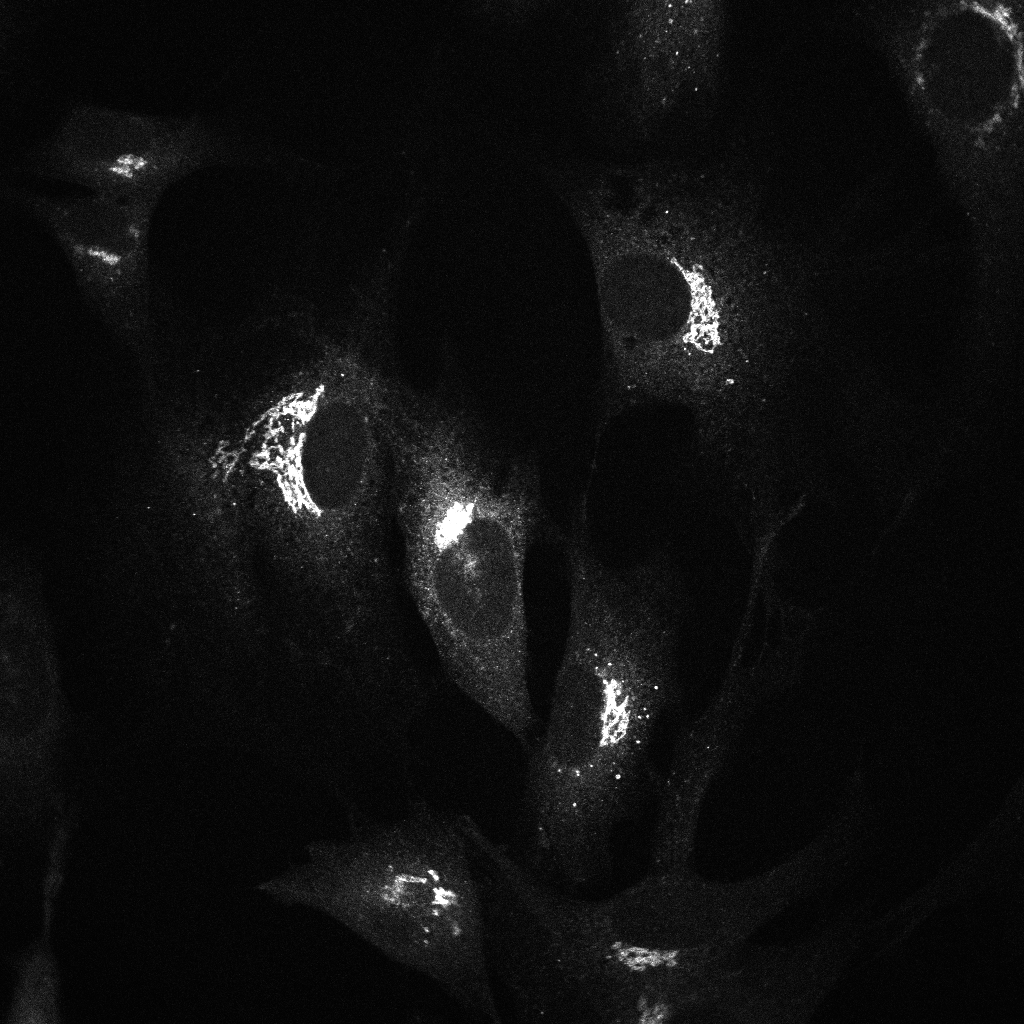

Supplement: Supplementary file 5 — Source data Fig. 2 [file 44318_2024_131_MOESM5_ESM.zip › Figure 2/2A/Figure2A_GrowingBafA1_EGFP-YIPF4.tif]

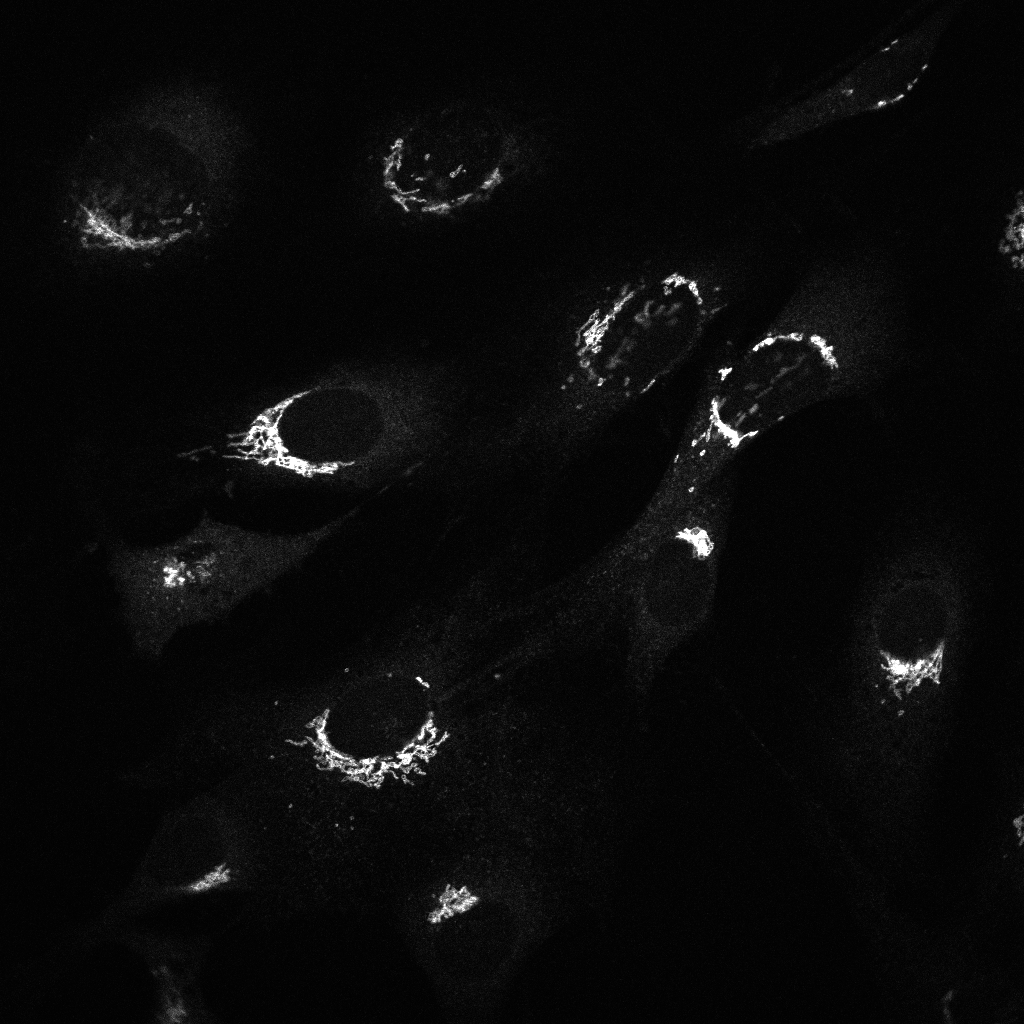

Supplement: Supplementary file 5 — Source data Fig. 2 [file 44318_2024_131_MOESM5_ESM.zip › Figure 2/2A/Figure2A_Starvation_EGFP-YIPF3.tif]

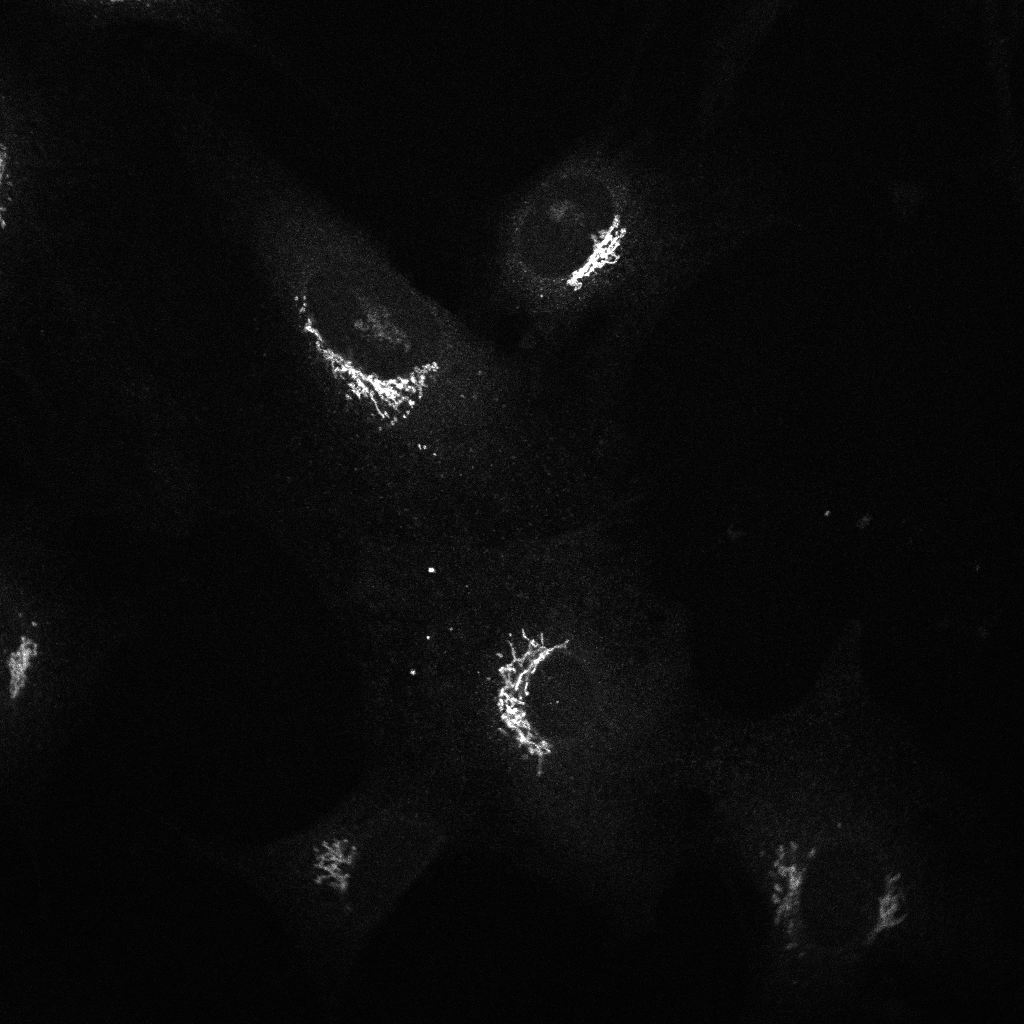

Supplement: Supplementary file 5 — Source data Fig. 2 [file 44318_2024_131_MOESM5_ESM.zip › Figure 2/2A/Figure2A_Starvation_EGFP-YIPF4.tif]

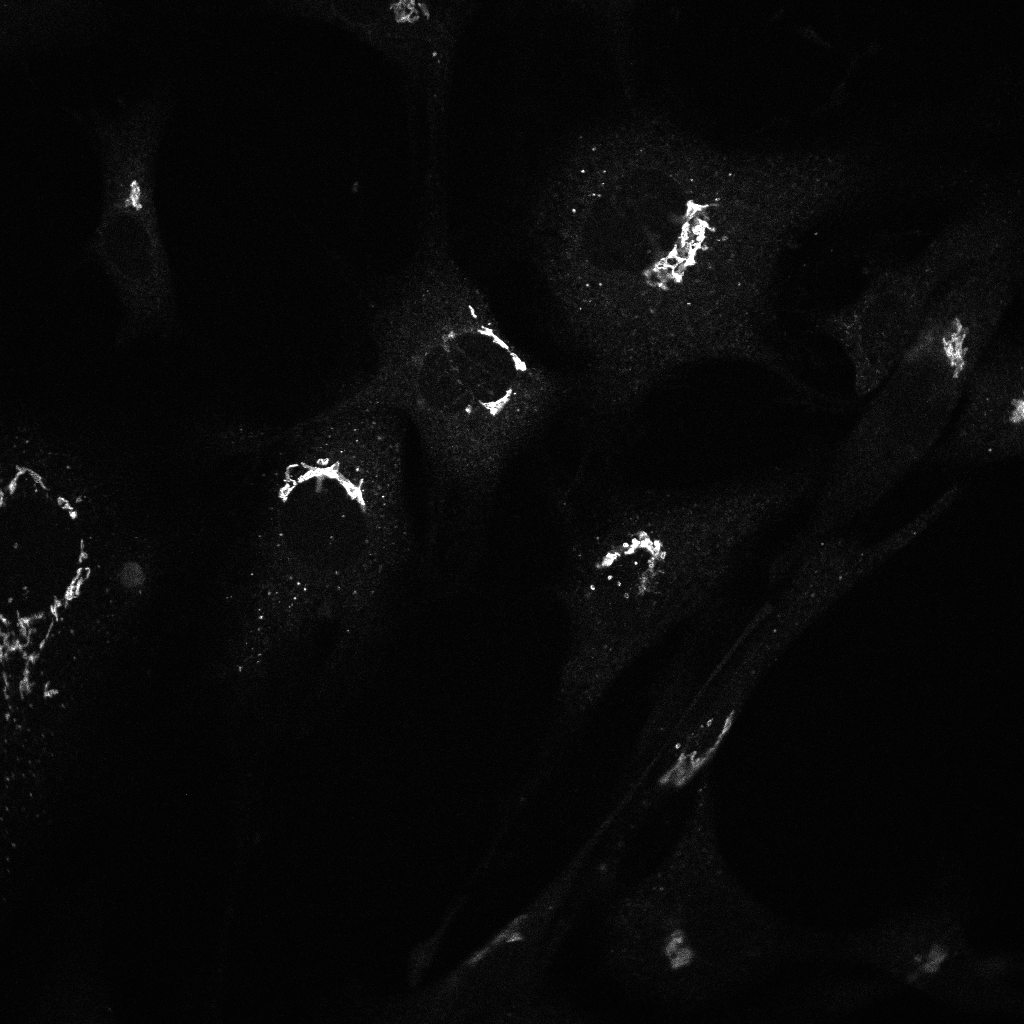

Supplement: Supplementary file 5 — Source data Fig. 2 [file 44318_2024_131_MOESM5_ESM.zip › Figure 2/2A/Figure2A_StarvationBafA1_EGFP-YIPF3.tif]

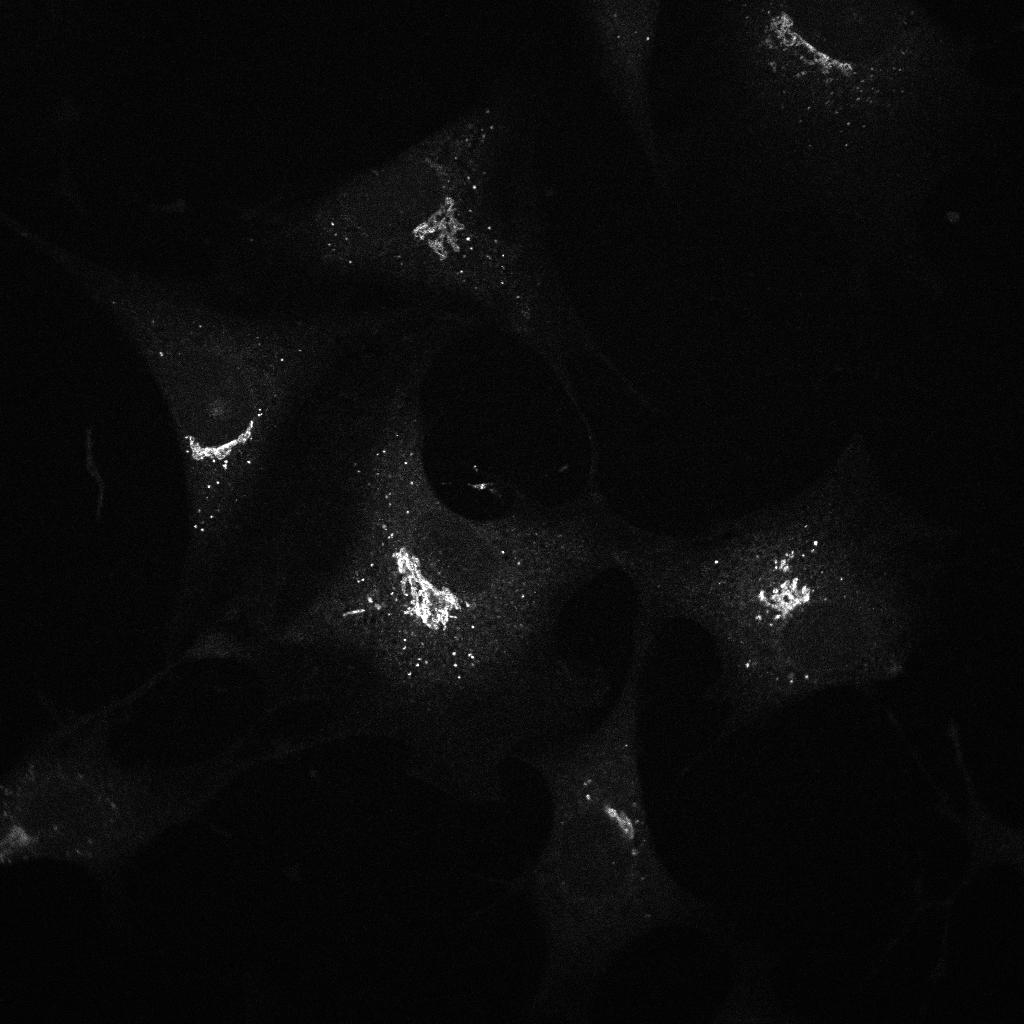

Supplement: Supplementary file 5 — Source data Fig. 2 [file 44318_2024_131_MOESM5_ESM.zip › Figure 2/2A/Figure2A_StarvationBafA1_EGFP-YIPF4.tif]

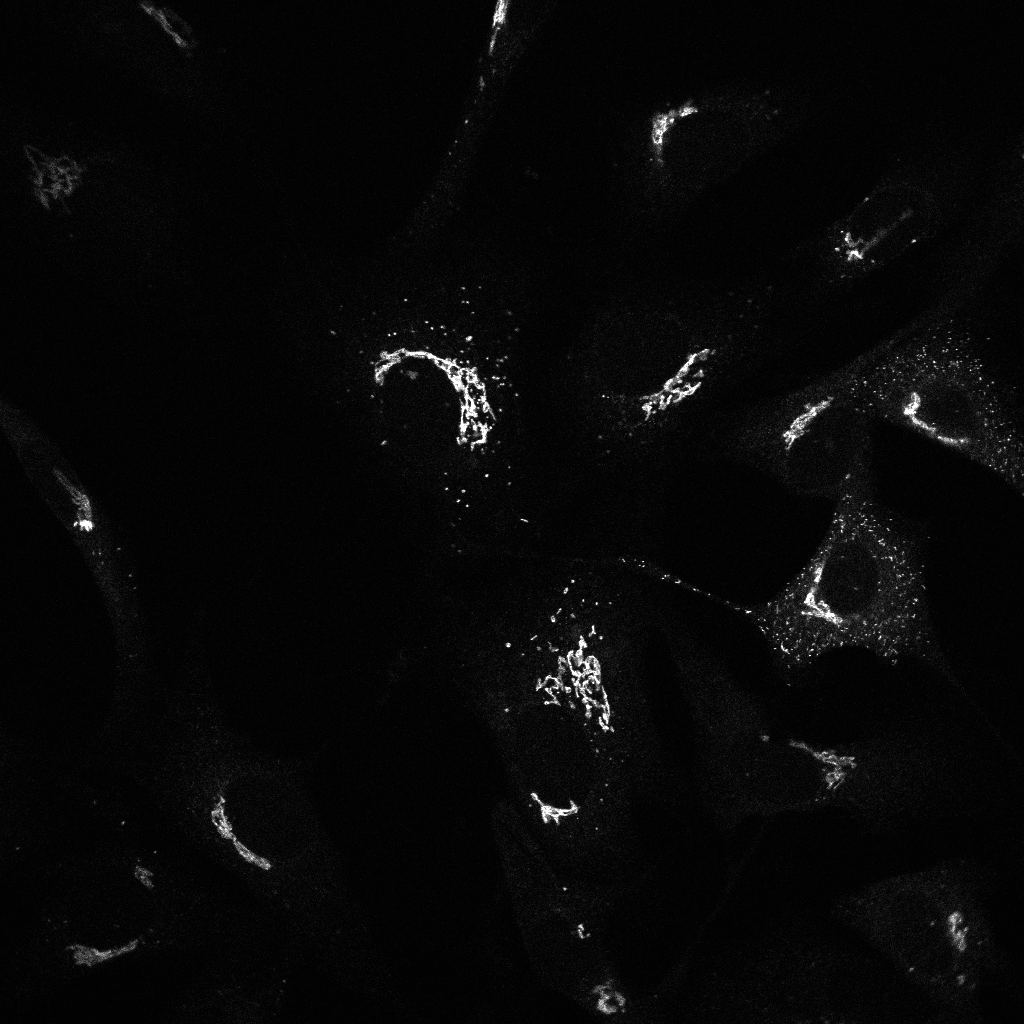

Supplement: Supplementary file 5 — Source data Fig. 2 [file 44318_2024_131_MOESM5_ESM.zip › Figure 2/2C/Figure2C_EGFP-YIPF3_EGFP.tif]

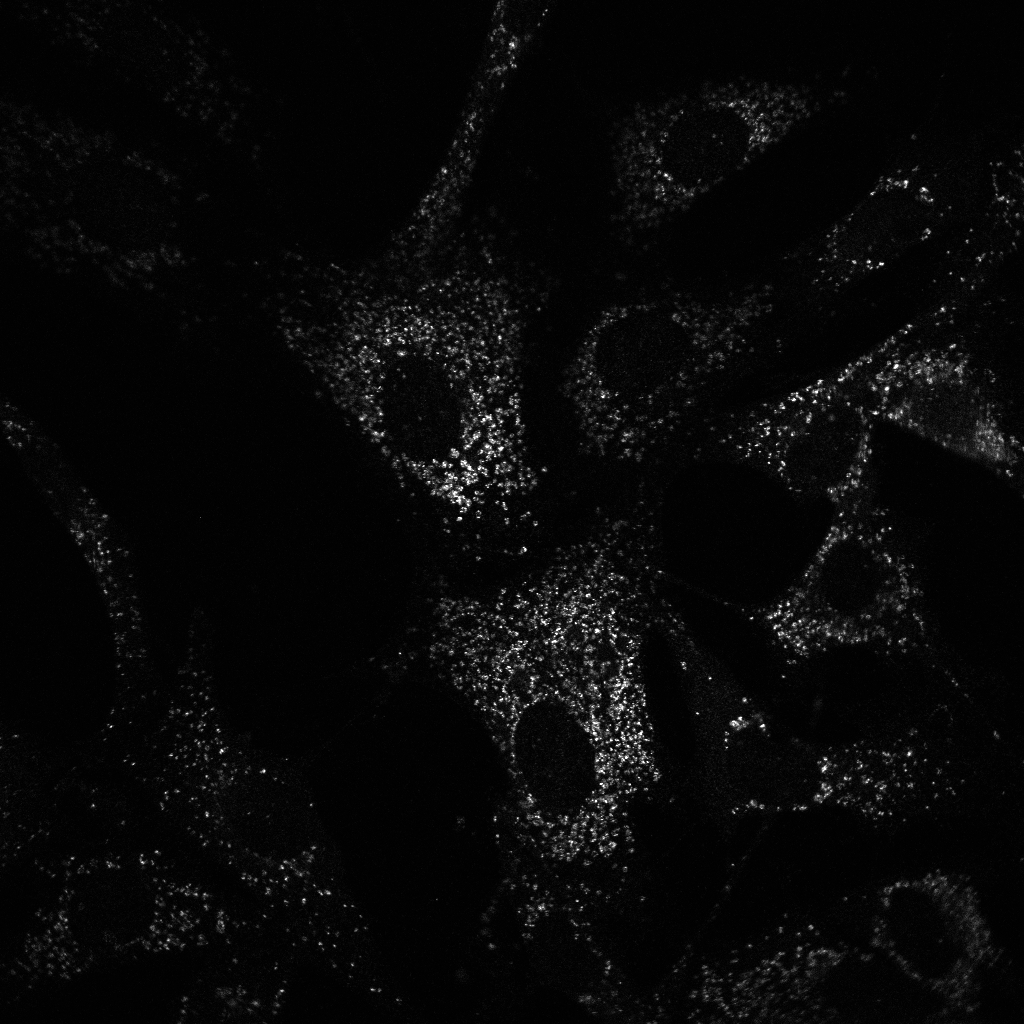

Supplement: Supplementary file 5 — Source data Fig. 2 [file 44318_2024_131_MOESM5_ESM.zip › Figure 2/2C/Figure2C_EGFP-YIPF3_LC3.tif]

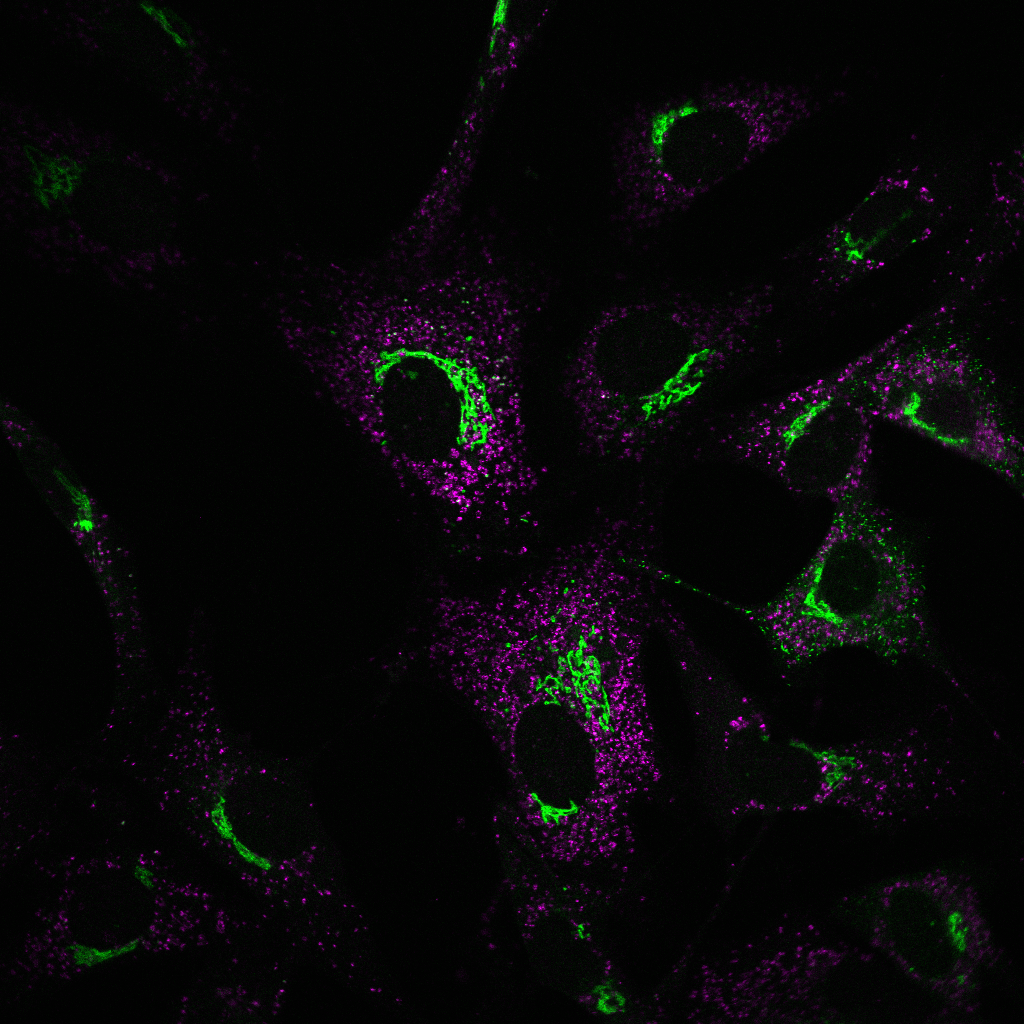

Supplement: Supplementary file 5 — Source data Fig. 2 [file 44318_2024_131_MOESM5_ESM.zip › Figure 2/2C/Figure2C_EGFP-YIPF3_merge.tif]

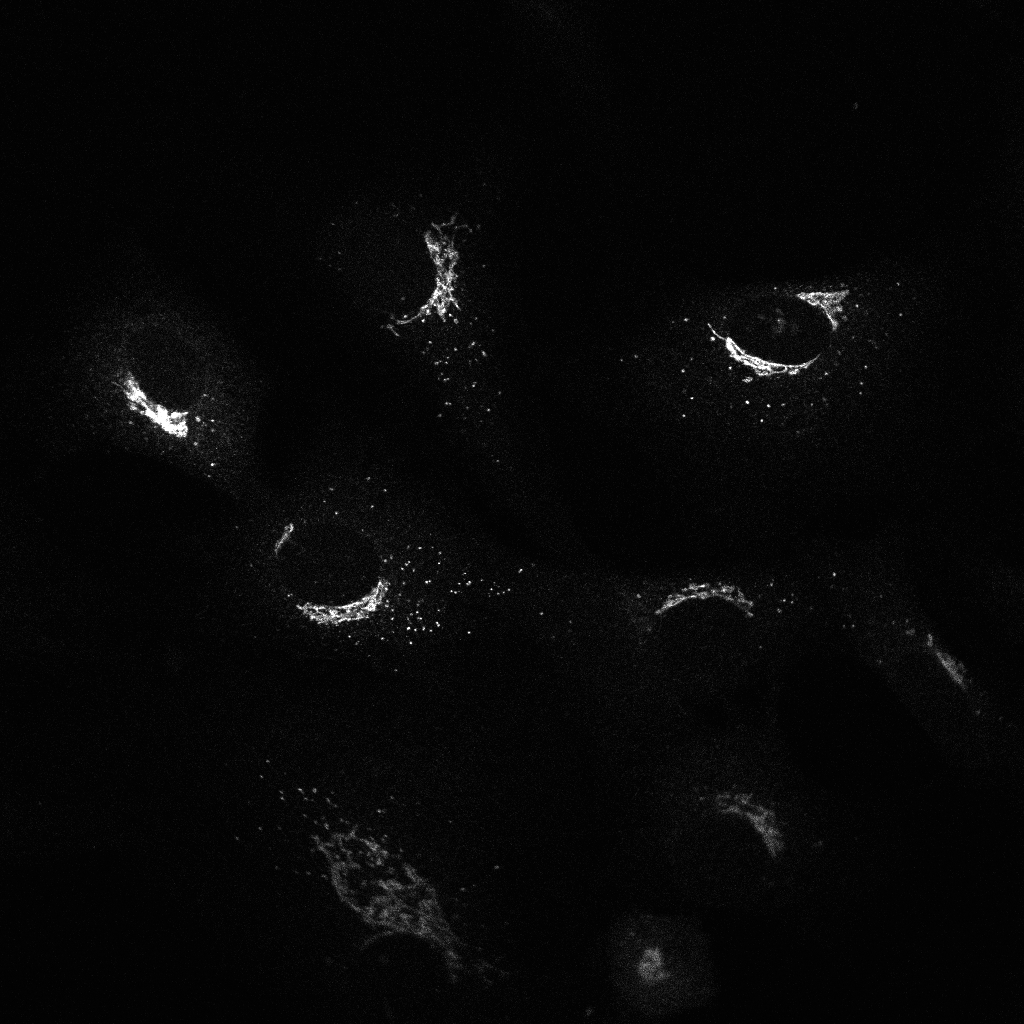

Supplement: Supplementary file 5 — Source data Fig. 2 [file 44318_2024_131_MOESM5_ESM.zip › Figure 2/2C/Figure2C_EGFP-YIPF4_EGFP.tif]

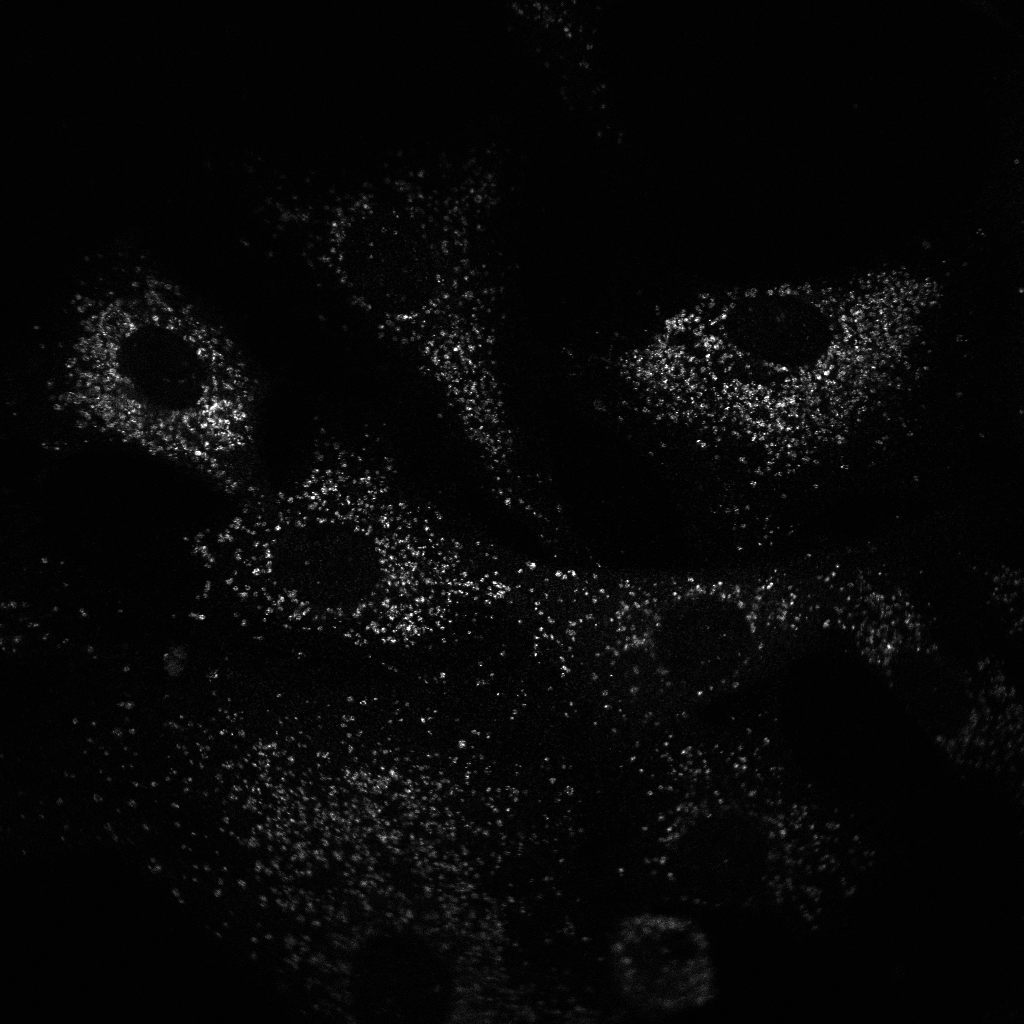

Supplement: Supplementary file 5 — Source data Fig. 2 [file 44318_2024_131_MOESM5_ESM.zip › Figure 2/2C/Figure2C_EGFP-YIPF4_LC3.tif]

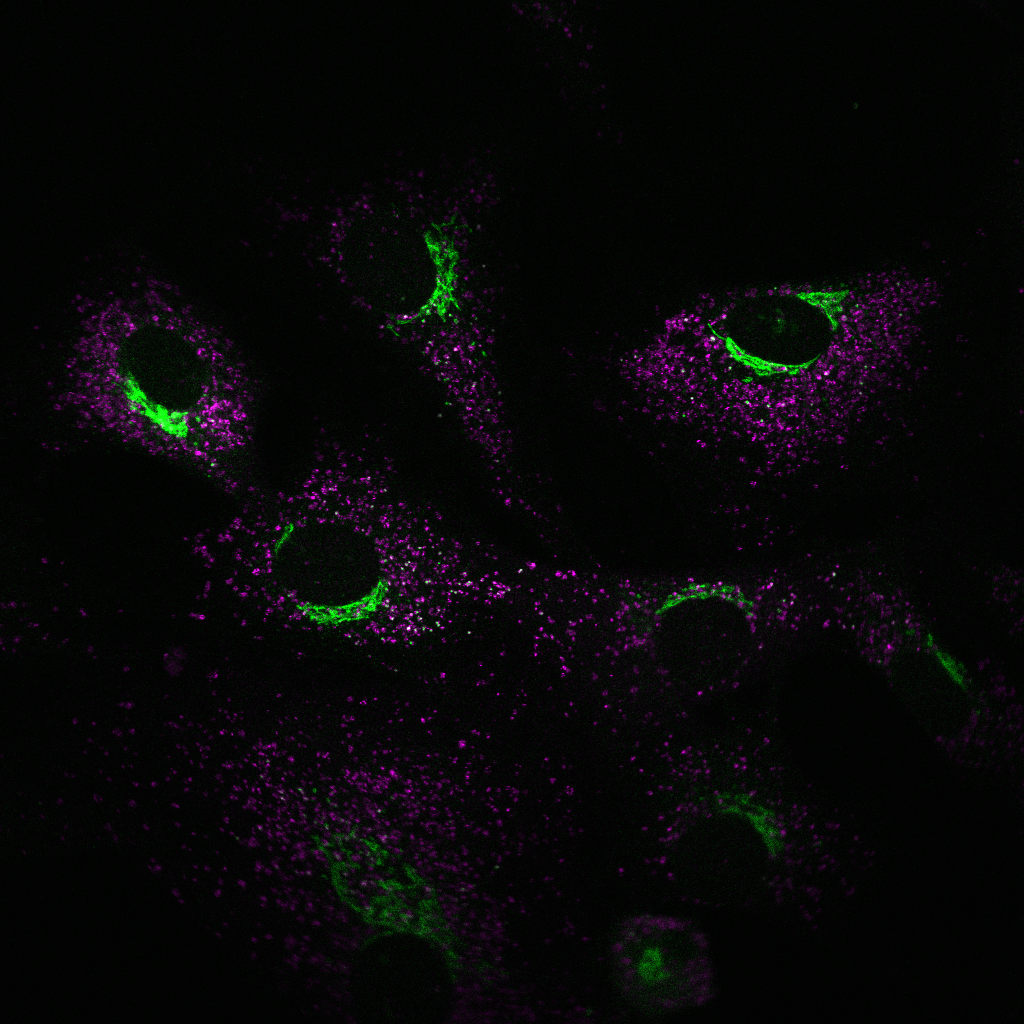

Supplement: Supplementary file 5 — Source data Fig. 2 [file 44318_2024_131_MOESM5_ESM.zip › Figure 2/2C/Figure2C_EGFP-YIPF4_merge.tif]

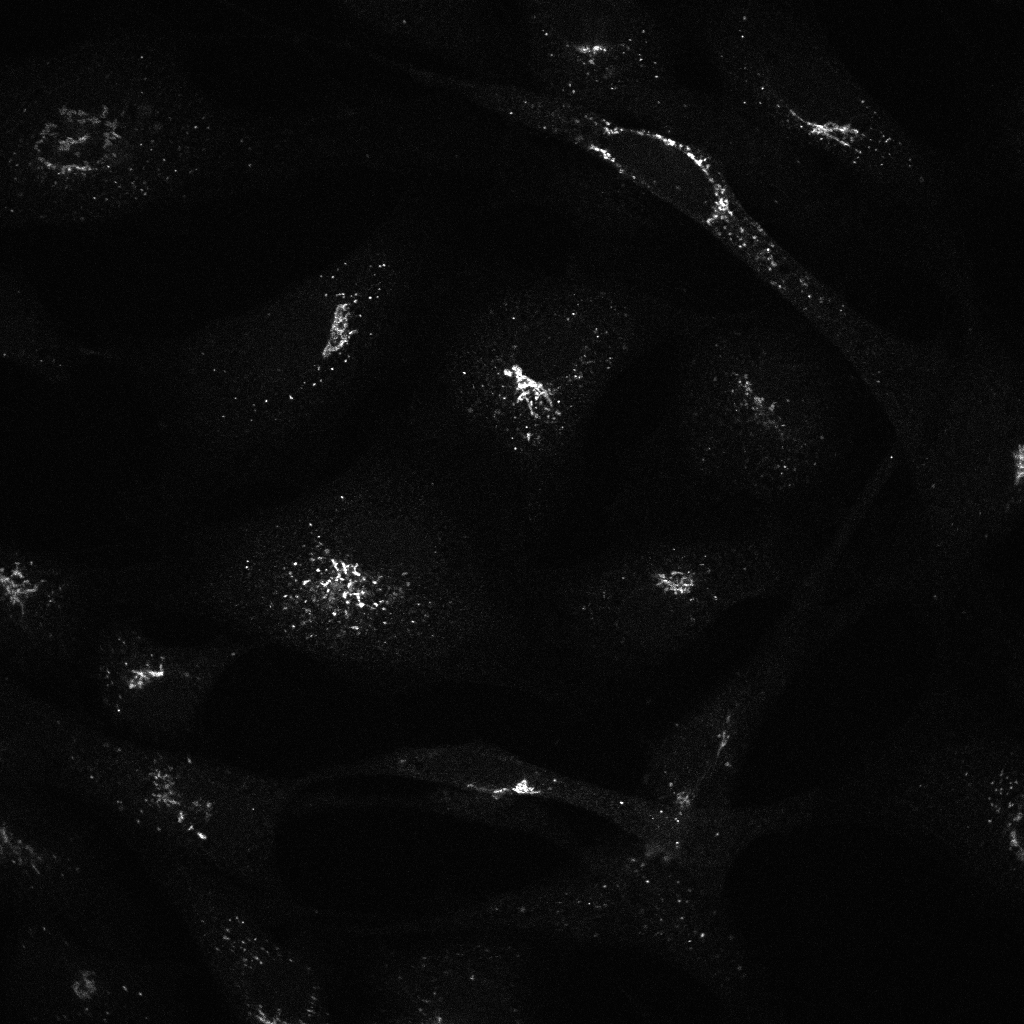

Supplement: Supplementary file 5 — Source data Fig. 2 [file 44318_2024_131_MOESM5_ESM.zip › Figure 2/2D/Figure2D_EGFP-YIPF4_EGFP.tif]

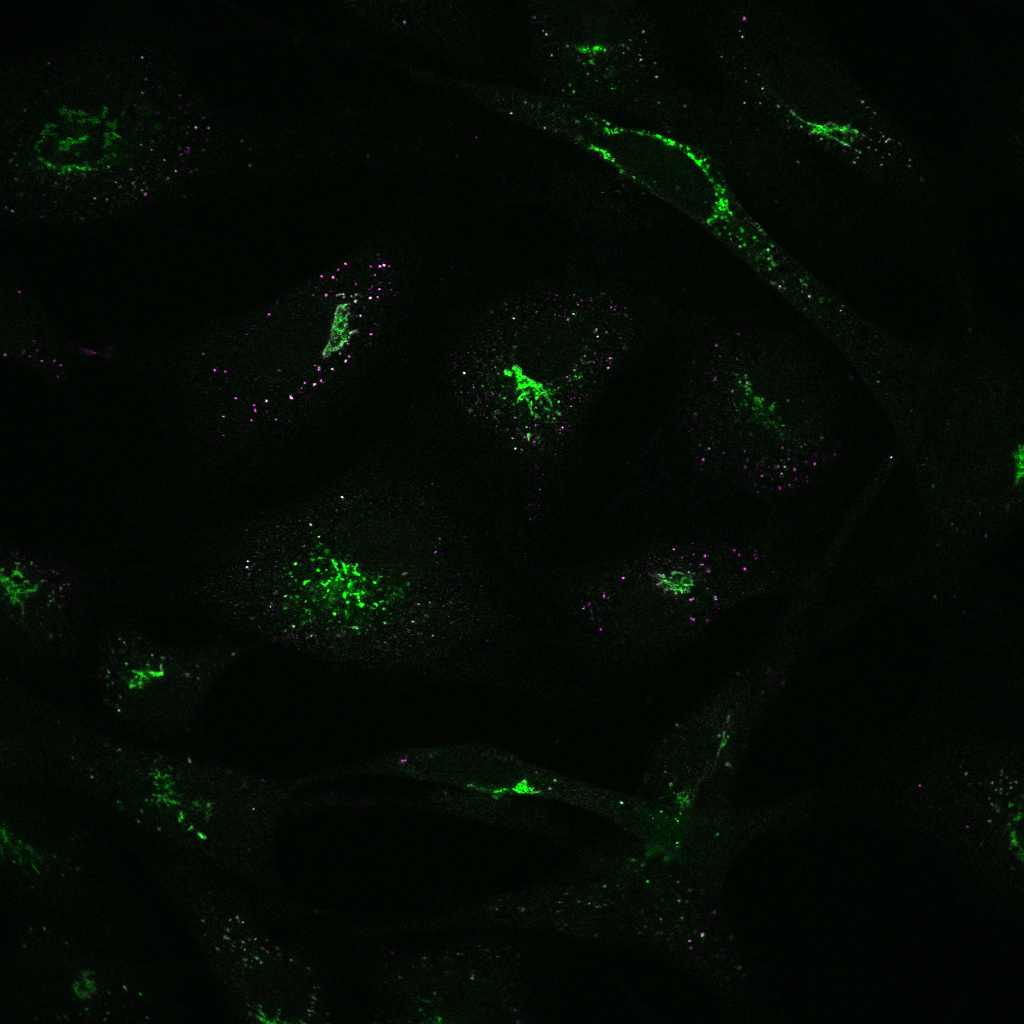

Supplement: Supplementary file 5 — Source data Fig. 2 [file 44318_2024_131_MOESM5_ESM.zip › Figure 2/2D/Figure2D_EGFP-YIPF4_merge.tif]

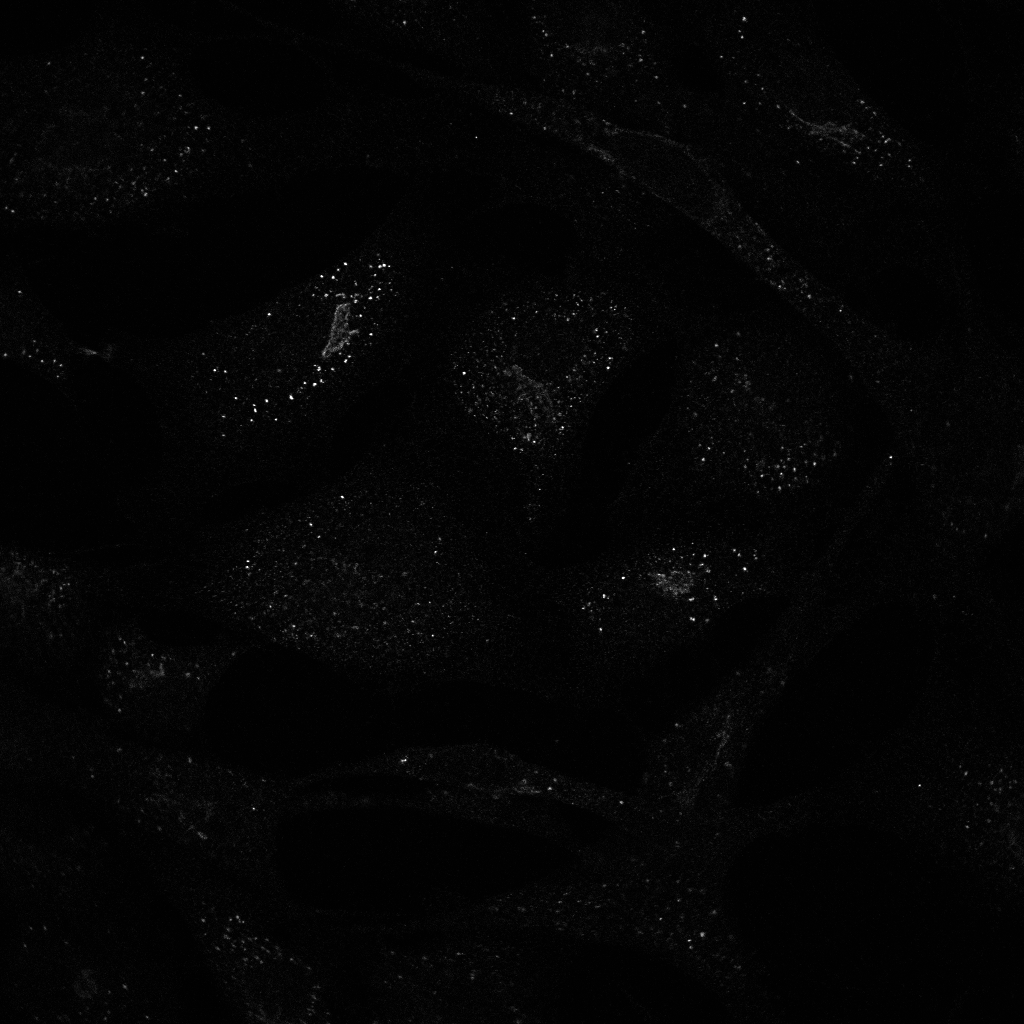

Supplement: Supplementary file 5 — Source data Fig. 2 [file 44318_2024_131_MOESM5_ESM.zip › Figure 2/2D/Figure2D_EGFP-YIPF4_YIPF3.tif]

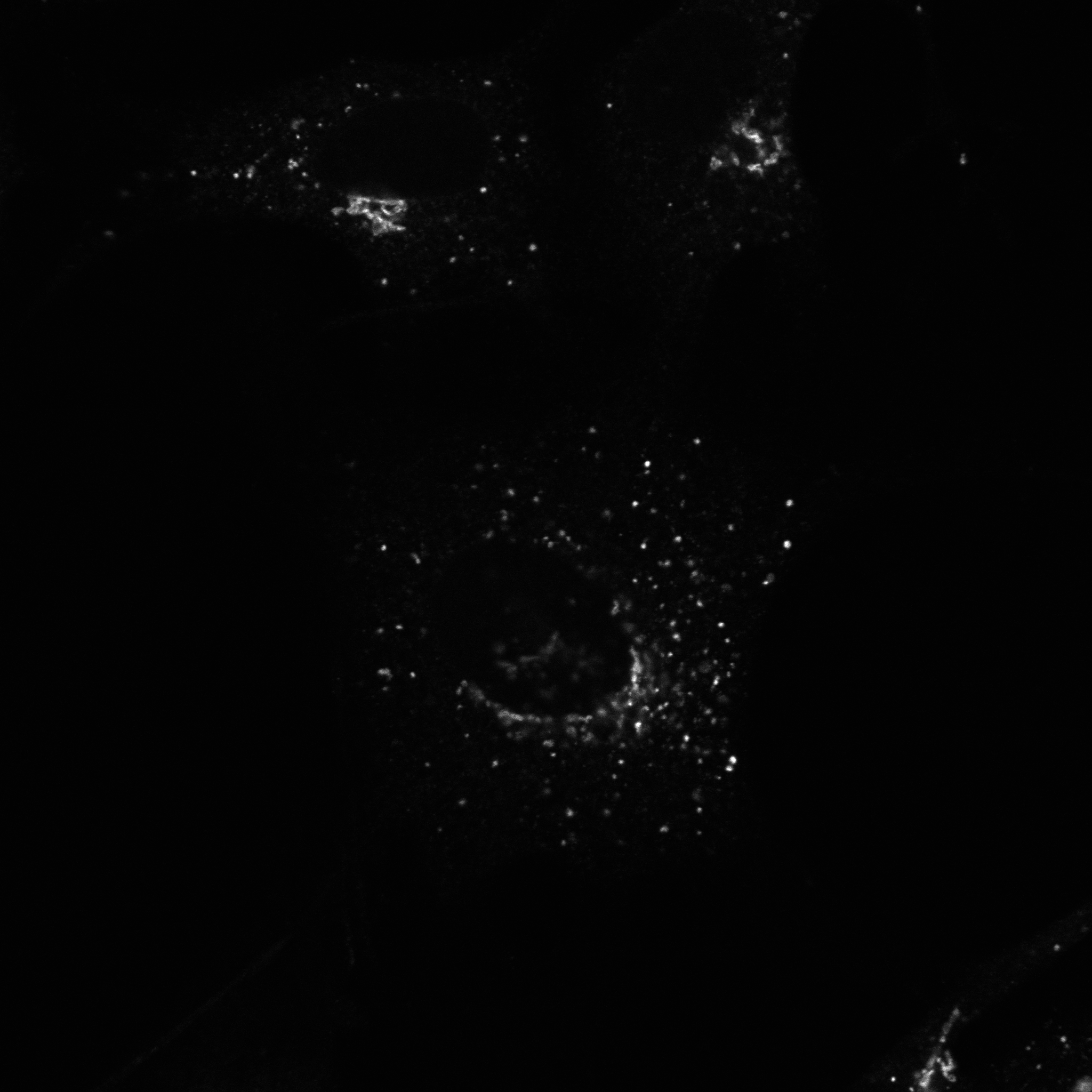

Supplement: Supplementary file 5 — Source data Fig. 2 [file 44318_2024_131_MOESM5_ESM.zip › Figure 2/2E/Figure2E_GM130_EGFP.tif]

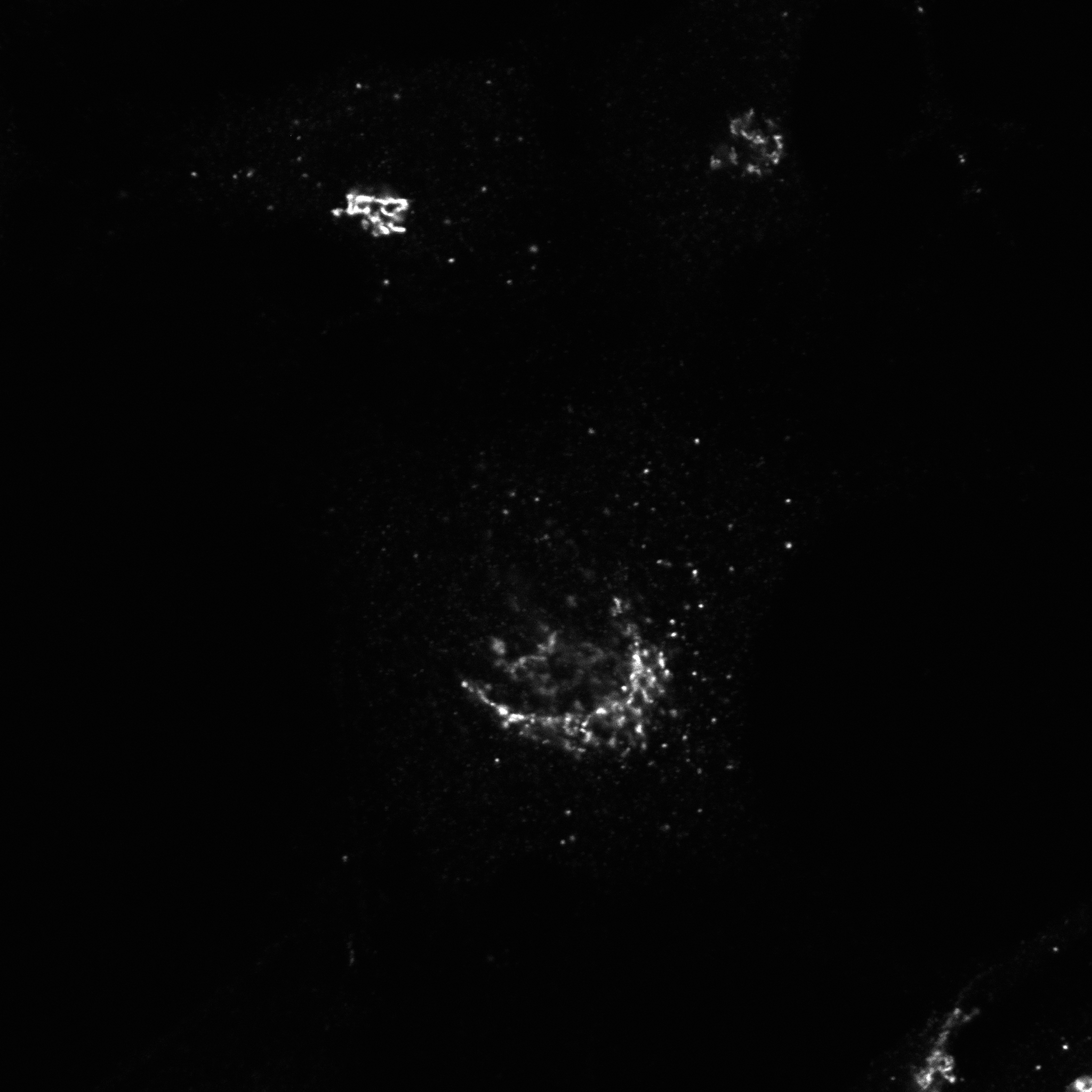

Supplement: Supplementary file 5 — Source data Fig. 2 [file 44318_2024_131_MOESM5_ESM.zip › Figure 2/2E/Figure2E_GM130_GM130.tif]

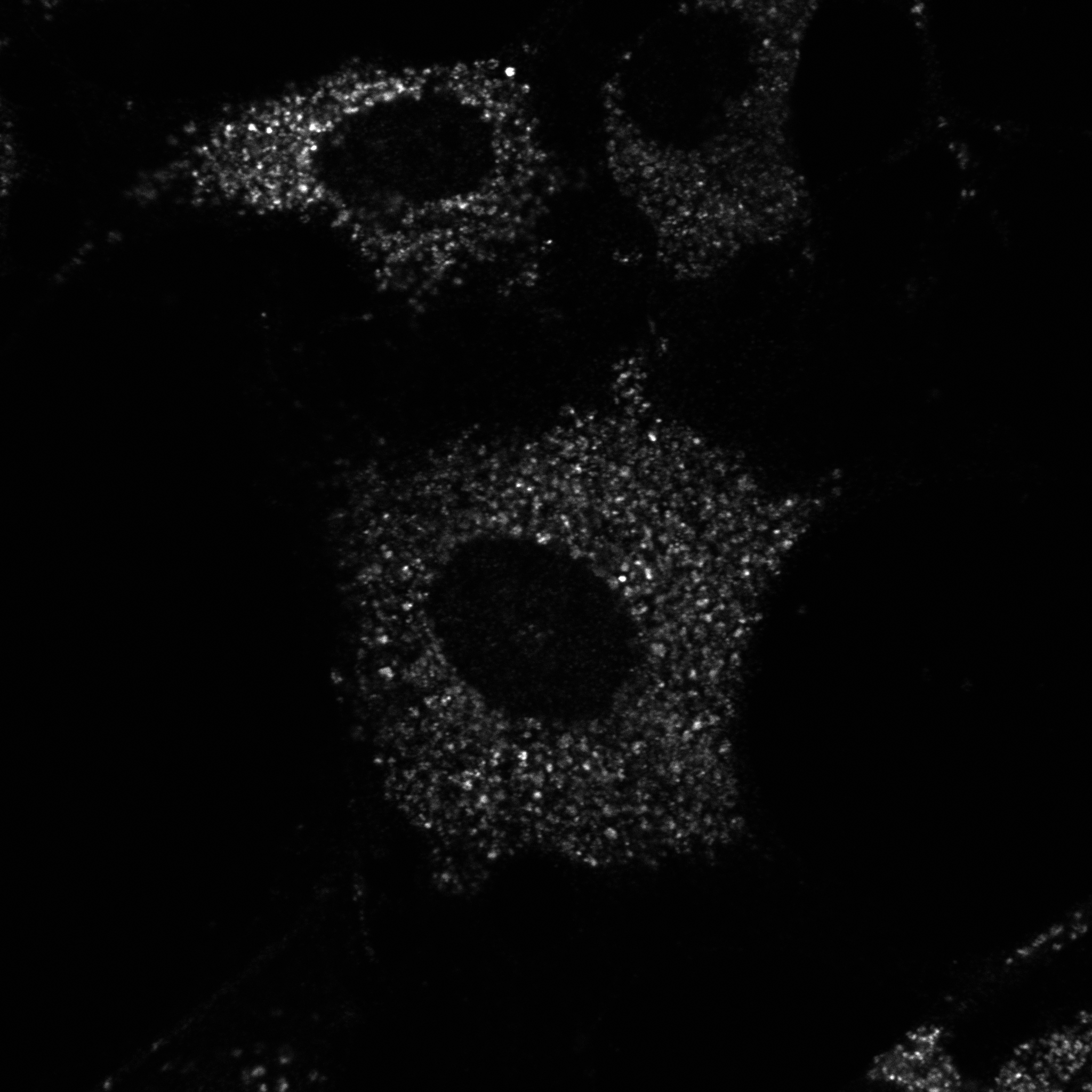

Supplement: Supplementary file 5 — Source data Fig. 2 [file 44318_2024_131_MOESM5_ESM.zip › Figure 2/2E/Figure2E_GM130_LC3.tif]

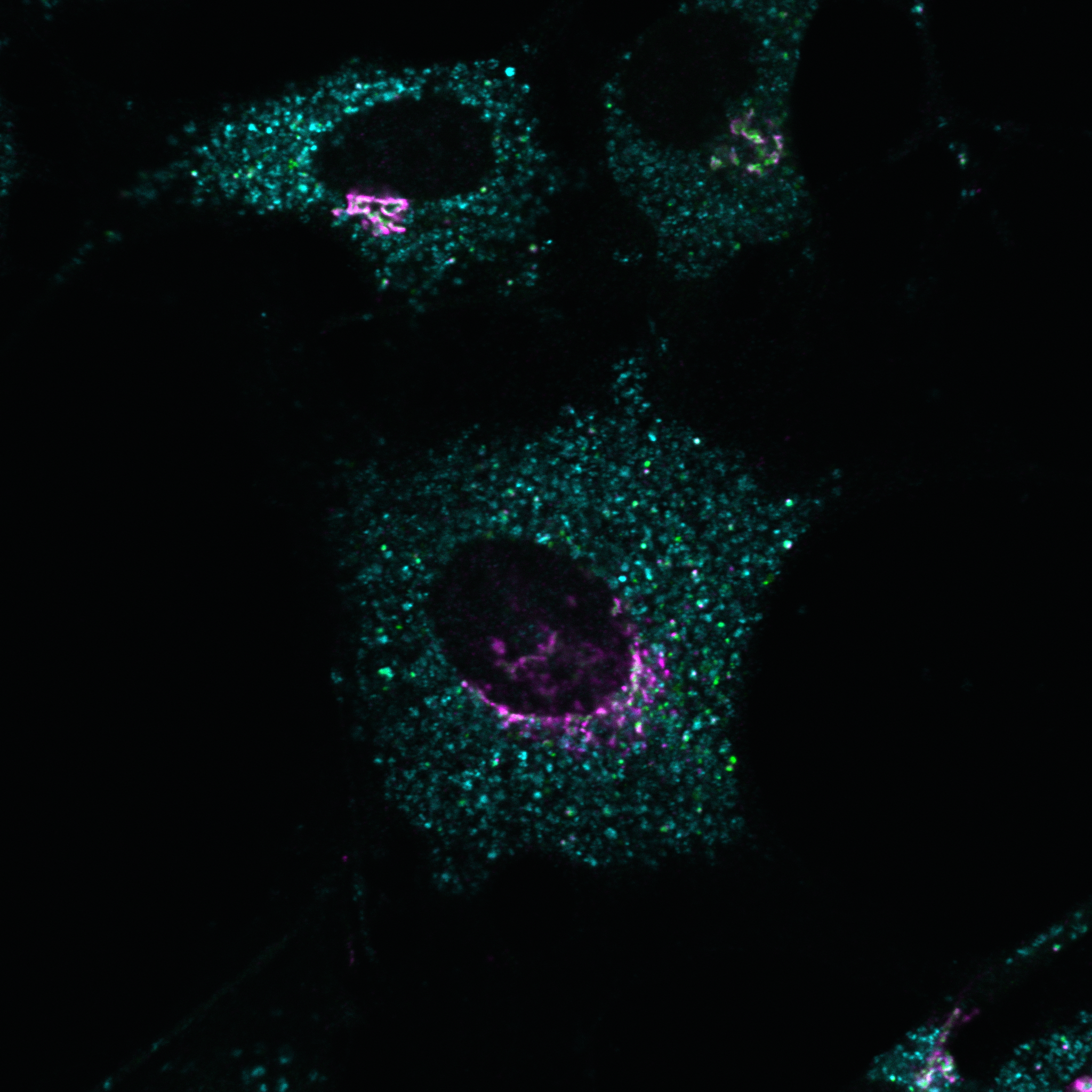

Supplement: Supplementary file 5 — Source data Fig. 2 [file 44318_2024_131_MOESM5_ESM.zip › Figure 2/2E/Figure2E_GM130_merge.tif]

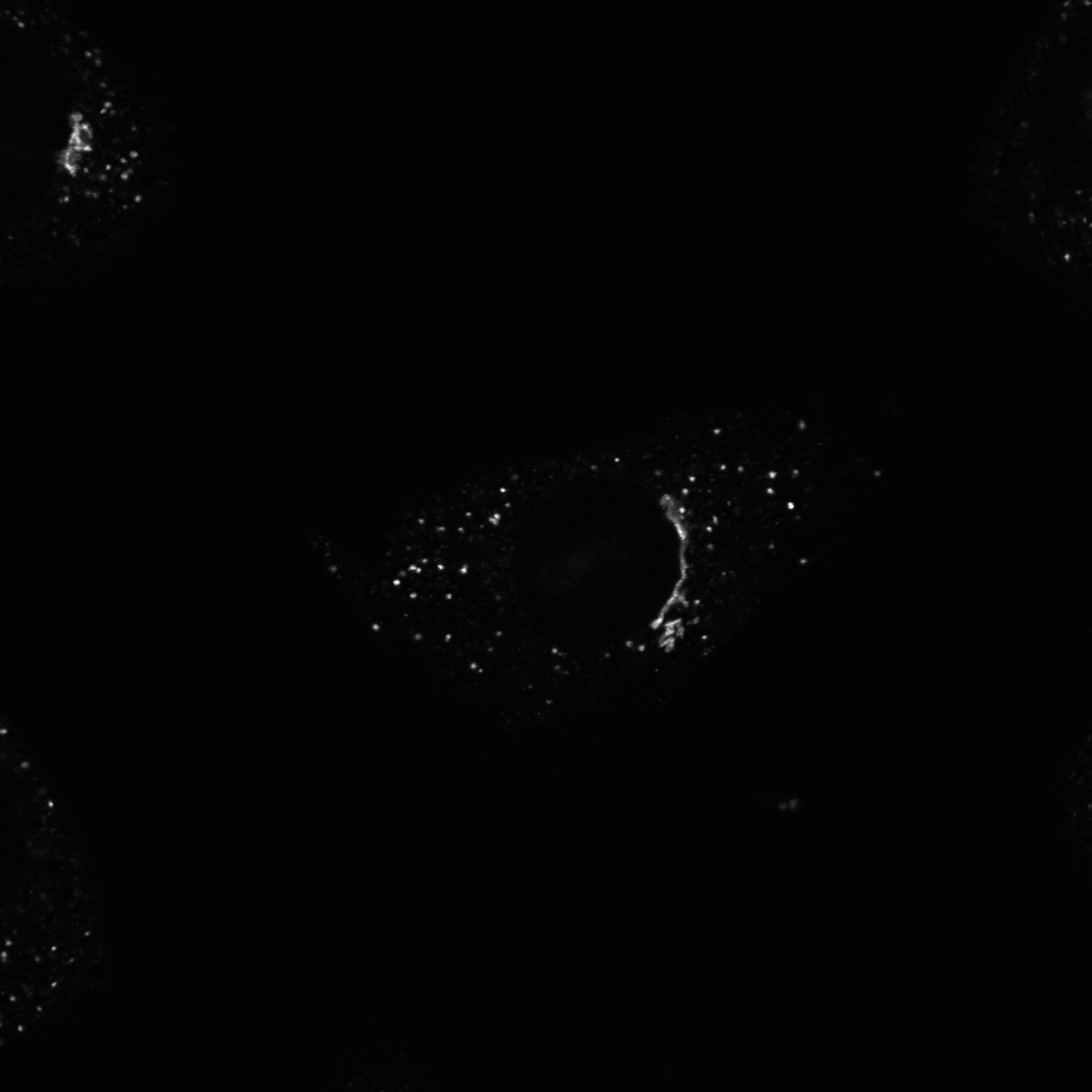

Supplement: Supplementary file 5 — Source data Fig. 2 [file 44318_2024_131_MOESM5_ESM.zip › Figure 2/2E/Figure2E_MAN2A1-mCherry_EGFP.tif]

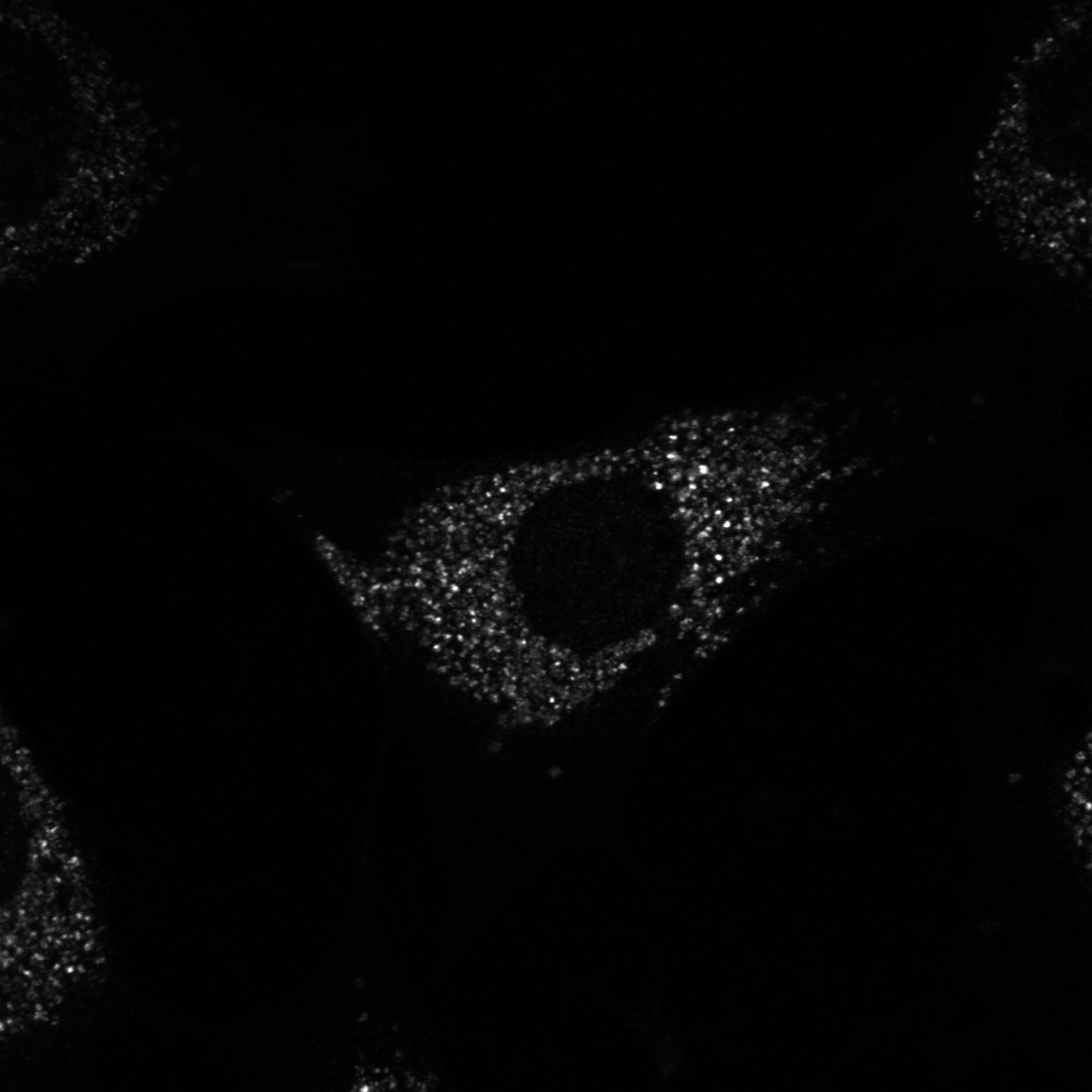

Supplement: Supplementary file 5 — Source data Fig. 2 [file 44318_2024_131_MOESM5_ESM.zip › Figure 2/2E/Figure2E_MAN2A1-mCherry_LC3.tif]

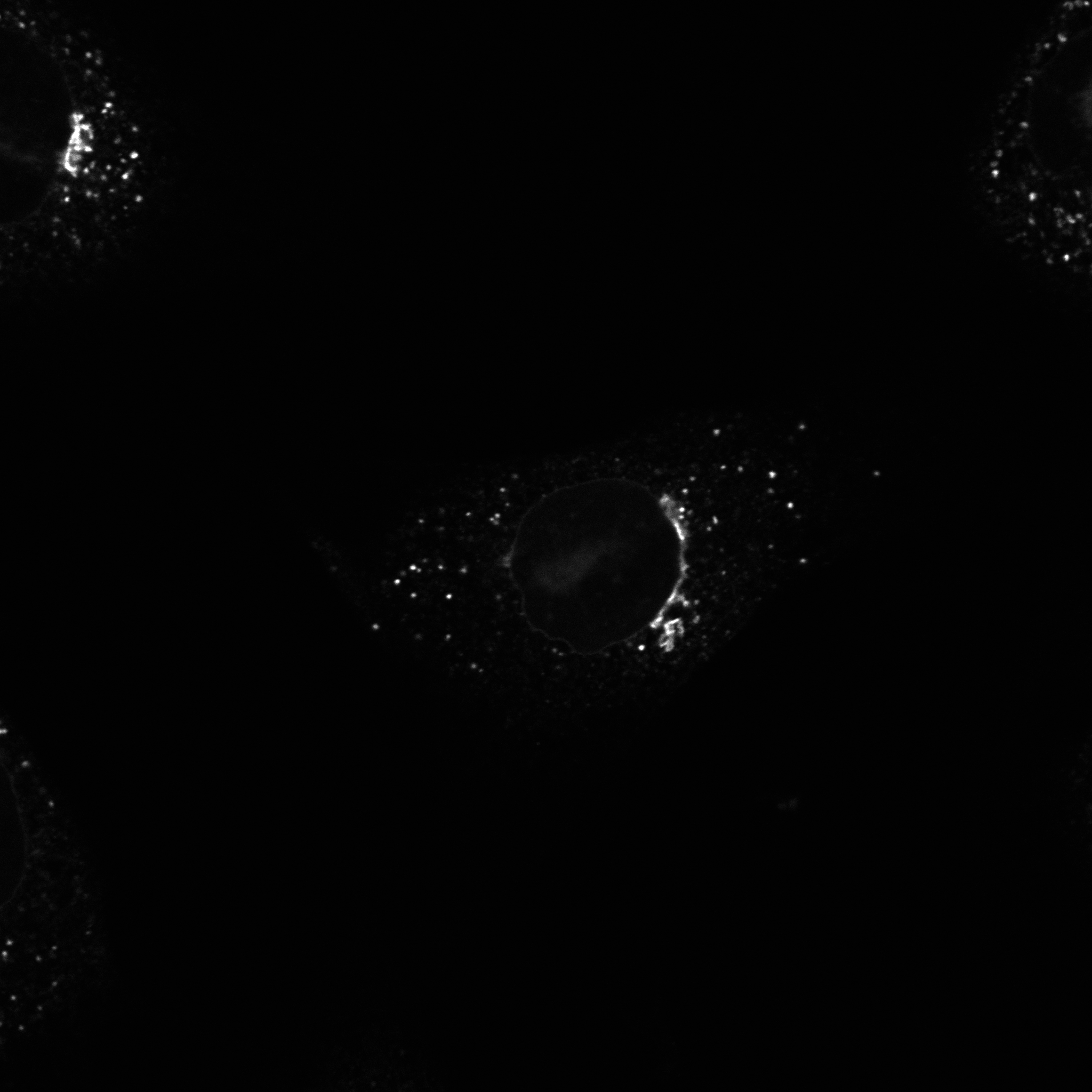

Supplement: Supplementary file 5 — Source data Fig. 2 [file 44318_2024_131_MOESM5_ESM.zip › Figure 2/2E/Figure2E_MAN2A1-mCherry_mCherry.tif]

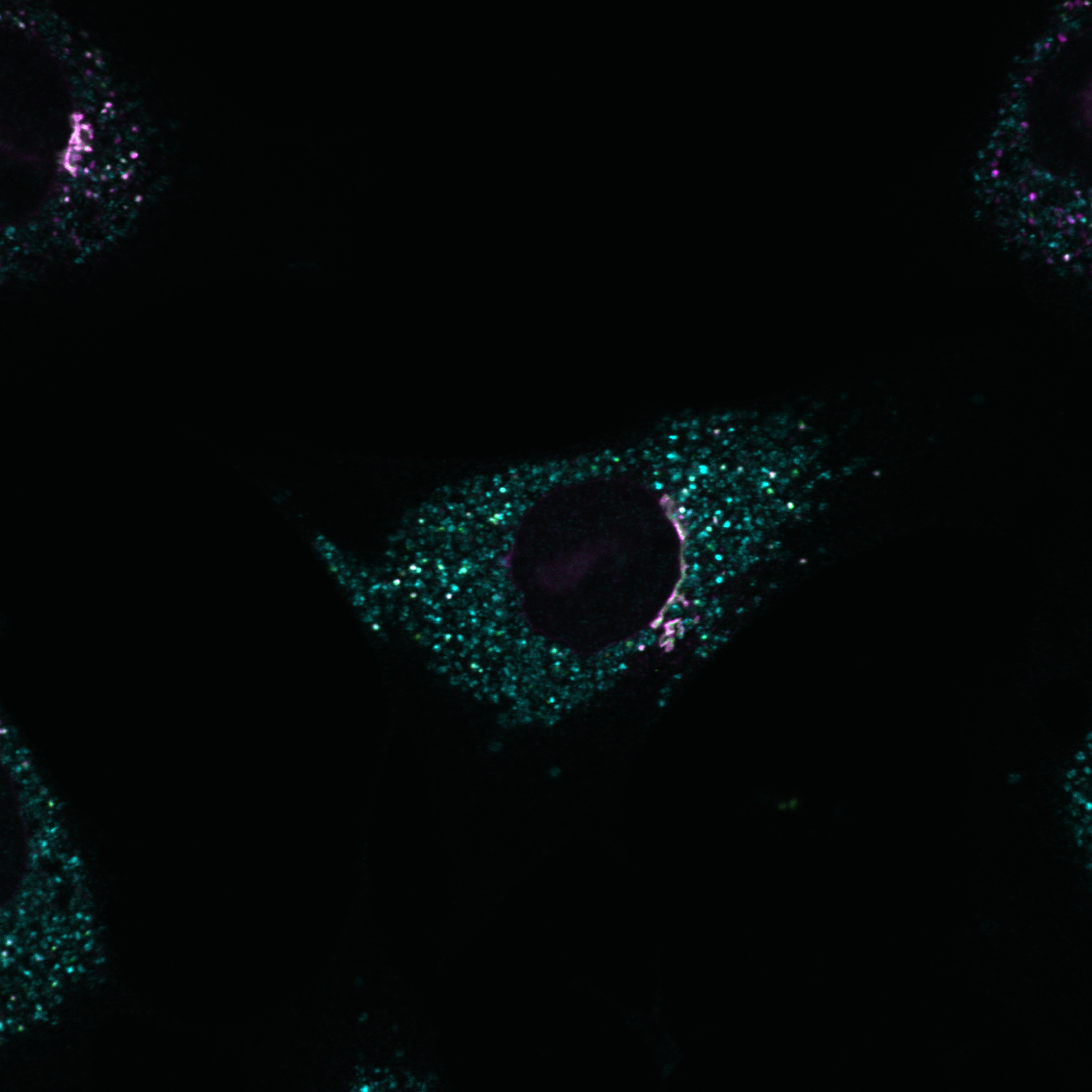

Supplement: Supplementary file 5 — Source data Fig. 2 [file 44318_2024_131_MOESM5_ESM.zip › Figure 2/2E/Figure2E_MAN2A1-mCherry_merge.tif]

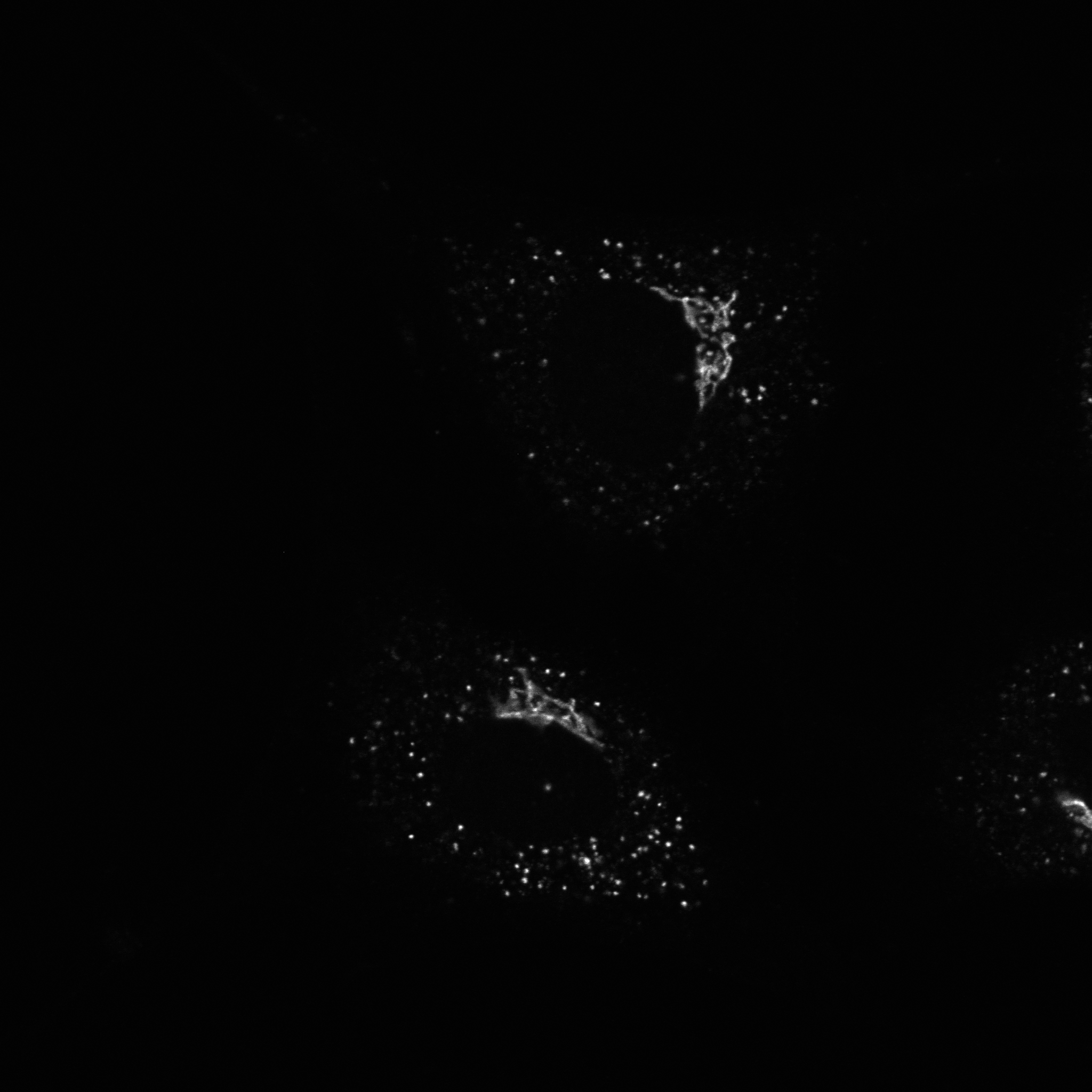

Supplement: Supplementary file 5 — Source data Fig. 2 [file 44318_2024_131_MOESM5_ESM.zip › Figure 2/2E/Figure2E_TMEM165_EGFP.tif]

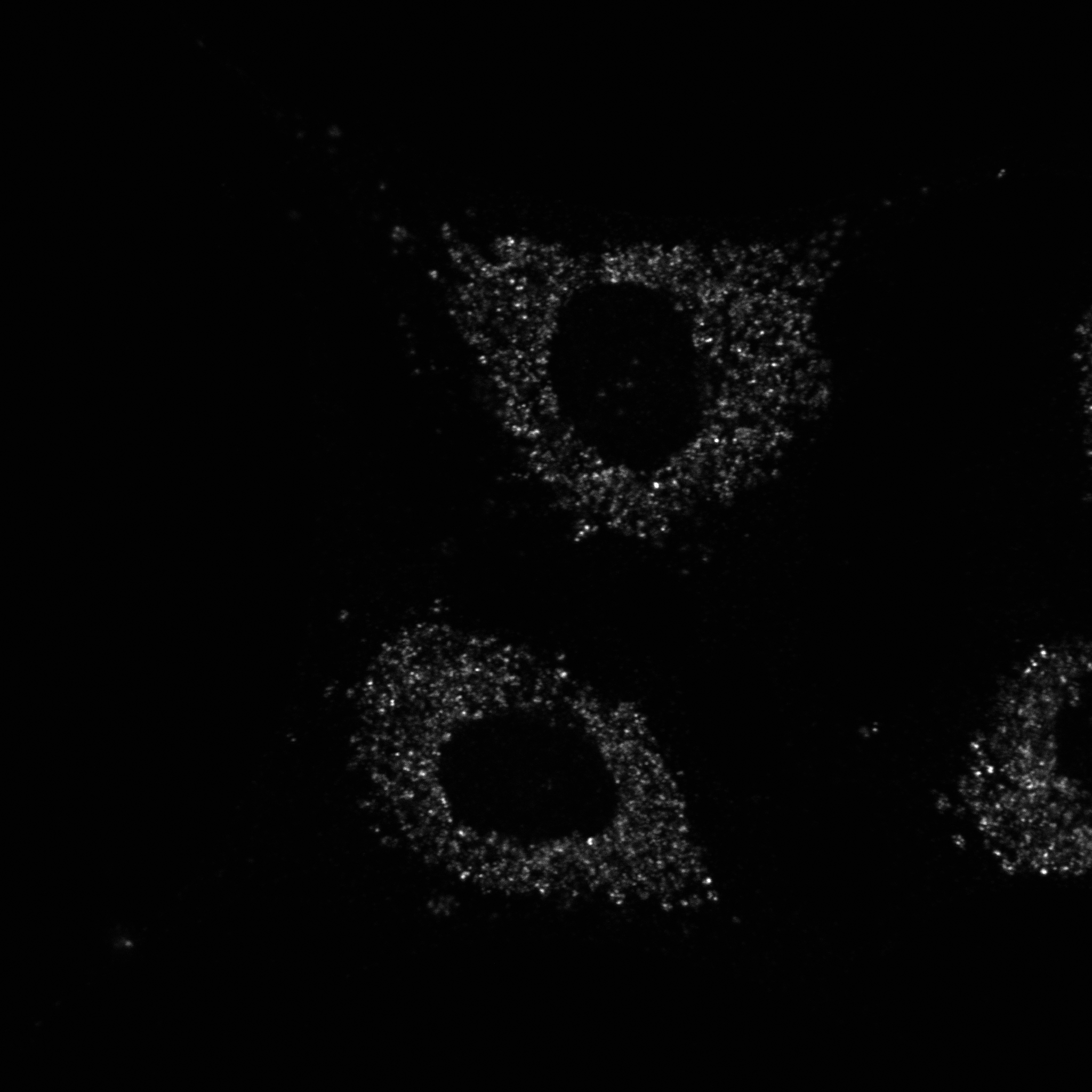

Supplement: Supplementary file 5 — Source data Fig. 2 [file 44318_2024_131_MOESM5_ESM.zip › Figure 2/2E/Figure2E_TMEM165_LC3.tif]

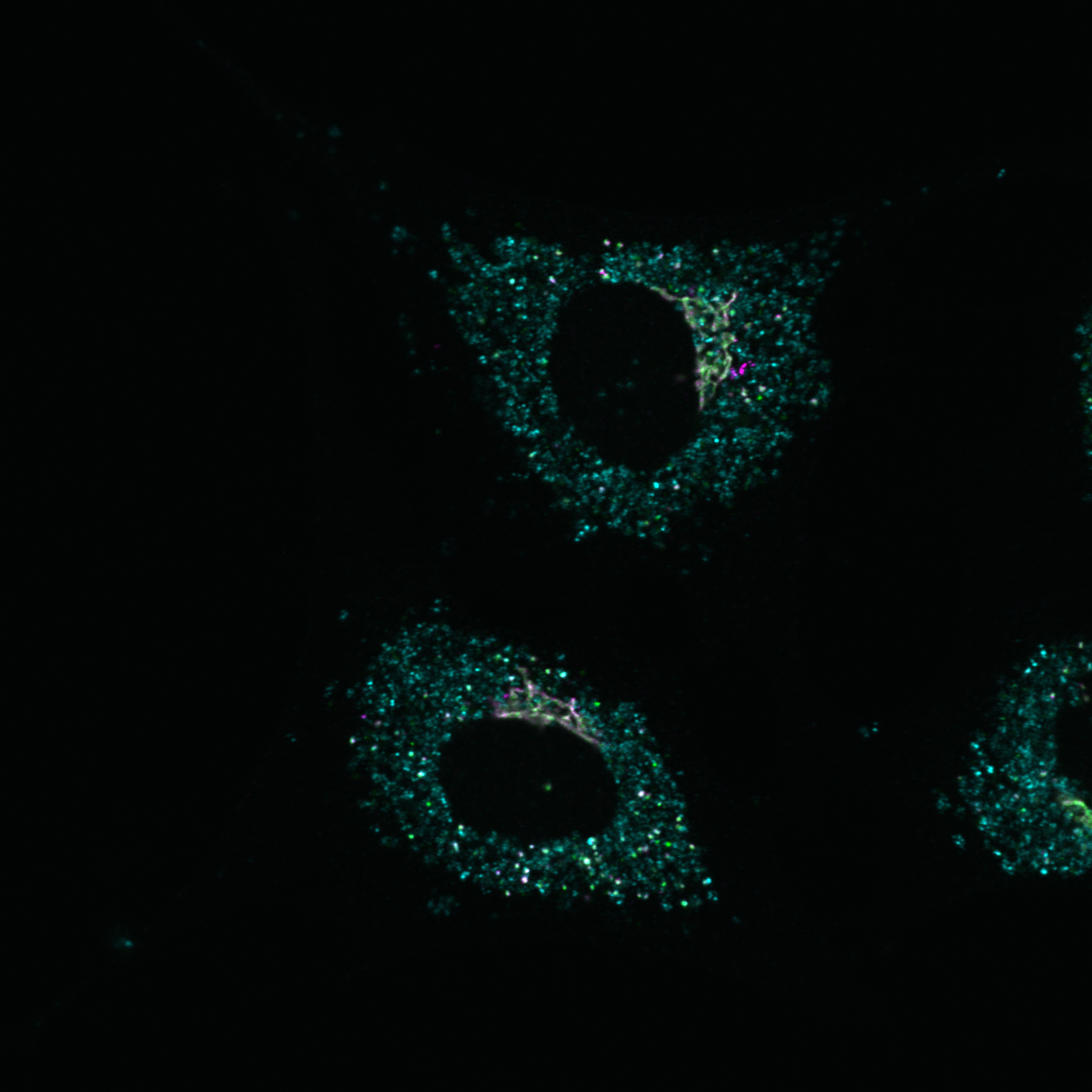

Supplement: Supplementary file 5 — Source data Fig. 2 [file 44318_2024_131_MOESM5_ESM.zip › Figure 2/2E/Figure2E_TMEM165_merge.tif]

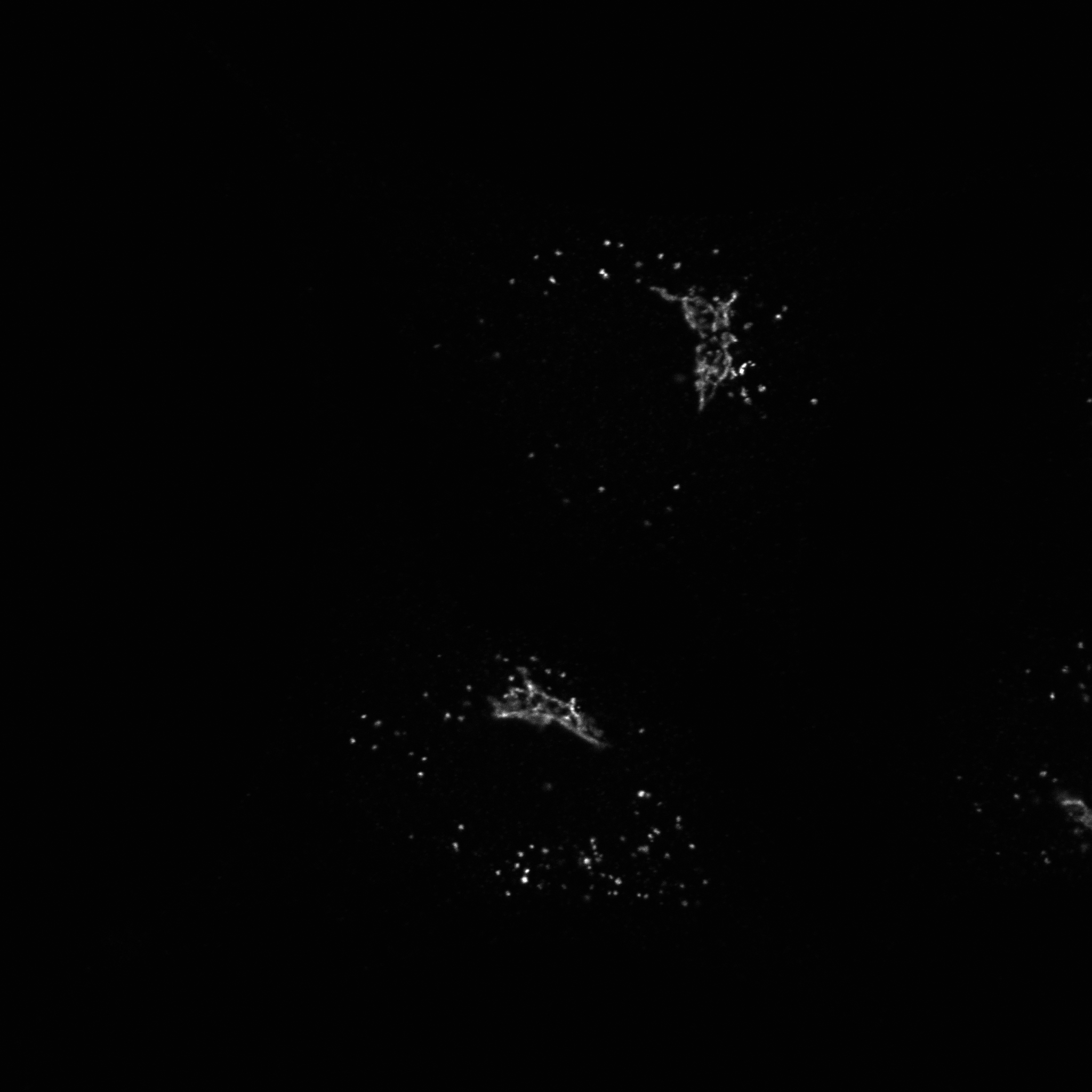

Supplement: Supplementary file 5 — Source data Fig. 2 [file 44318_2024_131_MOESM5_ESM.zip › Figure 2/2E/Figure2E_TMEM165_TMEM165.tif]

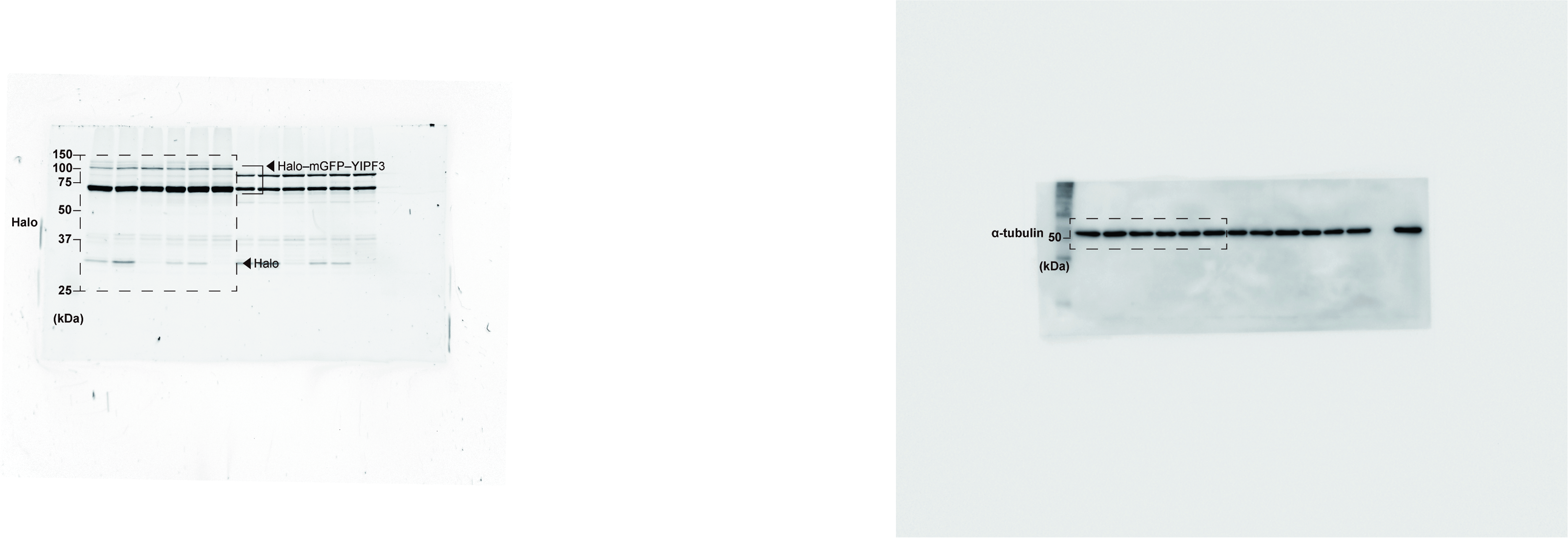

Supplement: Supplementary file 6 — Source data Fig. 3 [file 44318_2024_131_MOESM6_ESM.zip › Figure 3/3D/western_Figure3D.tif]

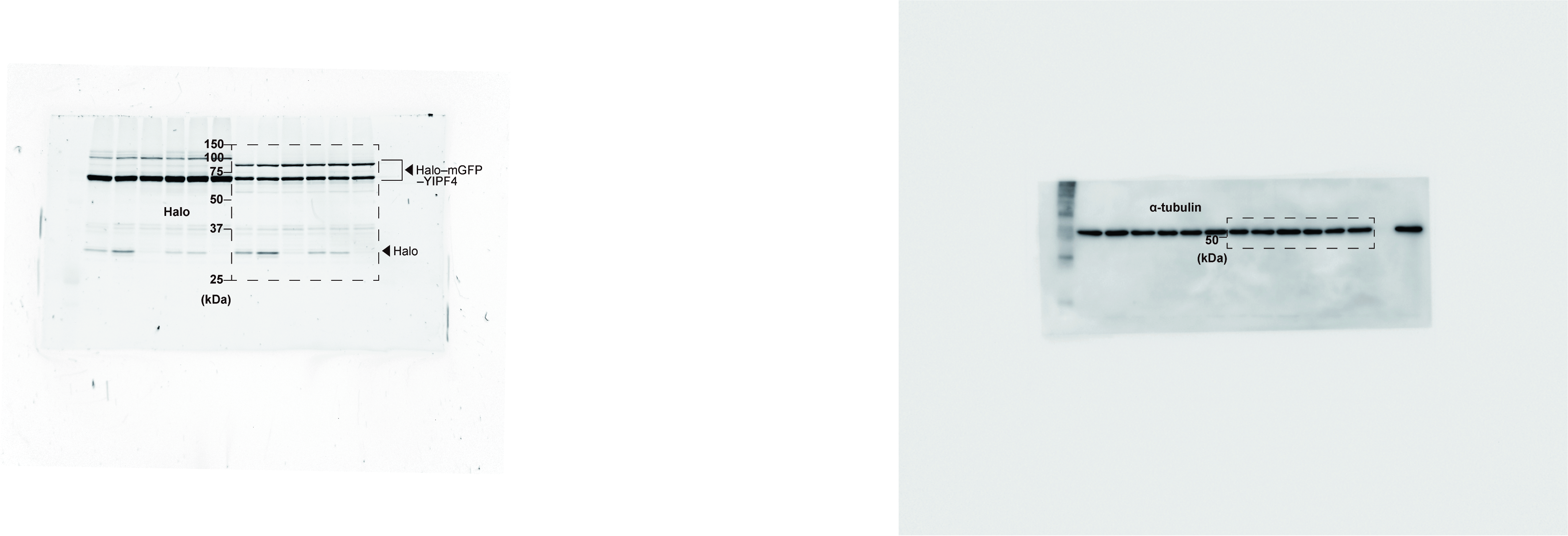

Supplement: Supplementary file 6 — Source data Fig. 3 [file 44318_2024_131_MOESM6_ESM.zip › Figure 3/3F/western_Figure3F.tif]

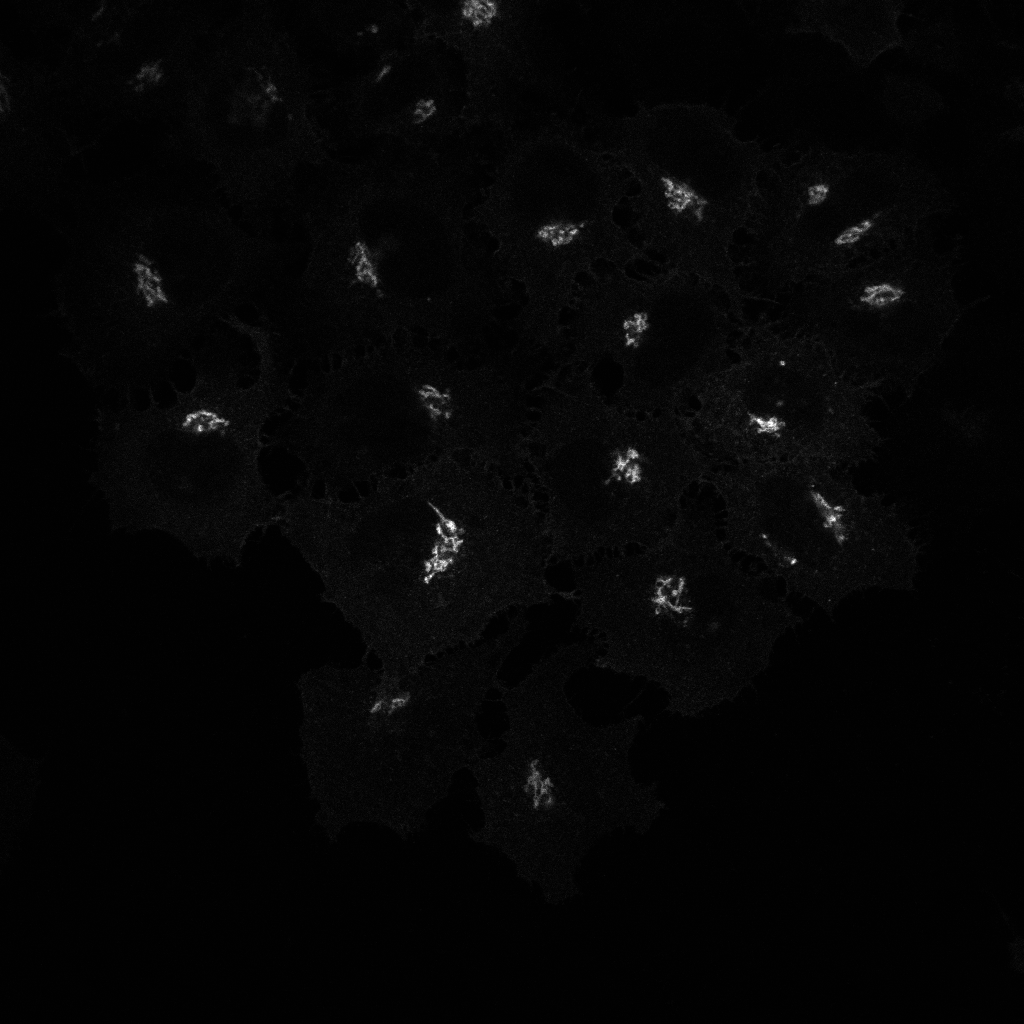

Supplement: Supplementary file 8 — Source data Fig. 5 [file 44318_2024_131_MOESM8_ESM.zip › Figure 5/5B/Figure5B_GM130_EGFP.tif]

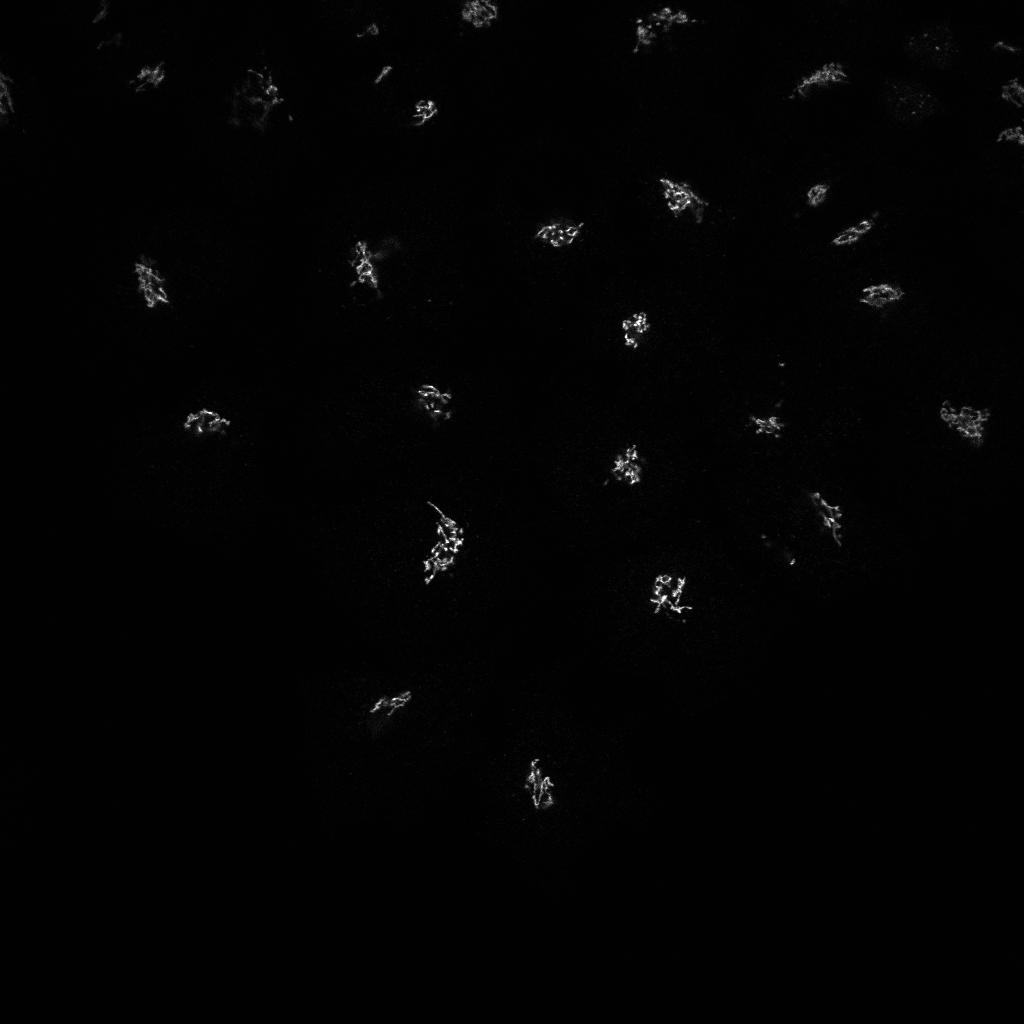

Supplement: Supplementary file 8 — Source data Fig. 5 [file 44318_2024_131_MOESM8_ESM.zip › Figure 5/5B/Figure5B_GM130_GM130.tif]

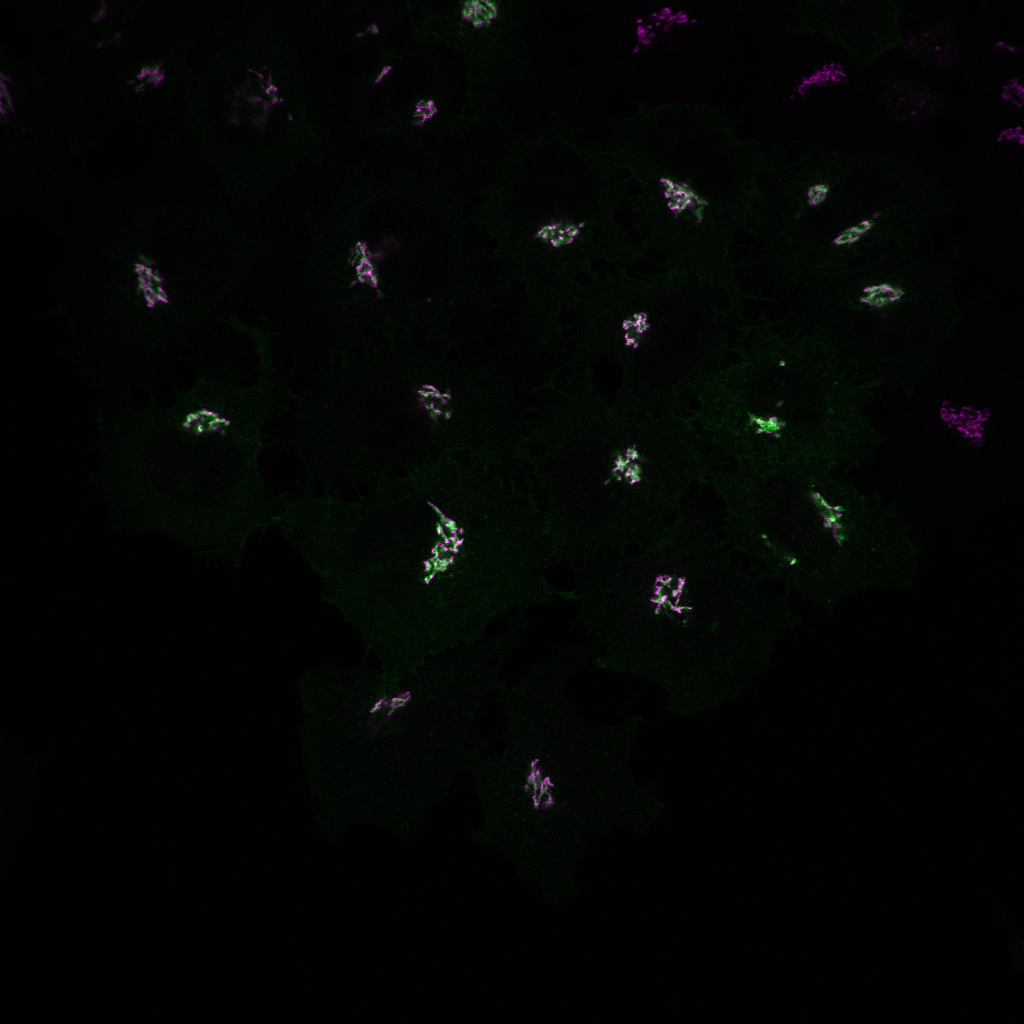

Supplement: Supplementary file 8 — Source data Fig. 5 [file 44318_2024_131_MOESM8_ESM.zip › Figure 5/5B/Figure5B_GM130_merge.tif]

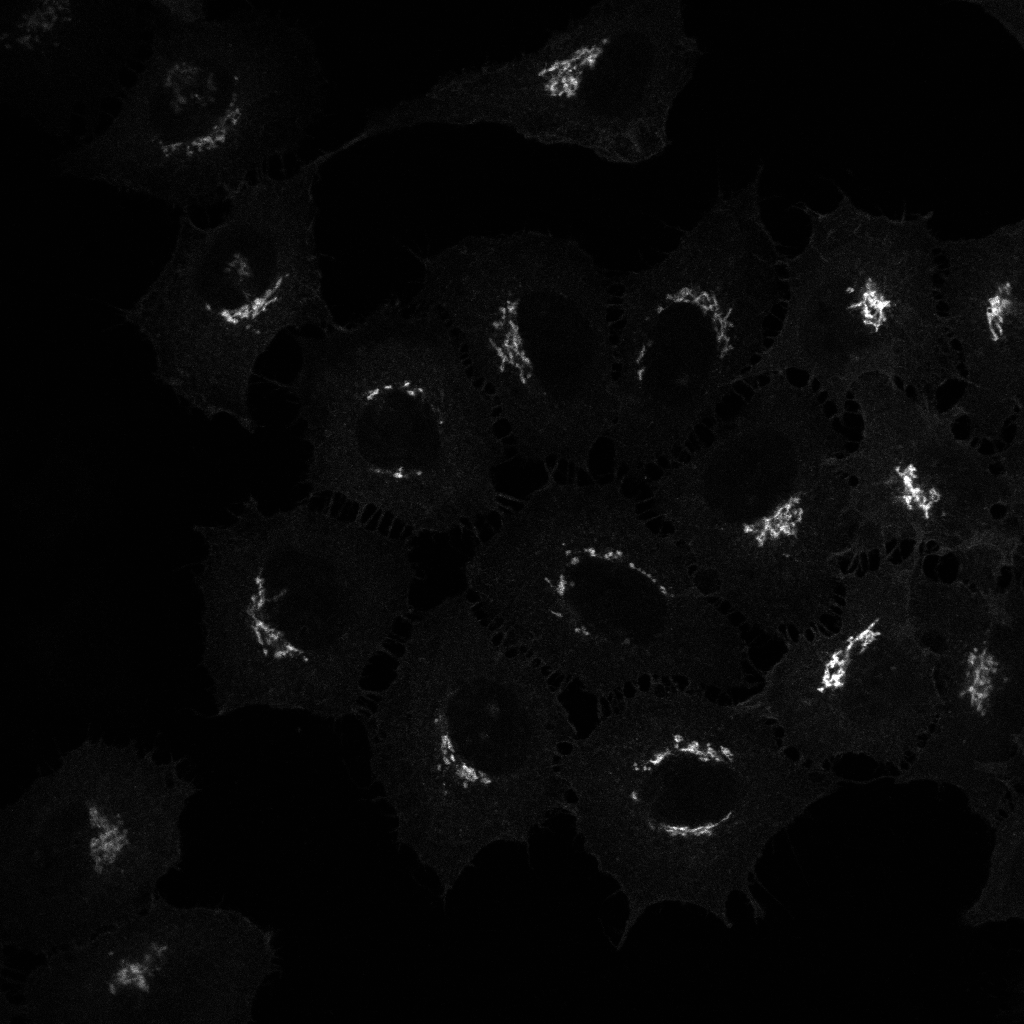

Supplement: Supplementary file 8 — Source data Fig. 5 [file 44318_2024_131_MOESM8_ESM.zip › Figure 5/5B/Figure5B_p230_EGFP.tif]

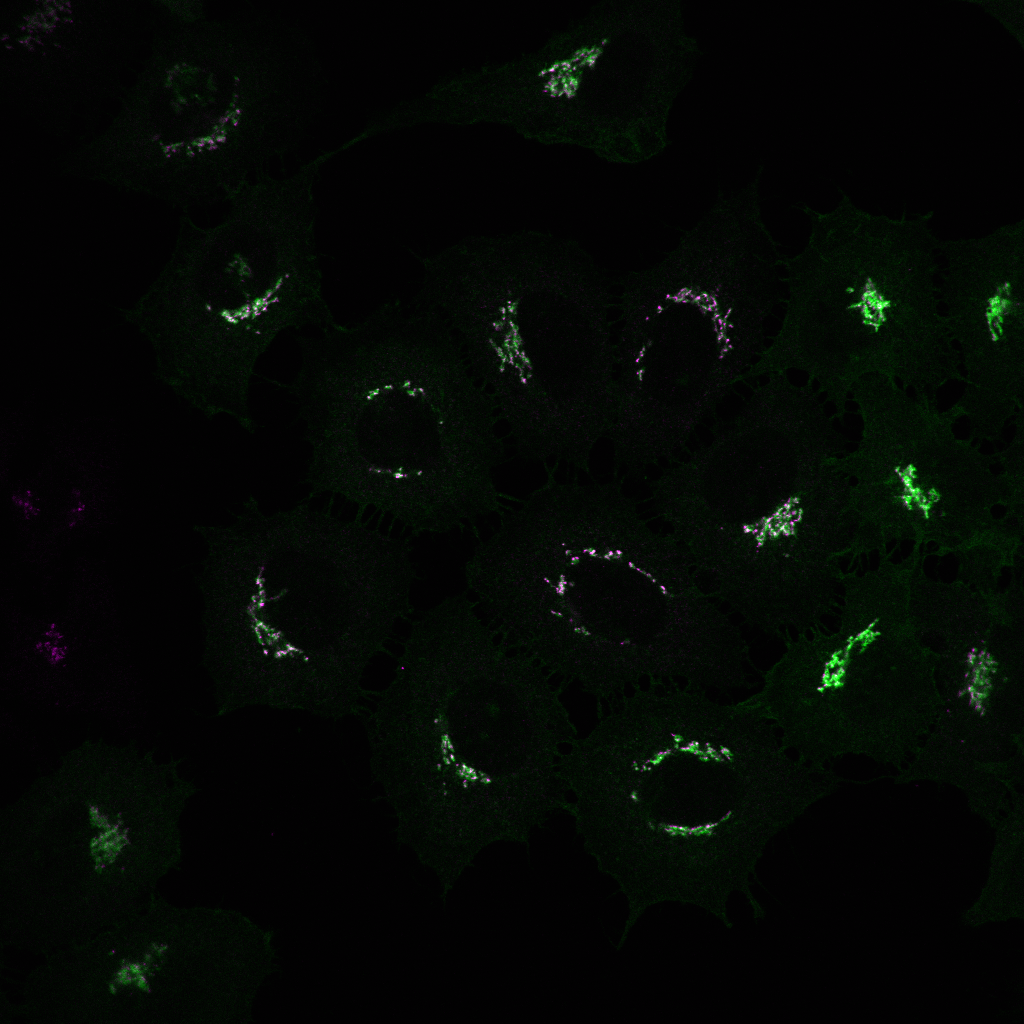

Supplement: Supplementary file 8 — Source data Fig. 5 [file 44318_2024_131_MOESM8_ESM.zip › Figure 5/5B/Figure5B_p230_merge.tif]

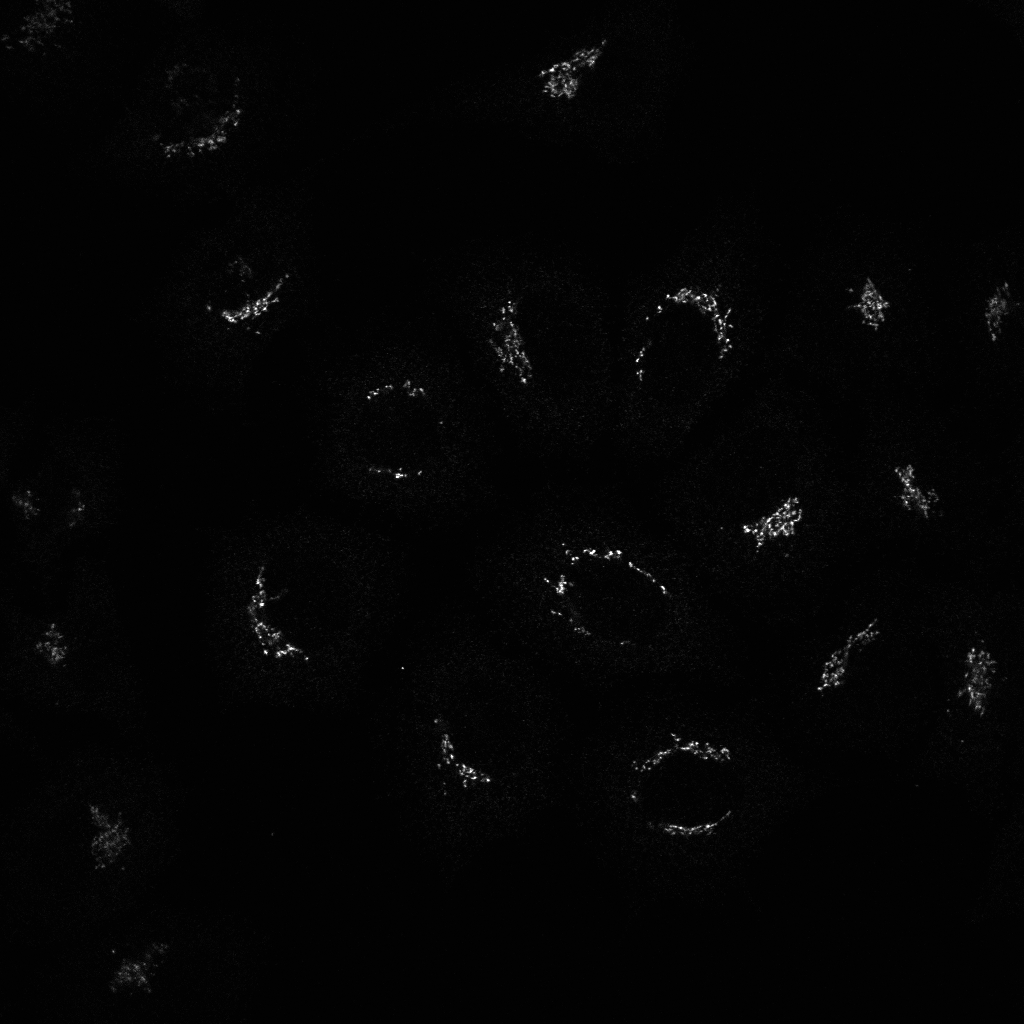

Supplement: Supplementary file 8 — Source data Fig. 5 [file 44318_2024_131_MOESM8_ESM.zip › Figure 5/5B/Figure5B_p230_p230tif.tif]

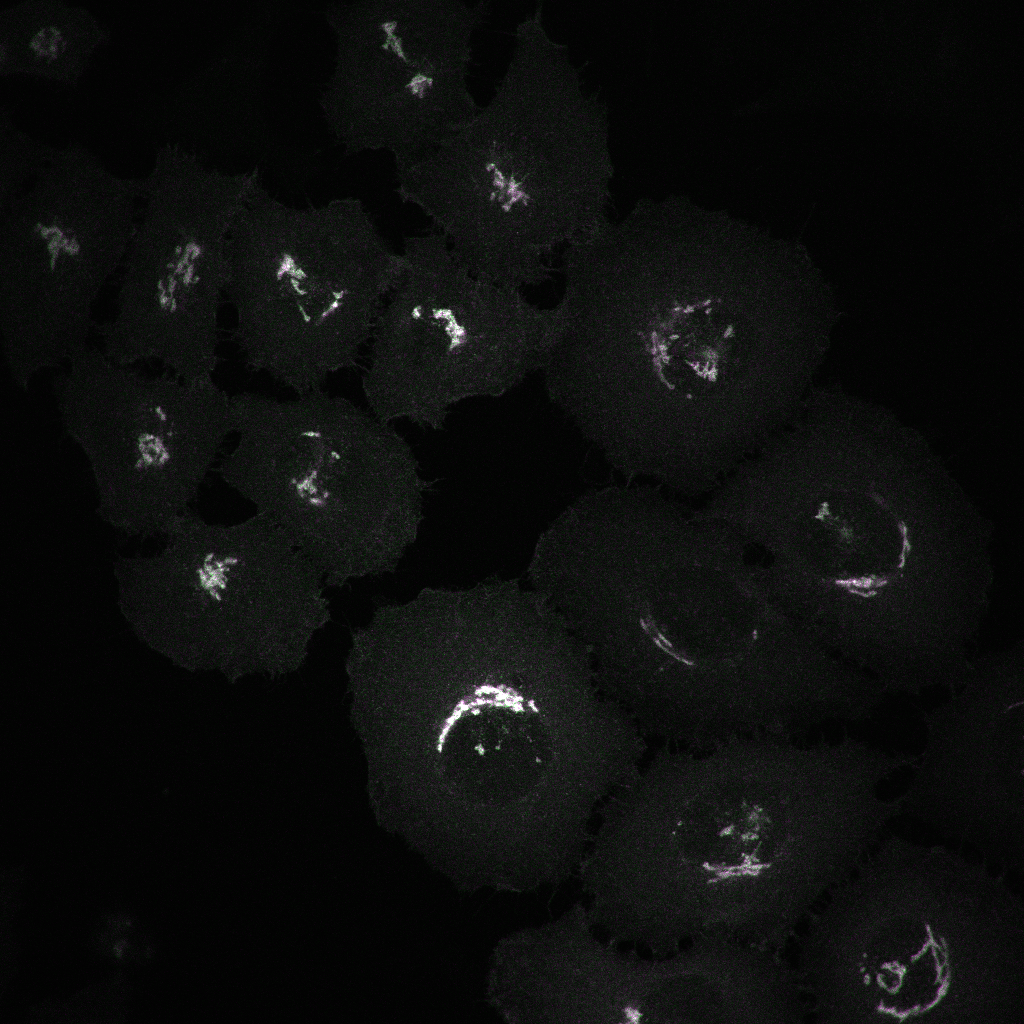

Supplement: Supplementary file 8 — Source data Fig. 5 [file 44318_2024_131_MOESM8_ESM.zip › Figure 5/5C/Figure5C_Growing_FIP200KO.tif]

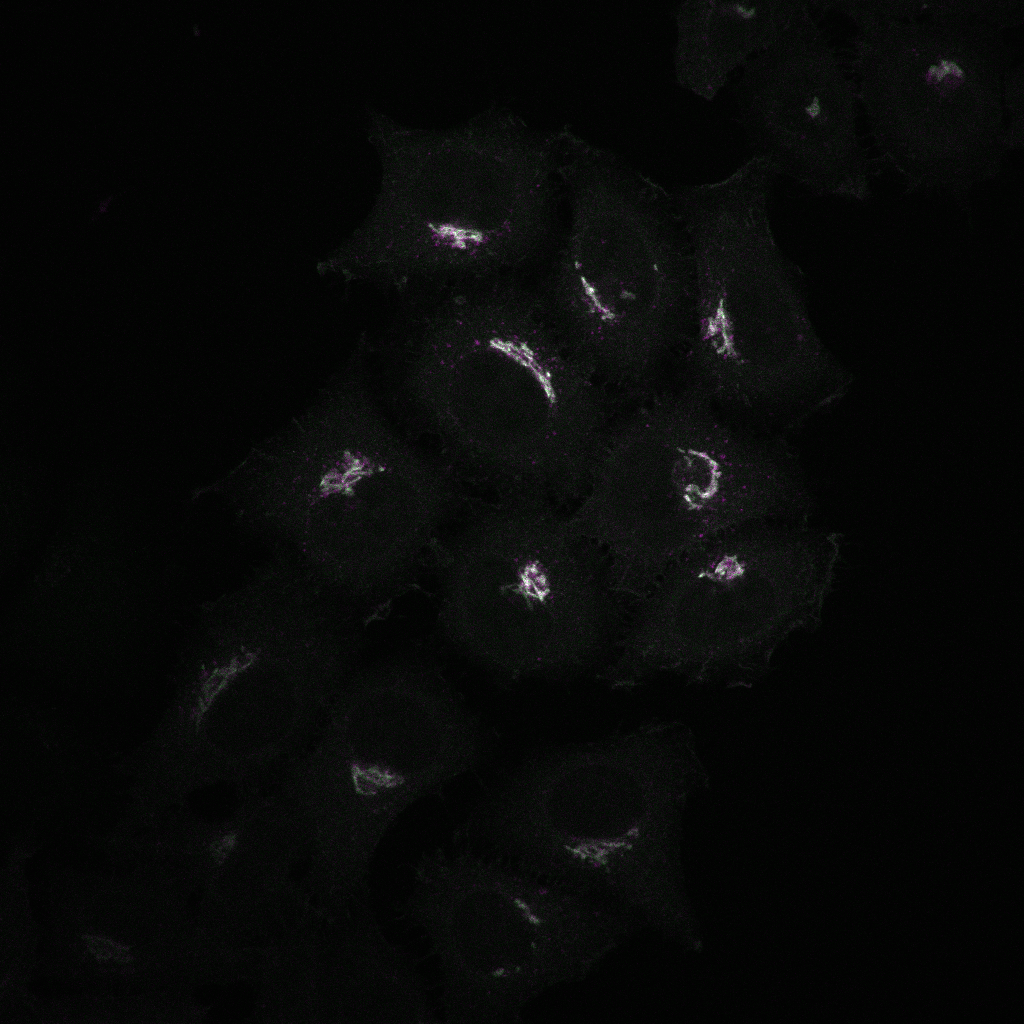

Supplement: Supplementary file 8 — Source data Fig. 5 [file 44318_2024_131_MOESM8_ESM.zip › Figure 5/5C/Figure5C_Growing_WT.tif]

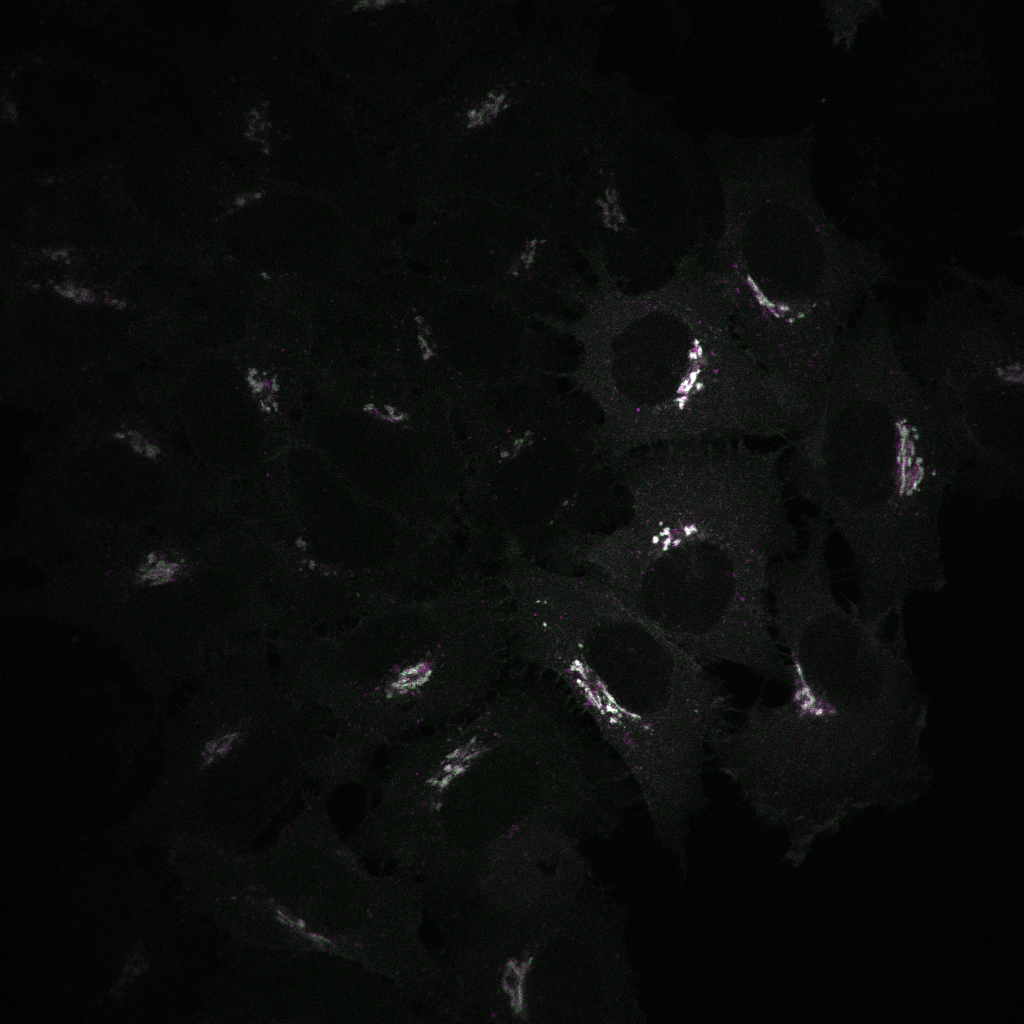

Supplement: Supplementary file 8 — Source data Fig. 5 [file 44318_2024_131_MOESM8_ESM.zip › Figure 5/5C/Figure5C_Growing_YIPF3KO.tif]

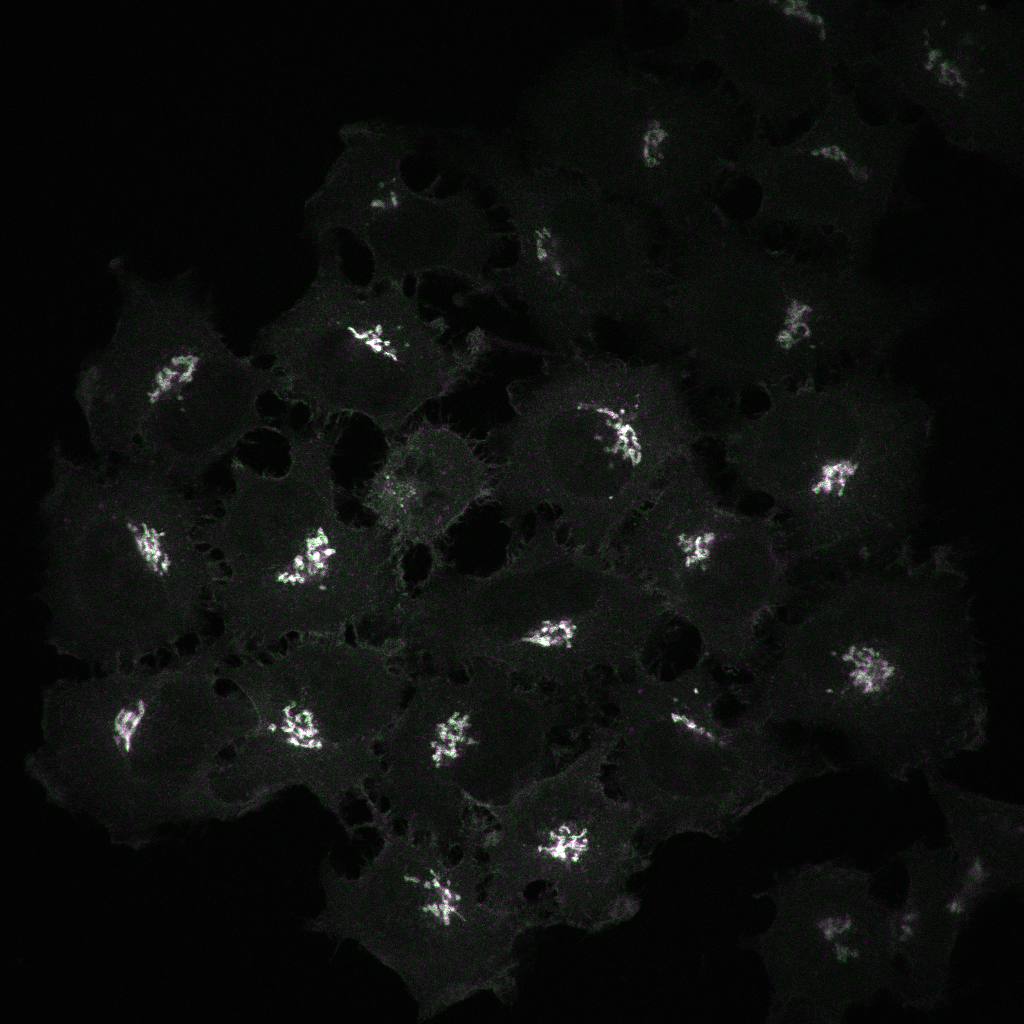

Supplement: Supplementary file 8 — Source data Fig. 5 [file 44318_2024_131_MOESM8_ESM.zip › Figure 5/5C/Figure5C_Growing_YIPF4KO.tif]

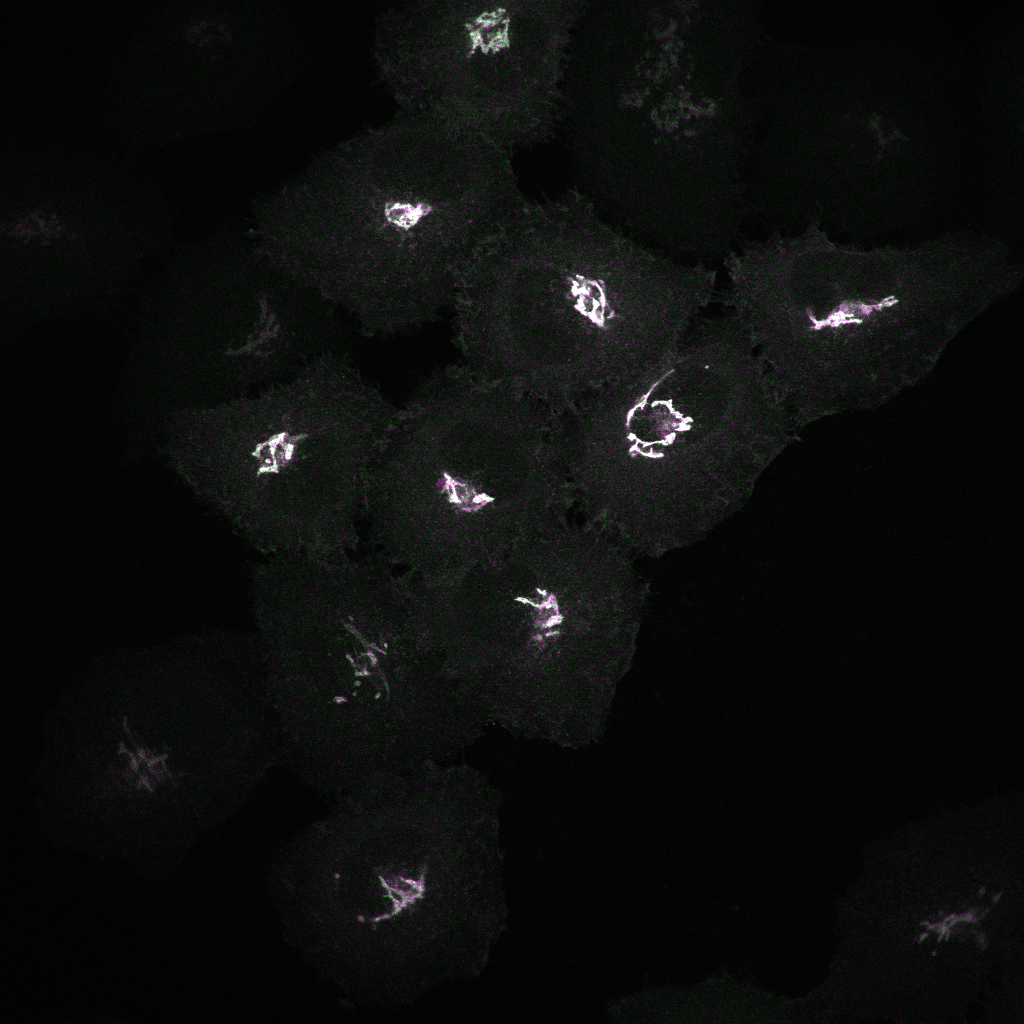

Supplement: Supplementary file 8 — Source data Fig. 5 [file 44318_2024_131_MOESM8_ESM.zip › Figure 5/5C/Figure5C_Starvation_FIP200KO.tif]

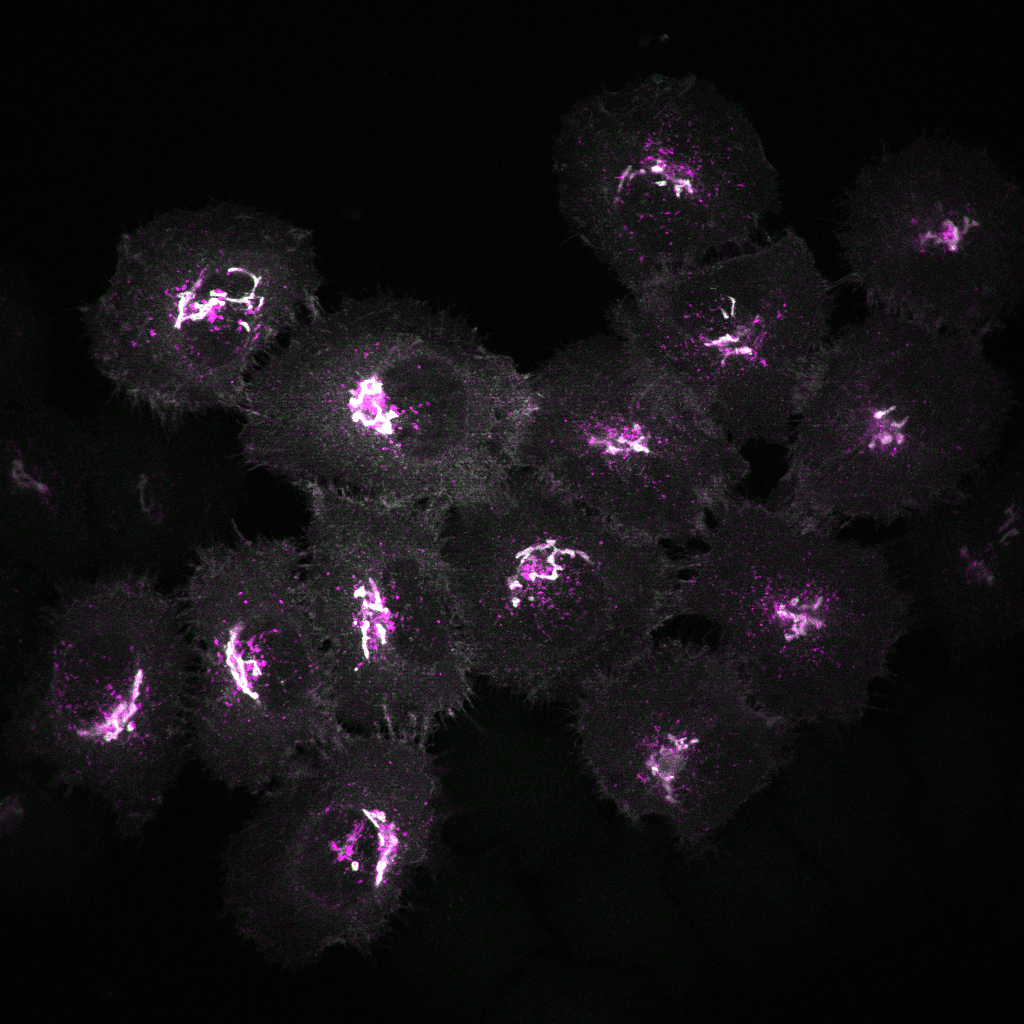

Supplement: Supplementary file 8 — Source data Fig. 5 [file 44318_2024_131_MOESM8_ESM.zip › Figure 5/5C/Figure5C_Starvation_WT.tif]

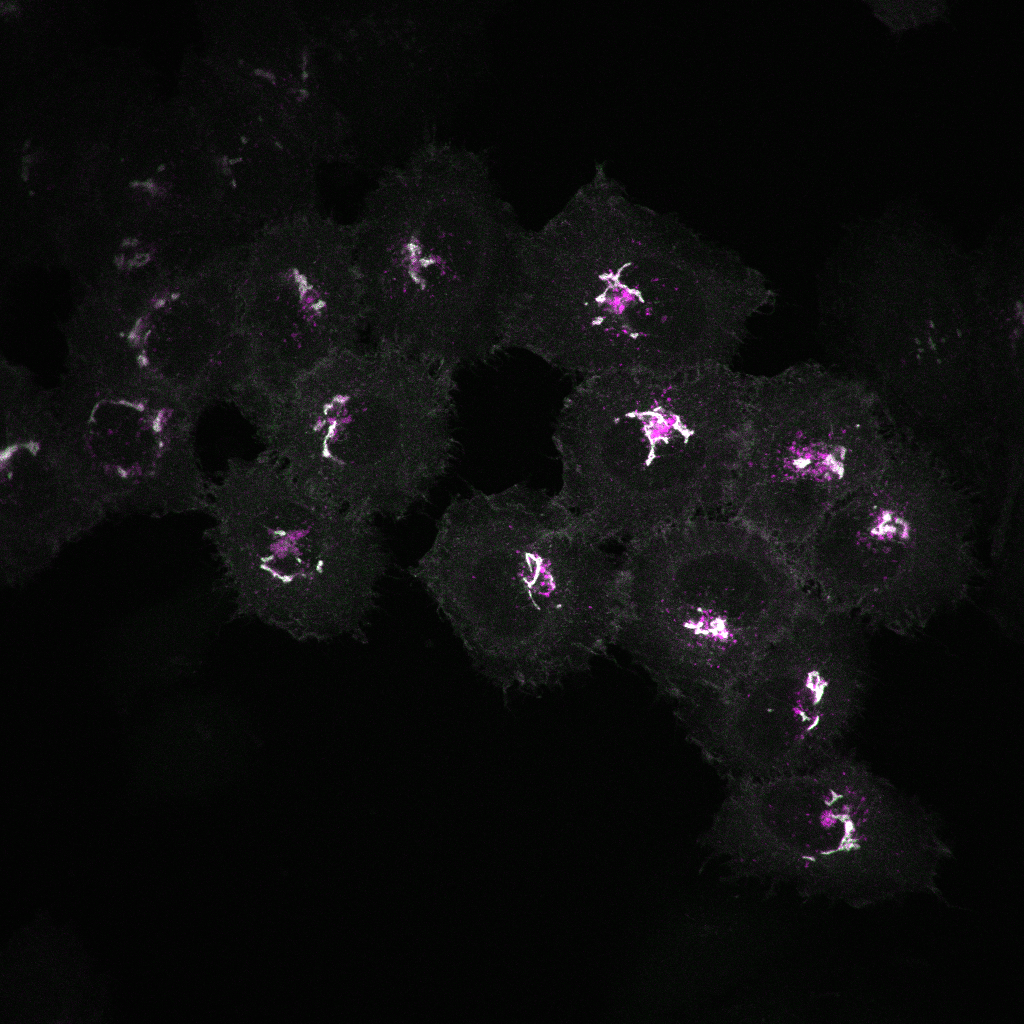

Supplement: Supplementary file 8 — Source data Fig. 5 [file 44318_2024_131_MOESM8_ESM.zip › Figure 5/5C/Figure5C_Starvation_YIPF3KO.tif]

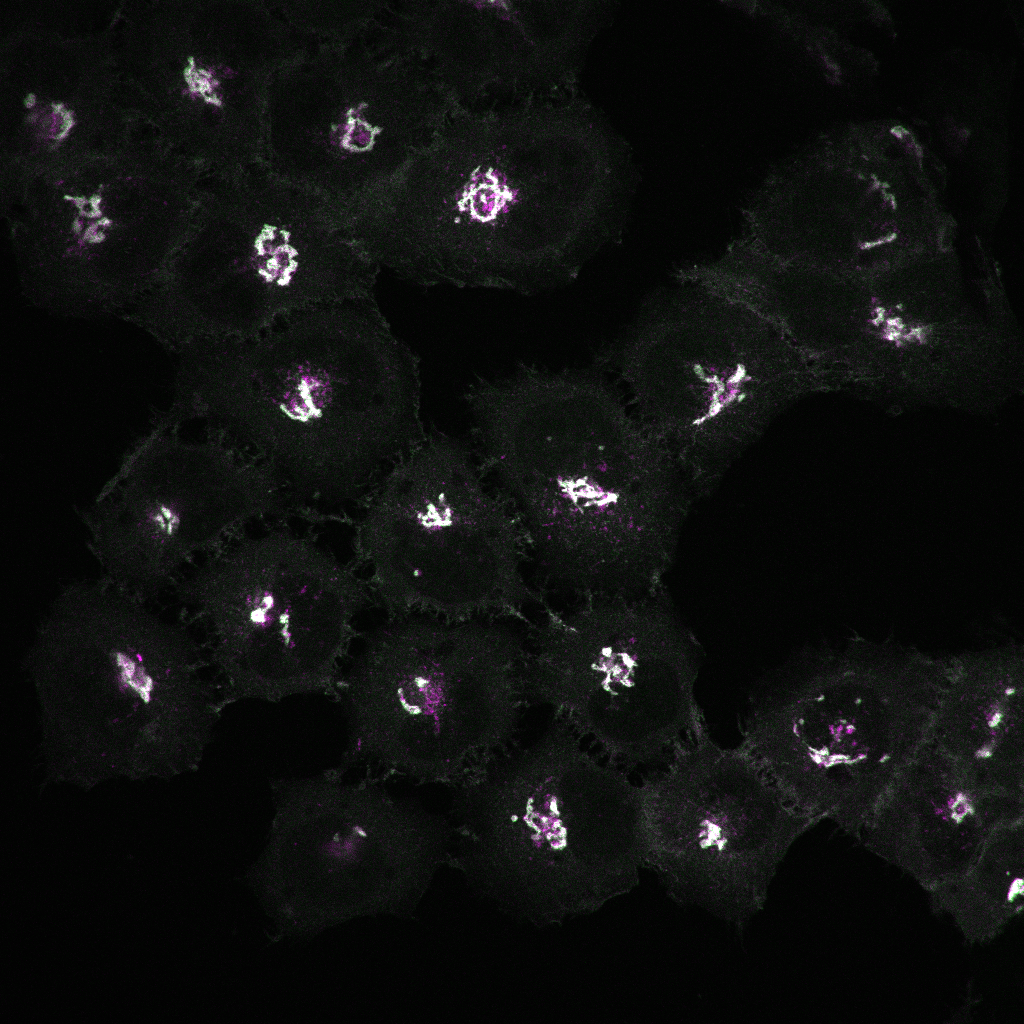

Supplement: Supplementary file 8 — Source data Fig. 5 [file 44318_2024_131_MOESM8_ESM.zip › Figure 5/5C/Figure5C_Starvation_YIPF4KO.tif]

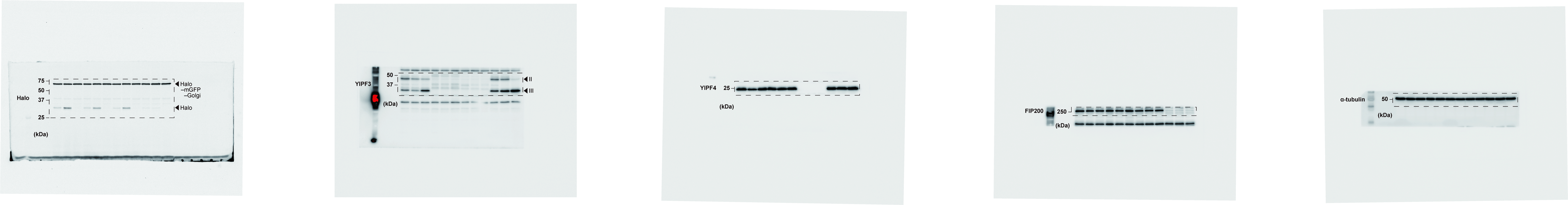

Supplement: Supplementary file 9 — Source data Fig. 6 [file 44318_2024_131_MOESM9_ESM.zip › Figure 6/6B/western_Figure6B.tif]

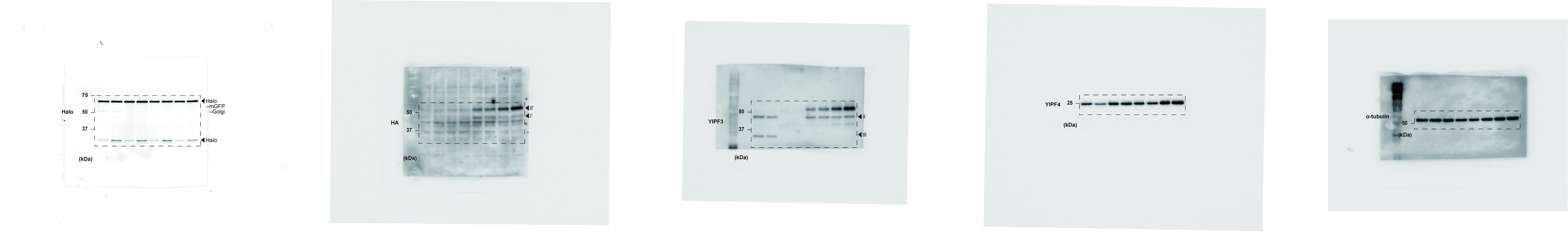

Supplement: Supplementary file 12 — Source data Fig. 9 [file 44318_2024_131_MOESM12_ESM.zip › Figure 9/9E/western_Figure9E.tif]

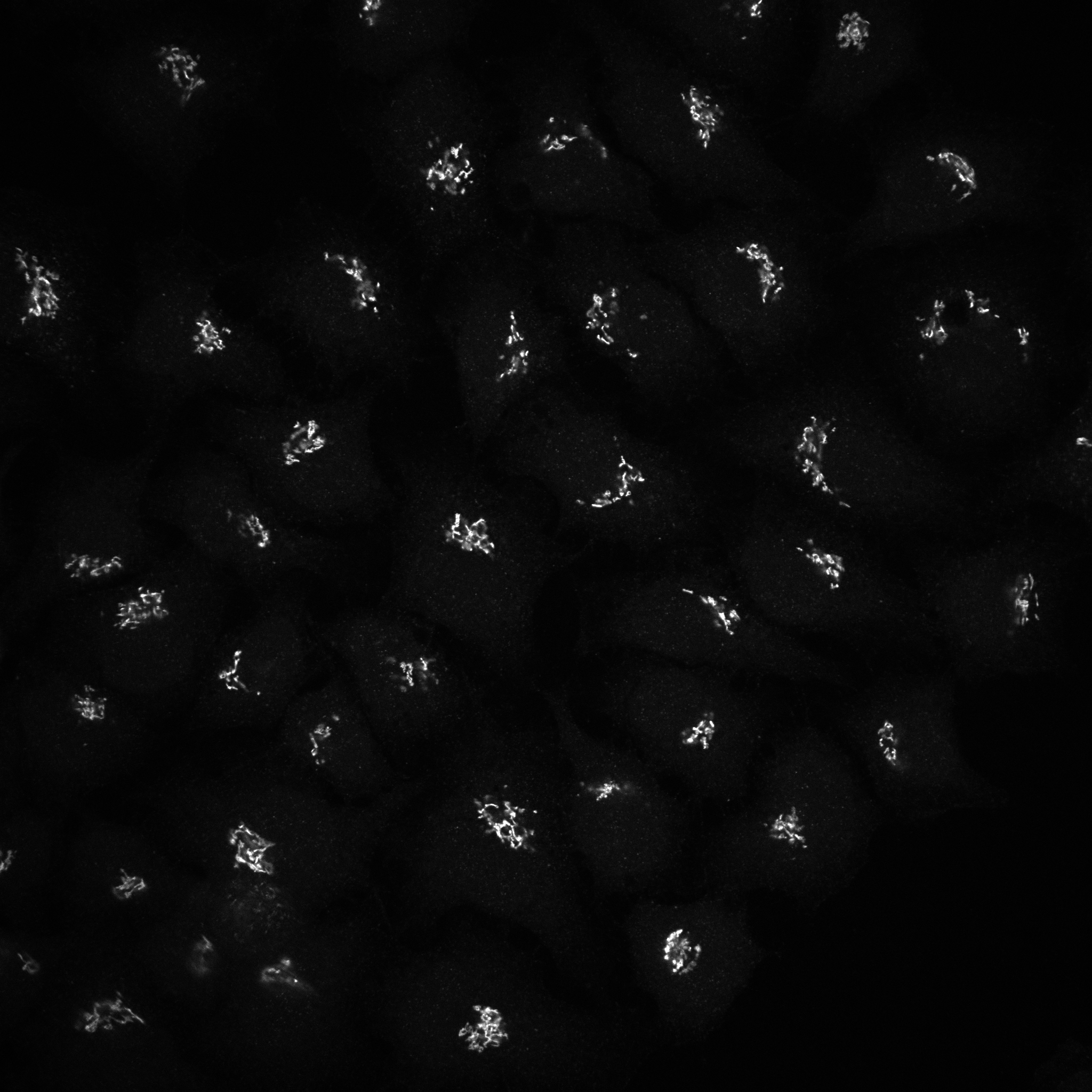

Supplement: Supplementary file 13 — EV and Appendix Figure Source Data [file 44318_2024_131_MOESM13_ESM.zip › ExpandedFigure 1/EV1B/EV1B_GM130_Growing_YIPF3KO.tif]

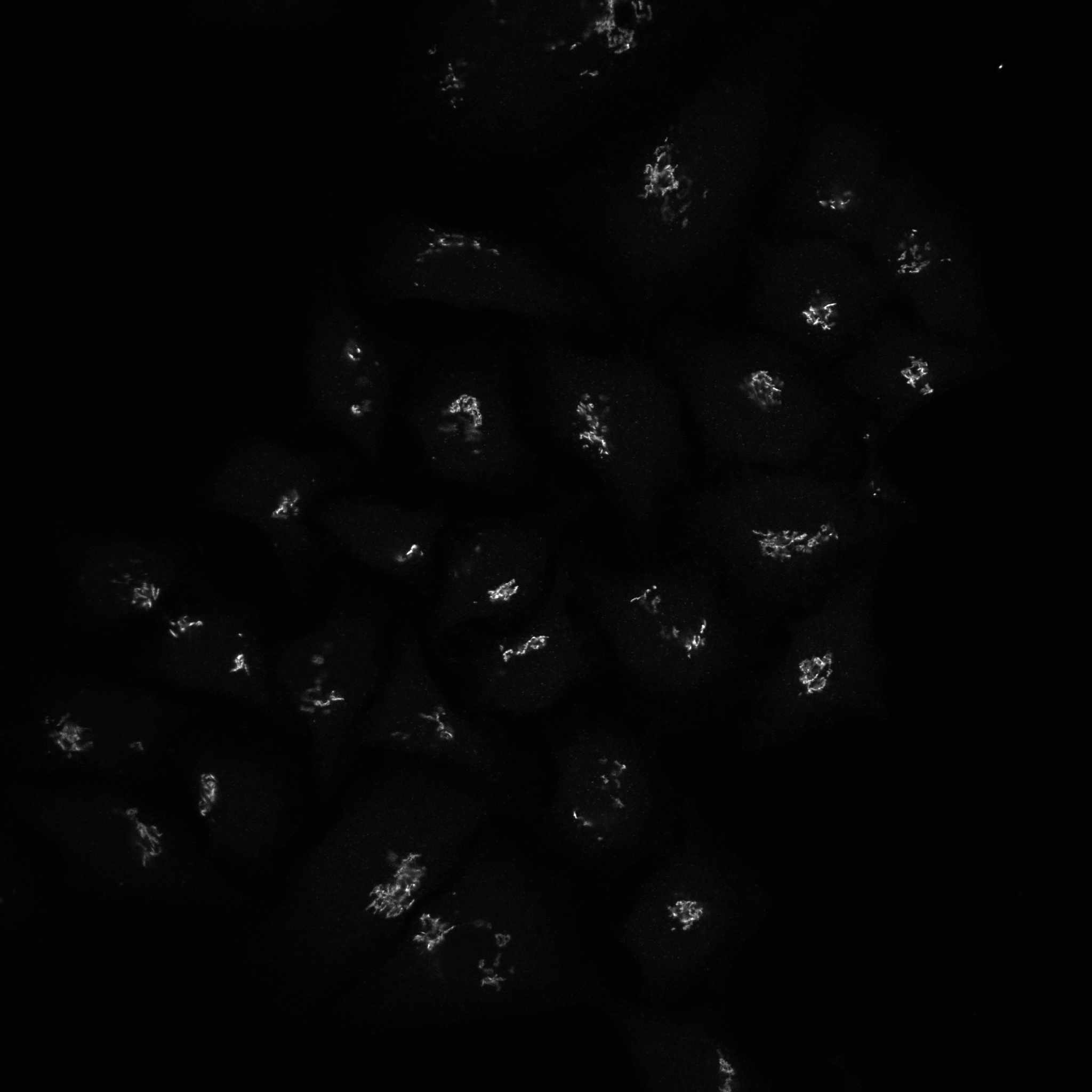

Supplement: Supplementary file 13 — EV and Appendix Figure Source Data [file 44318_2024_131_MOESM13_ESM.zip › ExpandedFigure 1/EV1B/FigureEV1B_GM130_Growing_WT.tif]

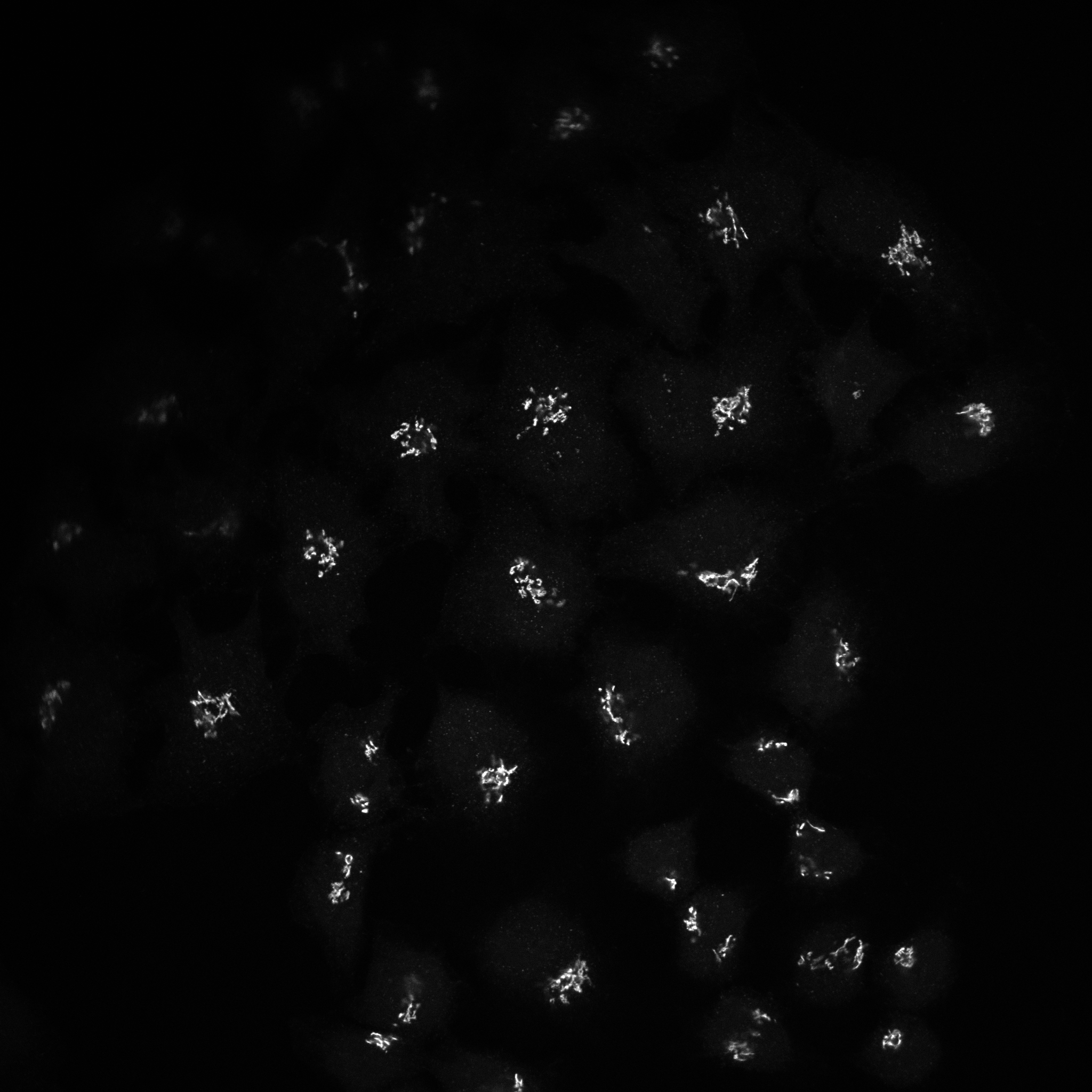

Supplement: Supplementary file 13 — EV and Appendix Figure Source Data [file 44318_2024_131_MOESM13_ESM.zip › ExpandedFigure 1/EV1B/FigureEV1B_GM130_Growing_YIPF4KO.tif]

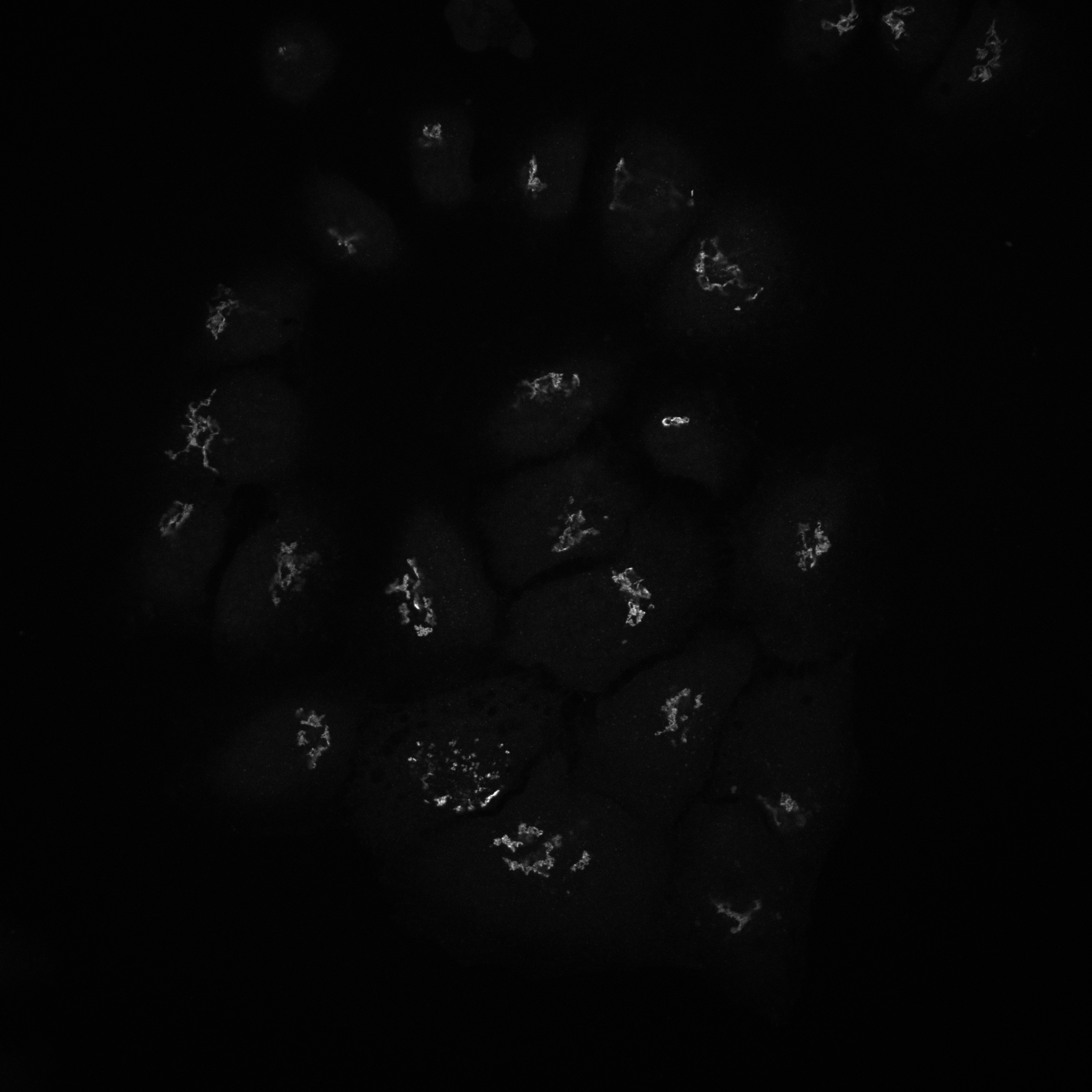

Supplement: Supplementary file 13 — EV and Appendix Figure Source Data [file 44318_2024_131_MOESM13_ESM.zip › ExpandedFigure 1/EV1B/FigureEV1B_GM130_Starvation_WT.tif]

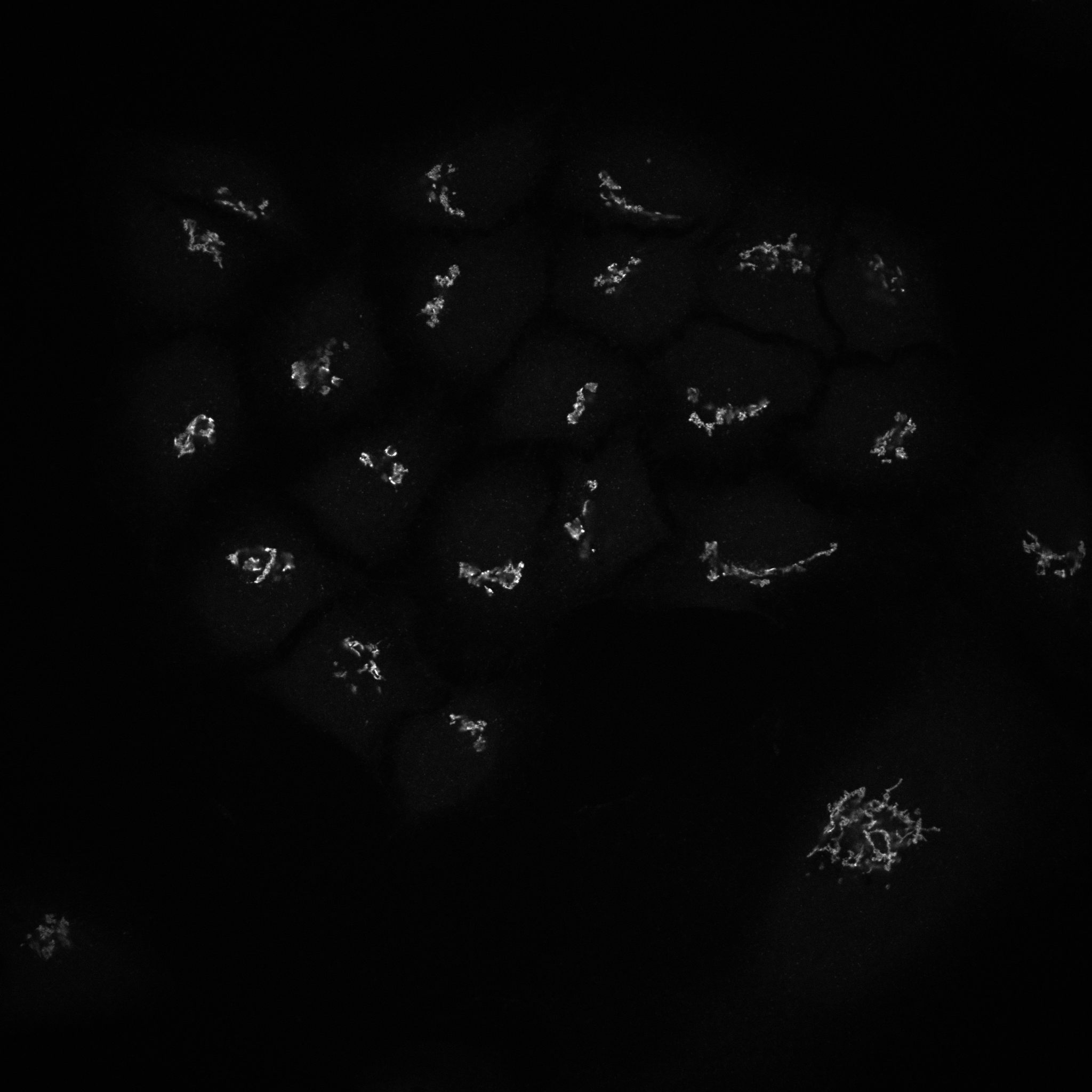

Supplement: Supplementary file 13 — EV and Appendix Figure Source Data [file 44318_2024_131_MOESM13_ESM.zip › ExpandedFigure 1/EV1B/FigureEV1B_GM130_Starvation_YIPF3KO.tif]

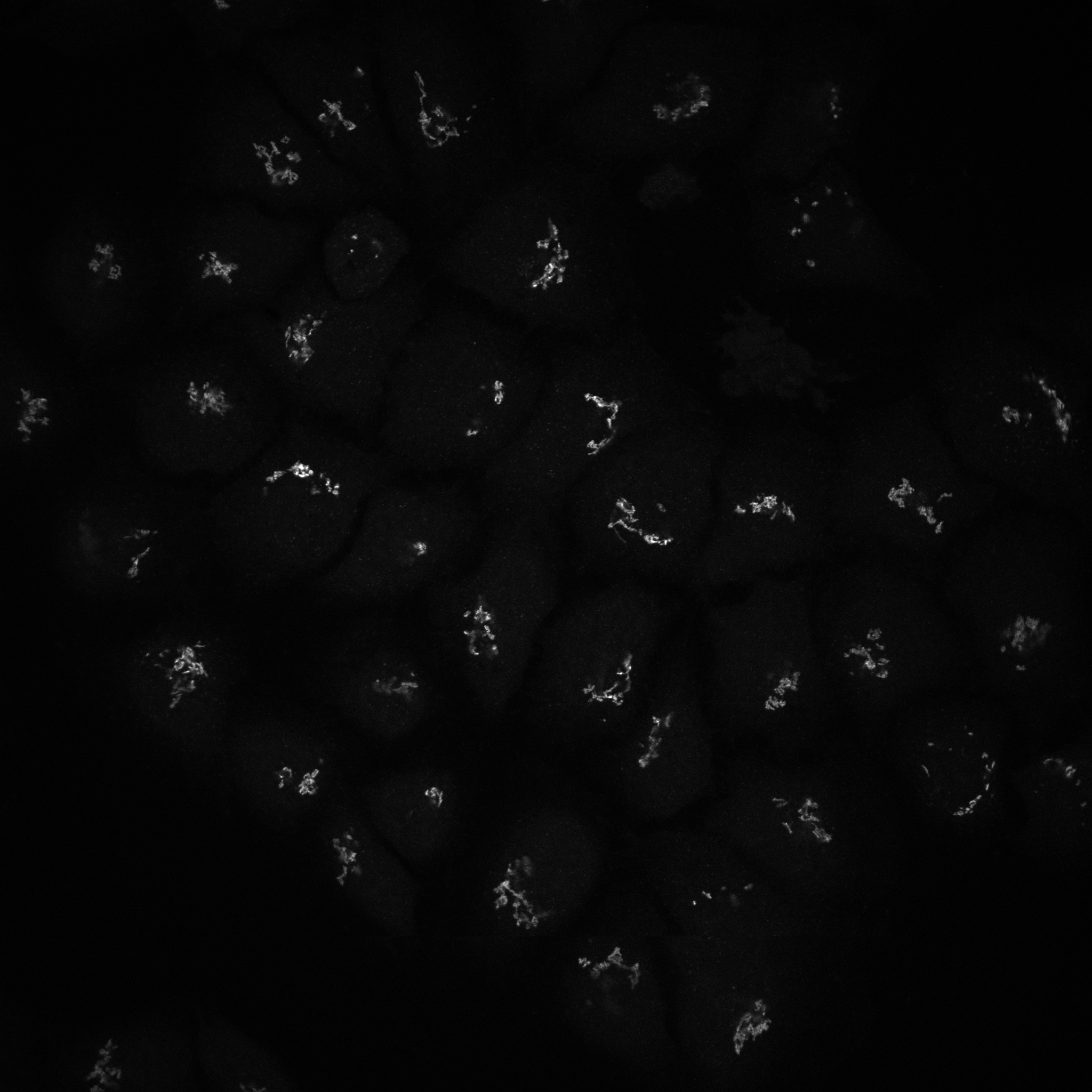

Supplement: Supplementary file 13 — EV and Appendix Figure Source Data [file 44318_2024_131_MOESM13_ESM.zip › ExpandedFigure 1/EV1B/FigureEV1B_GM130_Starvation_YIPF4KO.tif]

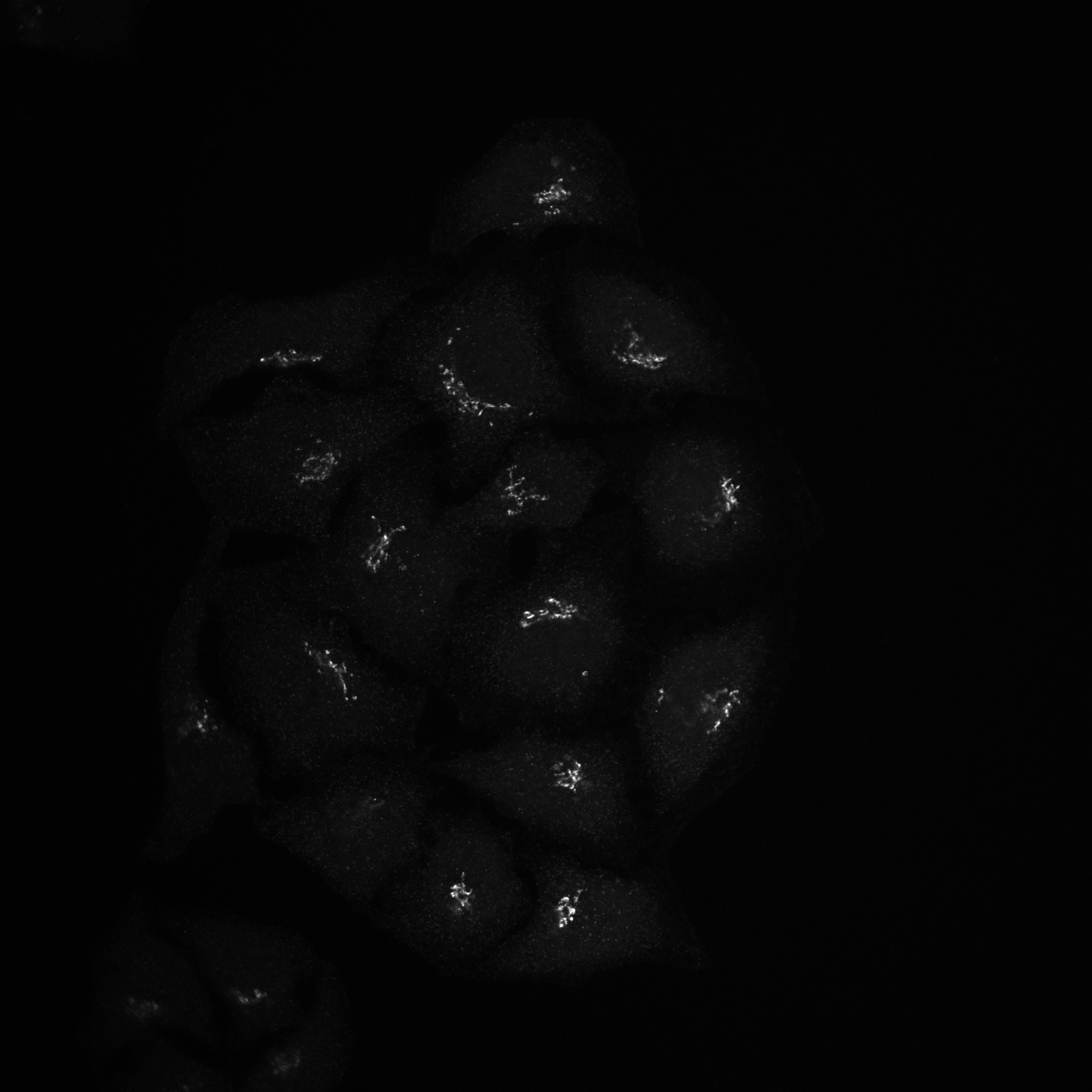

Supplement: Supplementary file 13 — EV and Appendix Figure Source Data [file 44318_2024_131_MOESM13_ESM.zip › ExpandedFigure 1/EV1B/FigureEV1B_p230_Growing_WT.tif]

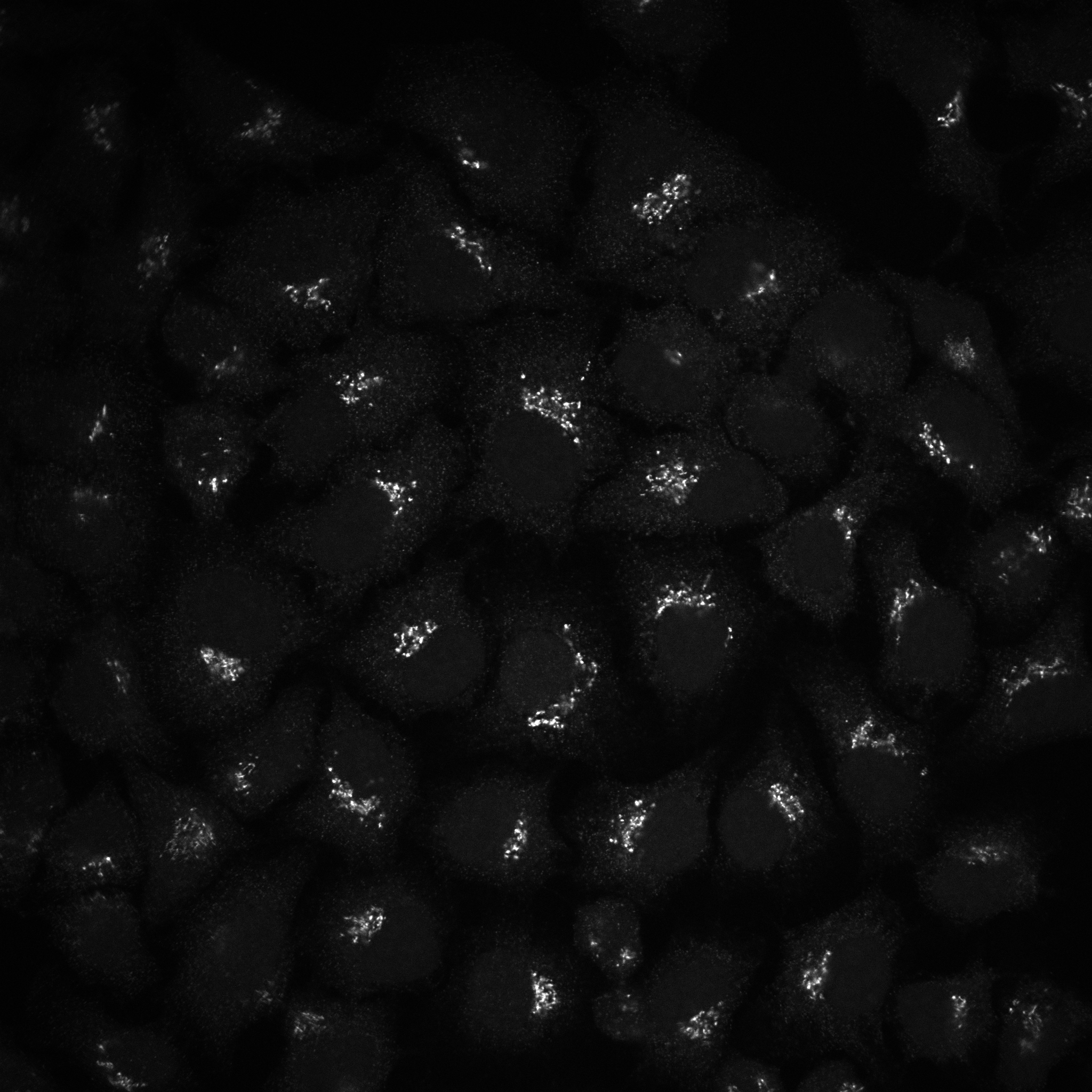

Supplement: Supplementary file 13 — EV and Appendix Figure Source Data [file 44318_2024_131_MOESM13_ESM.zip › ExpandedFigure 1/EV1B/FigureEV1B_p230_Growing_YIPF3KO.tif]

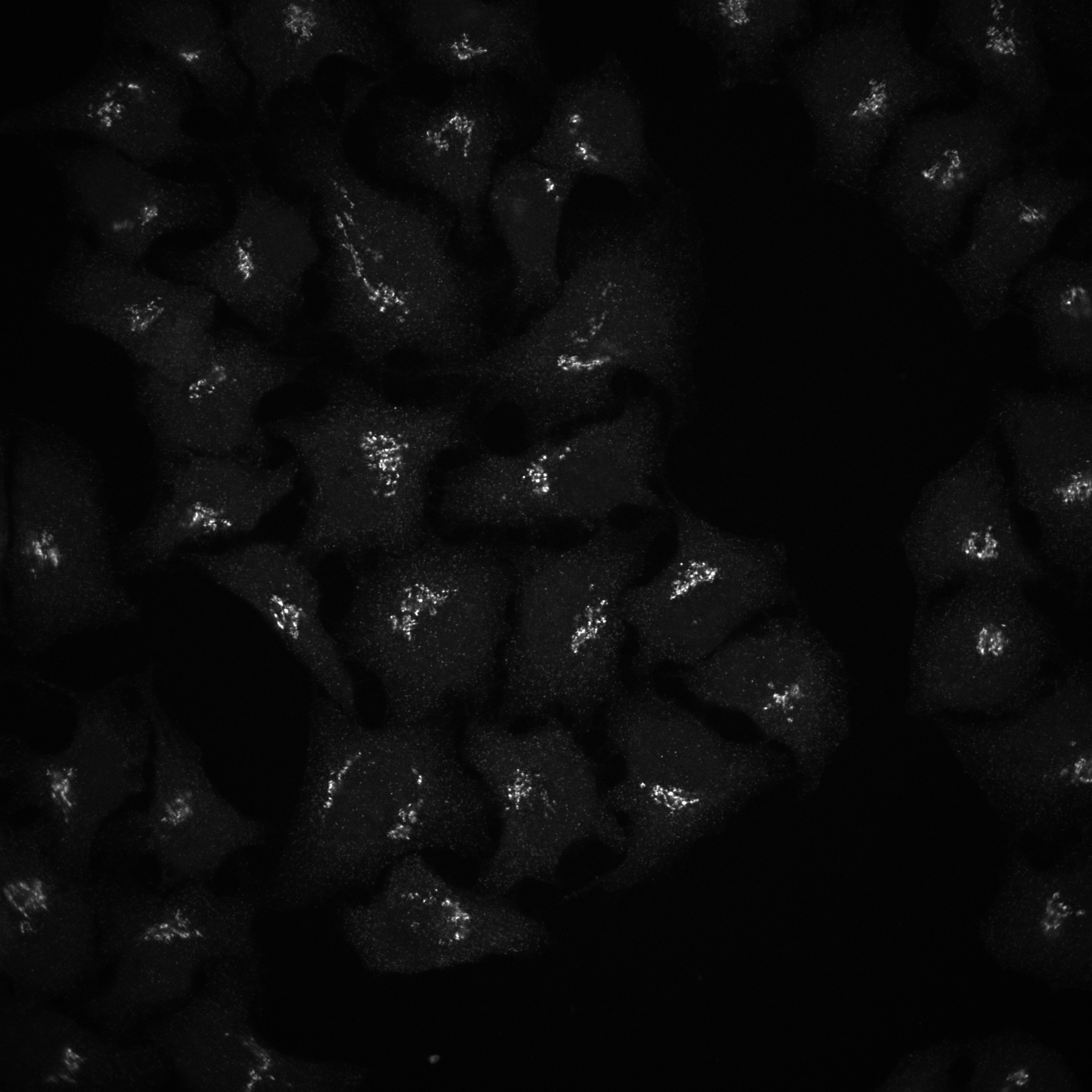

Supplement: Supplementary file 13 — EV and Appendix Figure Source Data [file 44318_2024_131_MOESM13_ESM.zip › ExpandedFigure 1/EV1B/FigureEV1B_p230_Growing_YIPF4KO.tif]

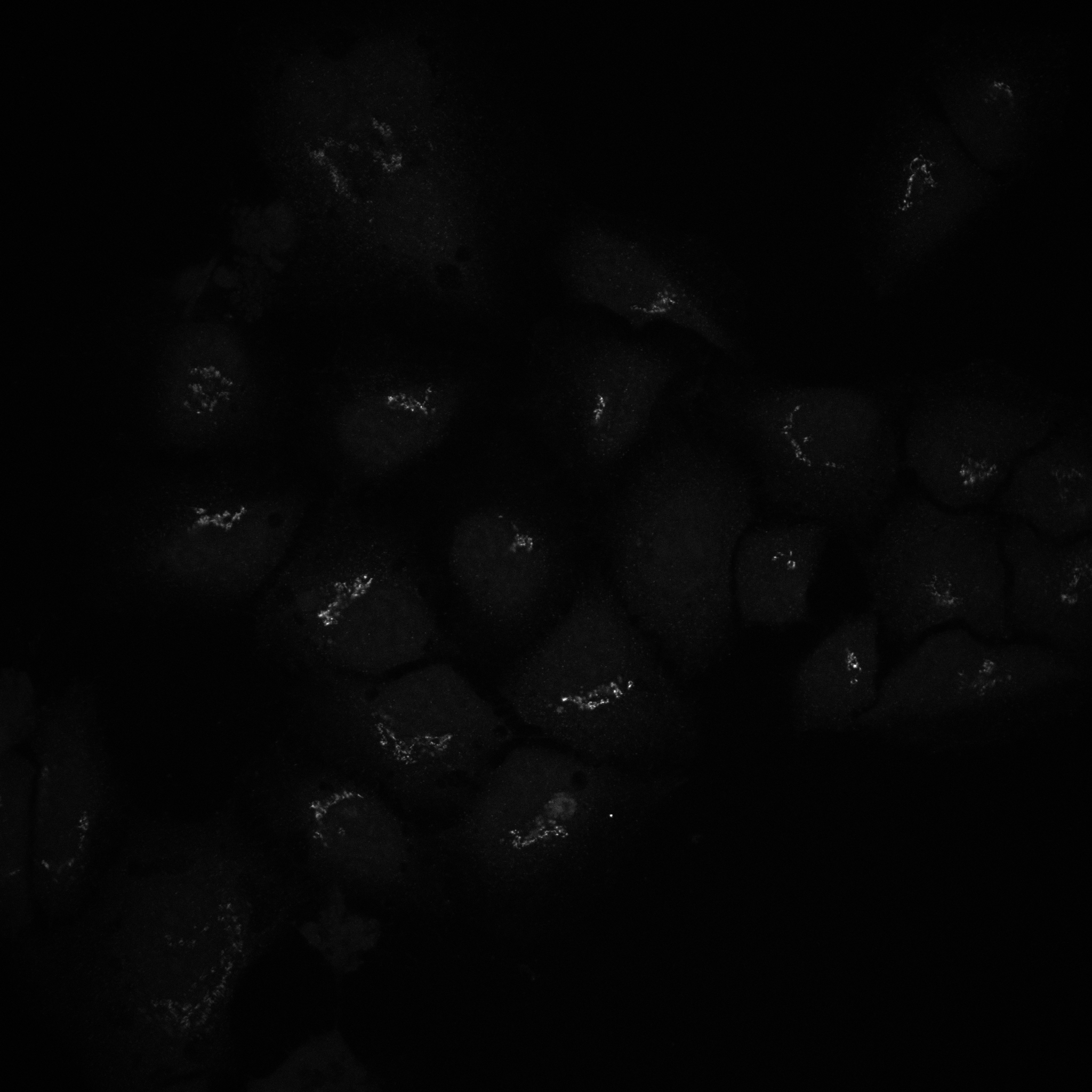

Supplement: Supplementary file 13 — EV and Appendix Figure Source Data [file 44318_2024_131_MOESM13_ESM.zip › ExpandedFigure 1/EV1B/FigureEV1B_p230_Starvation_WT.tif]

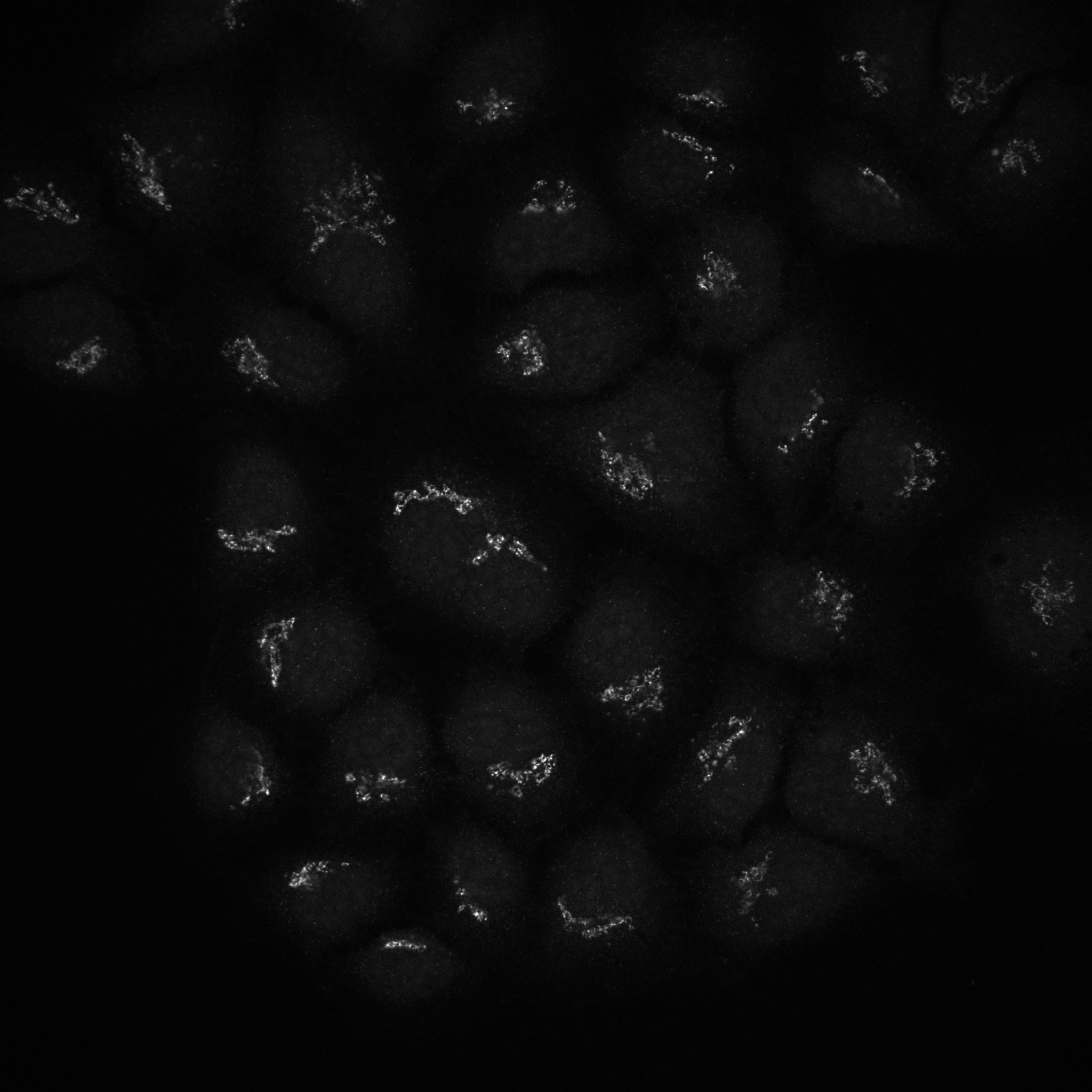

Supplement: Supplementary file 13 — EV and Appendix Figure Source Data [file 44318_2024_131_MOESM13_ESM.zip › ExpandedFigure 1/EV1B/FigureEV1B_p230_Starvation_YIPF3KO.tif]

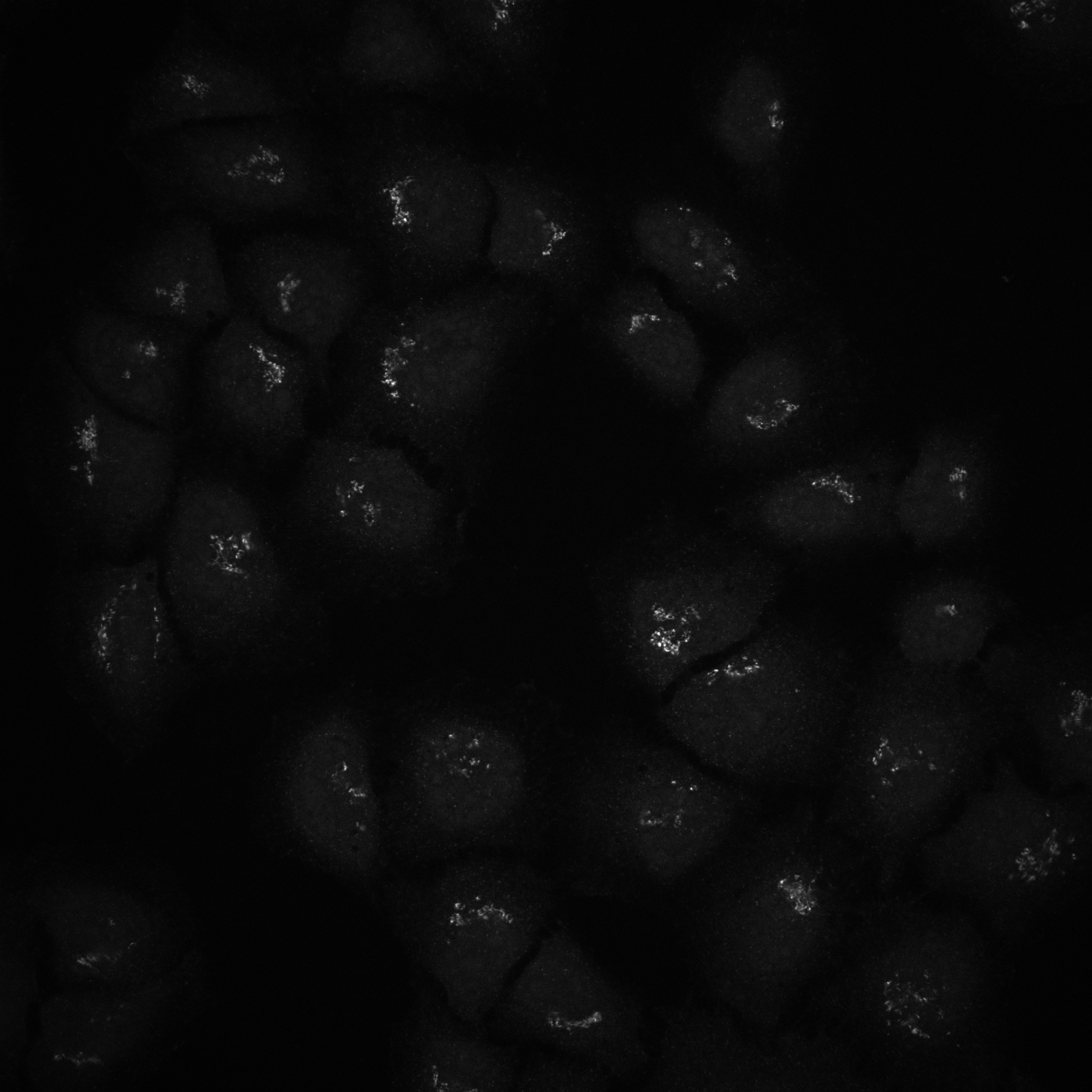

Supplement: Supplementary file 13 — EV and Appendix Figure Source Data [file 44318_2024_131_MOESM13_ESM.zip › ExpandedFigure 1/EV1B/FigureEV1B_p230_Starvation_YIPF4KO.tif]

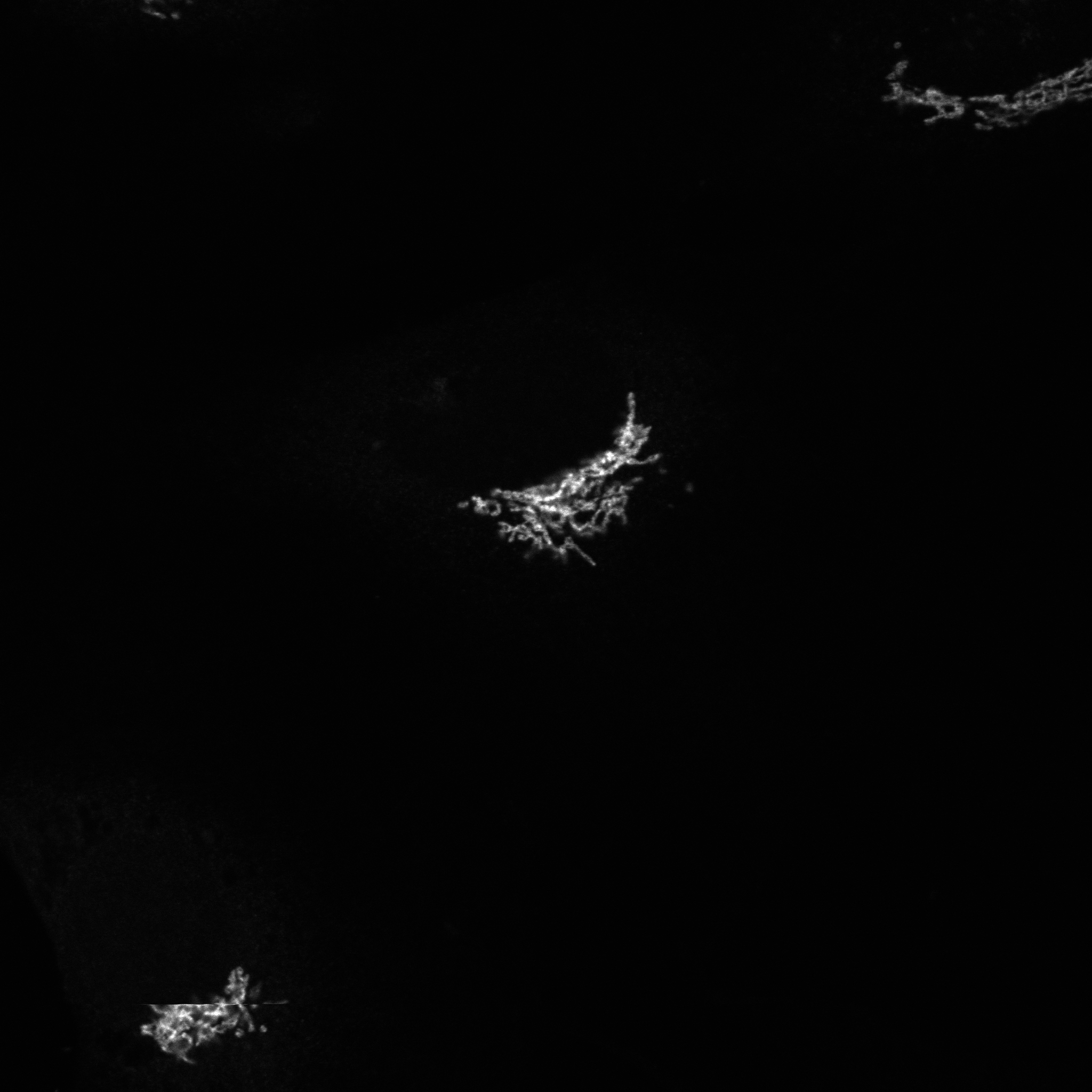

Supplement: Supplementary file 13 — EV and Appendix Figure Source Data [file 44318_2024_131_MOESM13_ESM.zip › ExpandedFigure 2/EV2A/FigureEV2A_EGFP-YIPF3_EGFP.tif]

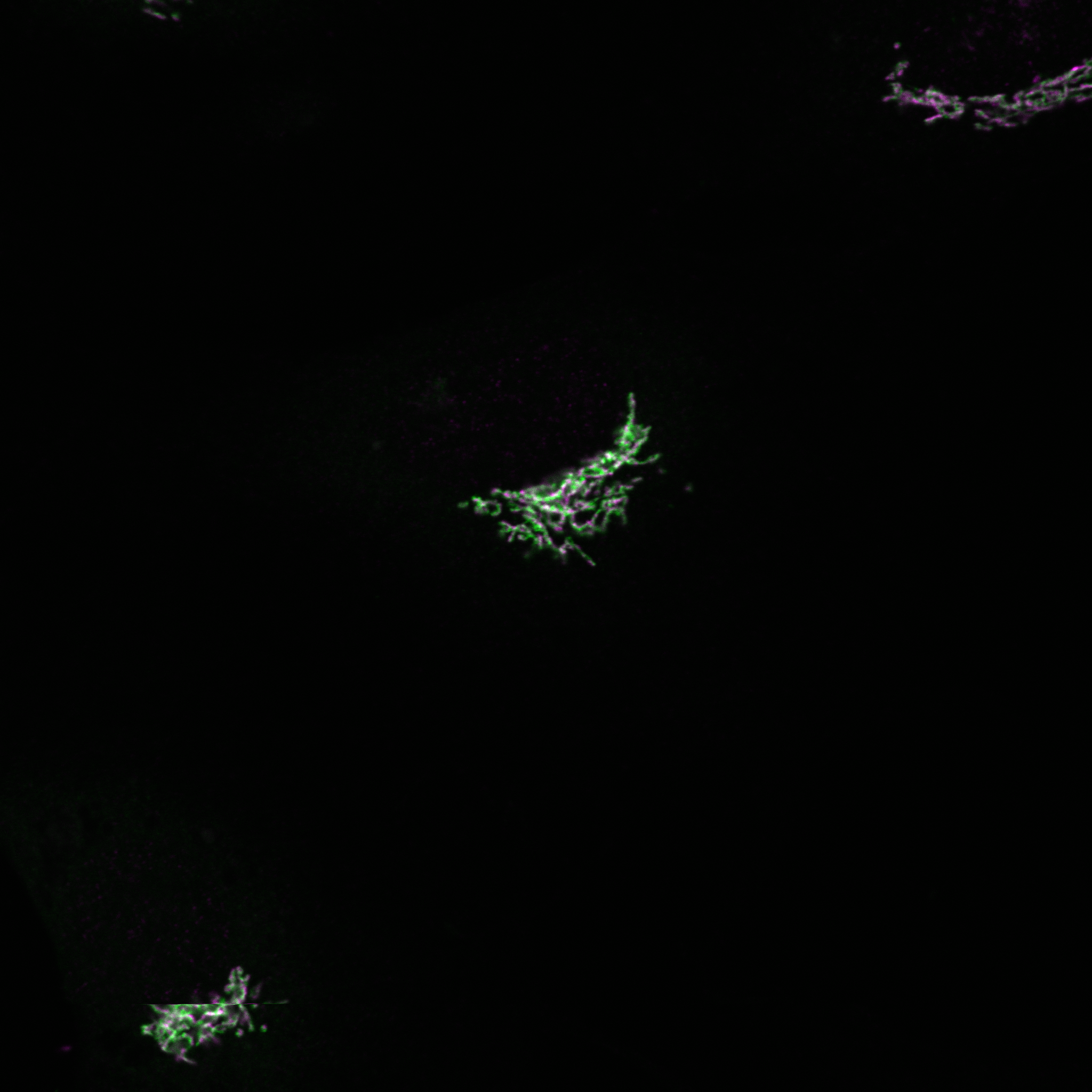

Supplement: Supplementary file 13 — EV and Appendix Figure Source Data [file 44318_2024_131_MOESM13_ESM.zip › ExpandedFigure 2/EV2A/FigureEV2A_EGFP-YIPF3_merge.tif]

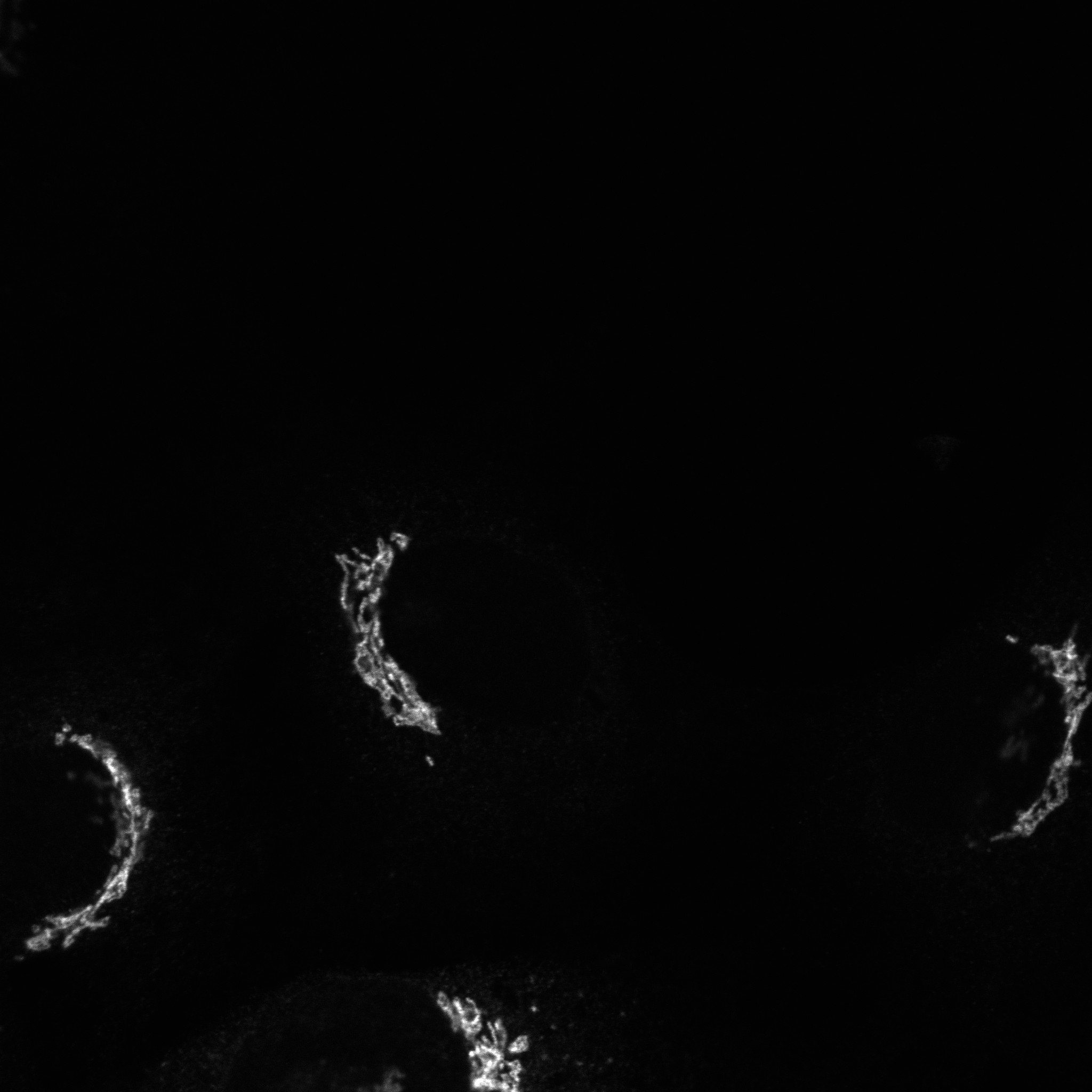

Supplement: Supplementary file 13 — EV and Appendix Figure Source Data [file 44318_2024_131_MOESM13_ESM.zip › ExpandedFigure 2/EV2A/FigureEV2A_EGFP-YIPF4_EGFP.tif]

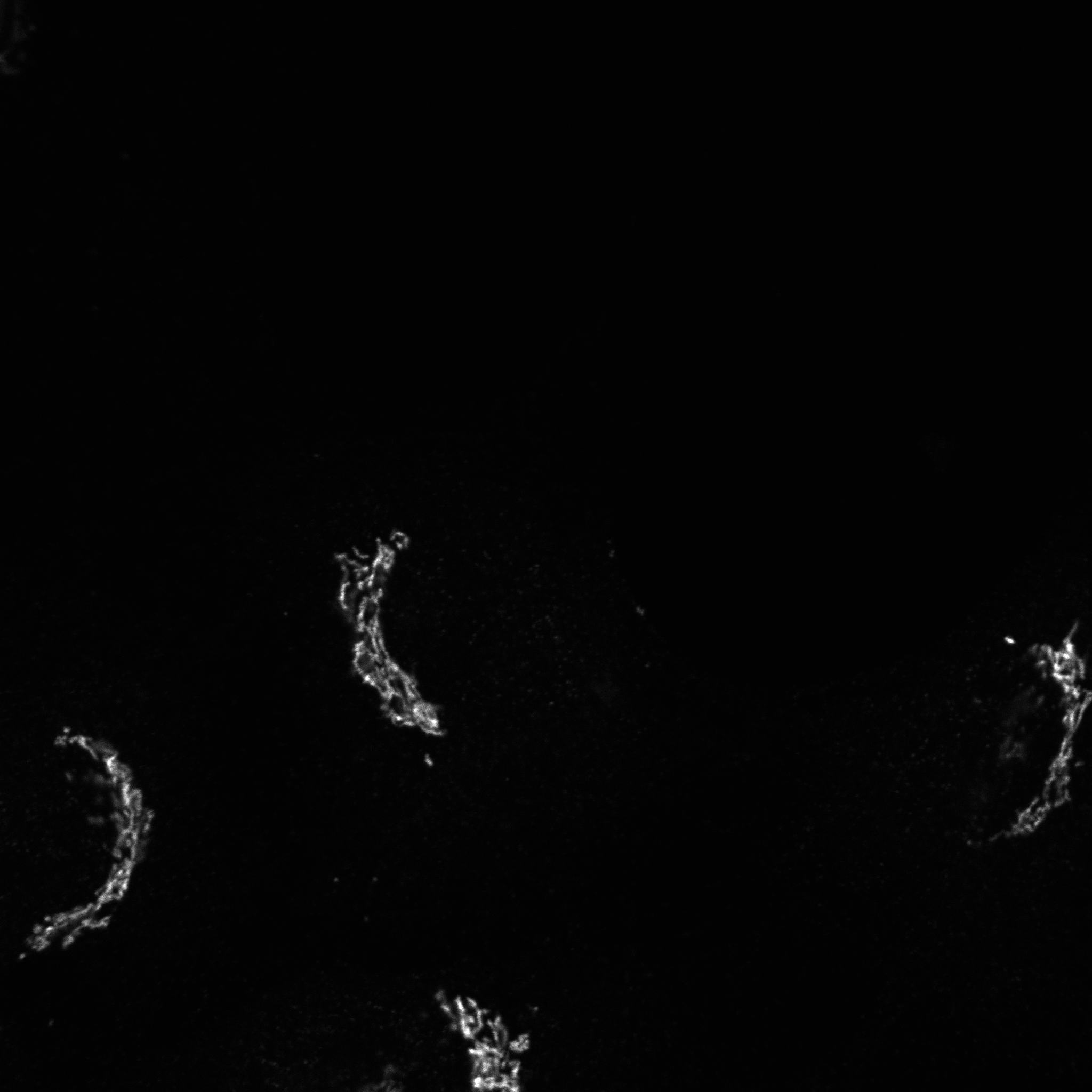

Supplement: Supplementary file 13 — EV and Appendix Figure Source Data [file 44318_2024_131_MOESM13_ESM.zip › ExpandedFigure 2/EV2A/FigureEV2A_EGFP-YIPF4_GM130.tif]

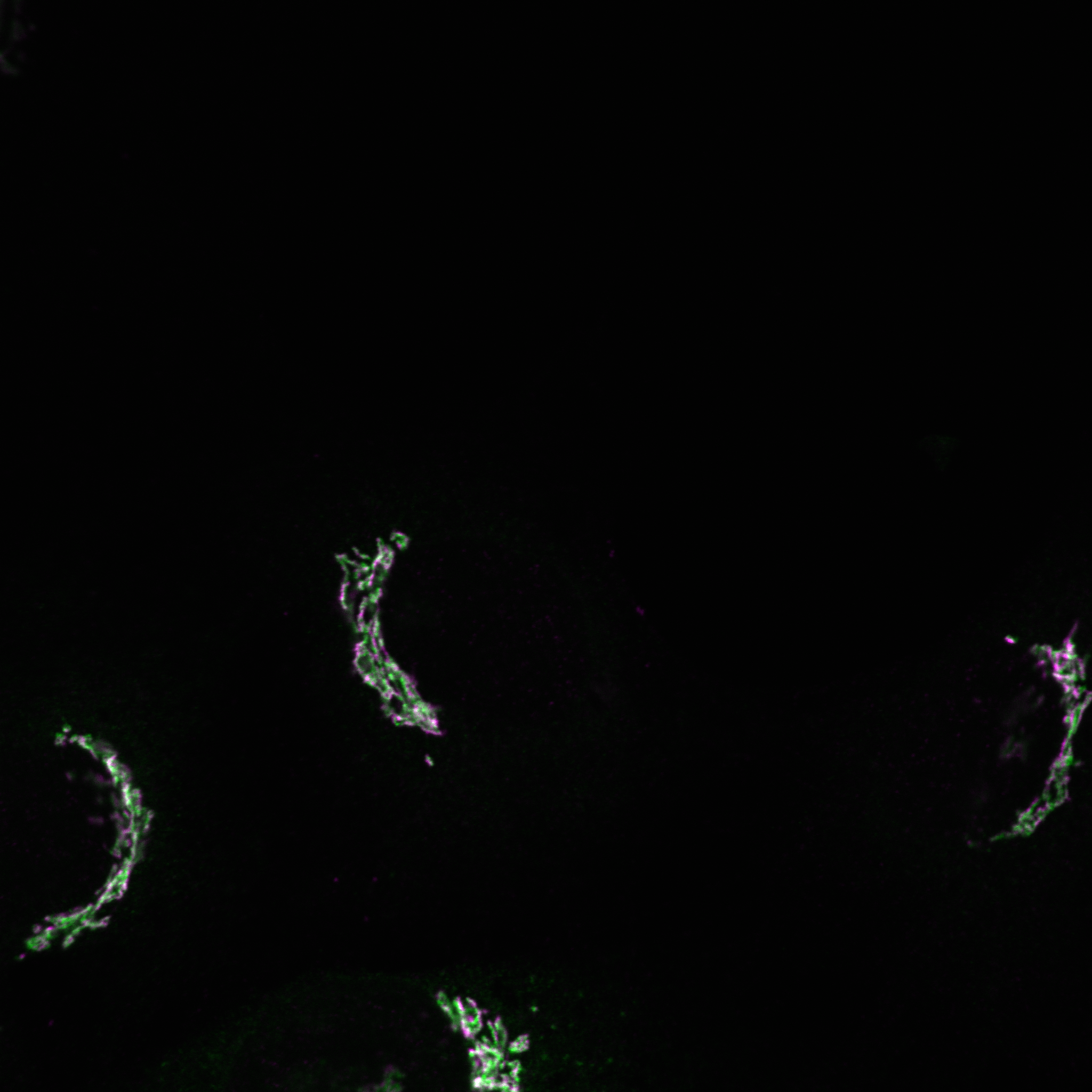

Supplement: Supplementary file 13 — EV and Appendix Figure Source Data [file 44318_2024_131_MOESM13_ESM.zip › ExpandedFigure 2/EV2A/FigureEV2A_EGFP-YIPF4_merge.tif]

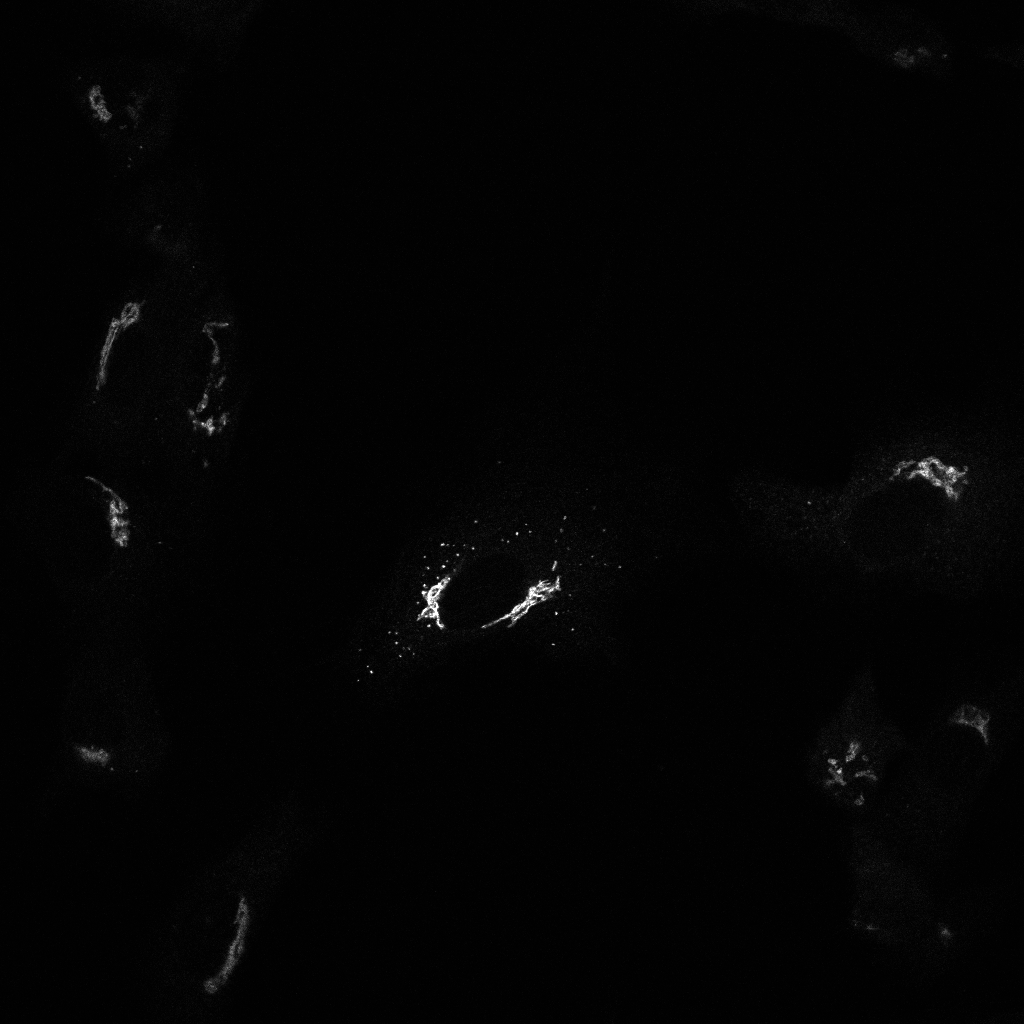

Supplement: Supplementary file 13 — EV and Appendix Figure Source Data [file 44318_2024_131_MOESM13_ESM.zip › ExpandedFigure 2/EV2B/FigureEV2B_EGFP-YIPF3_EGFP.tif]

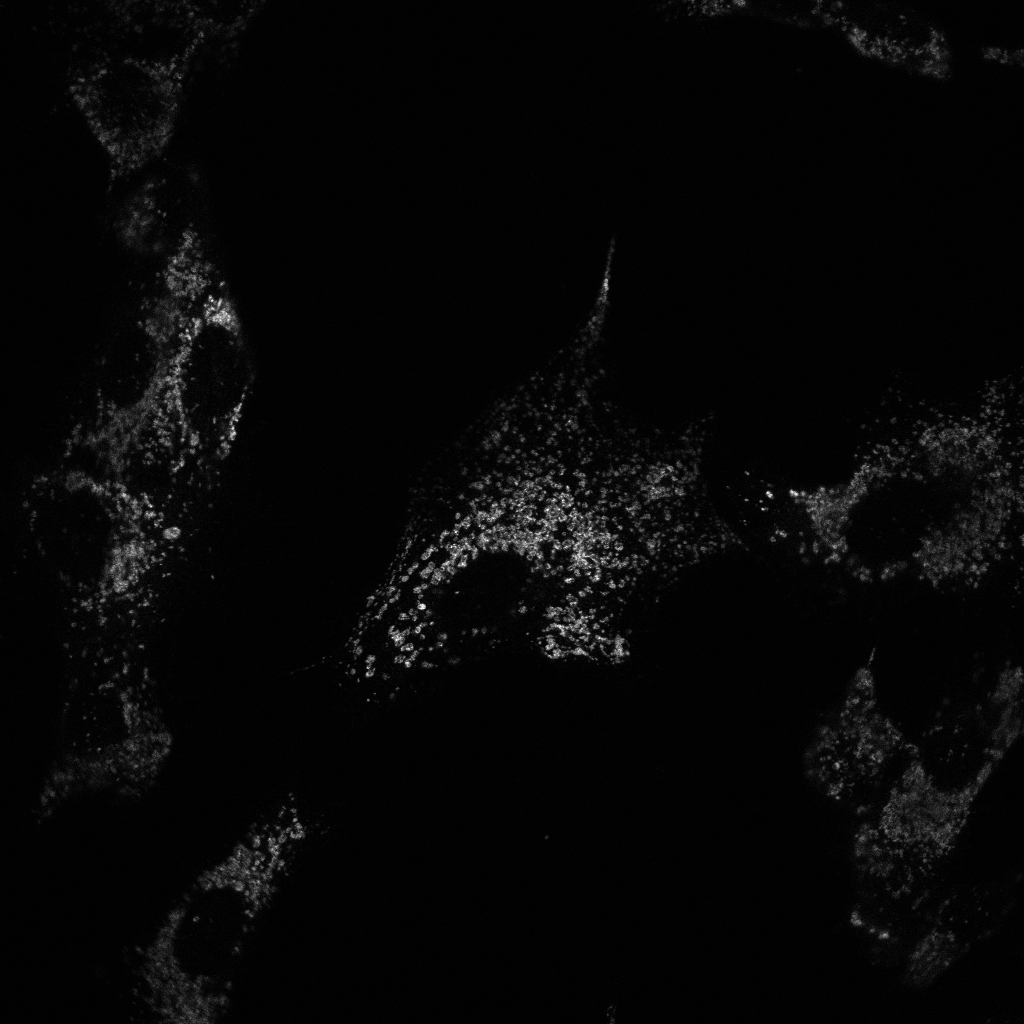

Supplement: Supplementary file 13 — EV and Appendix Figure Source Data [file 44318_2024_131_MOESM13_ESM.zip › ExpandedFigure 2/EV2B/FigureEV2B_EGFP-YIPF3_LAMP1.tif]

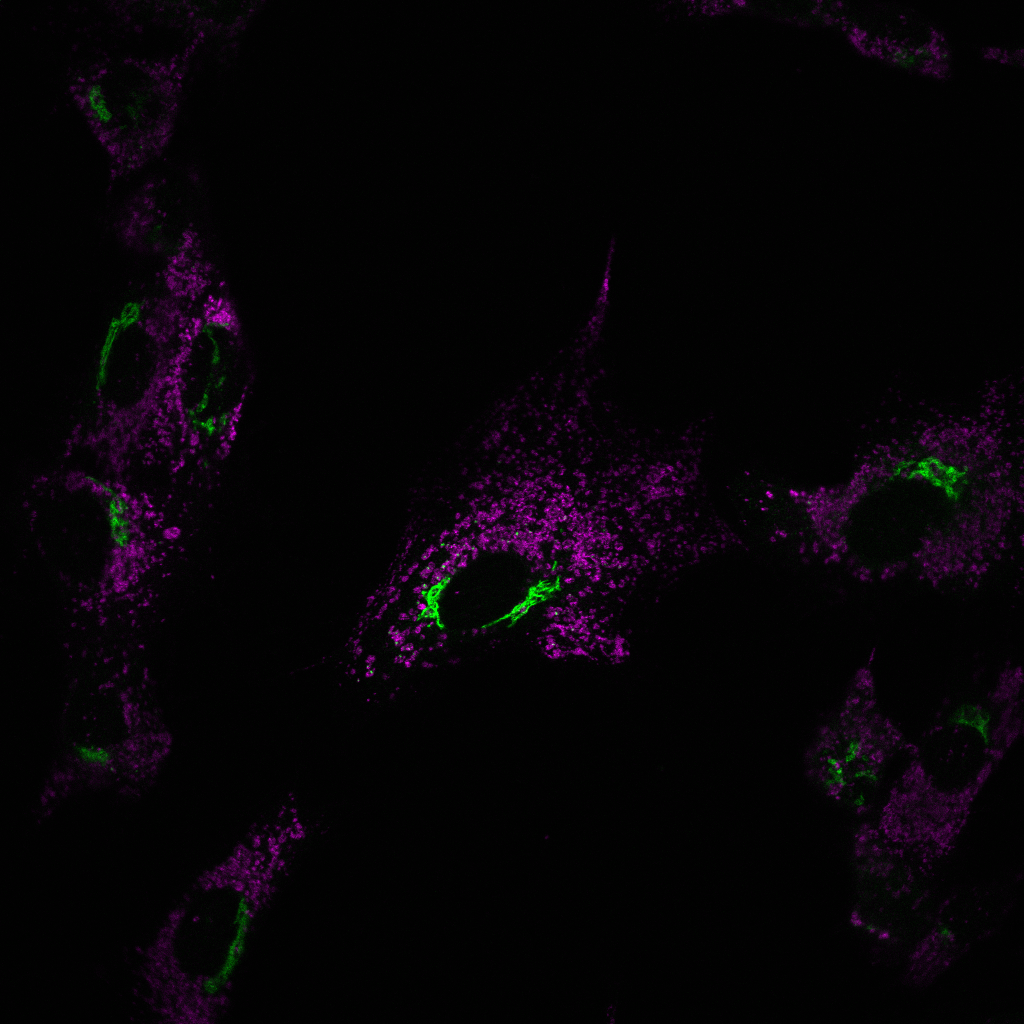

Supplement: Supplementary file 13 — EV and Appendix Figure Source Data [file 44318_2024_131_MOESM13_ESM.zip › ExpandedFigure 2/EV2B/FigureEV2B_EGFP-YIPF3_merge.tif]

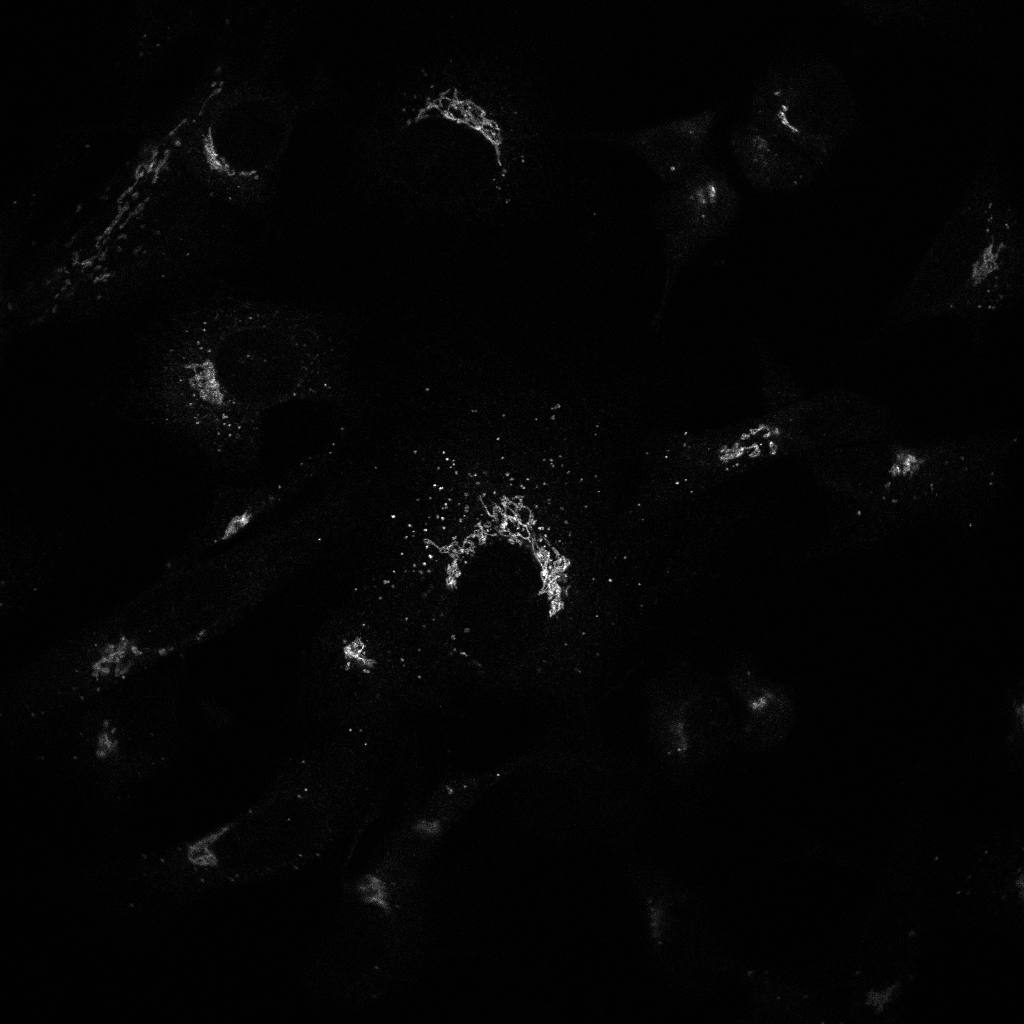

Supplement: Supplementary file 13 — EV and Appendix Figure Source Data [file 44318_2024_131_MOESM13_ESM.zip › ExpandedFigure 2/EV2B/FigureEV2B_EGFP-YIPF4_EGFP.tif]

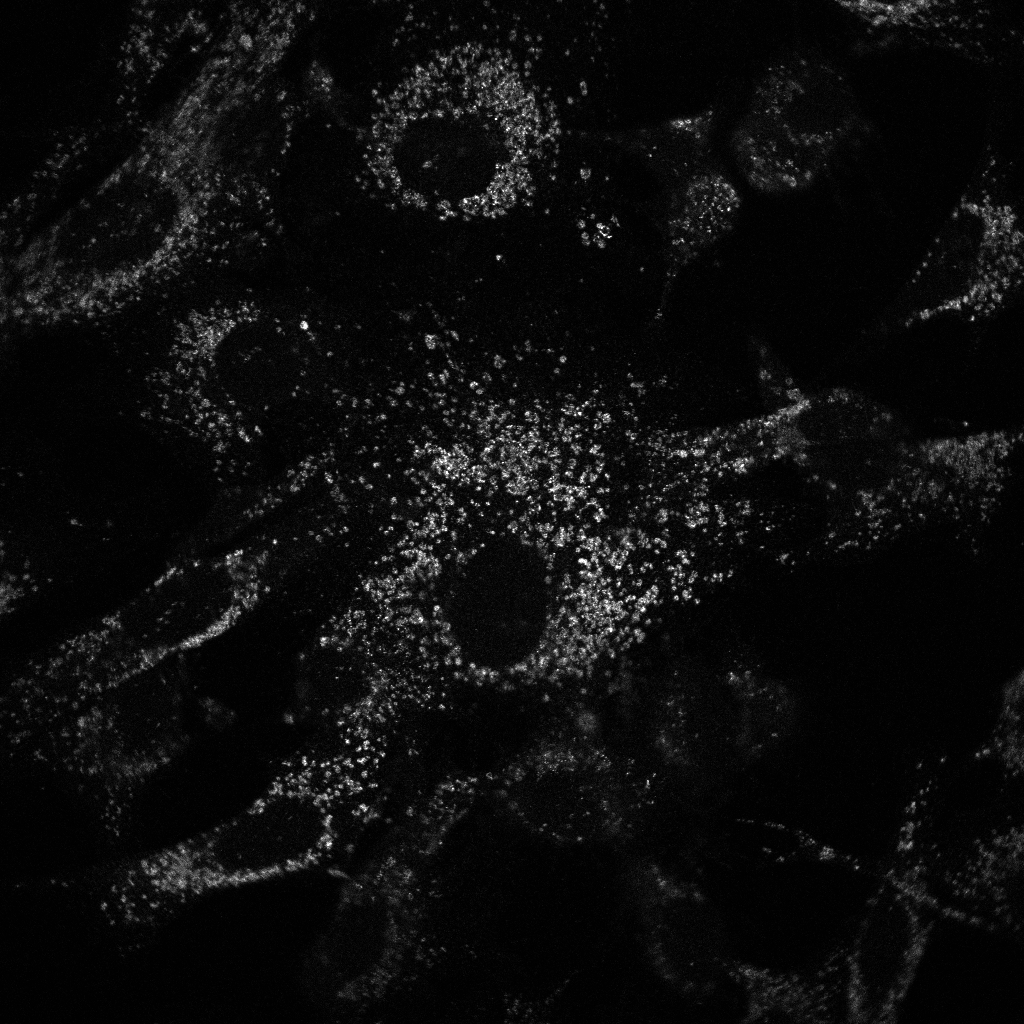

Supplement: Supplementary file 13 — EV and Appendix Figure Source Data [file 44318_2024_131_MOESM13_ESM.zip › ExpandedFigure 2/EV2B/FigureEV2B_EGFP-YIPF4_LAMP1.tif]

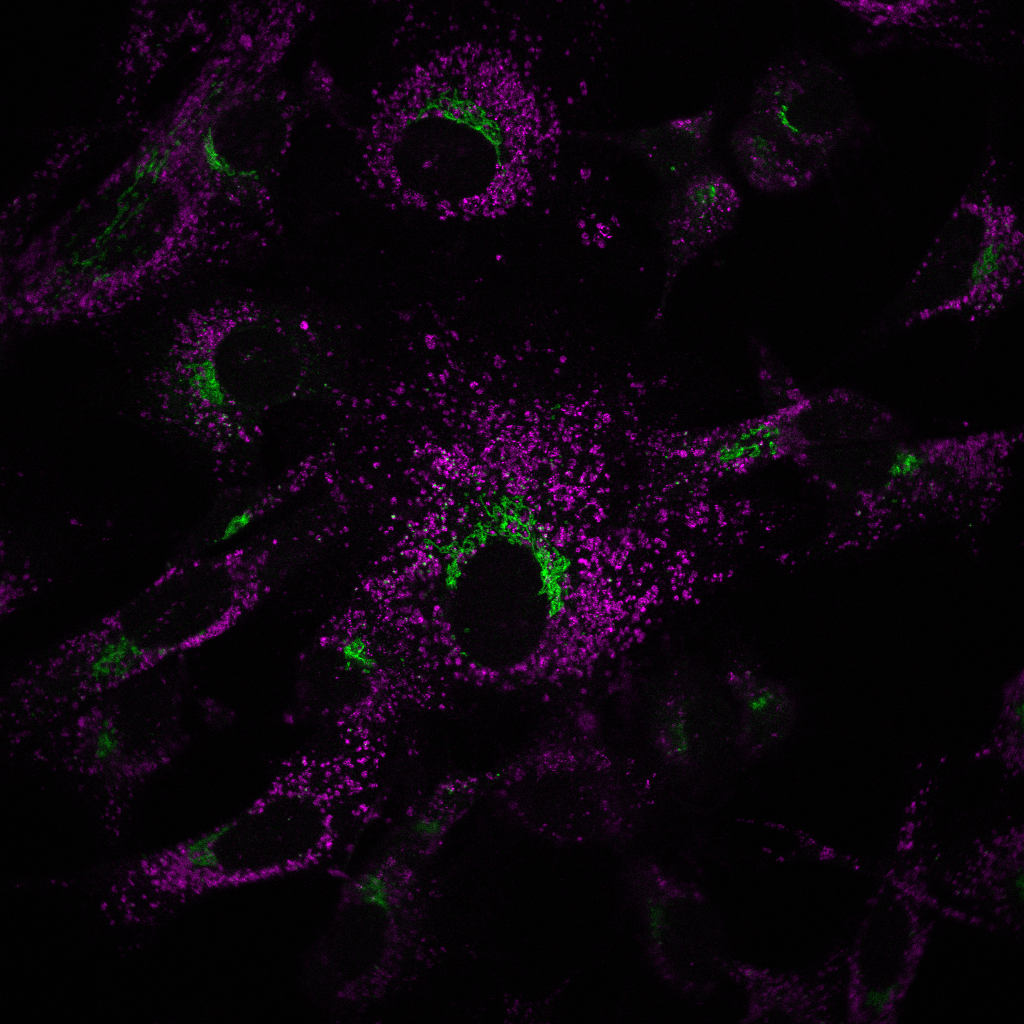

Supplement: Supplementary file 13 — EV and Appendix Figure Source Data [file 44318_2024_131_MOESM13_ESM.zip › ExpandedFigure 2/EV2B/FigureEV2B_EGFP-YIPF4_merge.tif]

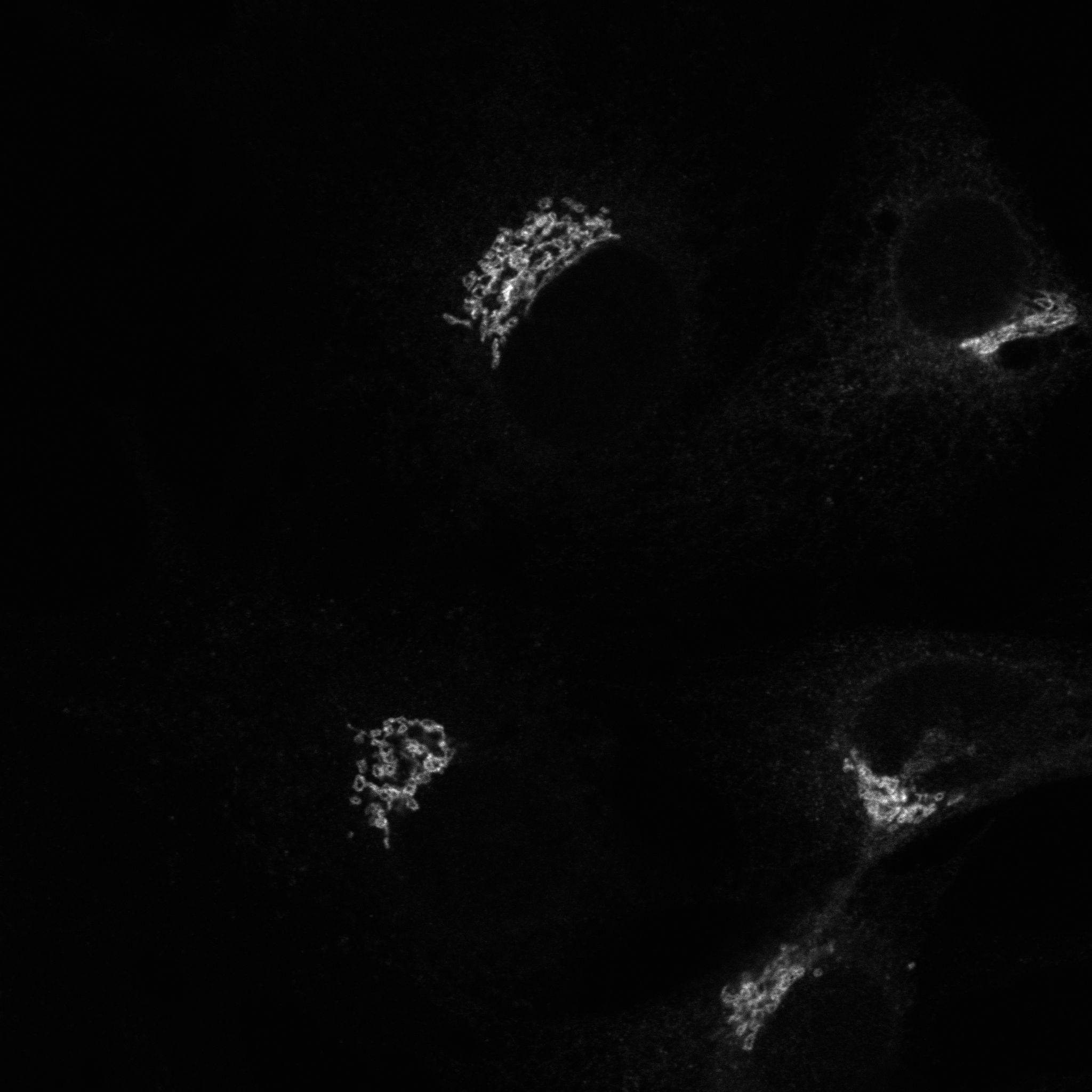

Supplement: Supplementary file 13 — EV and Appendix Figure Source Data [file 44318_2024_131_MOESM13_ESM.zip › ExpandedFigure 2/EV2C/FigureEV2C_MAN2A1-mCherry_EGFP.tif]

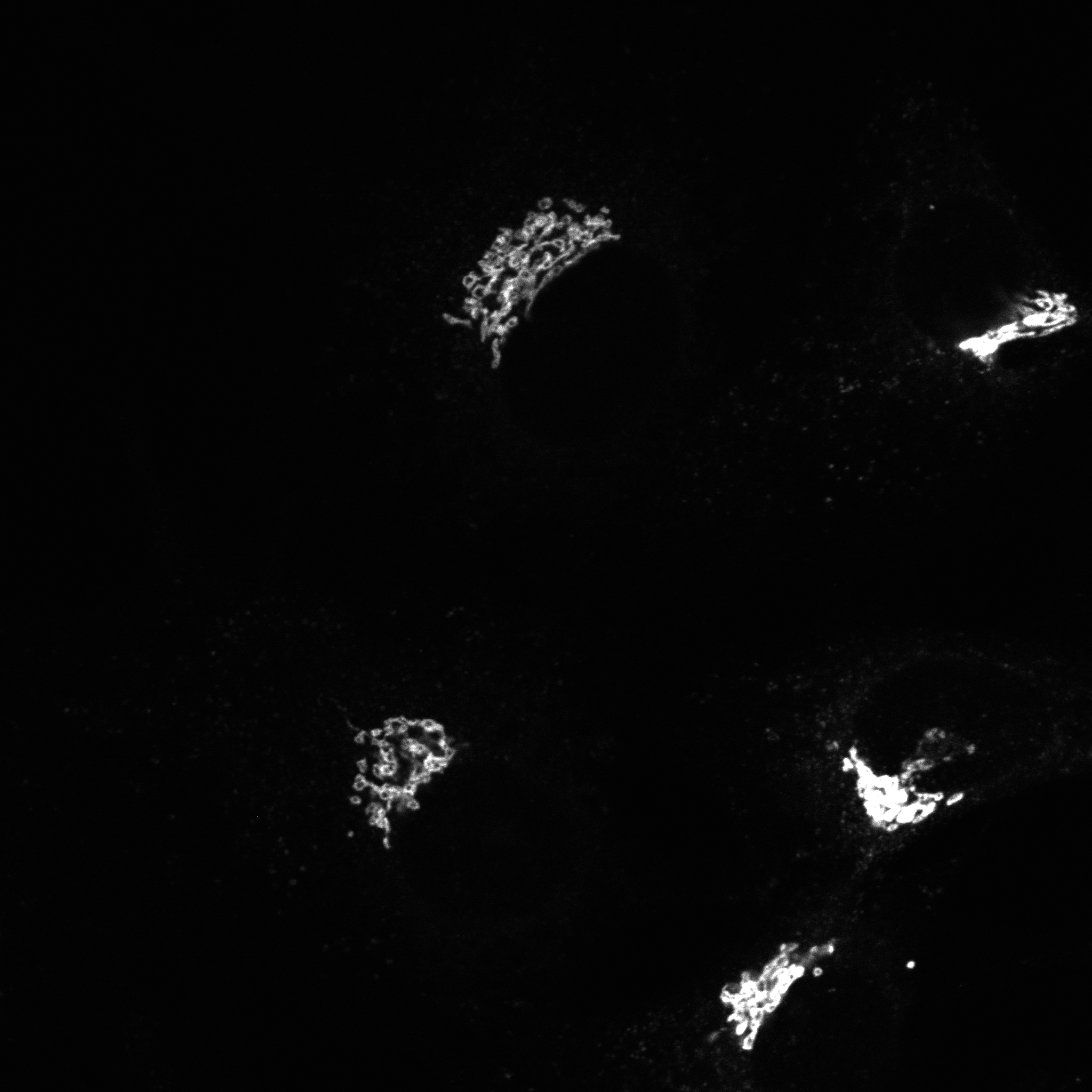

Supplement: Supplementary file 13 — EV and Appendix Figure Source Data [file 44318_2024_131_MOESM13_ESM.zip › ExpandedFigure 2/EV2C/FigureEV2C_MAN2A1-mCherry_mCherry.tif]

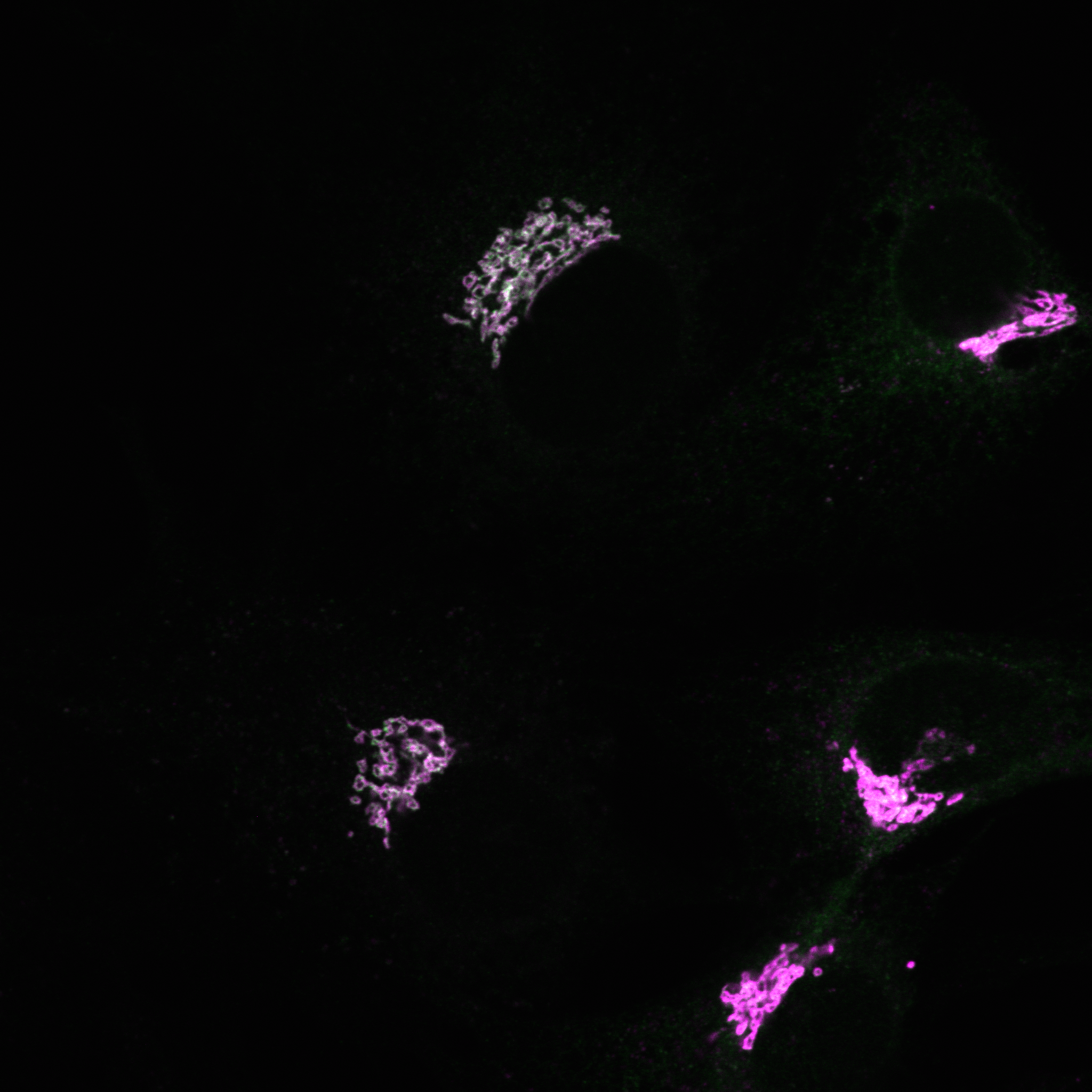

Supplement: Supplementary file 13 — EV and Appendix Figure Source Data [file 44318_2024_131_MOESM13_ESM.zip › ExpandedFigure 2/EV2C/FigureEV2C_MAN2A1-mCherry_merge.tif]

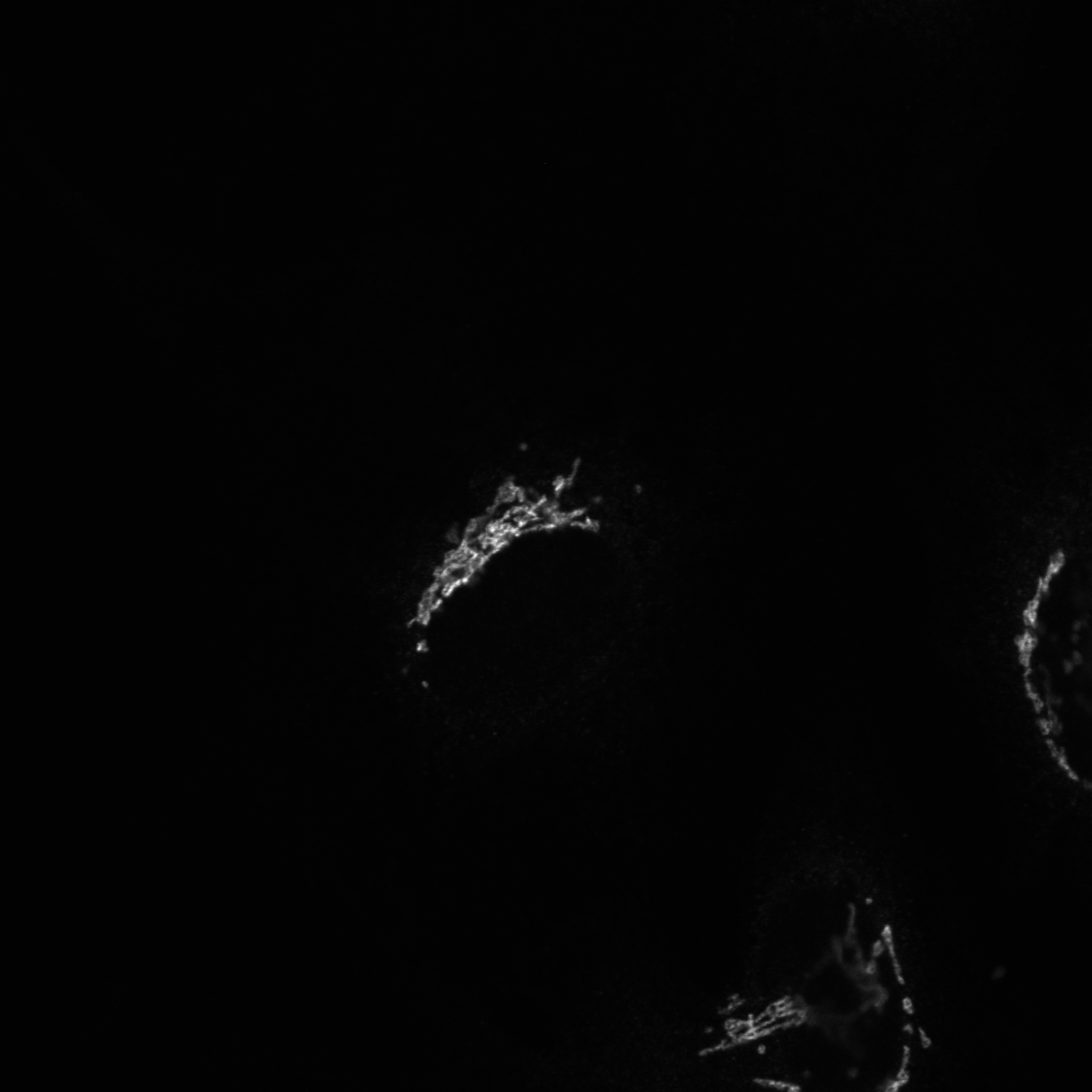

Supplement: Supplementary file 13 — EV and Appendix Figure Source Data [file 44318_2024_131_MOESM13_ESM.zip › ExpandedFigure 2/EV2C/FigureEV2C_TMEM165_EGFP.tif]

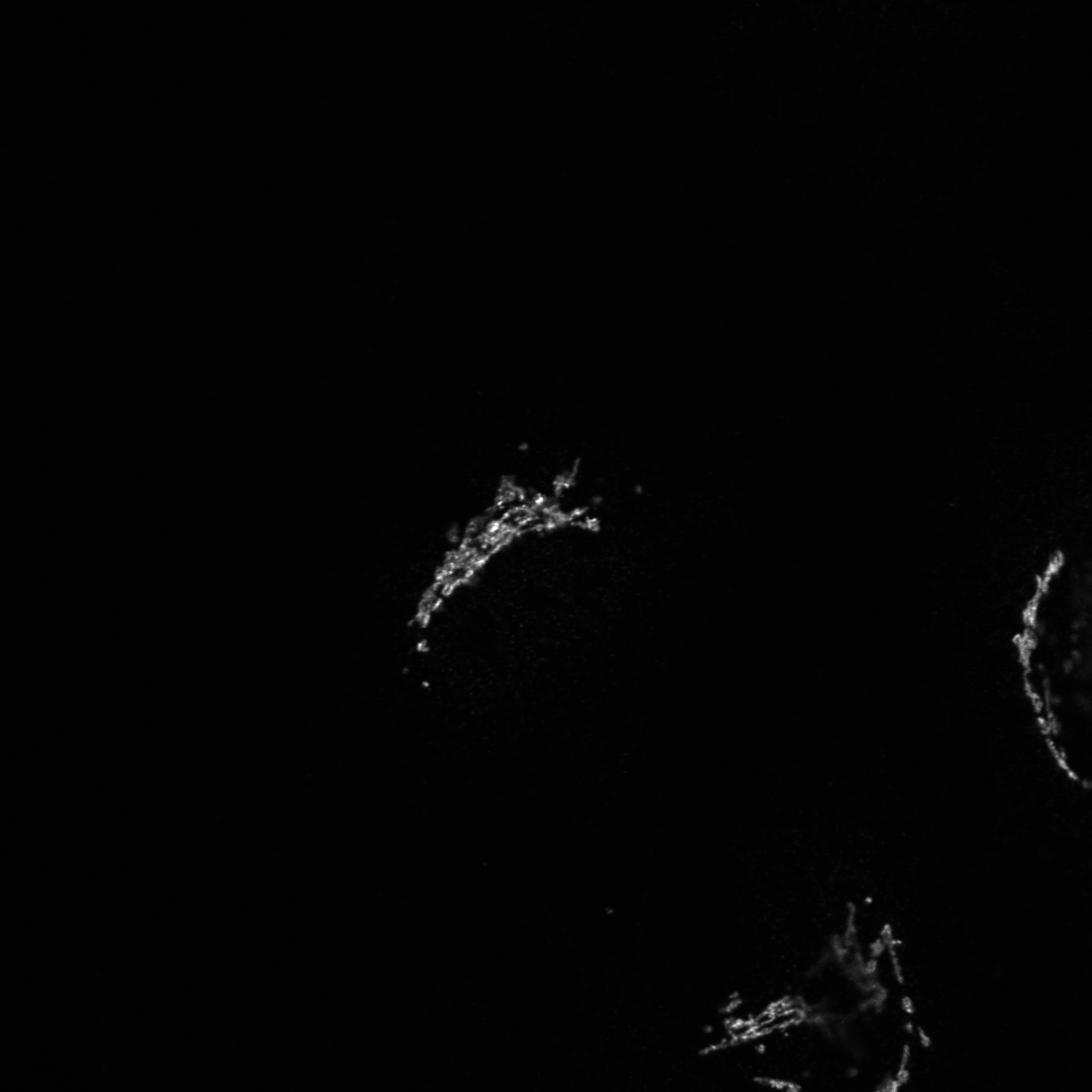

Supplement: Supplementary file 13 — EV and Appendix Figure Source Data [file 44318_2024_131_MOESM13_ESM.zip › ExpandedFigure 2/EV2C/FigureEV2C_TMEM165_TMEM165.tif]

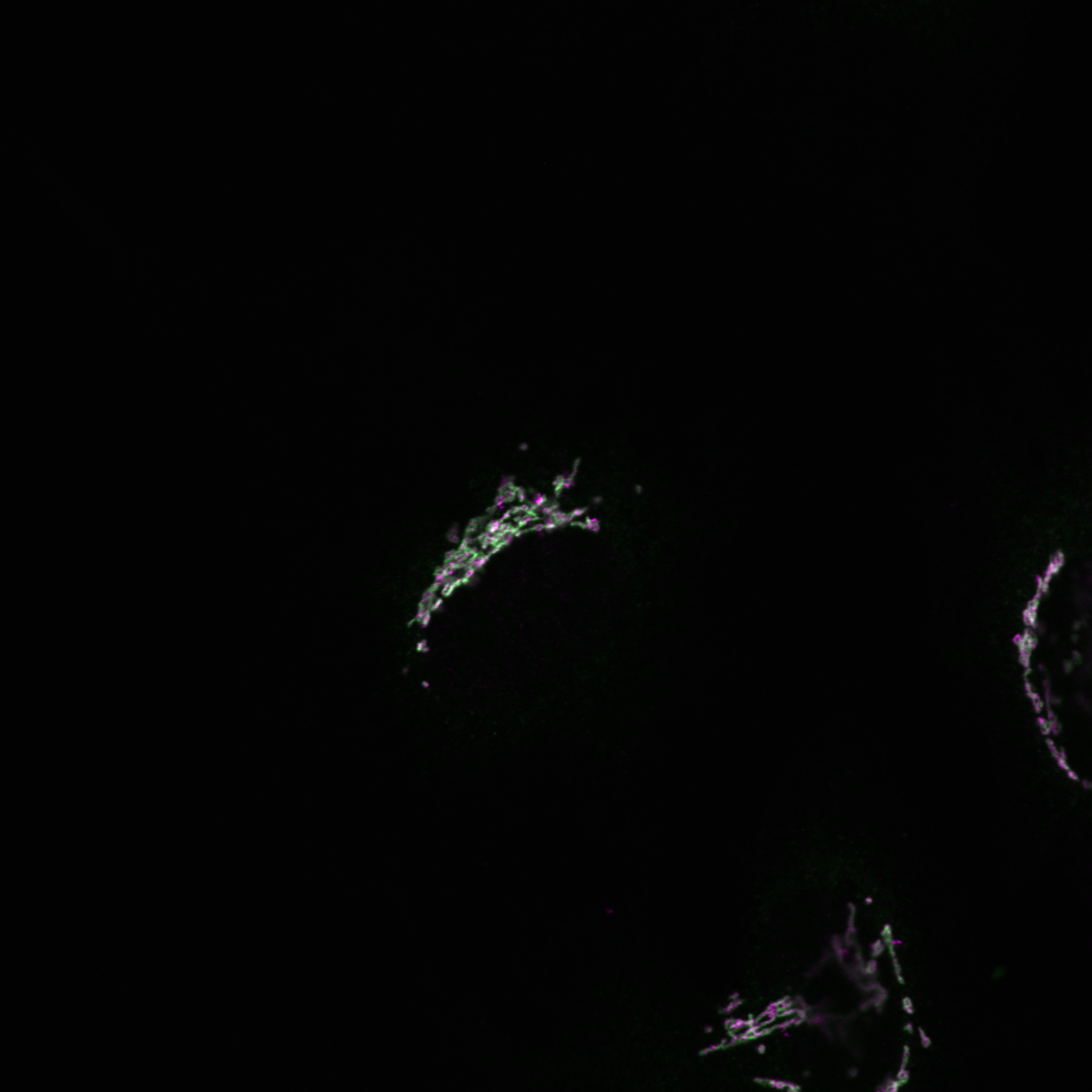

Supplement: Supplementary file 13 — EV and Appendix Figure Source Data [file 44318_2024_131_MOESM13_ESM.zip › ExpandedFigure 2/EV2C/FigureEV2C_TMEM165_merge.tif]

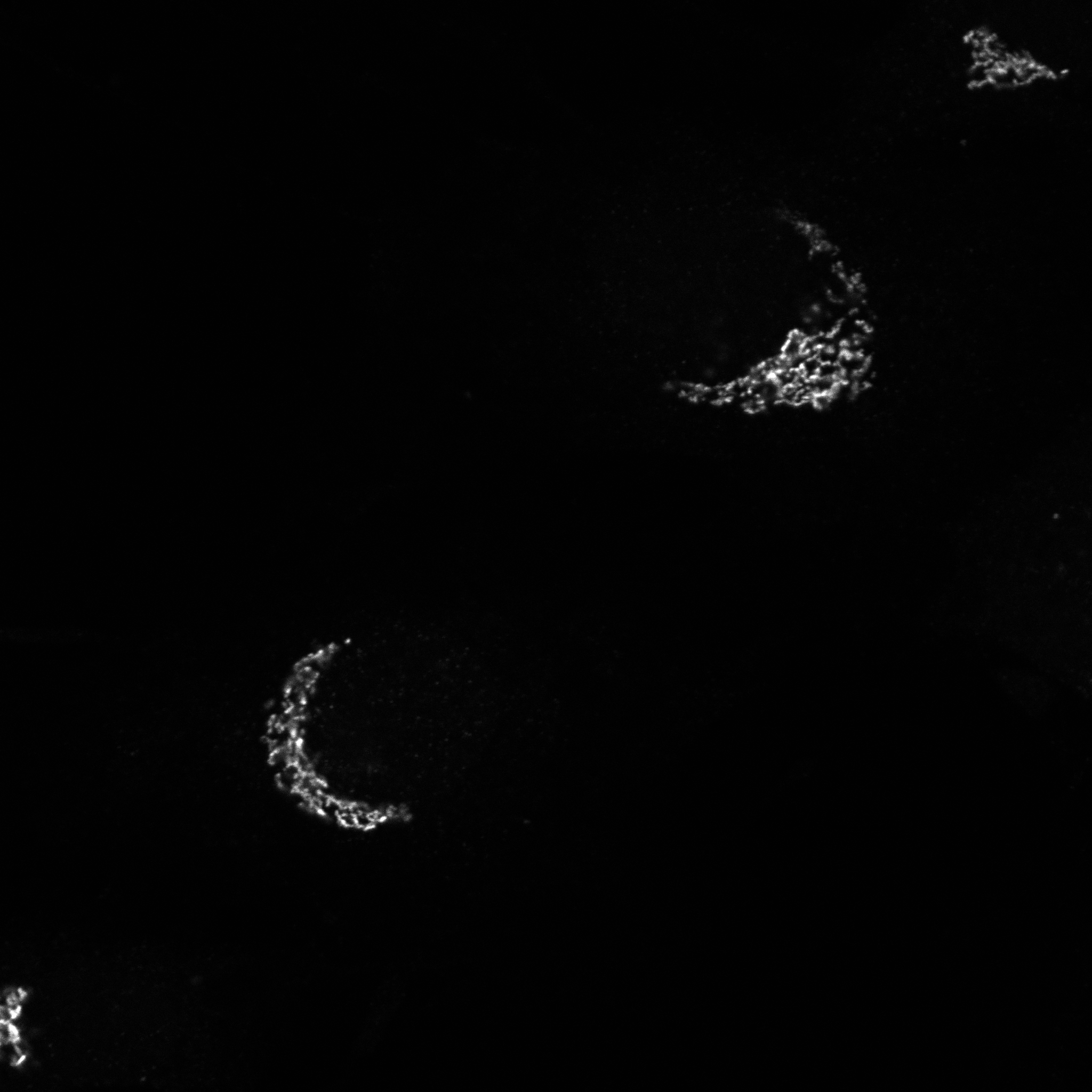

Supplement: Supplementary file 13 — EV and Appendix Figure Source Data [file 44318_2024_131_MOESM13_ESM.zip › ExpandedFigure 2/EV2D/FigureEV2D_GM130_Growing_GM130.tif]

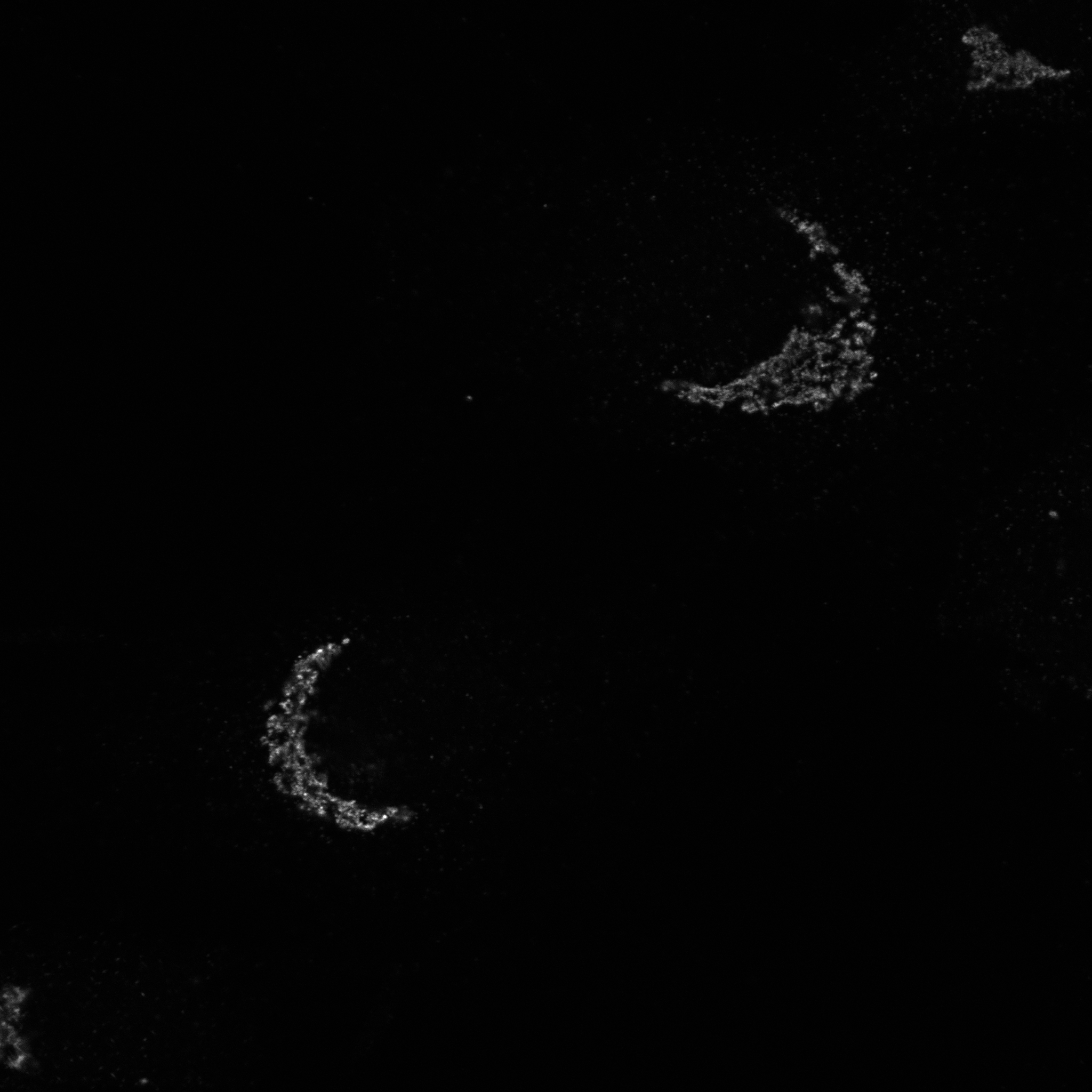

Supplement: Supplementary file 13 — EV and Appendix Figure Source Data [file 44318_2024_131_MOESM13_ESM.zip › ExpandedFigure 2/EV2D/FigureEV2D_GM130_Growing_YIPF3.tif]

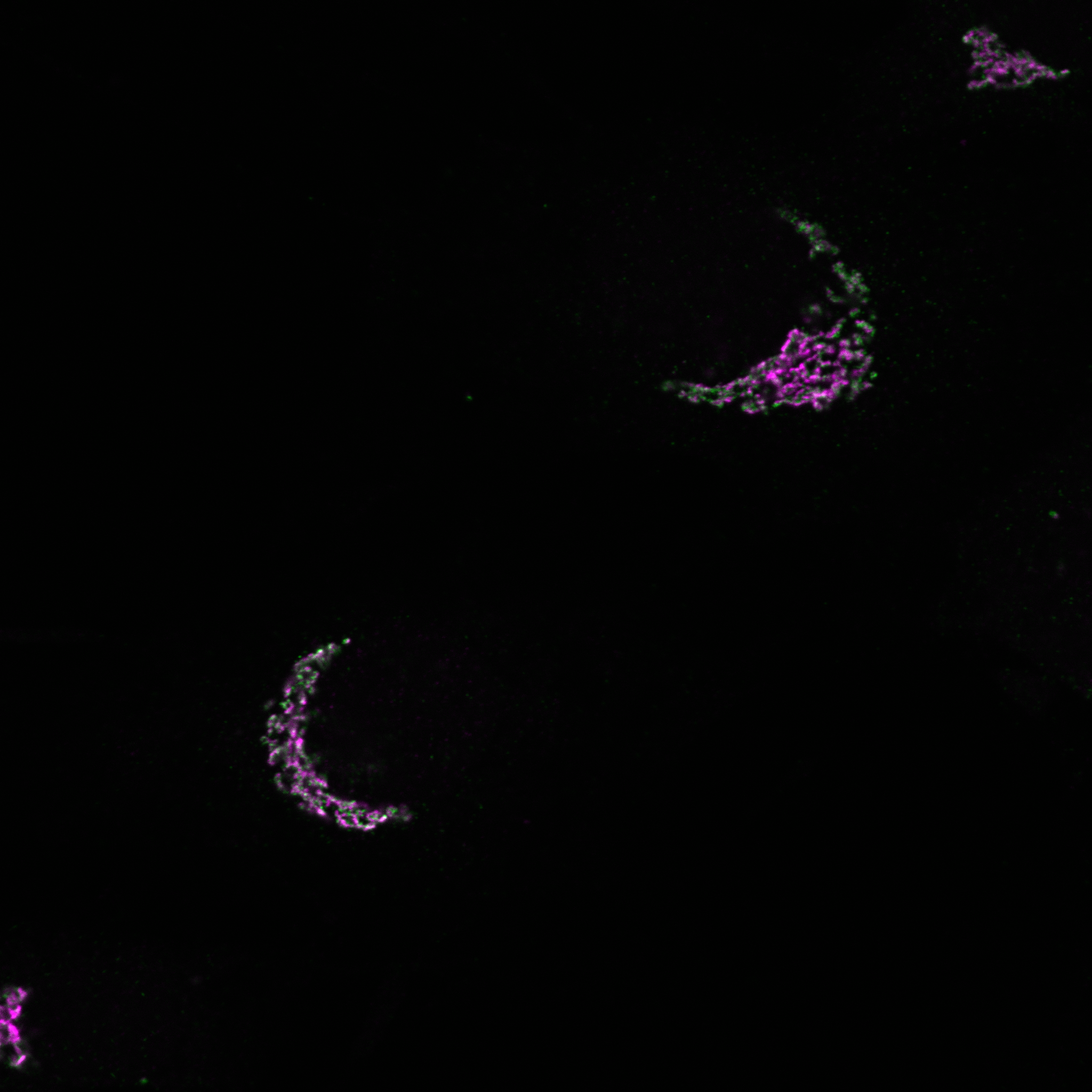

Supplement: Supplementary file 13 — EV and Appendix Figure Source Data [file 44318_2024_131_MOESM13_ESM.zip › ExpandedFigure 2/EV2D/FigureEV2D_GM130_Growing_merge.tif]

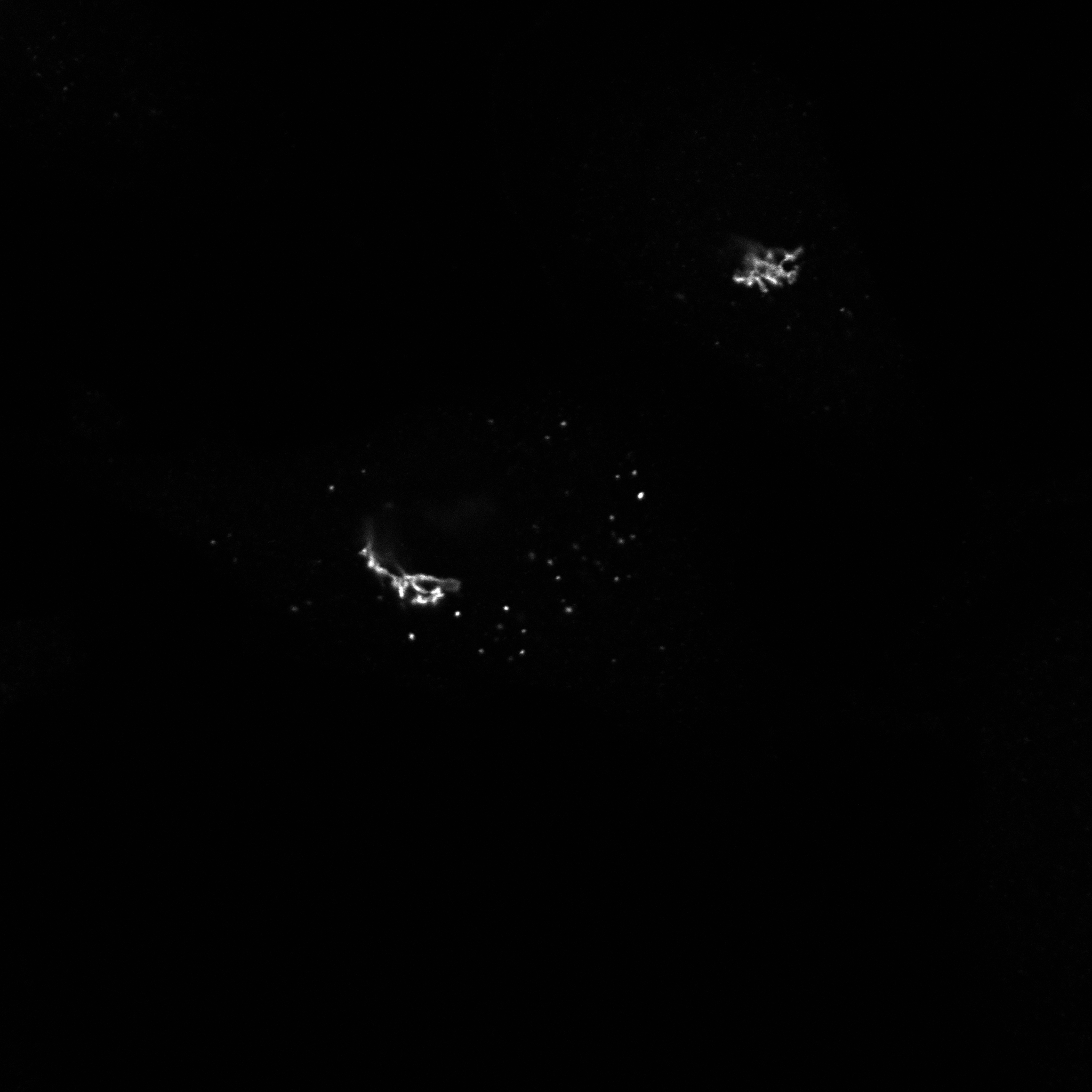

Supplement: Supplementary file 13 — EV and Appendix Figure Source Data [file 44318_2024_131_MOESM13_ESM.zip › ExpandedFigure 2/EV2D/FigureEV2D_GM130_StarvationBafA1_GM130.tif]

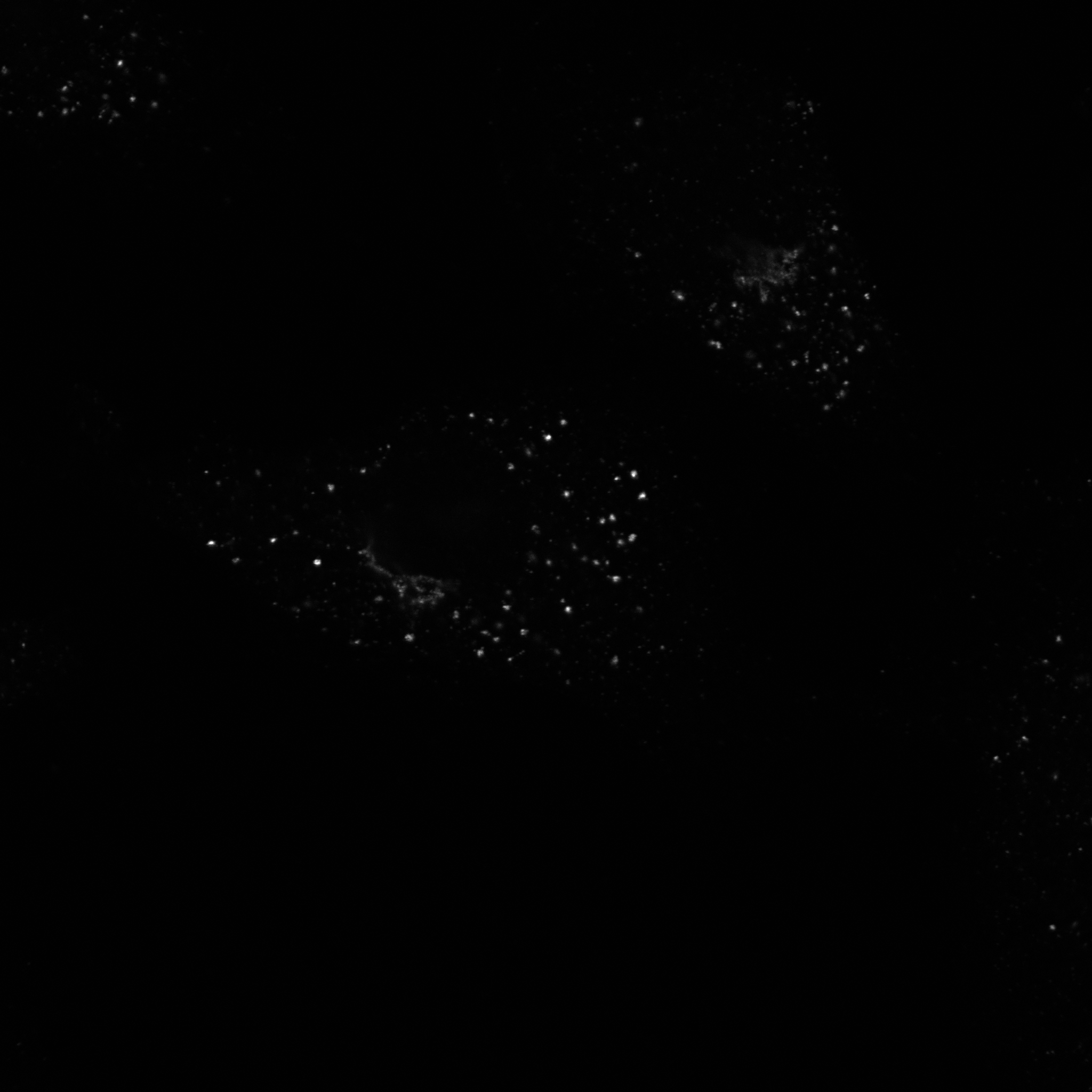

Supplement: Supplementary file 13 — EV and Appendix Figure Source Data [file 44318_2024_131_MOESM13_ESM.zip › ExpandedFigure 2/EV2D/FigureEV2D_GM130_StarvationBafA1_YIPF3.tif]

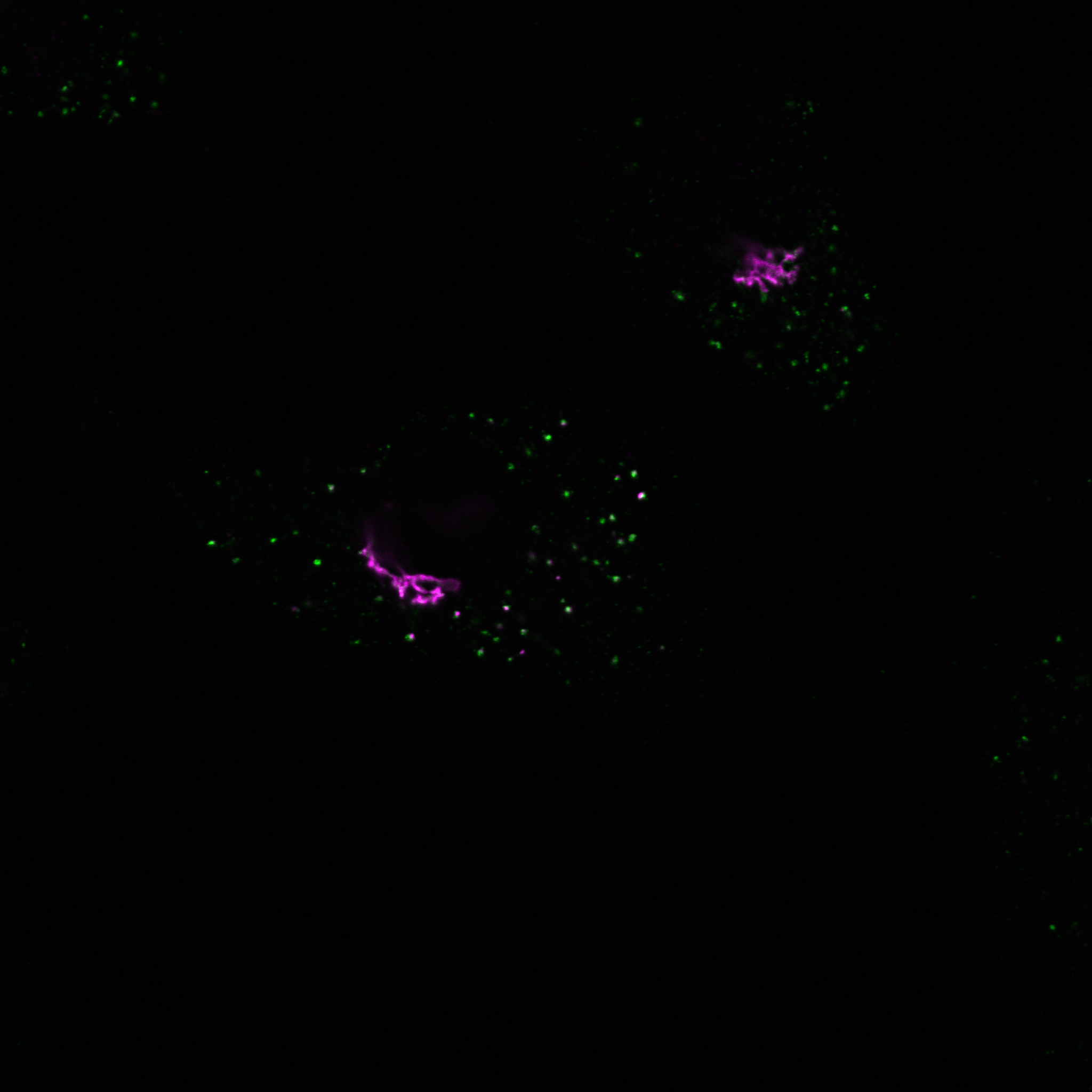

Supplement: Supplementary file 13 — EV and Appendix Figure Source Data [file 44318_2024_131_MOESM13_ESM.zip › ExpandedFigure 2/EV2D/FigureEV2D_GM130_StarvationBafA1_merge.tif]

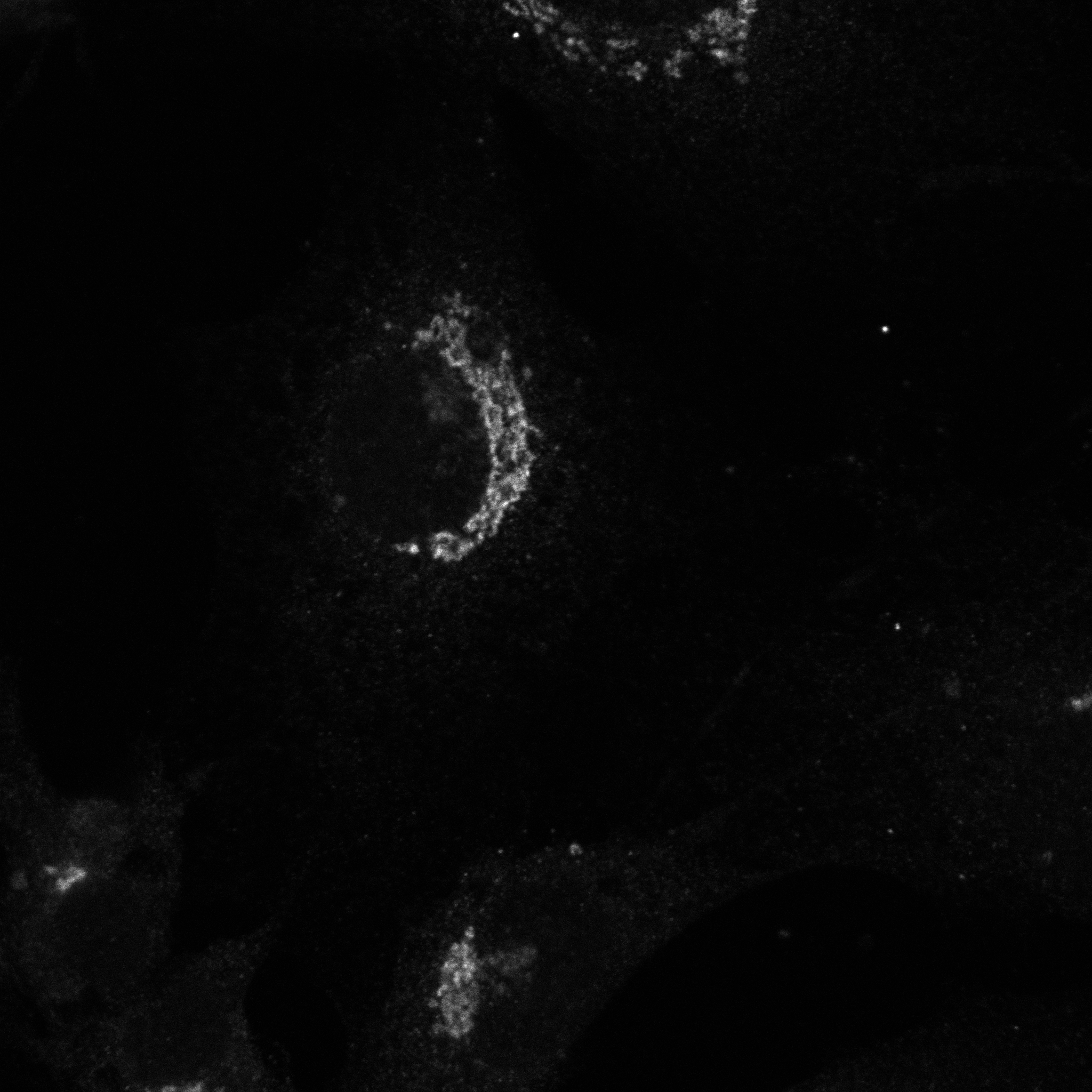

Supplement: Supplementary file 13 — EV and Appendix Figure Source Data [file 44318_2024_131_MOESM13_ESM.zip › ExpandedFigure 2/EV2D/FigureEV2D_GRASP55_Growing_GRASP55.tif]

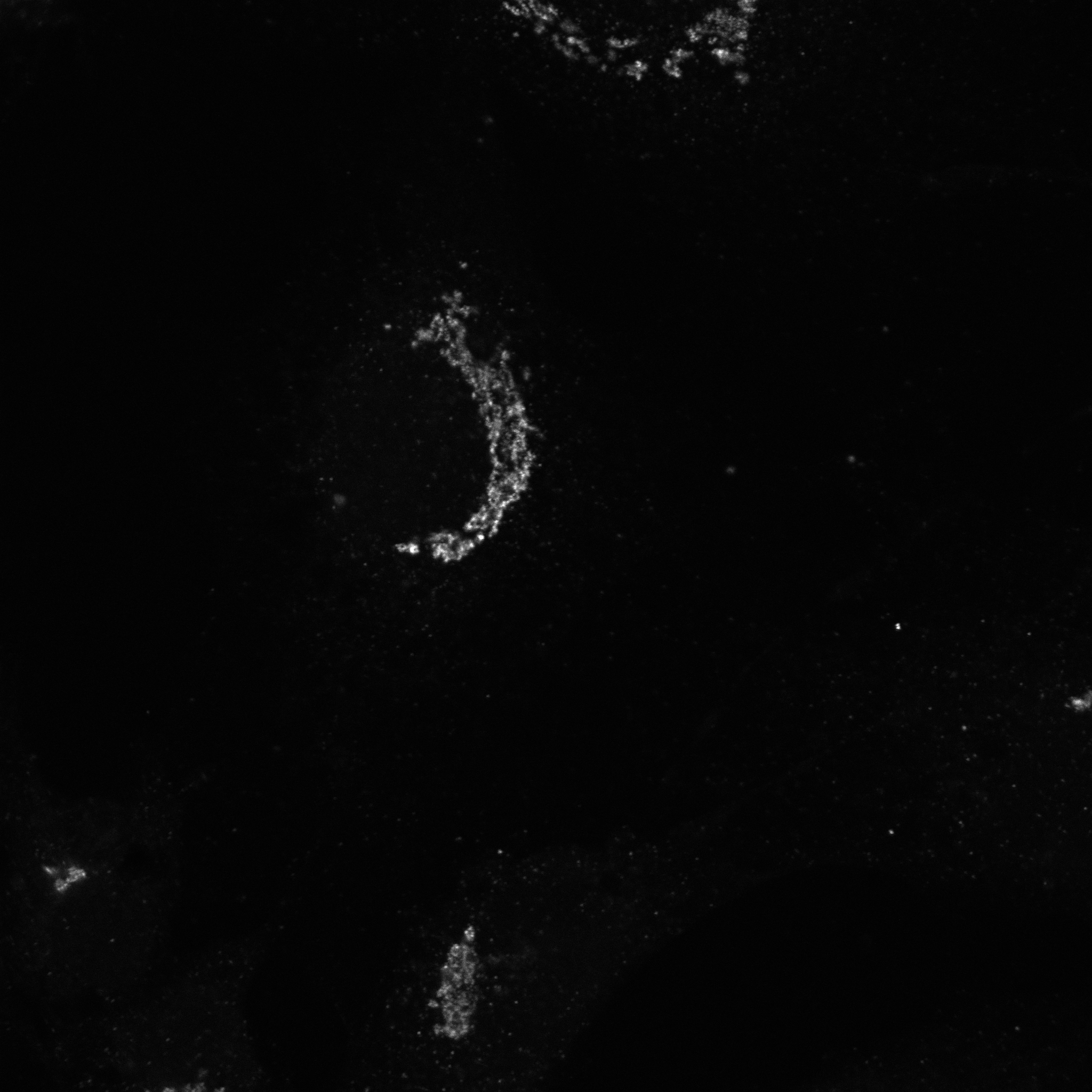

Supplement: Supplementary file 13 — EV and Appendix Figure Source Data [file 44318_2024_131_MOESM13_ESM.zip › ExpandedFigure 2/EV2D/FigureEV2D_GRASP55_Growing_YIPF3.tif]

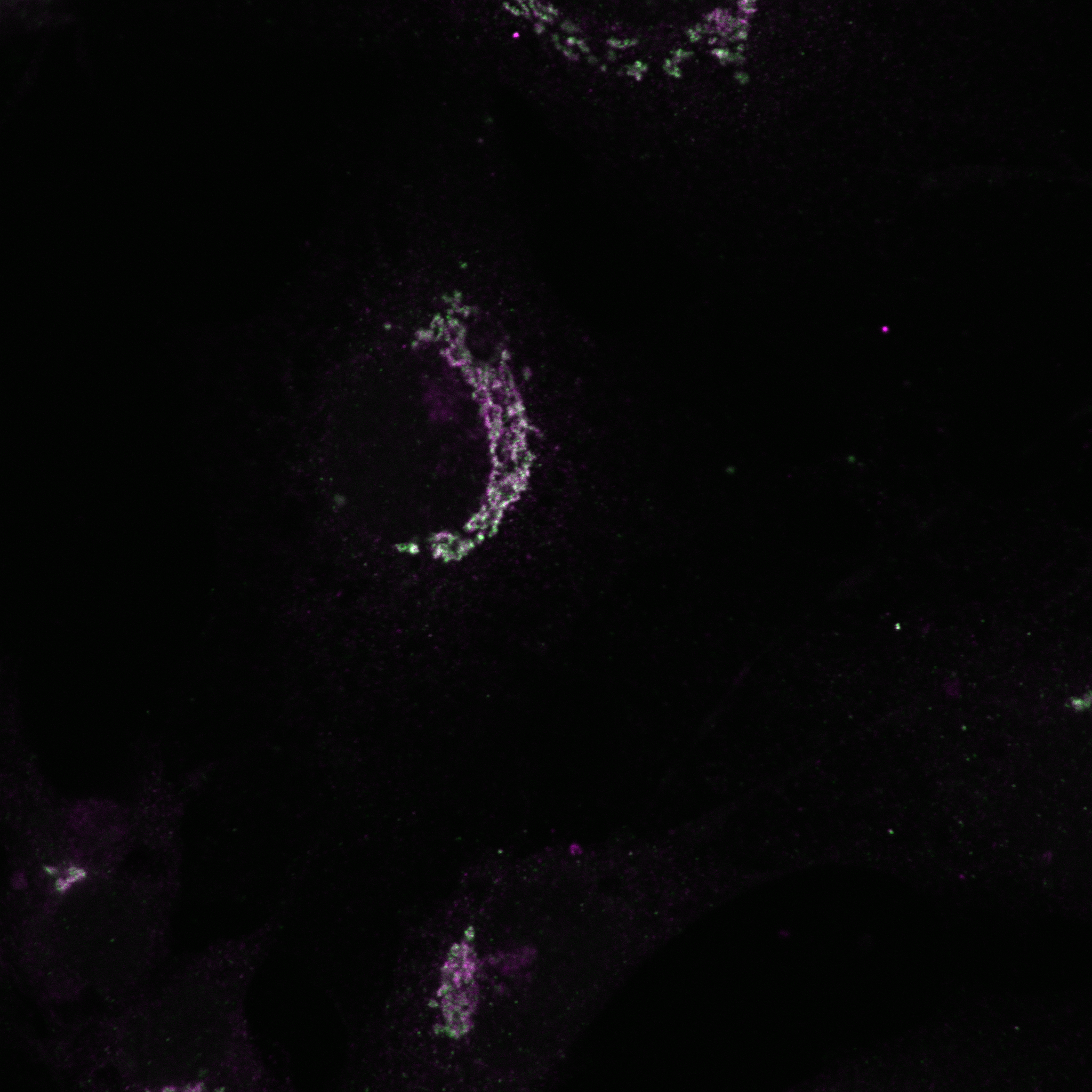

Supplement: Supplementary file 13 — EV and Appendix Figure Source Data [file 44318_2024_131_MOESM13_ESM.zip › ExpandedFigure 2/EV2D/FigureEV2D_GRASP55_Growing_merge.tif]

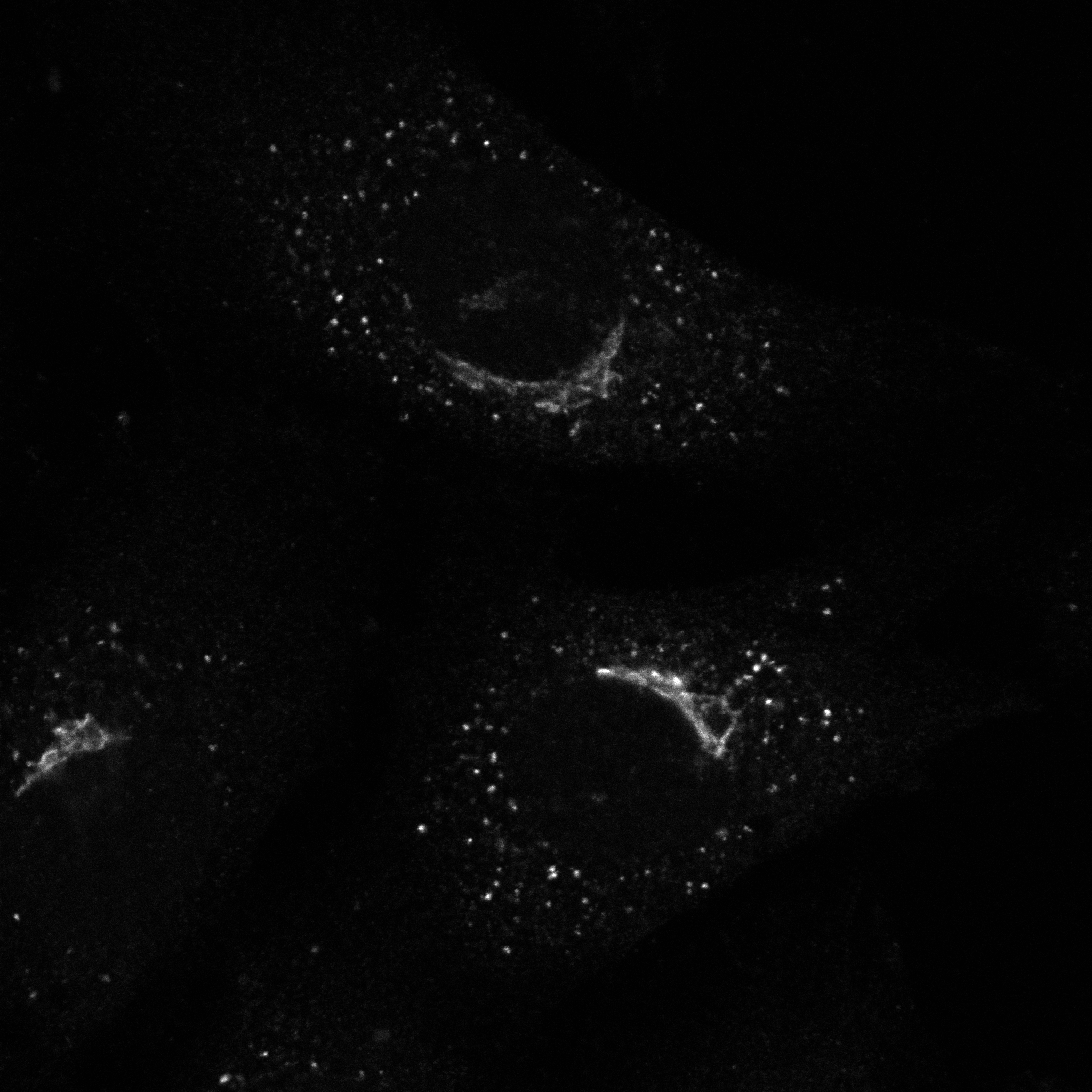

Supplement: Supplementary file 13 — EV and Appendix Figure Source Data [file 44318_2024_131_MOESM13_ESM.zip › ExpandedFigure 2/EV2D/FigureEV2D_GRASP55_StarvationBafA1_GRASP55.tif]

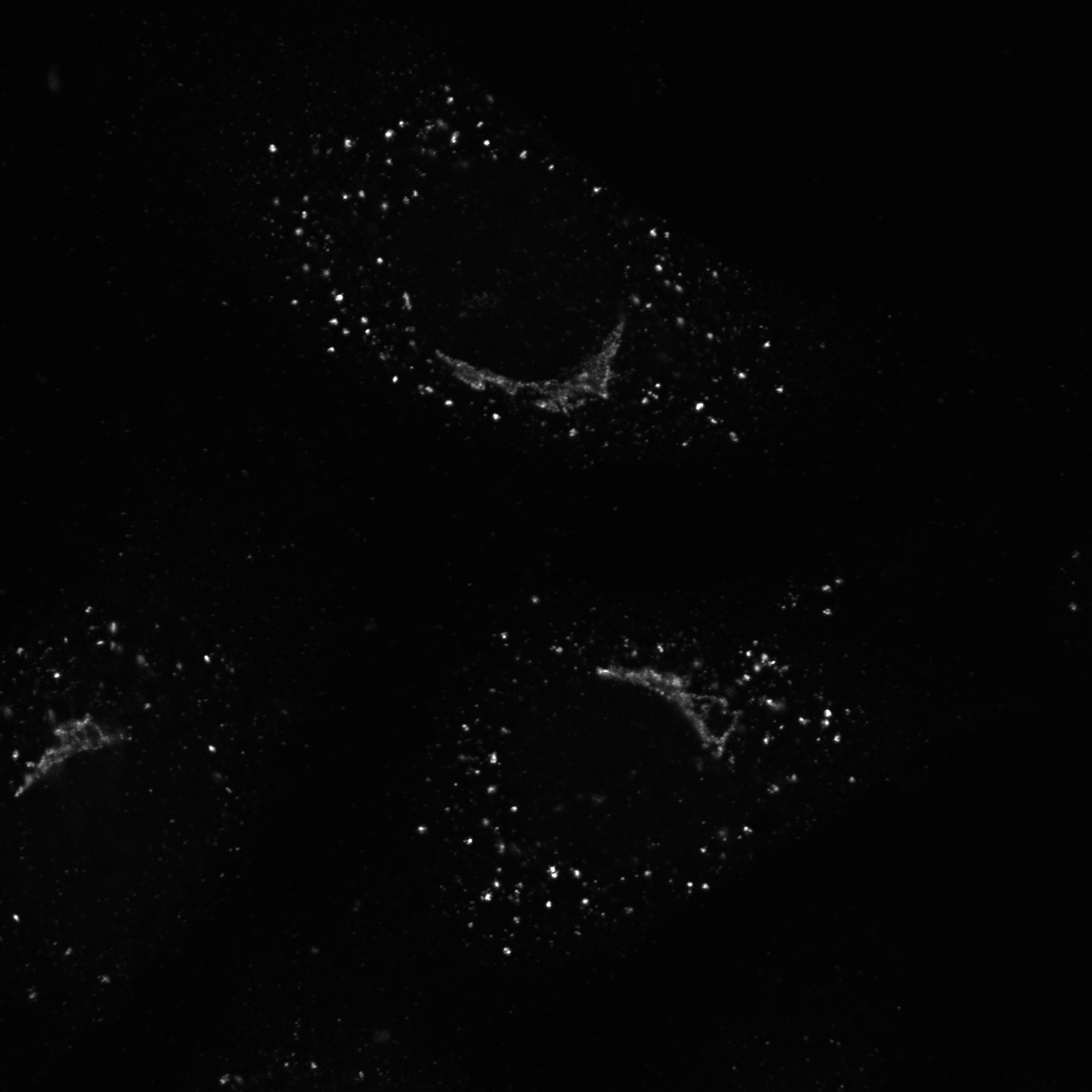

Supplement: Supplementary file 13 — EV and Appendix Figure Source Data [file 44318_2024_131_MOESM13_ESM.zip › ExpandedFigure 2/EV2D/FigureEV2D_GRASP55_StarvationBafA1_YIPF3.tif]

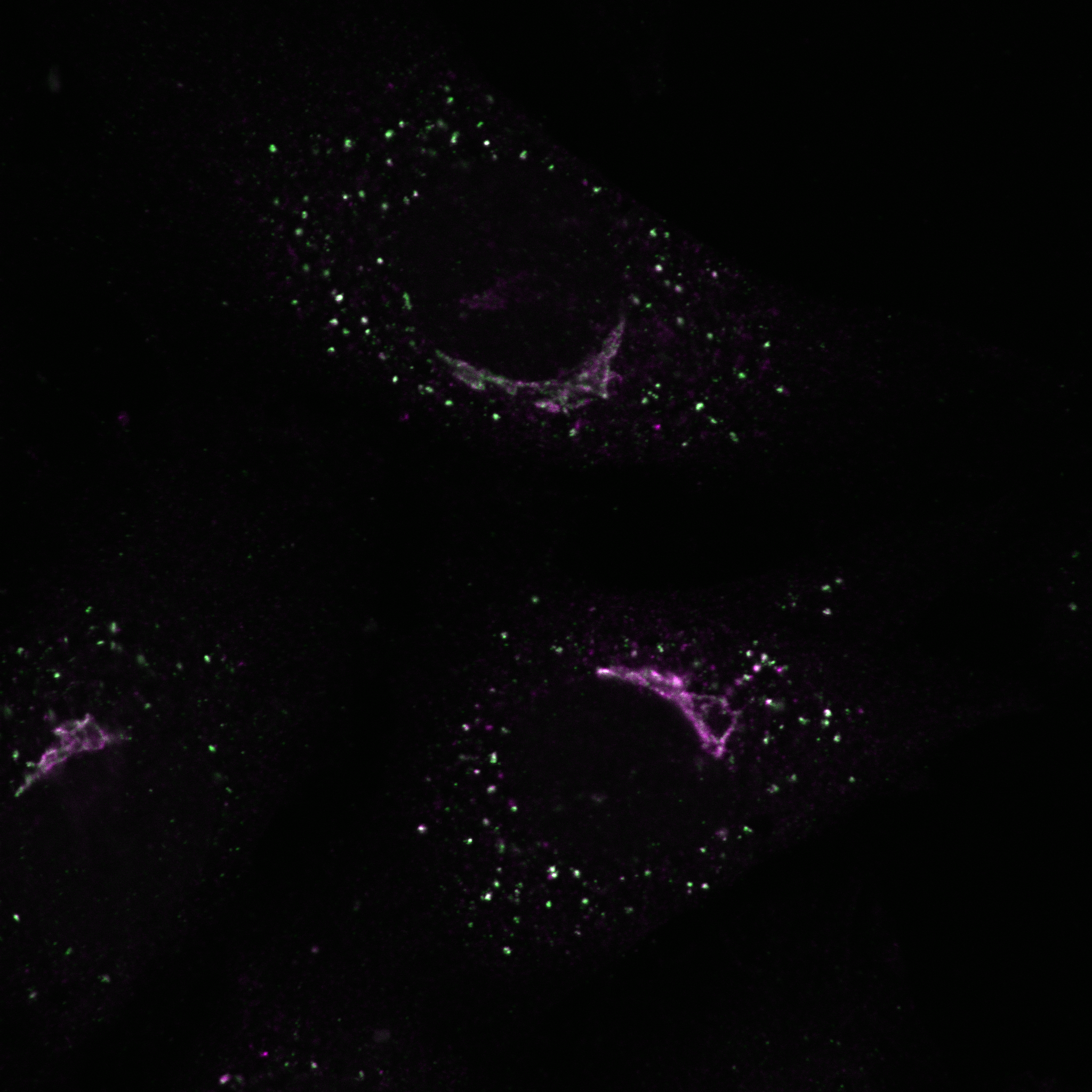

Supplement: Supplementary file 13 — EV and Appendix Figure Source Data [file 44318_2024_131_MOESM13_ESM.zip › ExpandedFigure 2/EV2D/FigureEV2D_GRASP55_StarvationBafA1_merge.tif]

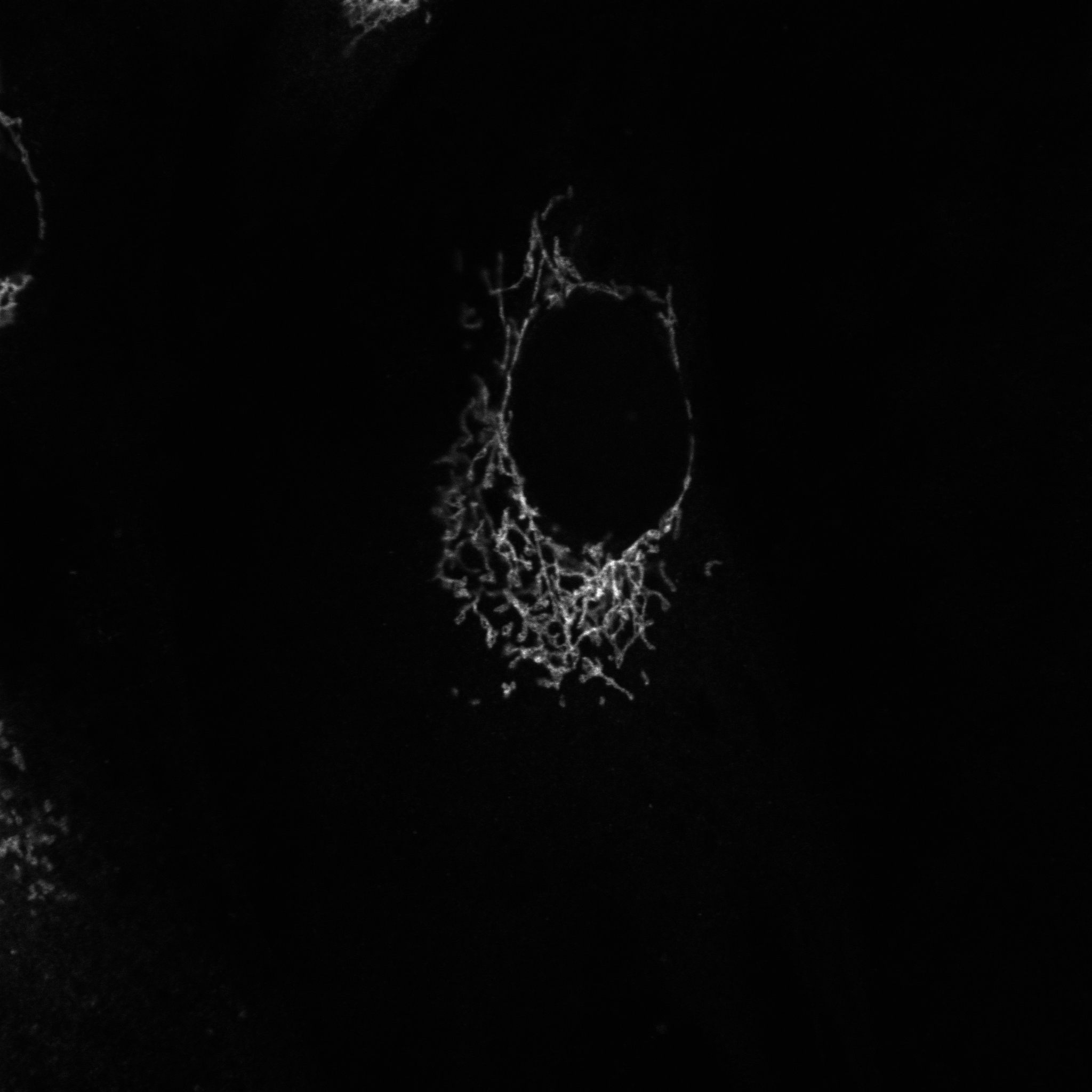

Supplement: Supplementary file 13 — EV and Appendix Figure Source Data [file 44318_2024_131_MOESM13_ESM.zip › ExpandedFigure 3/EV3A/FigureEV3A_EGFP-YIPF3LIR2A1_GM130_EGFP.tif]

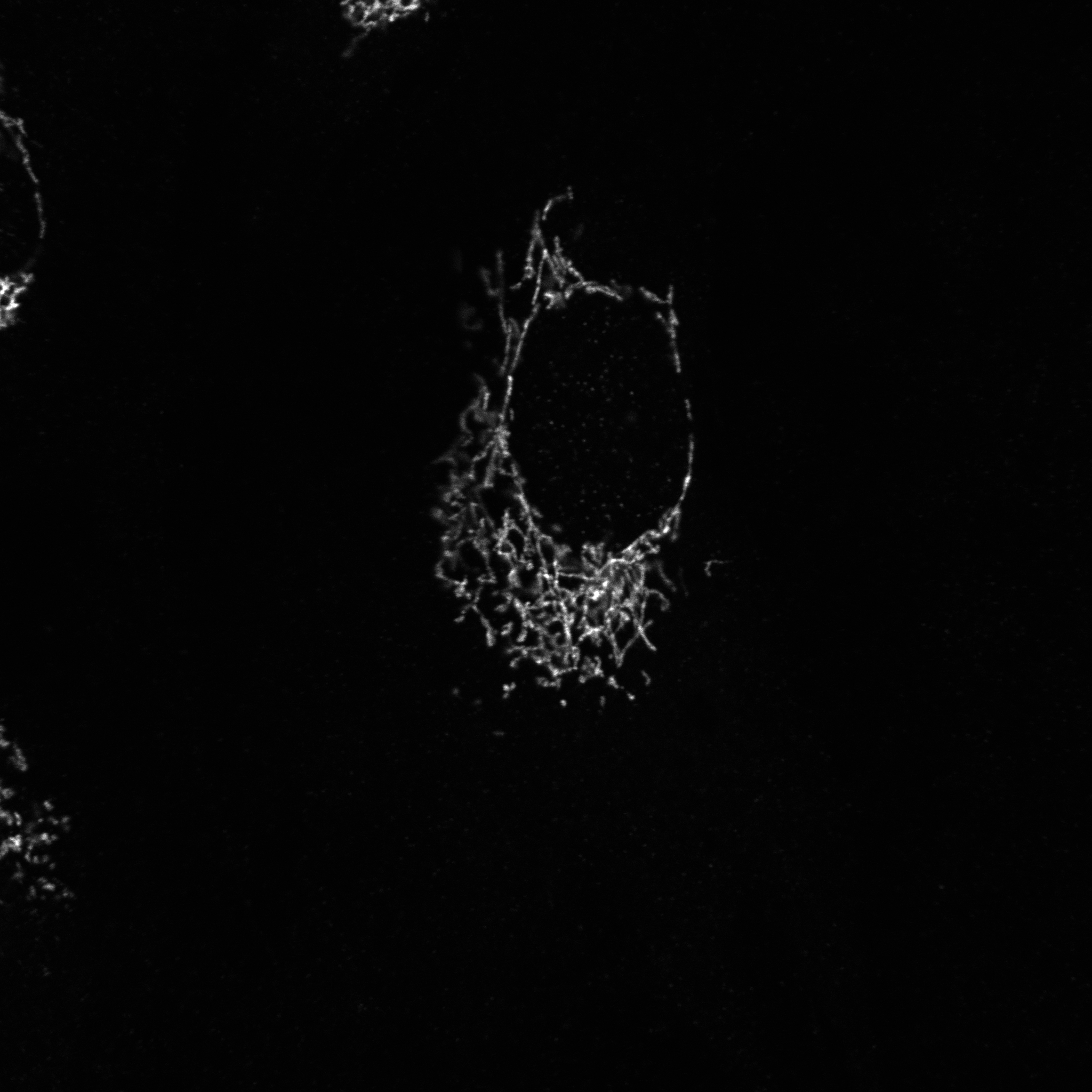

Supplement: Supplementary file 13 — EV and Appendix Figure Source Data [file 44318_2024_131_MOESM13_ESM.zip › ExpandedFigure 3/EV3A/FigureEV3A_EGFP-YIPF3LIR2A1_GM130_GM130.tif]

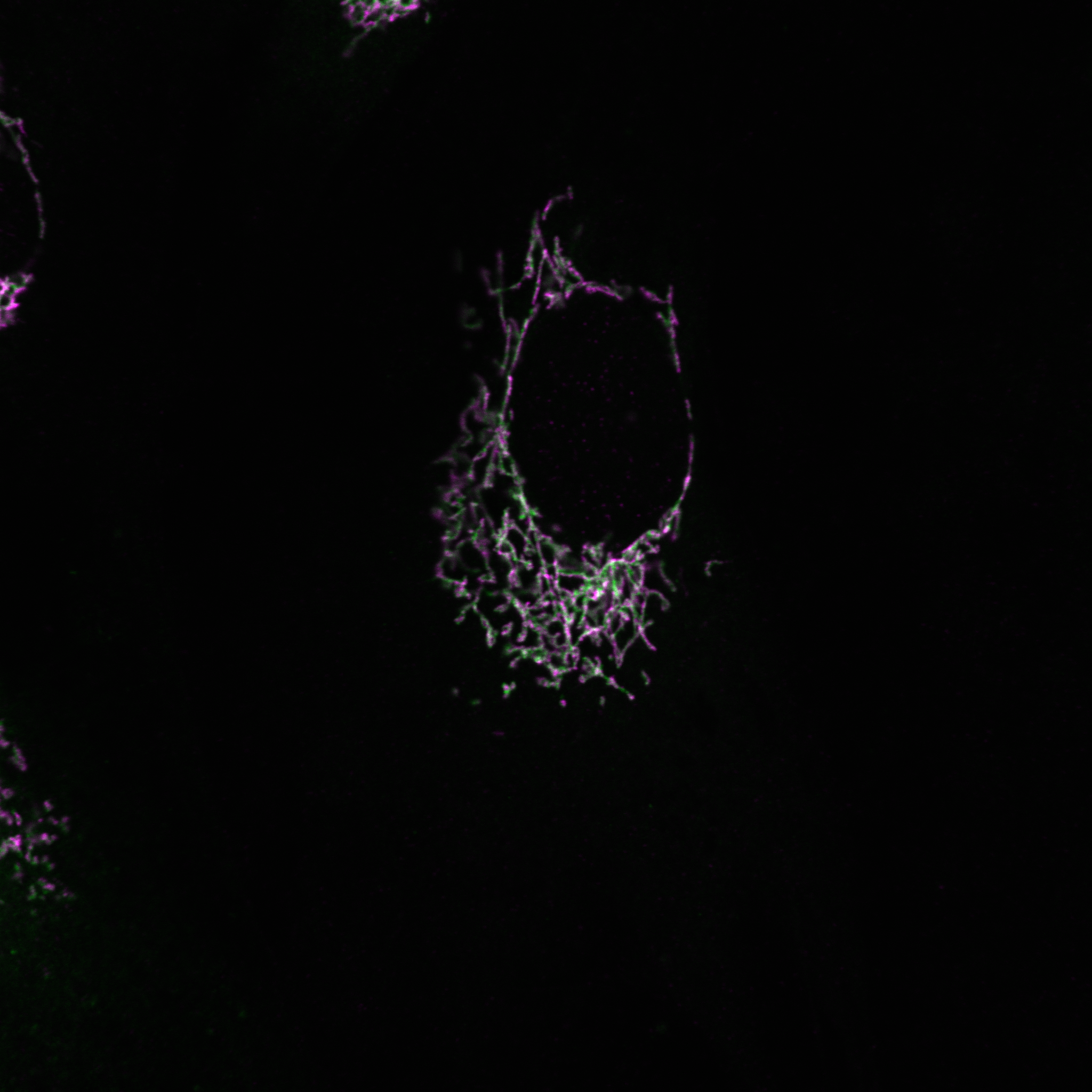

Supplement: Supplementary file 13 — EV and Appendix Figure Source Data [file 44318_2024_131_MOESM13_ESM.zip › ExpandedFigure 3/EV3A/FigureEV3A_EGFP-YIPF3LIR2A1_GM130_merge.tif]

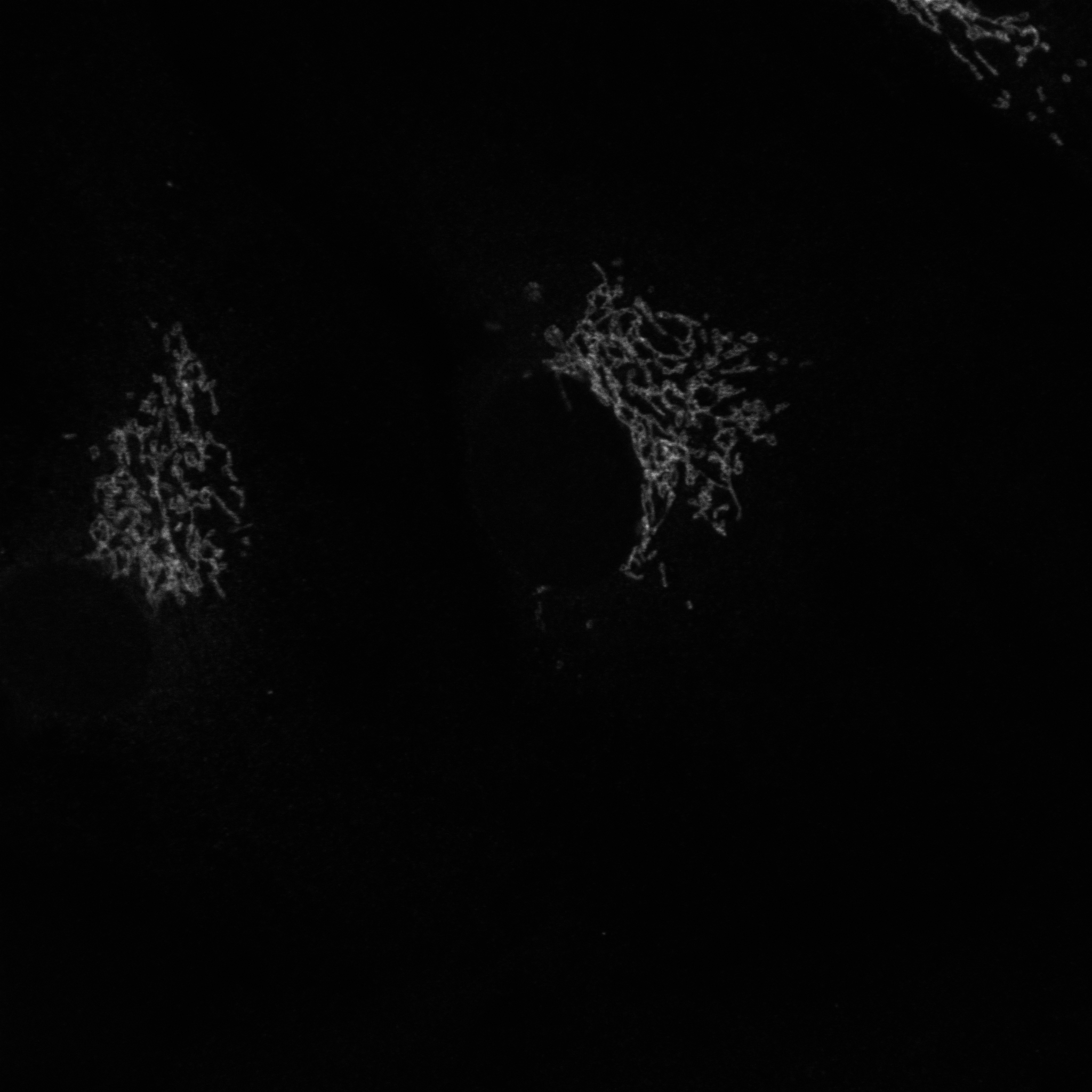

Supplement: Supplementary file 13 — EV and Appendix Figure Source Data [file 44318_2024_131_MOESM13_ESM.zip › ExpandedFigure 3/EV3A/FigureEV3A_EGFP-YIPF3LIR2A1_TMEM165_EGFP.tif]

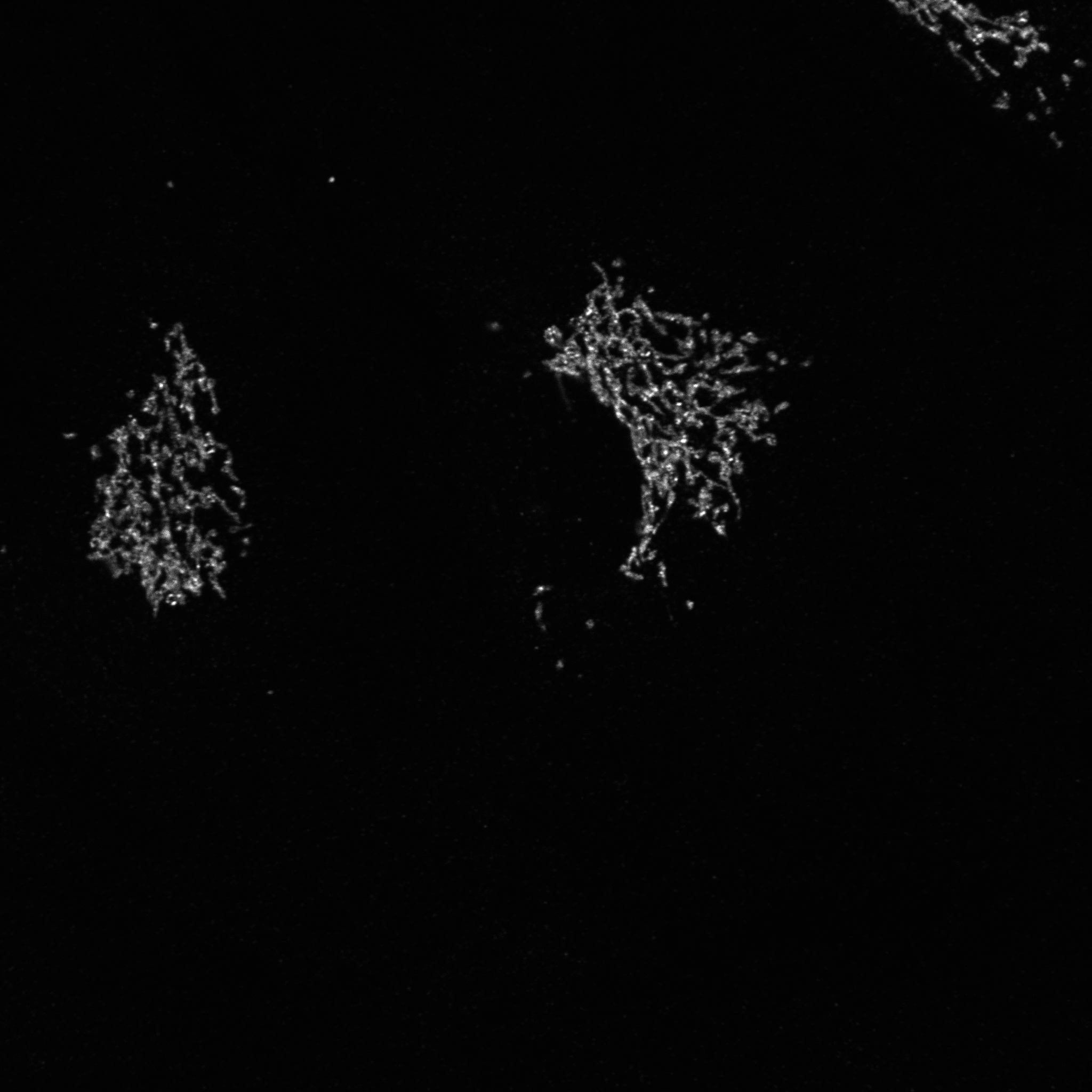

Supplement: Supplementary file 13 — EV and Appendix Figure Source Data [file 44318_2024_131_MOESM13_ESM.zip › ExpandedFigure 3/EV3A/FigureEV3A_EGFP-YIPF3LIR2A1_TMEM165_TMEM165.tif]

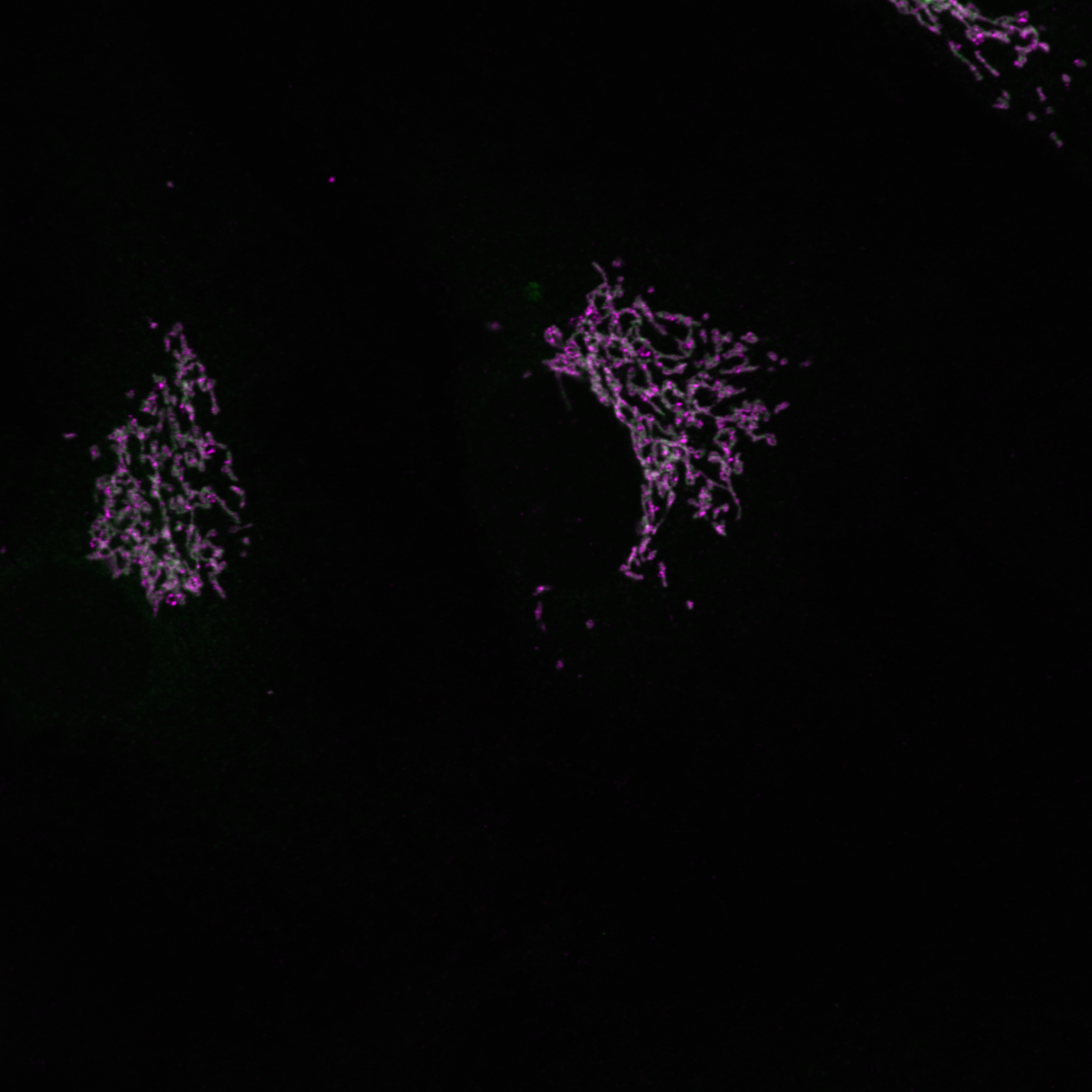

Supplement: Supplementary file 13 — EV and Appendix Figure Source Data [file 44318_2024_131_MOESM13_ESM.zip › ExpandedFigure 3/EV3A/FigureEV3A_EGFP-YIPF3LIR2A1_TMEM165_merge.tif]

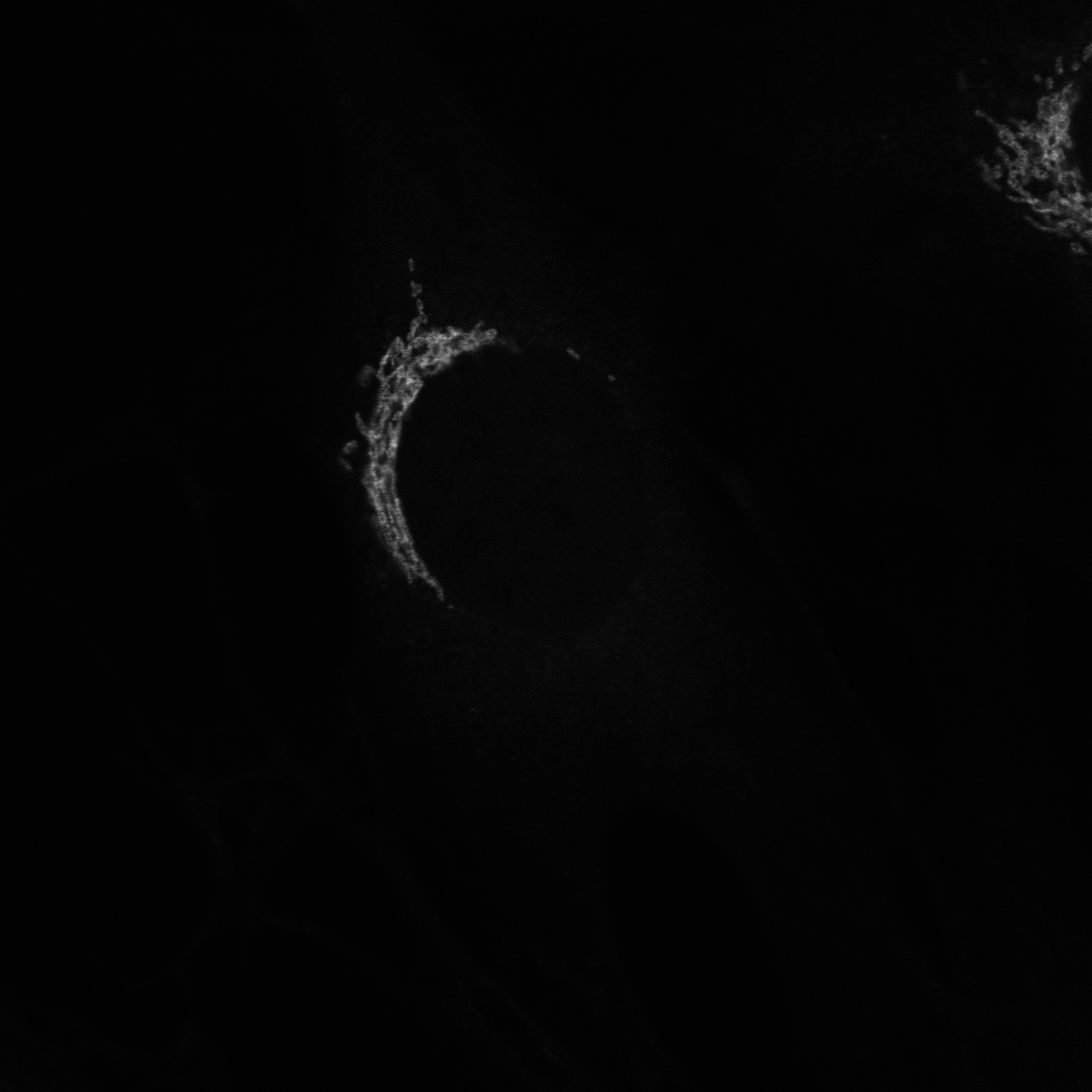

Supplement: Supplementary file 13 — EV and Appendix Figure Source Data [file 44318_2024_131_MOESM13_ESM.zip › ExpandedFigure 3/EV3A/FigureEV3A_EGFP-YIPF3WT_GM130_EGFP.tif]

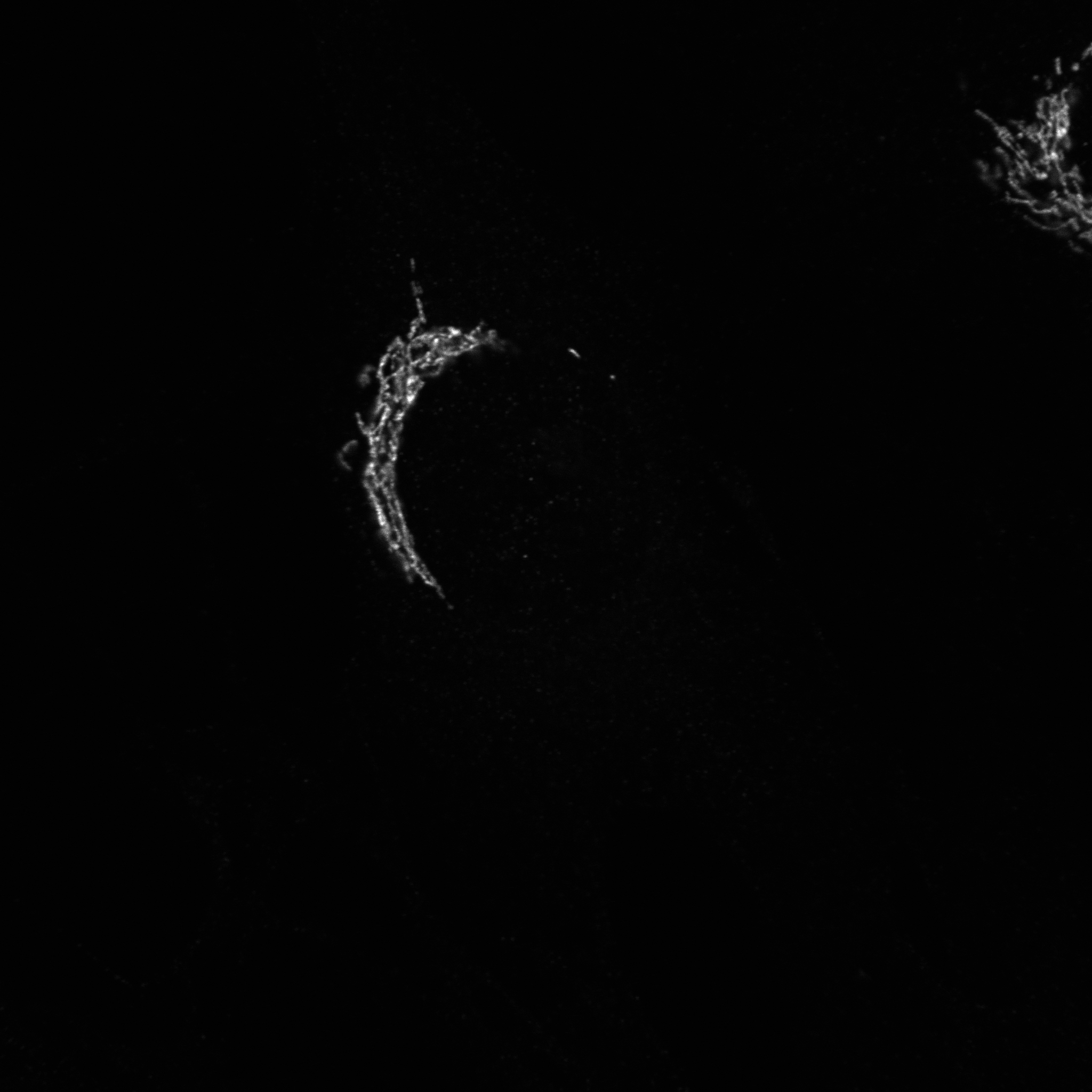

Supplement: Supplementary file 13 — EV and Appendix Figure Source Data [file 44318_2024_131_MOESM13_ESM.zip › ExpandedFigure 3/EV3A/FigureEV3A_EGFP-YIPF3WT_GM130_GM130.tif]

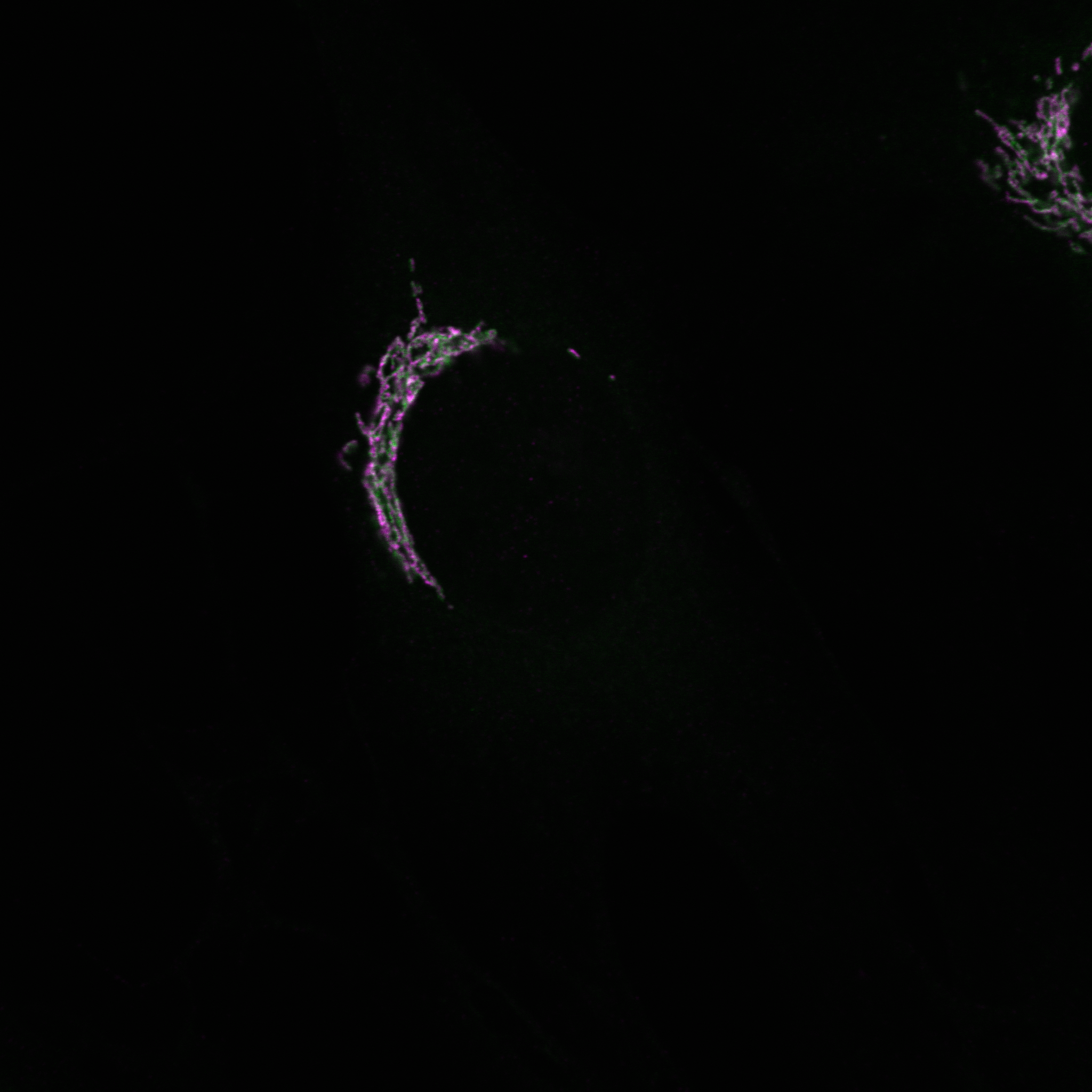

Supplement: Supplementary file 13 — EV and Appendix Figure Source Data [file 44318_2024_131_MOESM13_ESM.zip › ExpandedFigure 3/EV3A/FigureEV3A_EGFP-YIPF3WT_GM130_merge.tif]

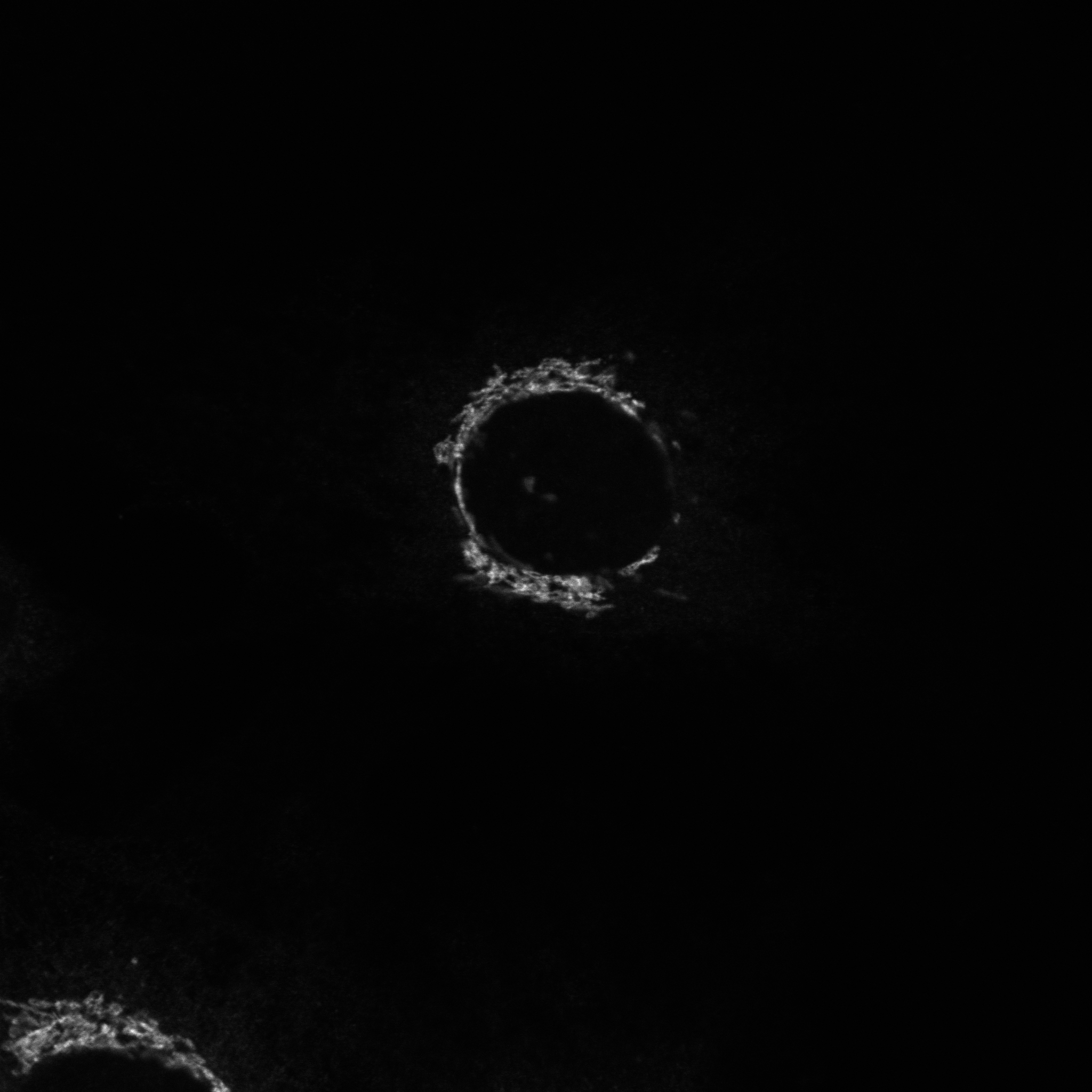

Supplement: Supplementary file 13 — EV and Appendix Figure Source Data [file 44318_2024_131_MOESM13_ESM.zip › ExpandedFigure 3/EV3A/FigureEV3A_EGFP-YIPF3WT_TMEM165_EGFP.tif]

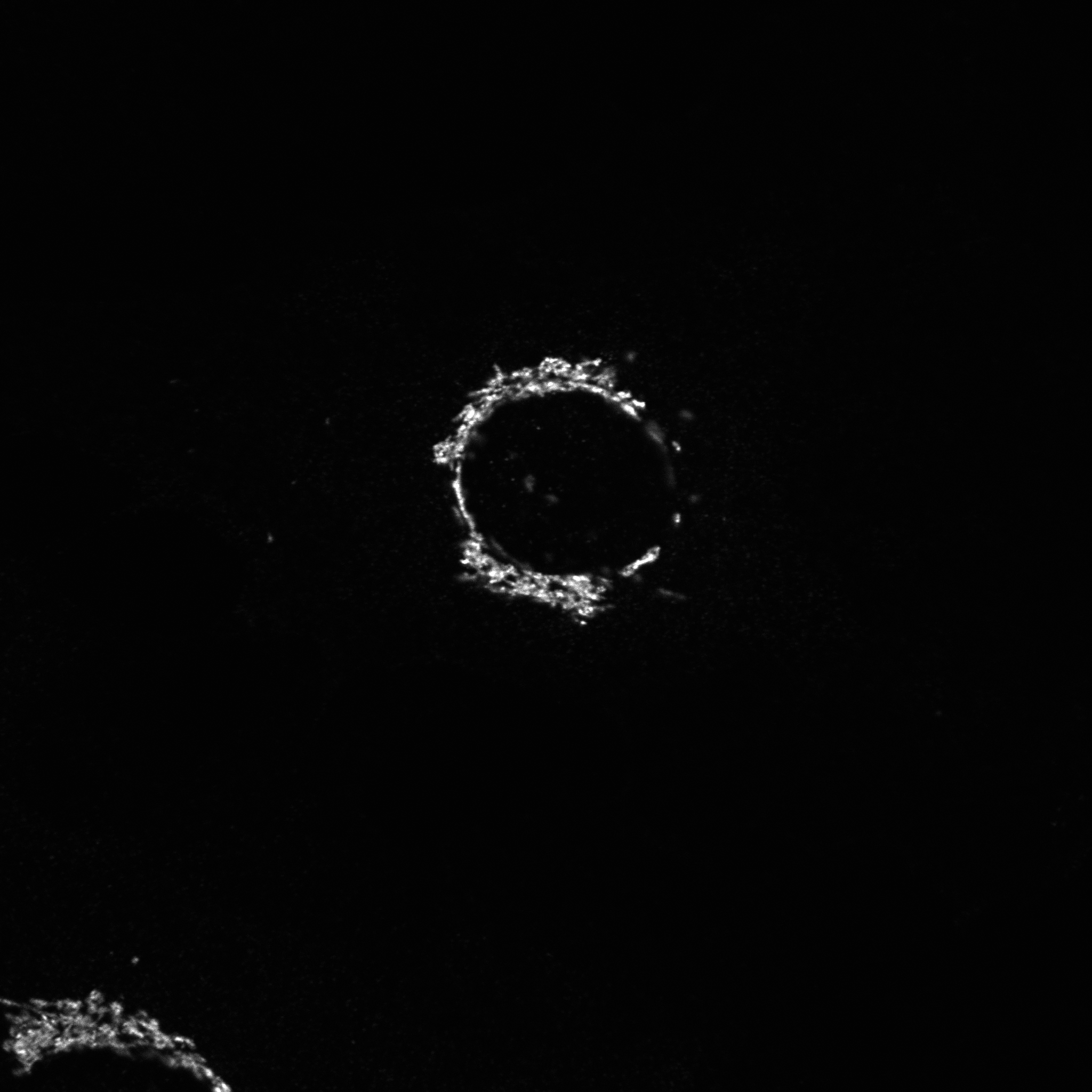

Supplement: Supplementary file 13 — EV and Appendix Figure Source Data [file 44318_2024_131_MOESM13_ESM.zip › ExpandedFigure 3/EV3A/FigureEV3A_EGFP-YIPF3WT_TMEM165_TMEM165.tif]
