# Supplementary figures and images for: YIPF3 and YIPF4 regulate autophagic turnover of the Golgi apparatus (part 2 of 2)
Source: EMBO J. 2024 May 31;43(14):8. doi: 10.1038/s44318-024-00131-3 (PMC11250848; doi:10.1038/s44318-024-00131-3)

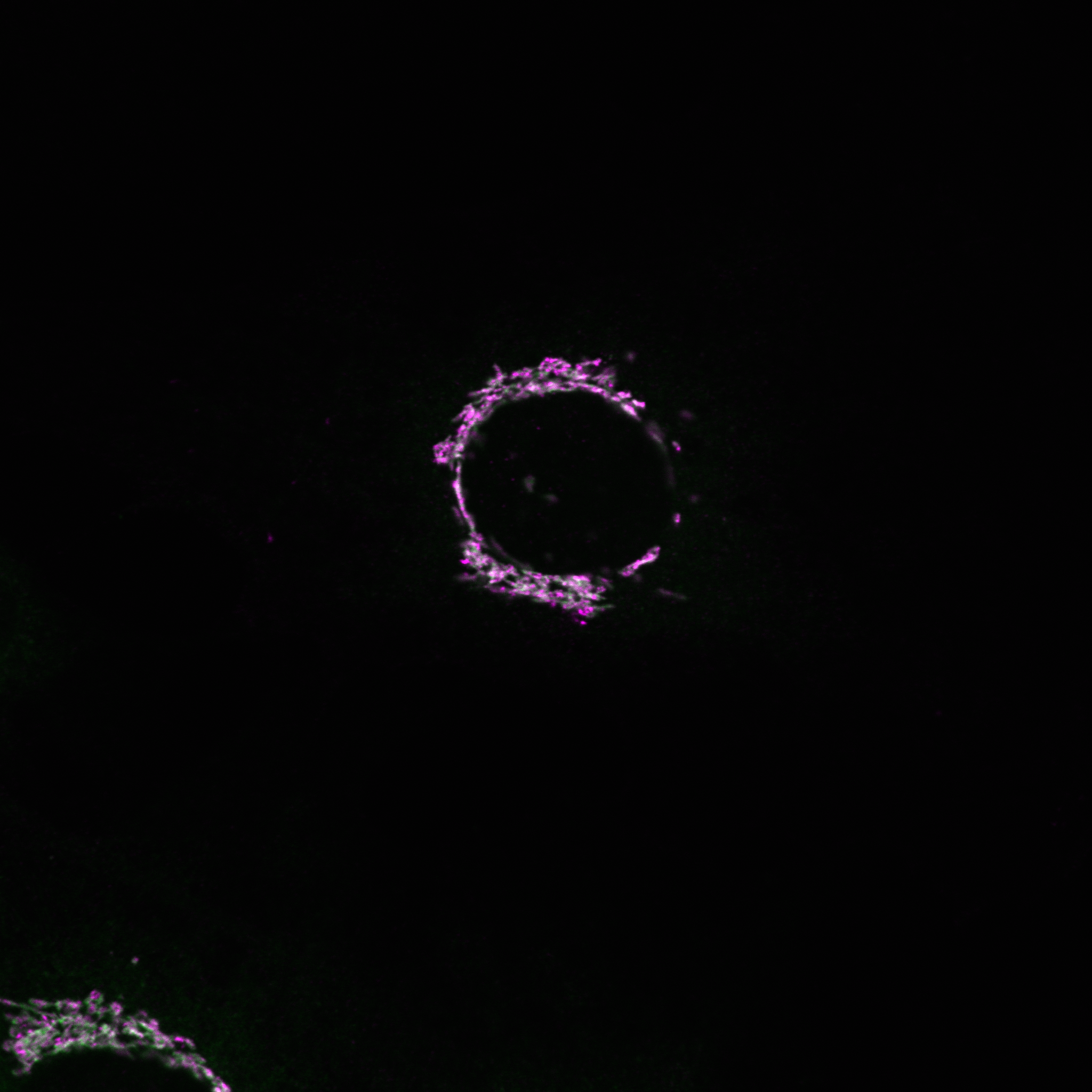

Supplement: Supplementary file 13 — EV and Appendix Figure Source Data [file 44318_2024_131_MOESM13_ESM.zip › ExpandedFigure 3/EV3A/FigureEV3A_EGFP-YIPF3WT_TMEM165_merge.tif]

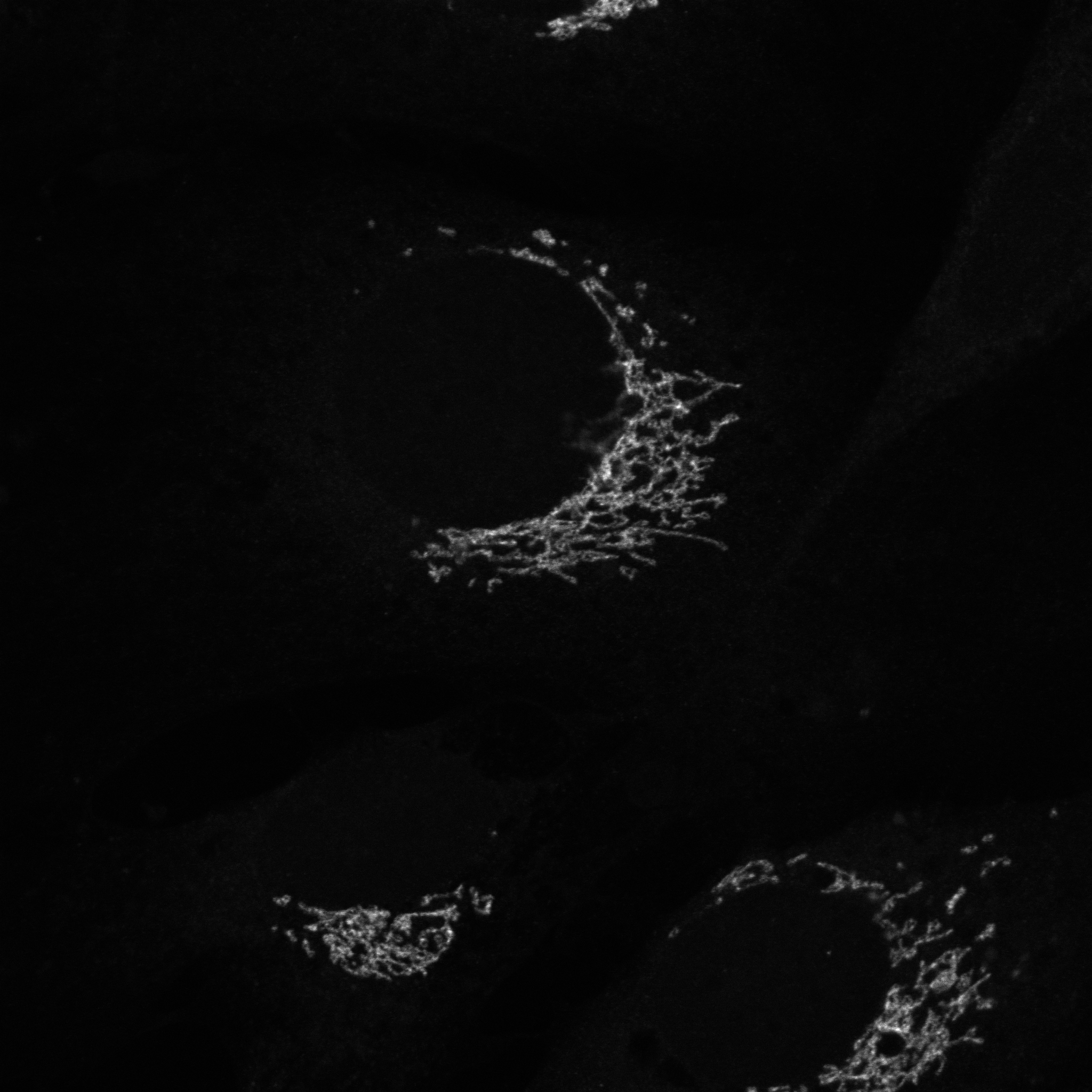

Supplement: Supplementary file 13 — EV and Appendix Figure Source Data [file 44318_2024_131_MOESM13_ESM.zip › ExpandedFigure 3/EV3B/FigureEV3B_EGFP-YIPF3LIR2A1_EGFP.tif]

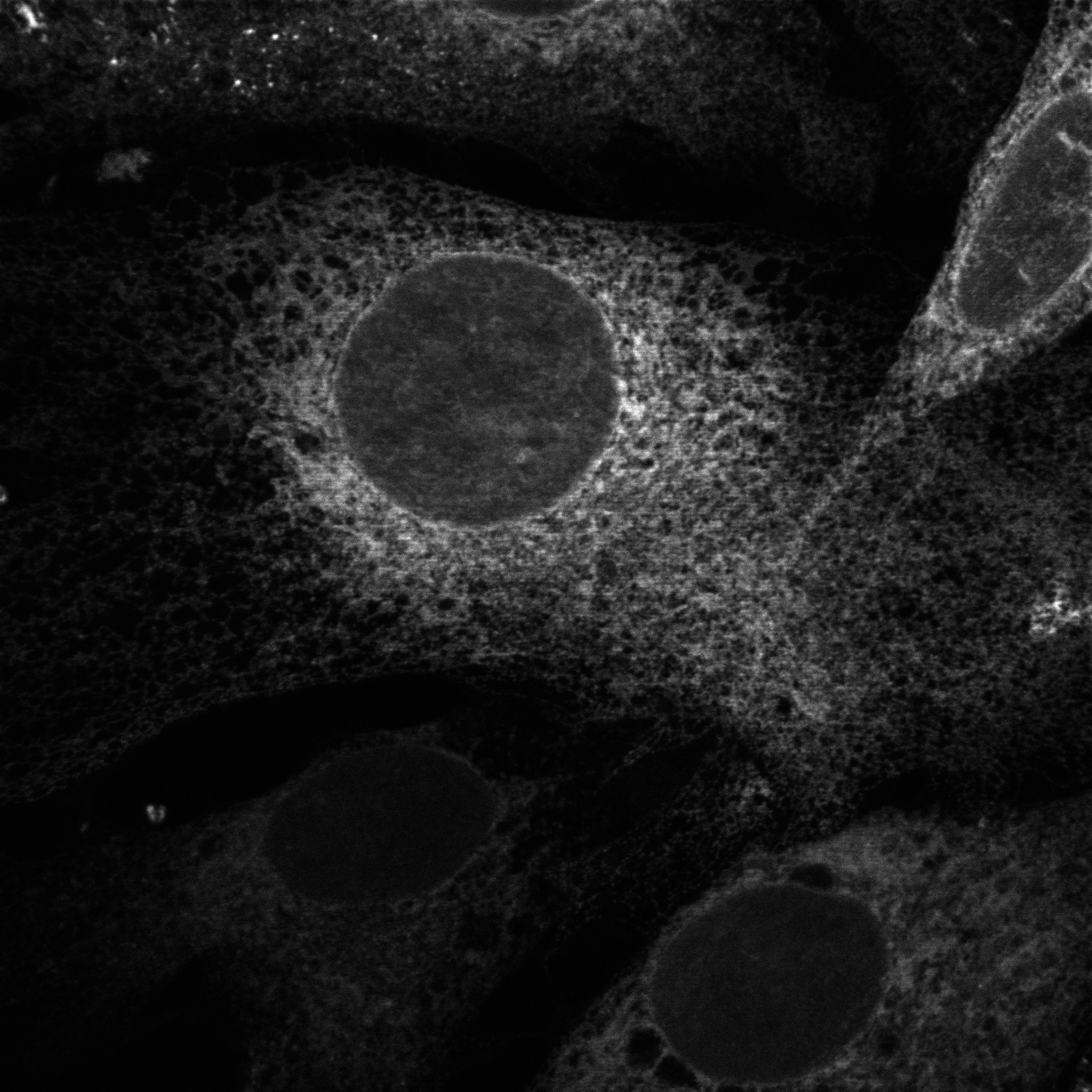

Supplement: Supplementary file 13 — EV and Appendix Figure Source Data [file 44318_2024_131_MOESM13_ESM.zip › ExpandedFigure 3/EV3B/FigureEV3B_EGFP-YIPF3LIR2A1_mCherry.tif]

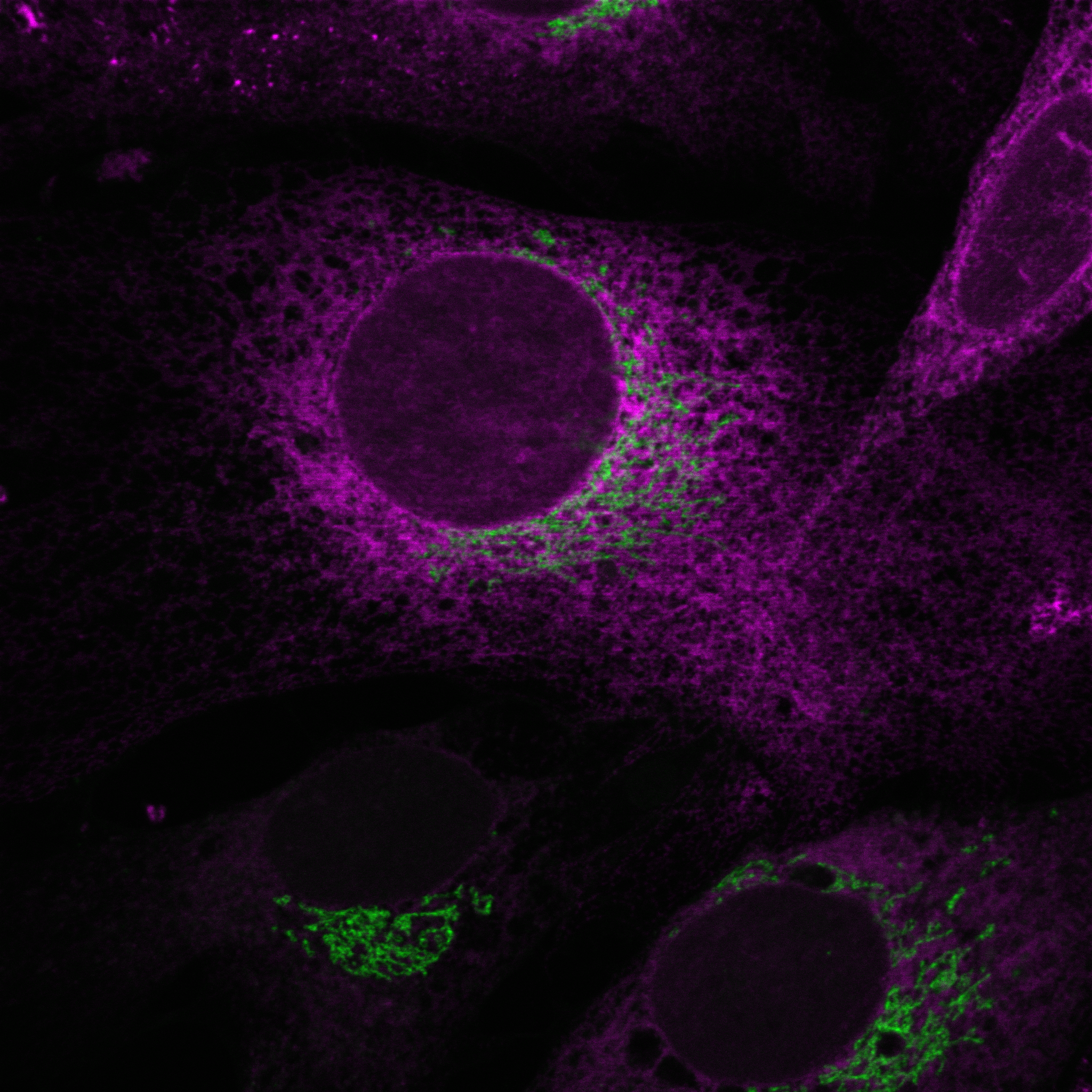

Supplement: Supplementary file 13 — EV and Appendix Figure Source Data [file 44318_2024_131_MOESM13_ESM.zip › ExpandedFigure 3/EV3B/FigureEV3B_EGFP-YIPF3LIR2A1_merge.tif]

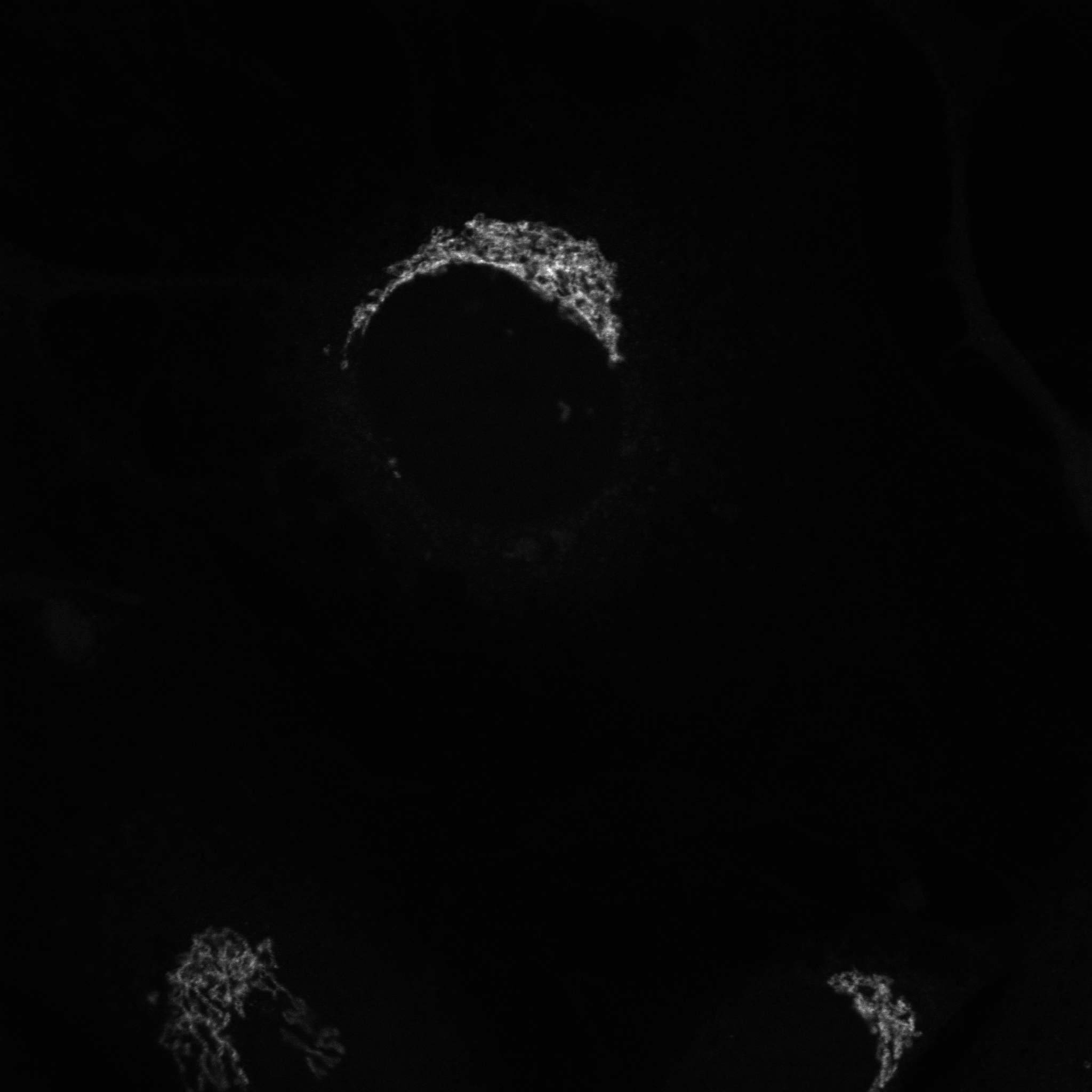

Supplement: Supplementary file 13 — EV and Appendix Figure Source Data [file 44318_2024_131_MOESM13_ESM.zip › ExpandedFigure 3/EV3B/FigureEV3B_EGFP-YIPF3WT_EGFP.tif]

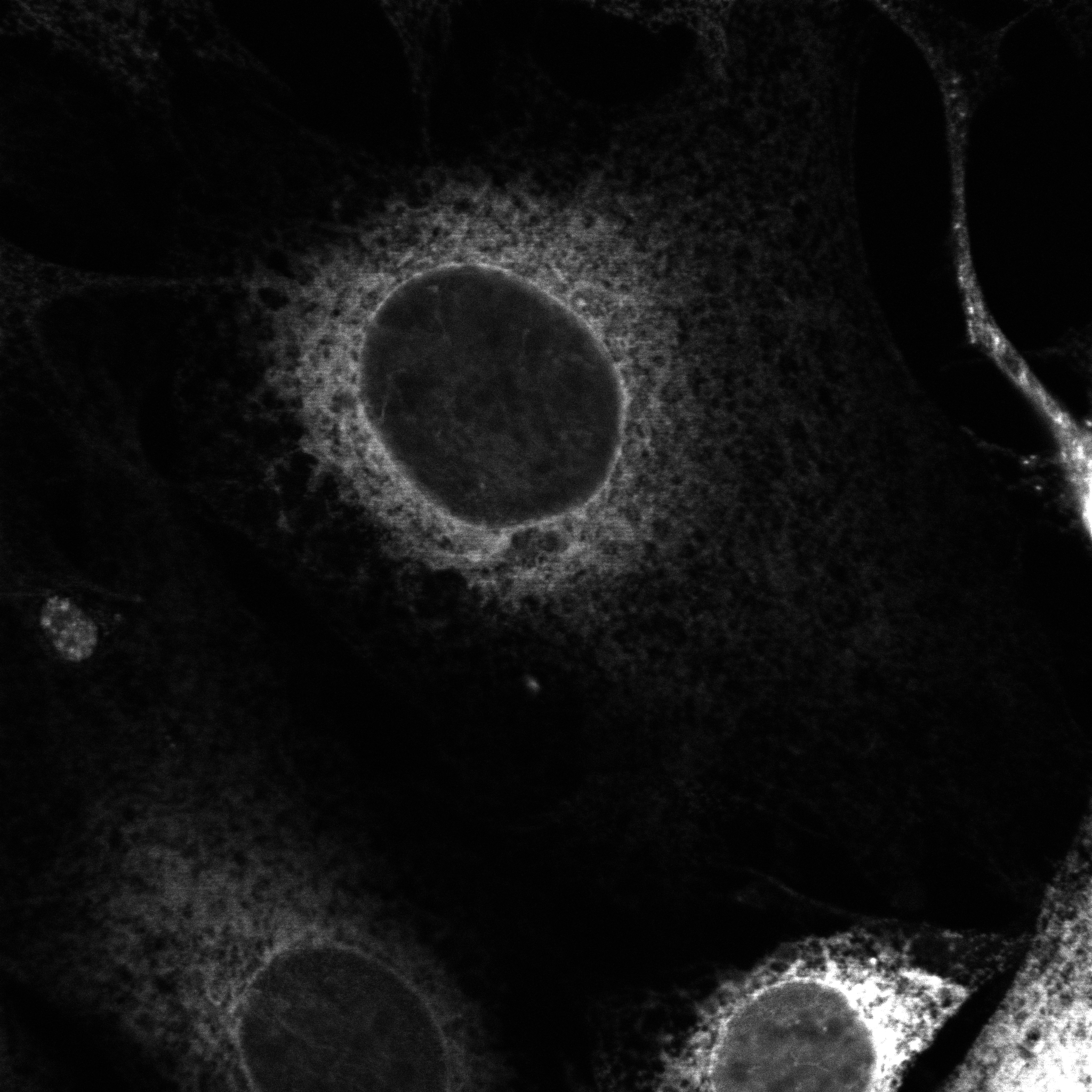

Supplement: Supplementary file 13 — EV and Appendix Figure Source Data [file 44318_2024_131_MOESM13_ESM.zip › ExpandedFigure 3/EV3B/FigureEV3B_EGFP-YIPF3WT_mCherry.tif]

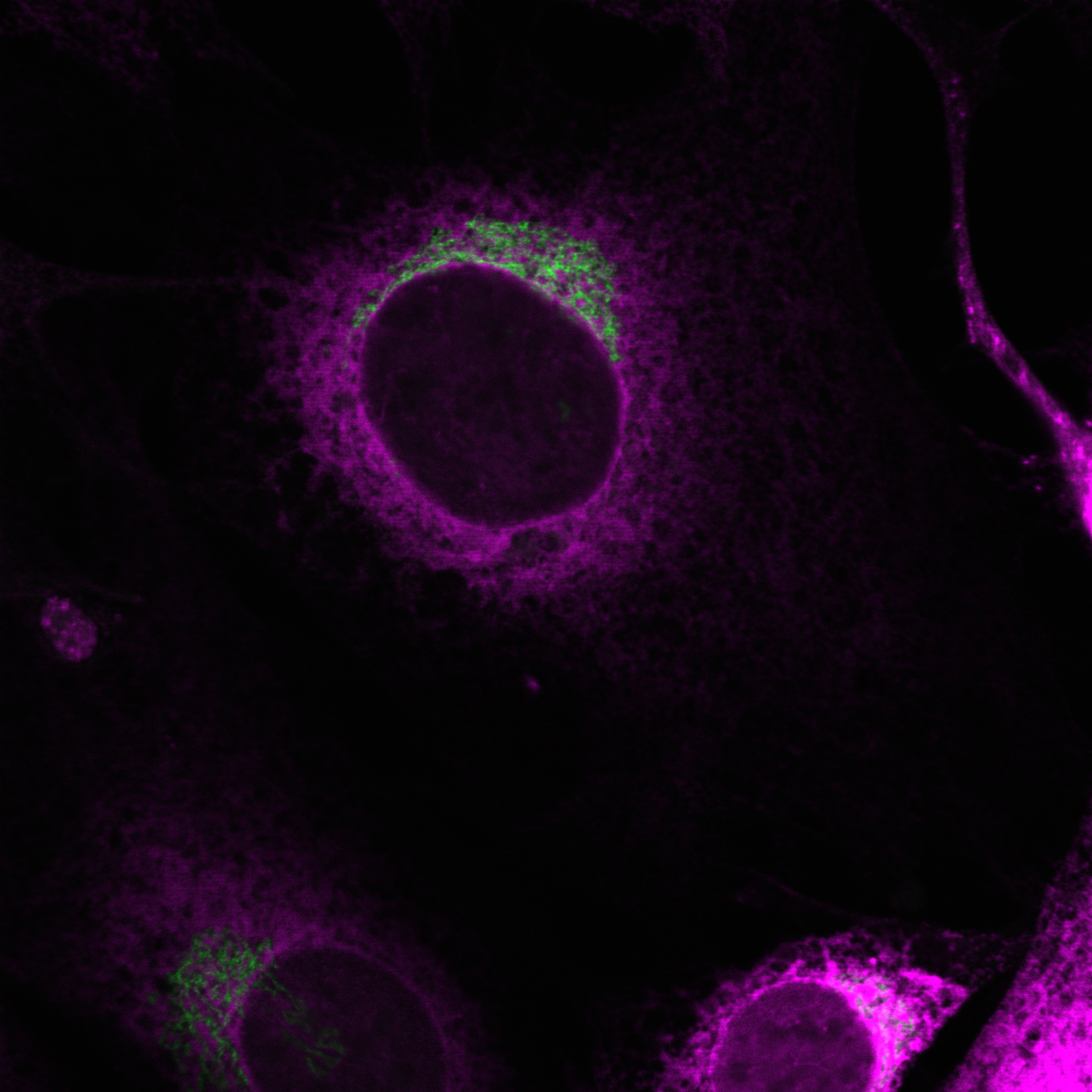

Supplement: Supplementary file 13 — EV and Appendix Figure Source Data [file 44318_2024_131_MOESM13_ESM.zip › ExpandedFigure 3/EV3B/FigureEV3B_EGFP-YIPF3WT_merge.tif]

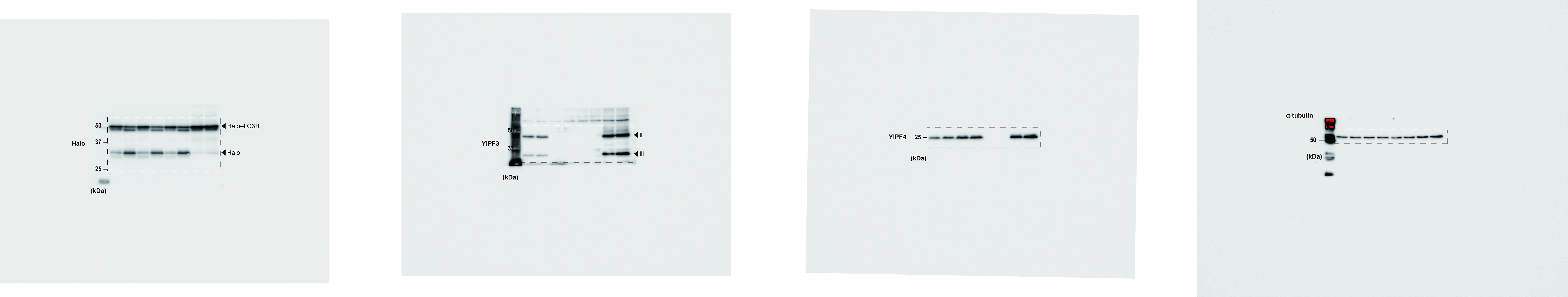

Supplement: Supplementary file 13 — EV and Appendix Figure Source Data [file 44318_2024_131_MOESM13_ESM.zip › ExpandedFigure 4/EV4A/western_FigureEV4A.tif]

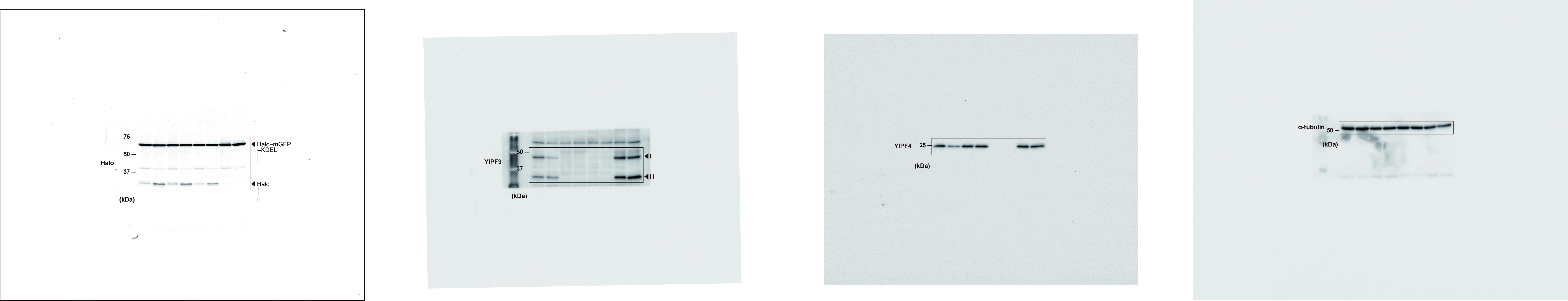

Supplement: Supplementary file 13 — EV and Appendix Figure Source Data [file 44318_2024_131_MOESM13_ESM.zip › ExpandedFigure 4/EV4C/western_FigureEV4C.tif]

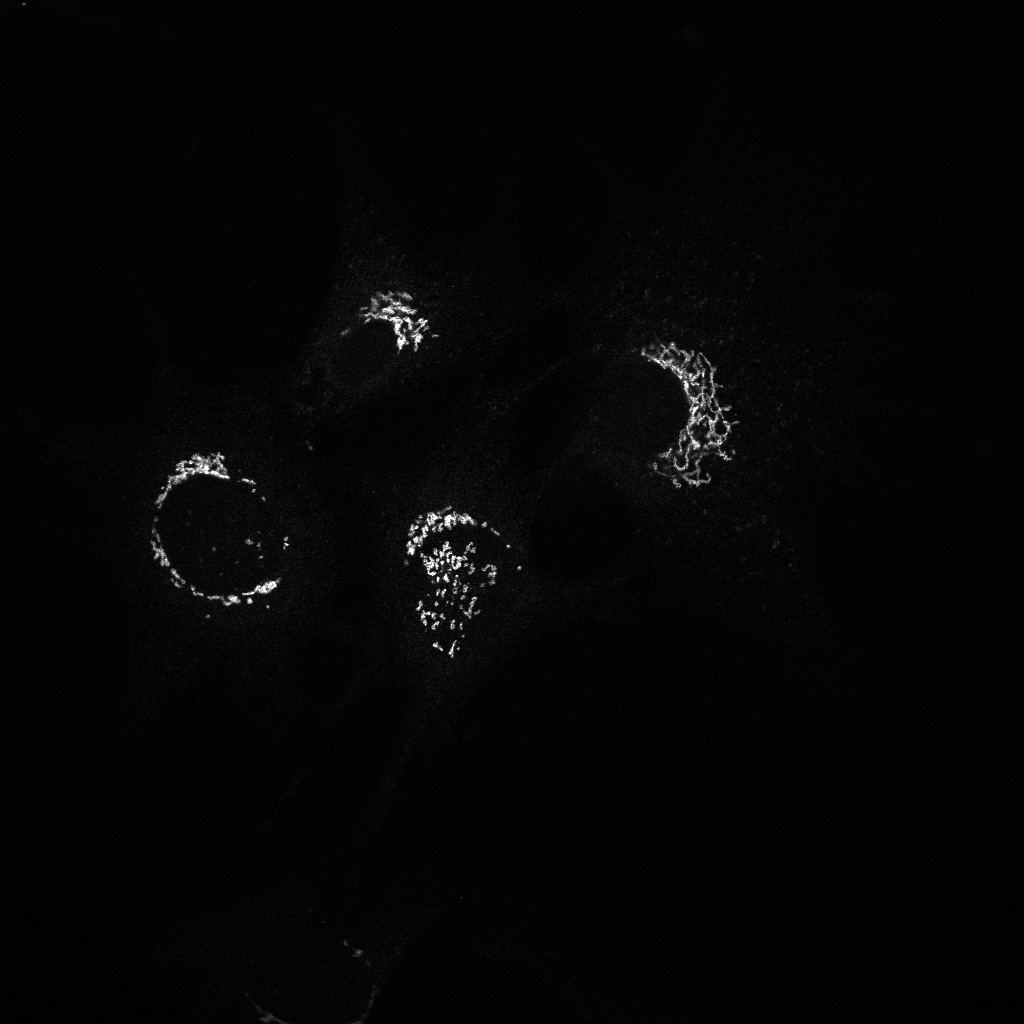

Supplement: Supplementary file 13 — EV and Appendix Figure Source Data [file 44318_2024_131_MOESM13_ESM.zip › ExpandedFigure 4/EV4E/FigureEV4E_EGFP-YIPF3_Growing_EGFP.tif]

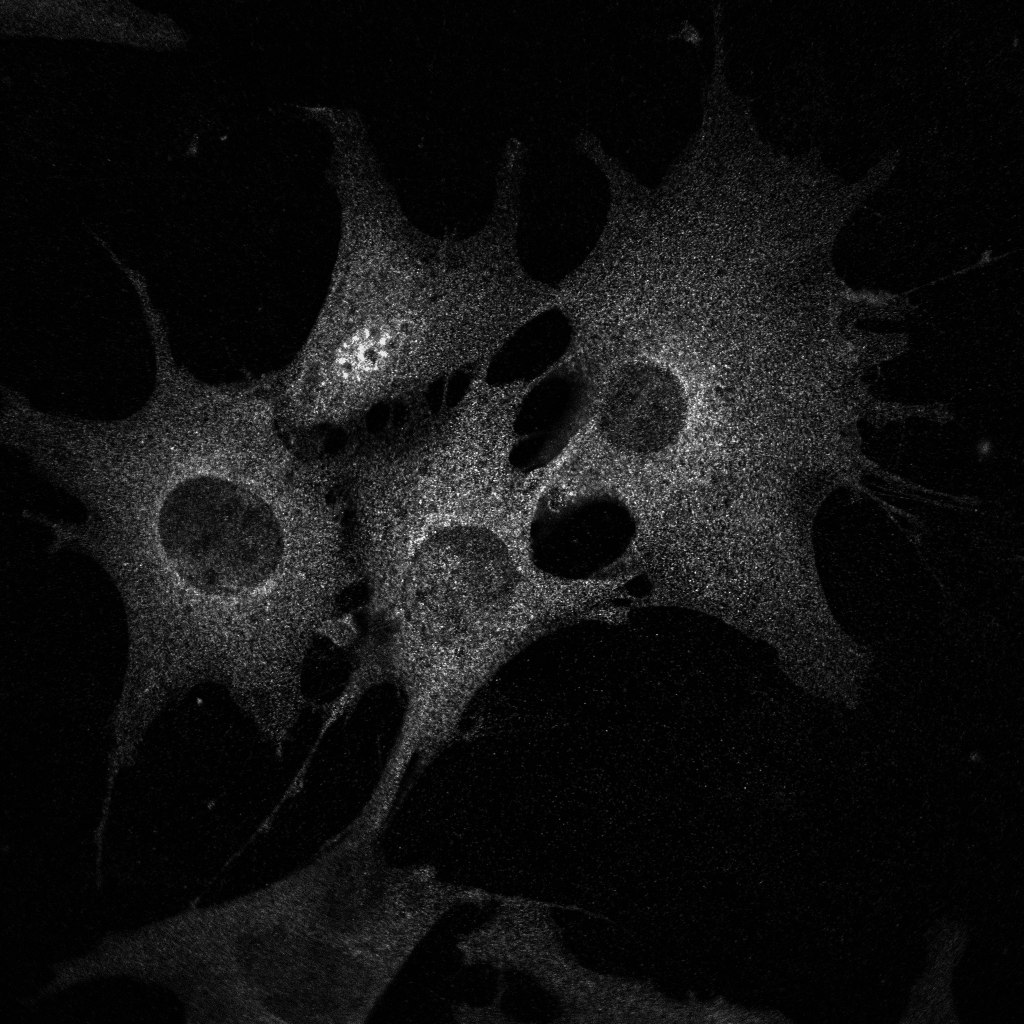

Supplement: Supplementary file 13 — EV and Appendix Figure Source Data [file 44318_2024_131_MOESM13_ESM.zip › ExpandedFigure 4/EV4E/FigureEV4E_EGFP-YIPF3_Growing_FIP200.tif]

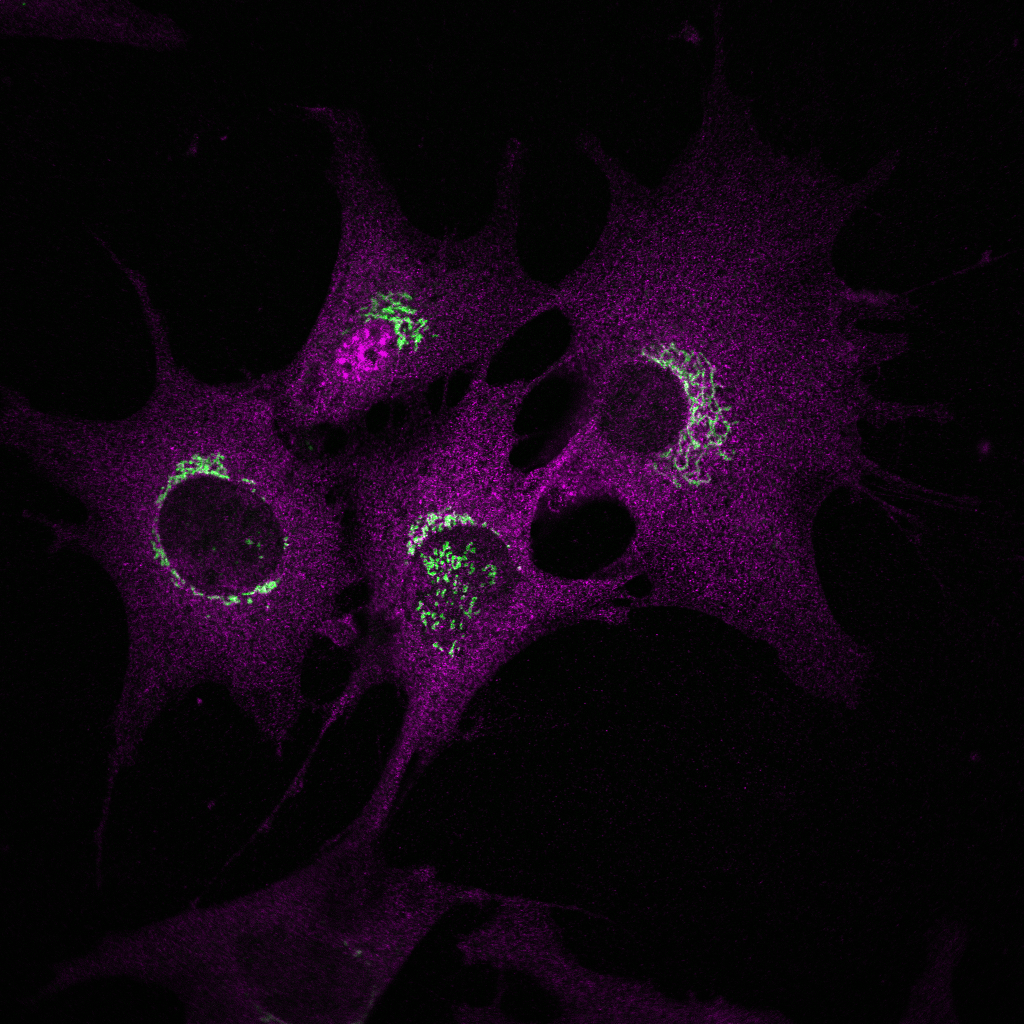

Supplement: Supplementary file 13 — EV and Appendix Figure Source Data [file 44318_2024_131_MOESM13_ESM.zip › ExpandedFigure 4/EV4E/FigureEV4E_EGFP-YIPF3_Growing_merge.tif]

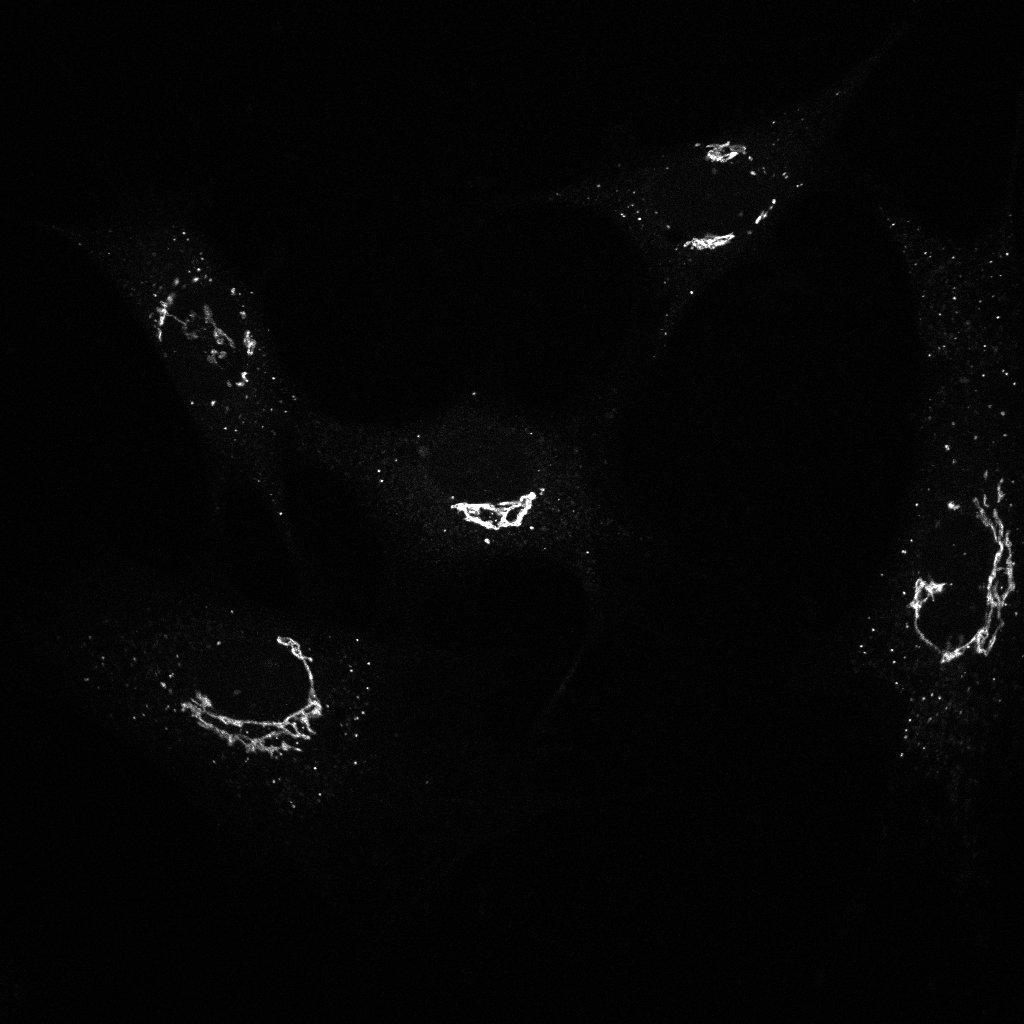

Supplement: Supplementary file 13 — EV and Appendix Figure Source Data [file 44318_2024_131_MOESM13_ESM.zip › ExpandedFigure 4/EV4E/FigureEV4E_EGFP-YIPF3_StarvationBafA1_EGFP.tif]

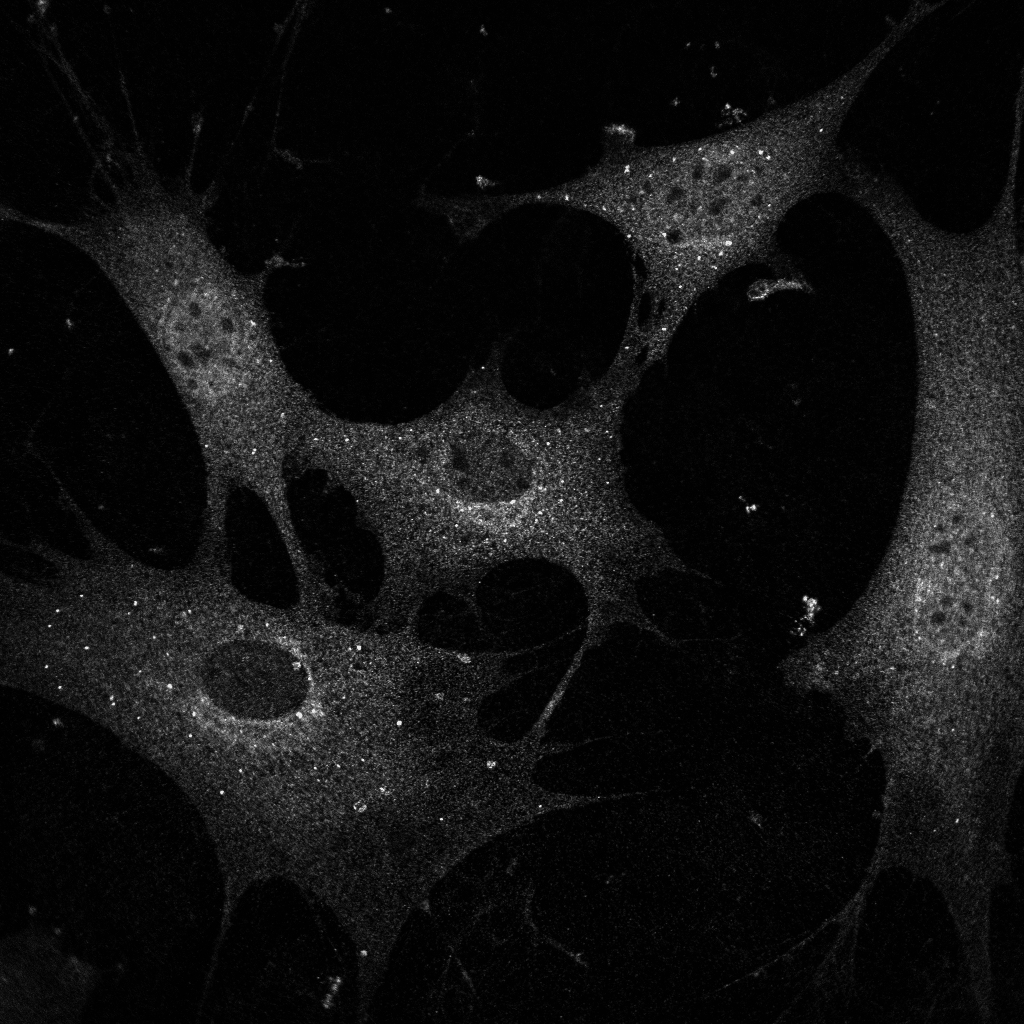

Supplement: Supplementary file 13 — EV and Appendix Figure Source Data [file 44318_2024_131_MOESM13_ESM.zip › ExpandedFigure 4/EV4E/FigureEV4E_EGFP-YIPF3_StarvationBafA1_FIP200.tif]

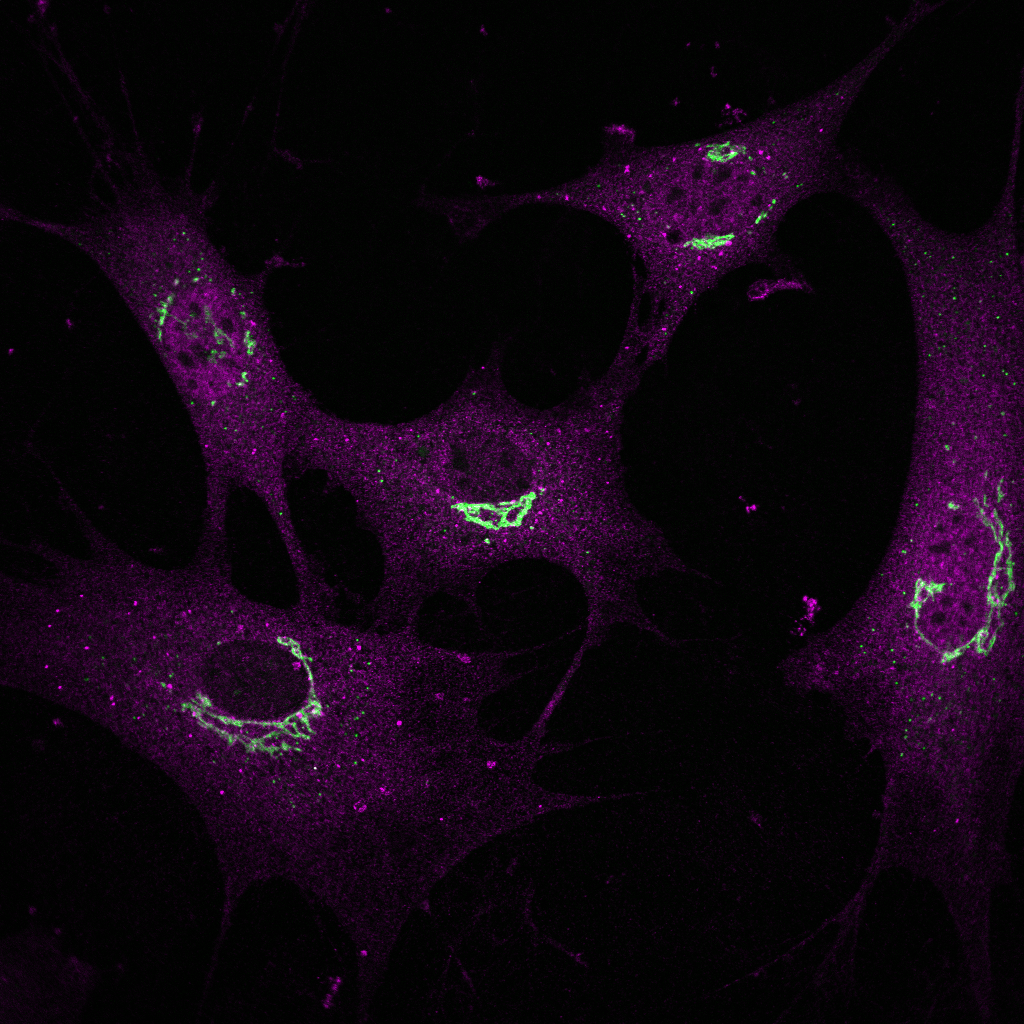

Supplement: Supplementary file 13 — EV and Appendix Figure Source Data [file 44318_2024_131_MOESM13_ESM.zip › ExpandedFigure 4/EV4E/FigureEV4E_EGFP-YIPF3_StarvationBafA1_merge.tif]

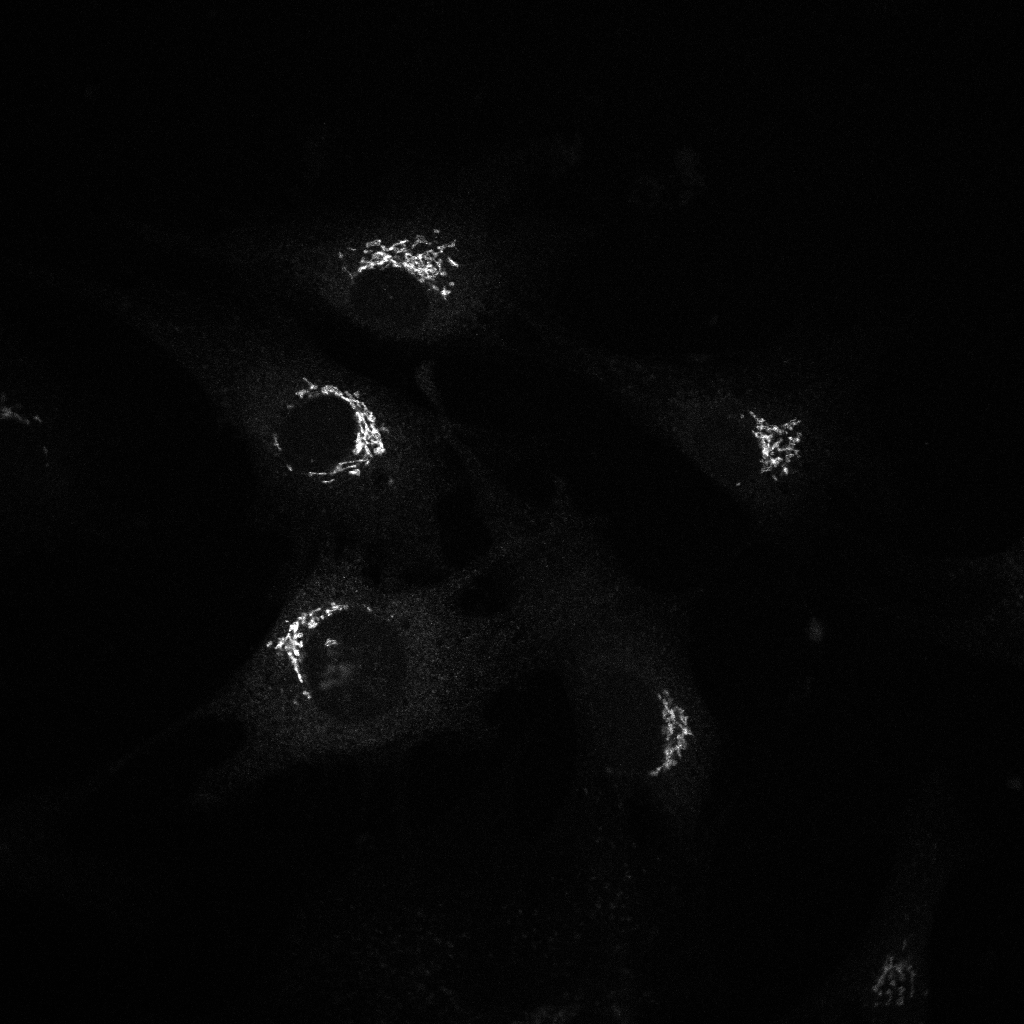

Supplement: Supplementary file 13 — EV and Appendix Figure Source Data [file 44318_2024_131_MOESM13_ESM.zip › ExpandedFigure 4/EV4E/FigureEV4E_EGFP-YIPF4_Growing_EGFP.tif]

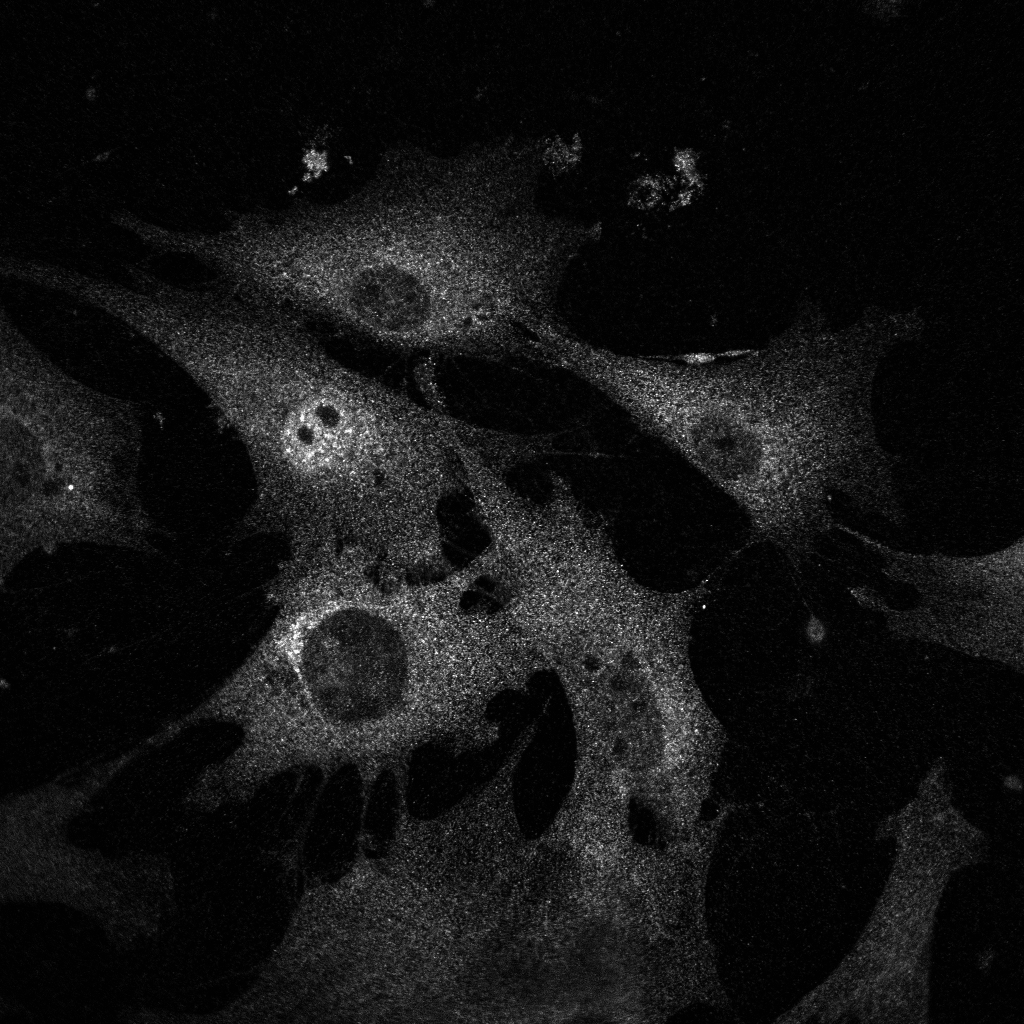

Supplement: Supplementary file 13 — EV and Appendix Figure Source Data [file 44318_2024_131_MOESM13_ESM.zip › ExpandedFigure 4/EV4E/FigureEV4E_EGFP-YIPF4_Growing_FIP200.tif]

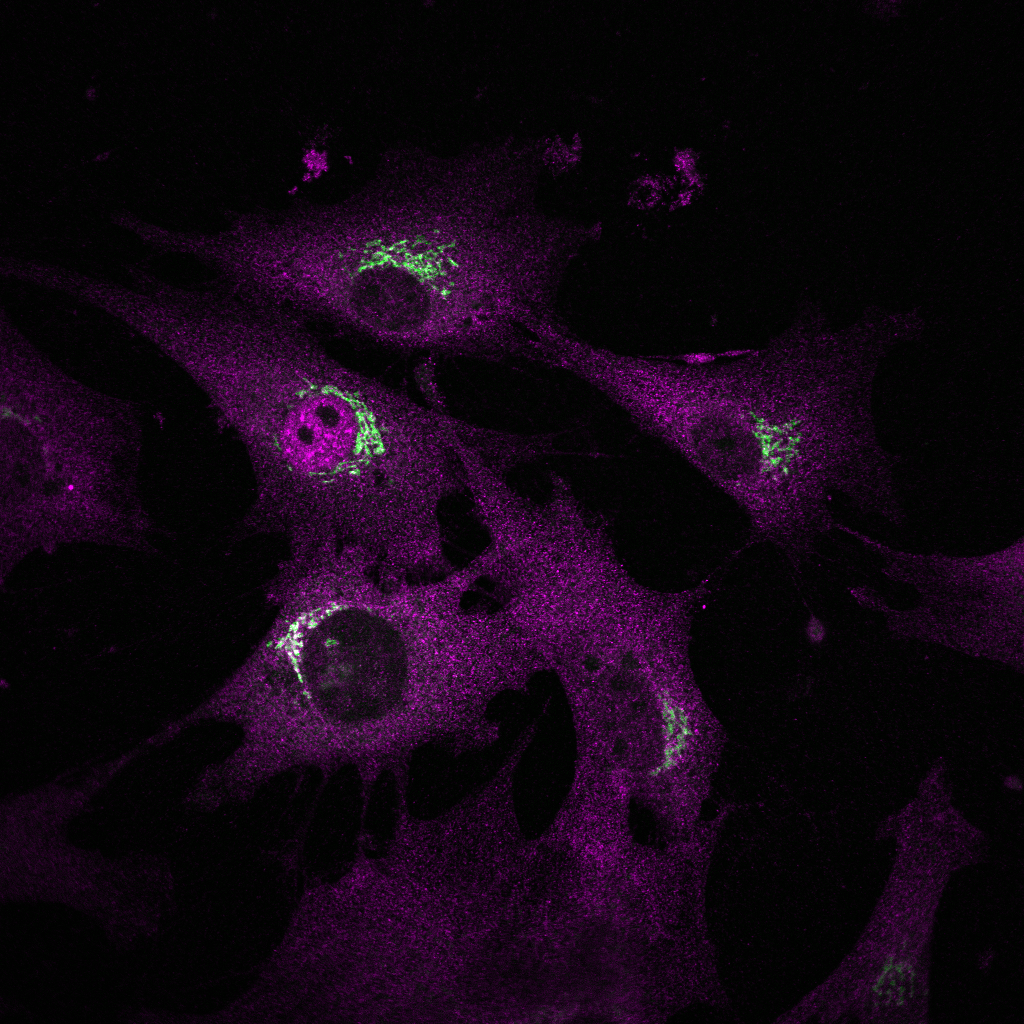

Supplement: Supplementary file 13 — EV and Appendix Figure Source Data [file 44318_2024_131_MOESM13_ESM.zip › ExpandedFigure 4/EV4E/FigureEV4E_EGFP-YIPF4_Growing_merge.tif]

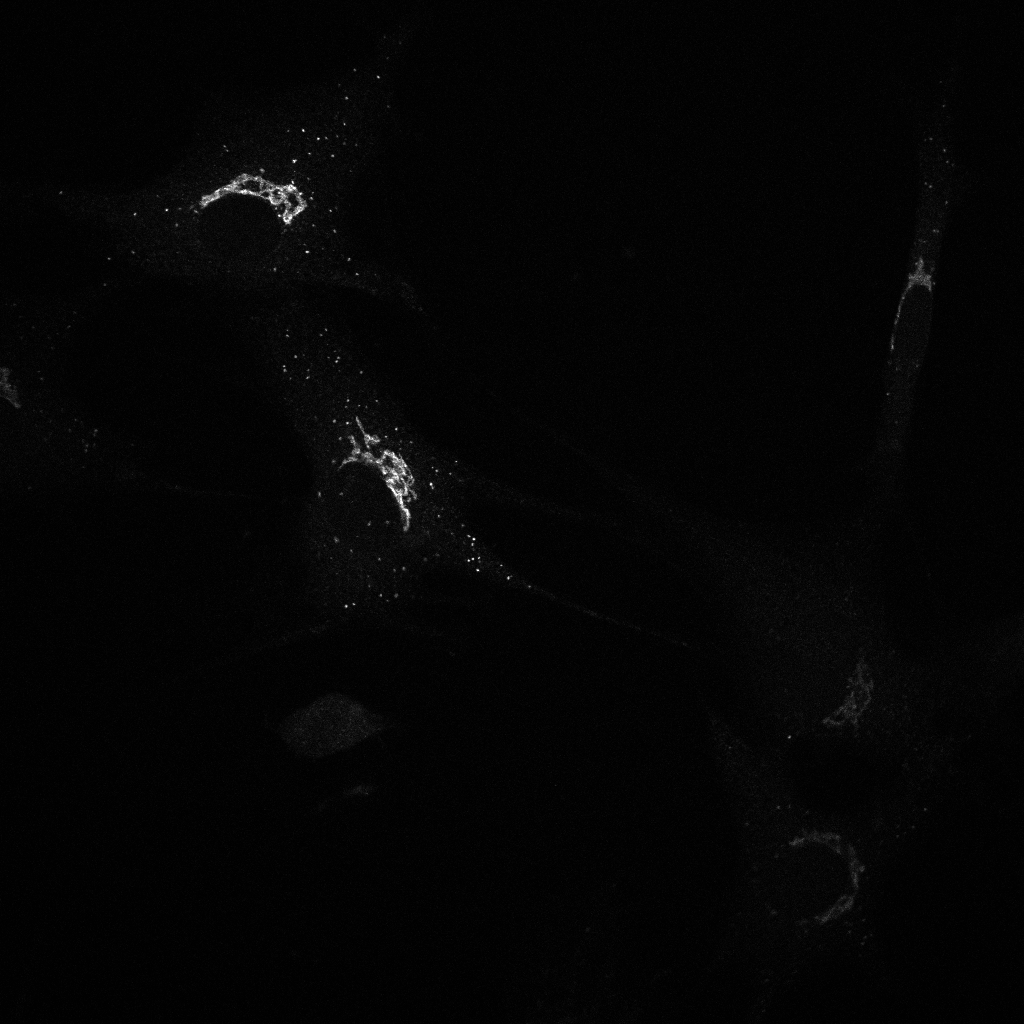

Supplement: Supplementary file 13 — EV and Appendix Figure Source Data [file 44318_2024_131_MOESM13_ESM.zip › ExpandedFigure 4/EV4E/FigureEV4E_EGFP-YIPF4_StarvationBafA1_EGFP.tif]

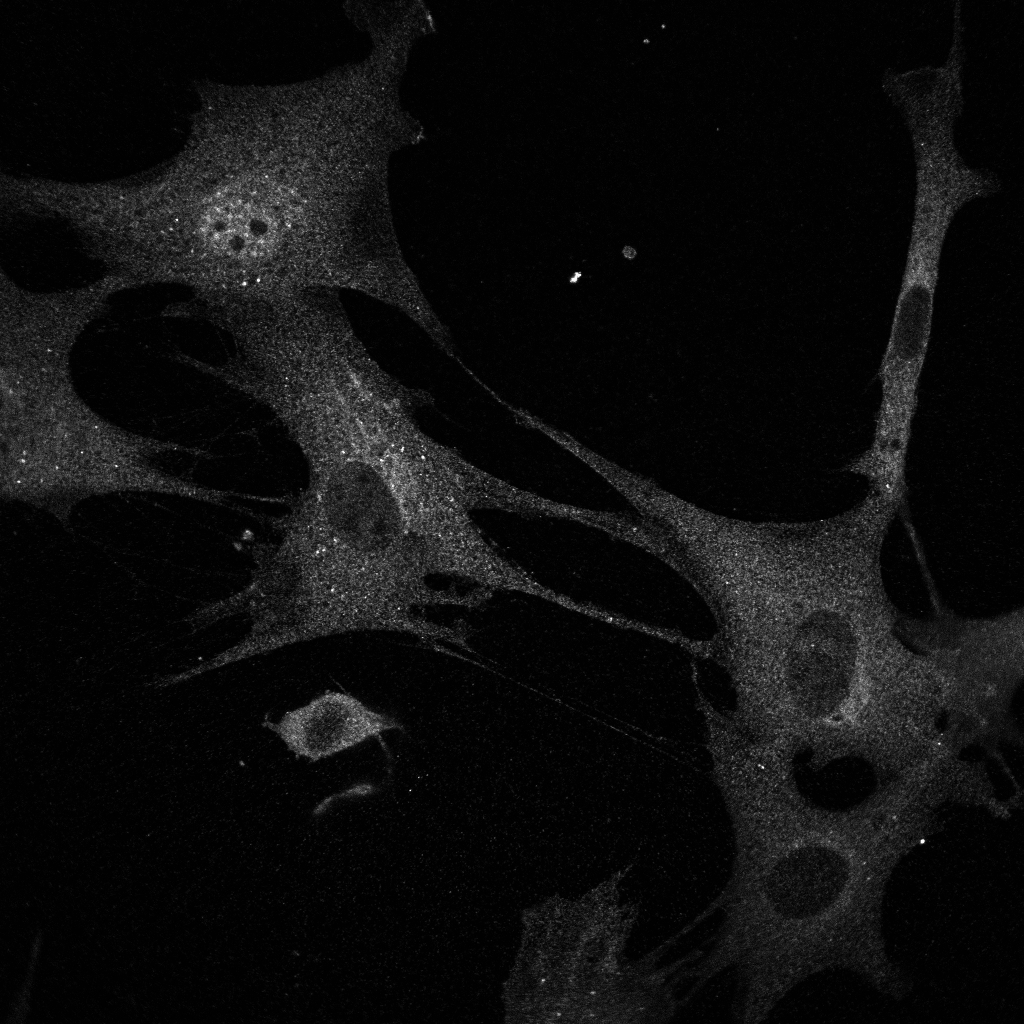

Supplement: Supplementary file 13 — EV and Appendix Figure Source Data [file 44318_2024_131_MOESM13_ESM.zip › ExpandedFigure 4/EV4E/FigureEV4E_EGFP-YIPF4_StarvationBafA1_FIP200.tif]

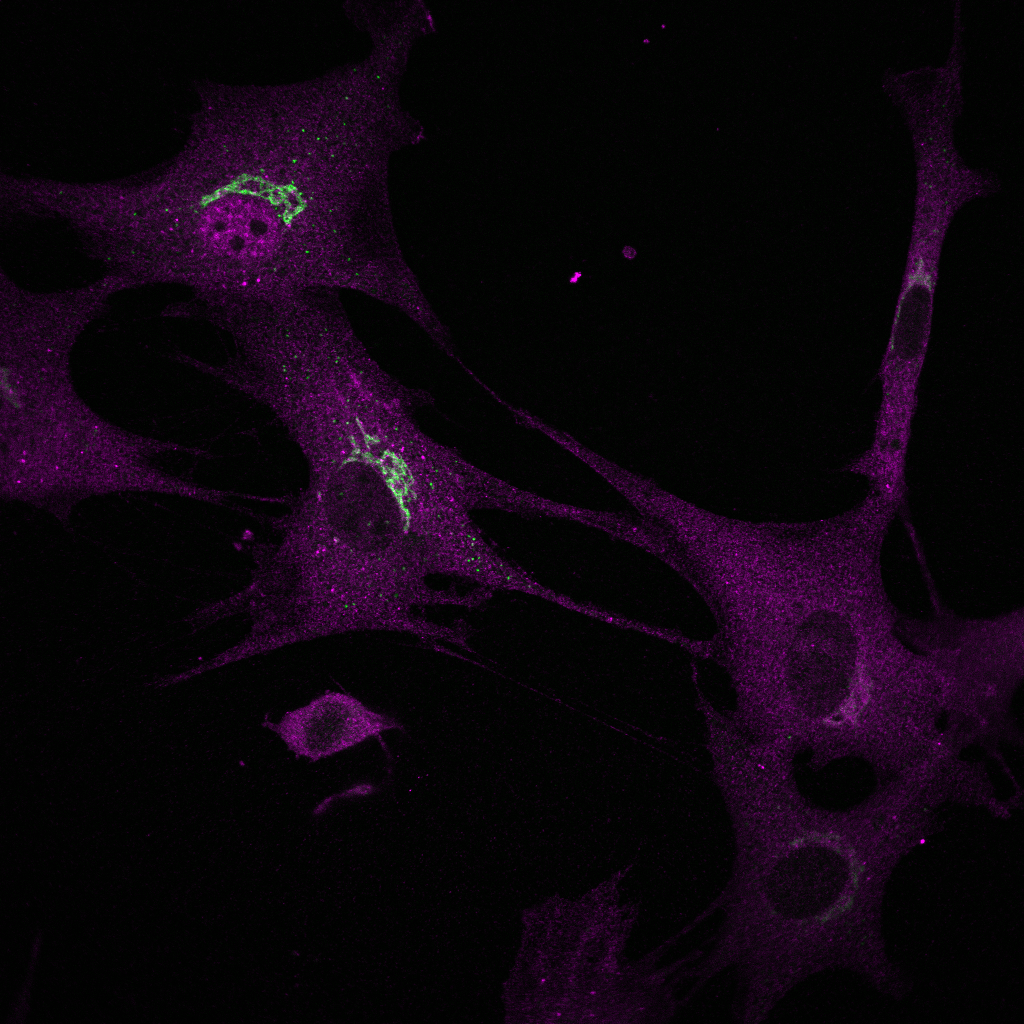

Supplement: Supplementary file 13 — EV and Appendix Figure Source Data [file 44318_2024_131_MOESM13_ESM.zip › ExpandedFigure 4/EV4E/FigureEV4E_EGFP-YIPF4_StarvationBafA1_merge.tif]

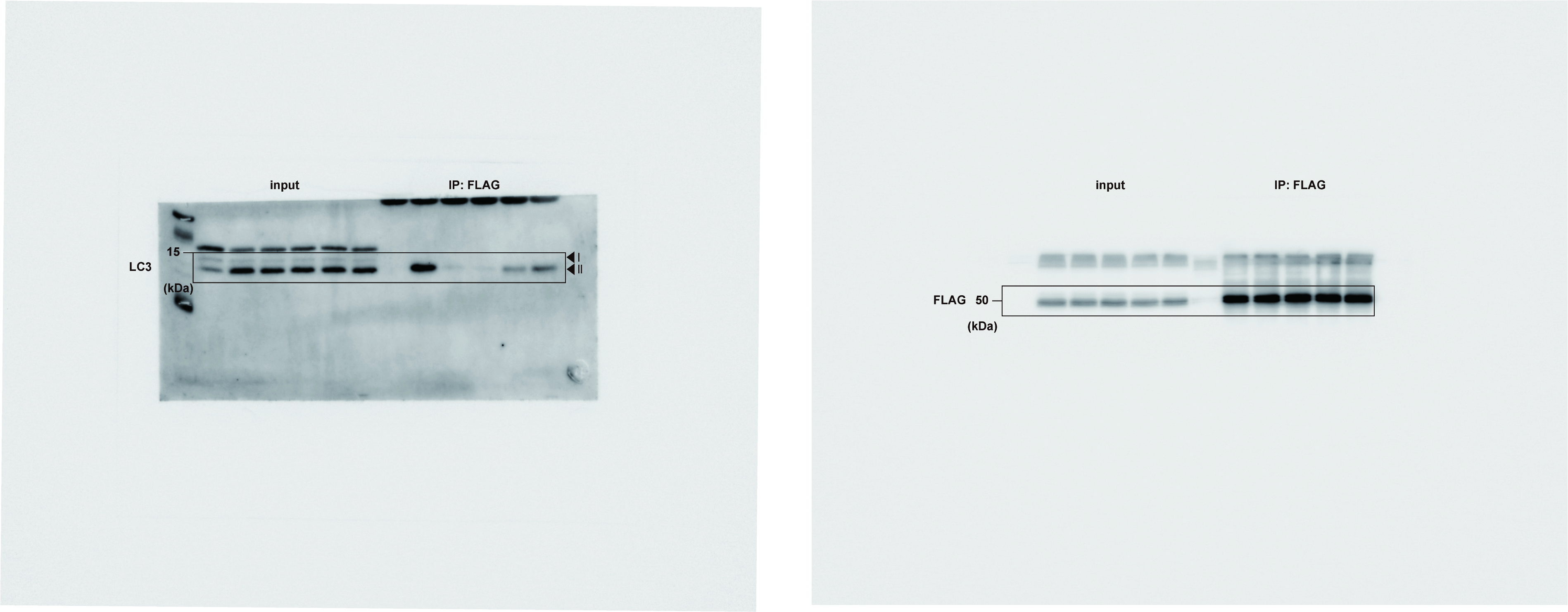

Supplement: Supplementary file 13 — EV and Appendix Figure Source Data [file 44318_2024_131_MOESM13_ESM.zip › ExpandedFigure 5/EV5/western_FigureEV5.tif]
