# Supplementary material for: YIPF3 and YIPF4 regulate autophagic turnover of the Golgi apparatus
Source: EMBO J. 2024 May 31;43(14):8. doi: 10.1038/s44318-024-00131-3 (PMC11250848; doi:10.1038/s44318-024-00131-3)
Supplement: Supplementary file 14 — Expanded View Figures [file 44318_2024_131_MOESM14_ESM.pdf]

Expanded View Figures

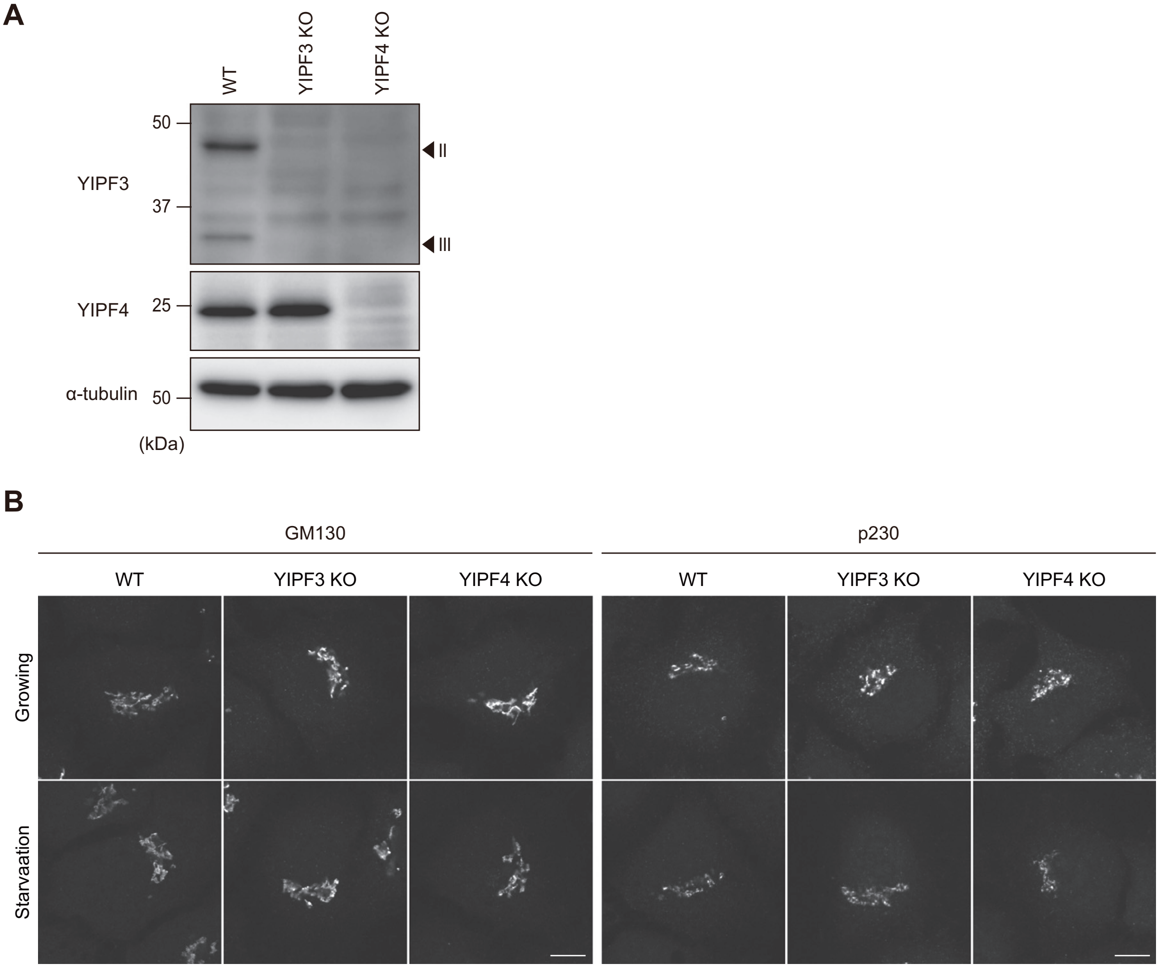

Figure EV1. Loss of YIPF4 suppresses YIPF3 protein levels.

(**A**) Immunoblotting of indicated proteins in WT, YIPF3-KO, and YIPF4-KO HeLa cells. (**B**) Immunofluorescence image of WT, YIPF3-KO and YIPF4-KO HeLa cells. Cells were cultured in DMEM or EBSS (9 h) and stained with anti-GM130 or anti-p230 antibodies. Scale bars, 10 μm. Source data are available online for this figure.

 © The Author(s)

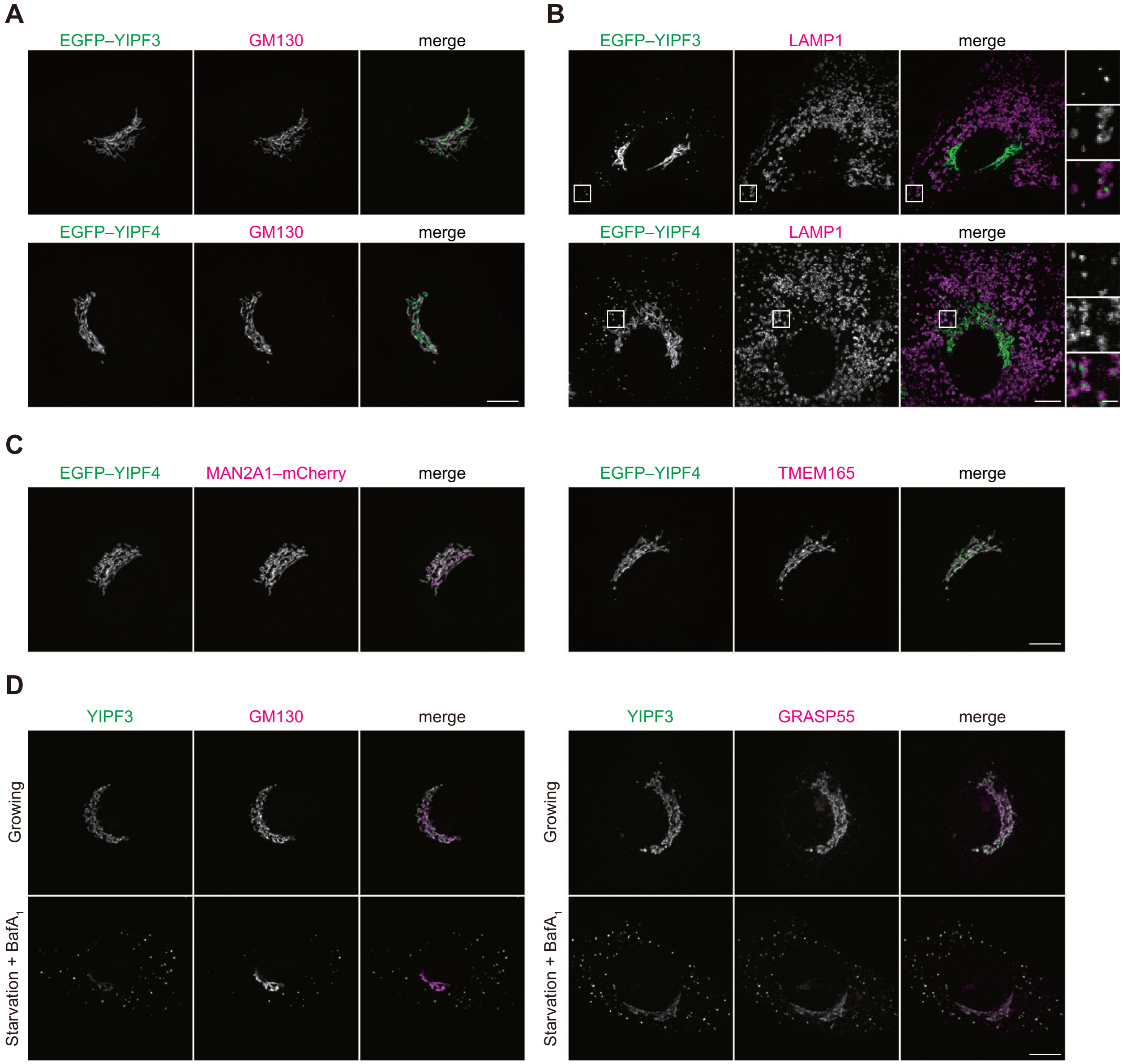

Figure EV2. EGFP-YIPF3 and EGFP-YIPF4 are localized to the Golgi apparatus under growing conditions, related to Fig. 2.

(**A**) Immunofluorescence image of MEFs stably expressing EGFP-YIPF3 or EGFP-YIPF4. Cells were cultured in DMEM and stained with anti-GM130 antibody. Scale bars, 10 µm and 1 µm (insets). (**B**) Immunofluorescence image of MEFs stably expressing EGFP-YIPF3 or EGFP-YIPF4. Cells were cultured in EBSS (2 h) in the presence of 125 nM Baf A_1 and stained with anti-LAMP1 antibodies. Scale bars, 10 µm and 1 µm (insets). (**C**) Immunofluorescence image of MEFs stably expressing EGFP-YIPF4 or coexpressing EGFP-YIPF4 and MAN2A1-mCherry. Cells were cultured in DMEM. The indicated cells were stained with anti-TMEM165 antibody. Scale bars, 10 µm. (**D**) Immunofluorescence image of MEFs cultured in DMEM or EBSS (6 h) in the presence of 125 nM Baf A_1. Each cell was stained with anti-YIPF3 antibody and the indicated cells were stained with anti-GM130 (*cis*-Golgi) or anti-GRASP55 (*medial*-Golgi) antibodies. Scale bars, 10 µm. The ratio of YIPF3 puncta positive for each Golgi protein to total YIPF3 puncta was quantified in three independent experiments (mean ± SD). Ten cells were analyzed per condition in each experiment. Source data are available online for this figure.

© The Author(s)

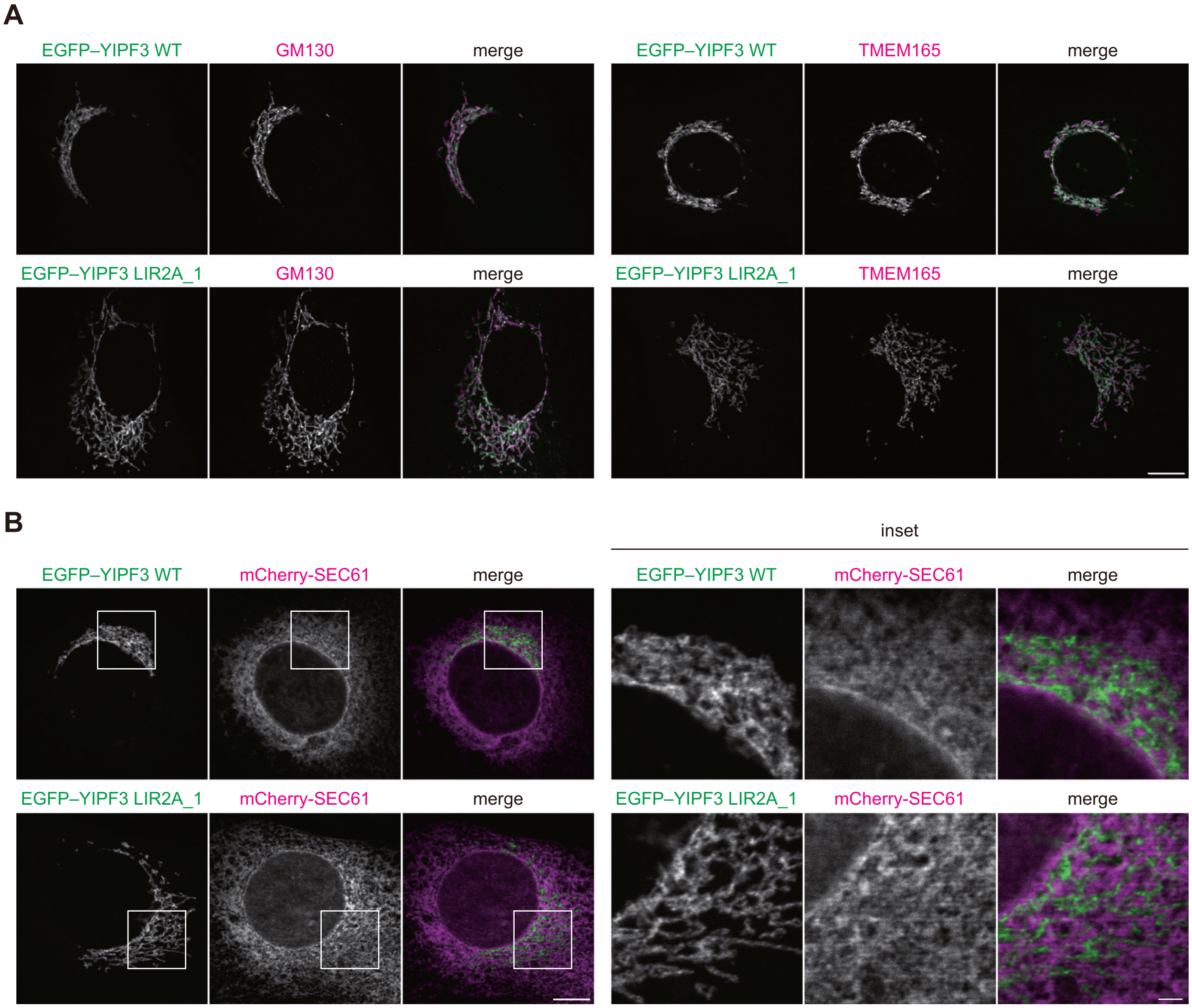

Figure EV3. The Golgi apparatus is partially discontiguous and expanded in cells stably expressing YIPF3 LIR2A mutant.

(**A**) Immunofluorescence image of MEFs stably expressing EGFP–YIPF3 WT or EGFP–YIPF3 LIR2A_1 under growing conditions. Cells were stained with anti-GM130 or anti-TMEM165 antibodies. Scale bars, 10 µm. (**B**) Immunofluorescence image of MEFs stably coexpressing EGFP–YIPF3 WT or EGFP–YIPF3 LIR2A_1 and mCherry-SEC61 under growing conditions. Scale bars, 10 µm and 2 µm (insets). Source data are available online for this figure.

 © The Author(s)

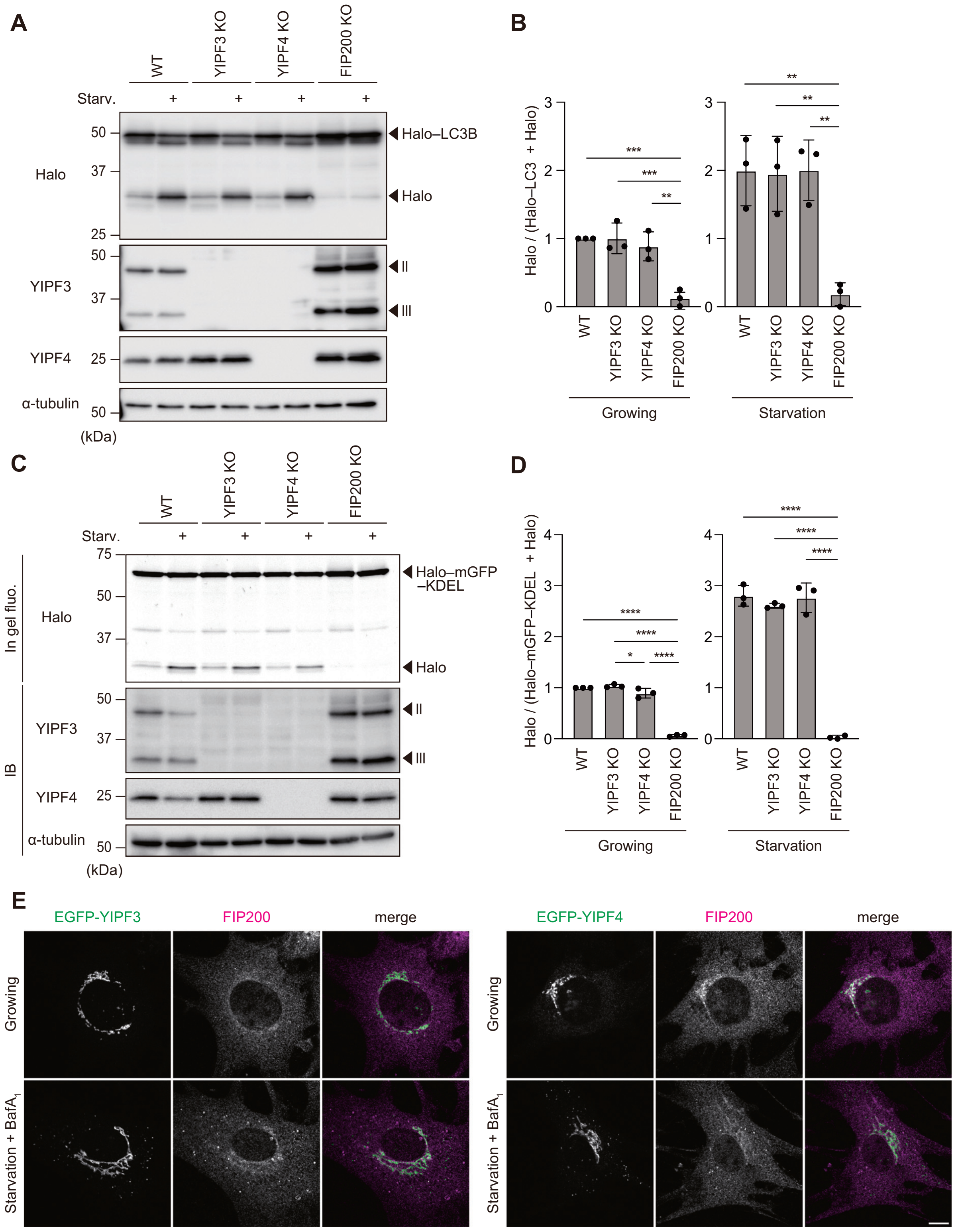
A
WT
YIPF3 KO
YIPF4 KO
FIP200 KO
Starv.
Halo
Halo–LC3B
YIPF3
II
III
YIPF4
α-tubulin
(kDa)
B
Halo / (Halo–LC3 + Halo)
Growing
Starvation
C
Halo–mGFP –KDEL
In gel fluo.
IB
D
Halo / (Halo–mGFP–KDEL + Halo)
E
EGFP-YIPF3
FIP200
merge
EGFP-YIPF4
Starvation + BafA1

Figure EV4. YIPF3 and YIPF4 are not involved in nonselective bulk autophagy and ER-phagy.

(**A**) Immunoblotting of indicated proteins in WT, YIPF3-KO, YIPF4-KO, and FIP200-KO HeLa cells stably expressing Halo–LC3B. After cells were cultured in the presence of TMR-conjugated ligands for 20 min and washed out, they were cultured in DMEM or EBSS for 4 h. (**B**) Quantification of results shown in (**A**). The ratio of Halo:Halo–LC3B is shown. Each ratio is normalized to WT cultured in nutrient-rich medium. Error bars indicate mean ± SD ($n = 3$ independent experiments). (**C**) In-gel fluorescence image of Halo and immunoblotting of indicated proteins in WT, YIPF3-KO, YIPF4-KO, and FIP200-KO HeLa cells stably expressing Halo–mGFP–KDEL. After cells were cultured in the presence of TMR-conjugated ligands for 20 min and washed out, they were cultured in DMEM or EBSS for 9 h. (**D**) Quantification of results shown in (**C**). The ratio of Halo:Halo–mGFP–KDEL is shown. Each ratio is normalized to WT cultured in nutrient-rich medium. Error bars indicate mean ± SD ($n = 3$ independent experiments). (**E**) Immunofluorescence image of MEFs stably expressing EGFP–YIPF3 or EGFP–YIPF4 cultured in DMEM or EBSS in the presence of 125 nM Baf A_1 for 2 h. Cells were stained with anti-FIP200 antibodies. Scale bars, 10 µm. Data information: *P* values were determined using one-way ANOVA with Tukey's multiple comparisons test (**B**, **D**). Symbols indicate: $*P \leq 0.05$; $**P \leq 0.01$, $***P \leq 0.001$, $****P \leq 0.0001$. Source data are available online for this figure.

 © The Author(s)

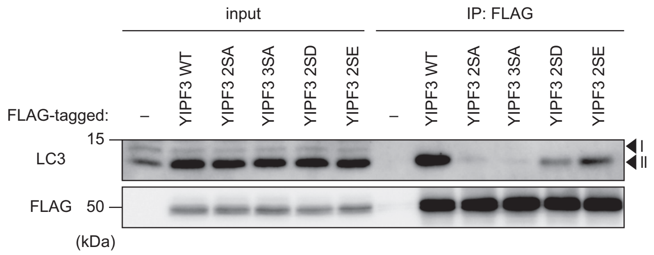

Figure EV5. YIPF3 phosphomimic mutants show reductions in their affinity to LC3.

Immunoprecipitation of FLAG–YIPF3, FLAG–YIPF3$^{S45A,\ S46A}$ (2SA), FLAG–YIPF3$^{S43A,\ S45A,\ S46A}$ (3SA), FLAG–YIPF3$^{S45D,\ S46D}$ (2 SD) or FLAG–YIPF3$^{S45E,\ S46E}$ (2SE) in HEK293T cells. Cells were transfected with FLAG-YIPF3 WT or each serine mutants. The lysates were immunoprecipitated with anti-FLAG antibody and detected with anti-LC3 and anti-FLAG antibodies. Source data are available online for this figure.

© The Author(s)
